# *Candida* species distribution, antifungal susceptibility and trends causing candidemia: a 10-year observation in eastern China

**DOI:** 10.7717/peerj.20832

**Published:** 2026-03-05

**Authors:** Peishan Cong, Biao Wang, Huiyang Qi, Guiming Zhang, Zhongjun Wang

**Affiliations:** 1 Department of Clinical Laboratory, Affiliated Hospital of Qingdao University, Qingdao, Shangdong, China; 2 Department of Clinical Laboratory, Peking University People’s Hospital, Qingdao, China; Women and Children’s Hospital, Qingdao University, Qingdao, China; 3 Department of Pathology, The Affiliated Hospital of Qingdao University, Qingdao, Shandong, China; 4 Department of Urology, The Affiliated Hospital of Qingdao University, Qingdao, China

**Keywords:** *Candida* species, Candidemia, Antifungal susceptibility, Risk factors

## Abstract

**Objective:**

Candidemia is a major global public health problem due to the high mortality rates and increasing antifungal resistance. This study investigated the epidemiology and risk factors of candidemia in eastern China over the past 10 years.

**Methods:**

A descriptive, cross-sectional study was conducted from January 2015 to December 2024 in the Affiliated Hospital of Qingdao University to investigate the possible routes and risk factors of candidemia. Antifungal susceptibility was determined using broth microdilution method.

**Results:**

The incidence of candidemia increased from 0.98% to 1.69% over the past 10 years. In total 276 candidemia cases, *Candida parapsilosis* was the most prevalent species (32.2%), followed by *Candida albicans* (31.9%) and *Candida tropicalis* (21.5%). *C. albicans* and *C. parapsilosis* were susceptible to antifungal agents (resistance rate <7%). *Candida tropicalis* and *Nakaseomyces glabratus* exhibited higher resistance rates to itraconazole (18.6% and 13%) and fluconazole (18.6% and 30.4%), respectively. Multivariate analysis identified hypertension (*P* < 0.001), diabetes mellitus (*P* = 0.042), chronic pulmonary diseases (*P* = 0.021), hematological malignancy (*P* = 0.012), solid organ tumor (*P* = 0.031), surgery (*P* < 0.001), prior antibiotic administration (*P* < 0.001) and intravascular catheters (*P* = 0.03), ICU admission (*P* < 0.001), mechanical ventilation (*P* = 0.02), and total parenteral nutrition (*P* < 0.001) as independent risk factors of candidemia.

**Conclusions:**

The incidence of candidemia has increased in recent years, with *C. parapsilosis* being the most prevalent species in candidemia. Effective strategies are needed to prevent candidemia from becoming a major concern.

## Introduction

The *Candida* genus is part of the body’s normal microbiota and primarily colonizes the skin, respiratory, gastrointestinal, and genital tracts. However, prolonged use of broad-spectrum antibiotics not only disrupts the integrity of the intestinal barrier but, when combined with corticosteroid-induced immunosuppression, creates a permissive microenvironment that facilitates *Candida* translocation into the bloodstream, increasing the risk of invasive candidiasis. Fungal pathogens and infections have become a growing global public health concern, and candidemia—specifically recognized as a life-threatening form of these infections—imposes a heavy burden of high mortality rates and hospital costs (Adam et al., 2021). An upward trend in candidemia infections has been noted due to the widespread use of broad-spectrum antibiotics, corticosteroids, immunosuppressants, anticancer therapy, and invasive devices, as well as solid organ transplantation (Thompson et al., 2024). In addition, tracheal intubation or tracheotomy and prior intensive care unit (ICU) hospitalizations could also increase the infection risk (Bassetti et al., 2017; Carbia et al., 2023).

The distribution of species causing invasive *Candida* infections varies among populations and geographical regions. Moreover, noticeable differences were observed in the proportions of *Candida* infections across hospital wards (Guinea, 2014). Candidemia caused by *Candida albicans* was the most frequently isolated (Pfaller et al., 2005). In addition, non-*albicans Candida* species, such as *Candida parapsilosis*, *Candida tropicalis*, and *Nakaseomyces glabratus* (formerly *Candida glabrata*), are increasingly being isolated from cases of candidemia, with growing antifungal resistance (Qiao et al., 2023). In 2022, the World Health Organization (WHO) released the “WHO Fungal Priority Pathogens List: Guidance on Research, Development and Public Health Actions” to systematically prioritize fungal pathogens, taking into account their unmet research and development needs and their significance in public health. As specified in the list, *Candida auris* (*Sakaguchia auris*, proposed) and *C. albicans* fall into the Critical Priority Group; *N. glabrata*, *C. tropicalis*, and *C. parapsilosis* are categorized under the High Priority Group; and *C. krusei* belongs to the Medium Priority Group (World Health Organization, 2022). The susceptibility of each *Candida* species to antifungal drugs varies. *C. albicans* harbors susceptibility to various antifungal drugs, while *N. glabratus* and *C. tropicalis* present reduced susceptibility to azoles. *C. krusei* is inherently resistant to fluconazole (FLC) (Tortorano et al., 2021; Boonsilp et al., 2021) *C. auris* is resistance to numerous antifungal drugs elevated the mortality risk and led to outbreaks in Brazil (Hou et al., 2017; De Almeida et al., 2021).

The fungal resistance intensifies the challenges in the clinical treatment of invasive *Candida* infections (Cetinkaya et al., 2023). Azoles, as first-line antifungal drugs, are widely used in clinical treatment. Nonetheless, the efficacy of azoles is compromised in the treatment of *N. glabratus* and *C. tropicalis* due to their reduced susceptibility. *C. krusei* was increasingly resistant to fluconazole, too (Bilal et al., 2022). Hence, numerous clinical guidelines have recommended echinocandins as the first-line antifungal agents (Tortorano et al., 2021). The emergence of fluconazole-resistant *C. parapsilosis*, fluconazole-, voriconazole (VRC)- and echinocandin-resistant *N. glabratus* strains, and the multi-antifungal-resistant *Candida auris* (*Sakaguchia auris*, proposed) poses formidable challenges to the effective clinical management of *Candida* infections (Pfaller et al., 2019; Castanheira et al., 2020). Despite improvements in disease diagnosis and the introduction of new antifungal drugs, the incidence of candidemia continues to rise (Domán et al., 2024).

Candidemia is a severe infection caused by the *Candida* genus and may cause multi-organ dysfunction. In recent years, the incidence rate of candidemia has gradually increased (Bilal et al., 2022). Consequently, quantifying serum BDG levels can predict the presence of invasive *Candida* spp*.* infection. The advantage of BDG-guided preemptive therapy is significant in reducing unnecessary antifungal treatments, which not only helps avoid adverse effects but also limits the development of drug resistance. Given an approximately 54% BDG diagnostic positivity rate for invasive fungal infections, further validation across different clinical settings is important to refine its role in clinical practice (Rouzé, Estella & Timsit, 2022).

In this study, we collected *Candida* strains isolated from clinical bloodstream infections at the Affiliated Hospital of Qingdao University between January 2015 and December 2024. We analyzed the species distribution, antifungal drug susceptibility, associated risk factors, morbidity rates, and the diagnostic value of the BDG test for candidemia. This research aims to provide a foundation for effective treatment strategies for candidemia and to support efforts in preventing the spread of drug-resistant *Candida*.

## Materials & Methods

### Ethics statement

This project was approved by the Ethics Committee of the Affiliated Hospital of Qingdao University (QYFY WZLL 29011). All participants provided written informed consent to participate.

### Isolates collection and study design

Between January 2015 and December 2024, a total of 3,838 bloodstream infections were recorded at the Affiliated Hospital of Qingdao University. During the same period, 276 non-duplicate *Candida* isolates were collected from patients diagnosed with candidemia. A retrospective analysis was conducted to identify these cases across ward distribution, the *Candida* species distribution, antifungal susceptibility profiles, and the changes in BDG assay results throughout the therapeutic course.

### Blood culture and *Candida* spp. identification

Blood cultures were performed using the BACTEC™ FX Automated Blood Culture System (BD Biosciences, Franklin Lakes, NJ, USA). Following a positive culture result, the culture bottle was removed, and the liquid medium was smeared for Gram staining. The medium was then inoculated onto 5% sheep blood agar, MacConkey agar, and chocolate agar plates. When yeast was observed microscopically on the smear, the blood culture bottles were further inoculated onto Sabouraud dextrose agar. Cultures were incubated at 35 °C for 24–48 h. Species identification of isolated colonies was performed using MALDI-TOF mass spectrometry (Bruker Daltonics, Bremen, Germany).

Additionally, clinical data, including age, sex, and ward information for these patients, were recorded.

### Antifungal susceptibility testing

The broth microdilution method (BMD) was employed for *Candida* susceptibility testing according to the Clinical and Laboratory Standards Institute (CLSI). Clinical breakpoints (CBPs) and epidemiological cutoff values (ECVs) were determined according to CLSI guidelines and are presented in Supplement 1; they were applied as described. Quality control strains, including *C. albicans* ATCC 90028, *C. parapsilosis* ATCC 22019, and *C. krusei* ATCC 6258, were used, with quality control for susceptibility and species identification performed weekly. Sabouraud dextrose agar and Columbia blood agar plates were acquired from Zhengzhou Antobio Co., Ltd. (Zhengzhou, China), while *Candida* chromogenic medium was obtained from Jinan Babio Co., Ltd. (Jinan, China).

### (1,3)-β-D-glucan (BDG) assay

BDG levels in patients’ serum were measured using the BDG assay kit (Zhanjiang A & C Biological LTD., Zhanjiang, China), by photometric analysis. The BDG assay was performed strictly according to the instructions provided in the BDG test kit manual. The cutoff value was 100.5 pg/mL, as recommended by the manufacturer.

### Statistical analysis

SPSS 22.0 software (IBM, Armonk, NY, USA) was used for statistical analysis. The association between categorical variables and group data was analyzed using the chi-square test. For non-normally distributed quantitative data, results were presented as medians with interquartile ranges. Variables related to bloodstream infections that were significant in the univariate analysis were entered into a multivariate logistic regression to identify independent risk factors. *P*-values less than 0.05 were considered statistically significant.

## Results

### Incidence trend of candidemia

**Figure 1:**
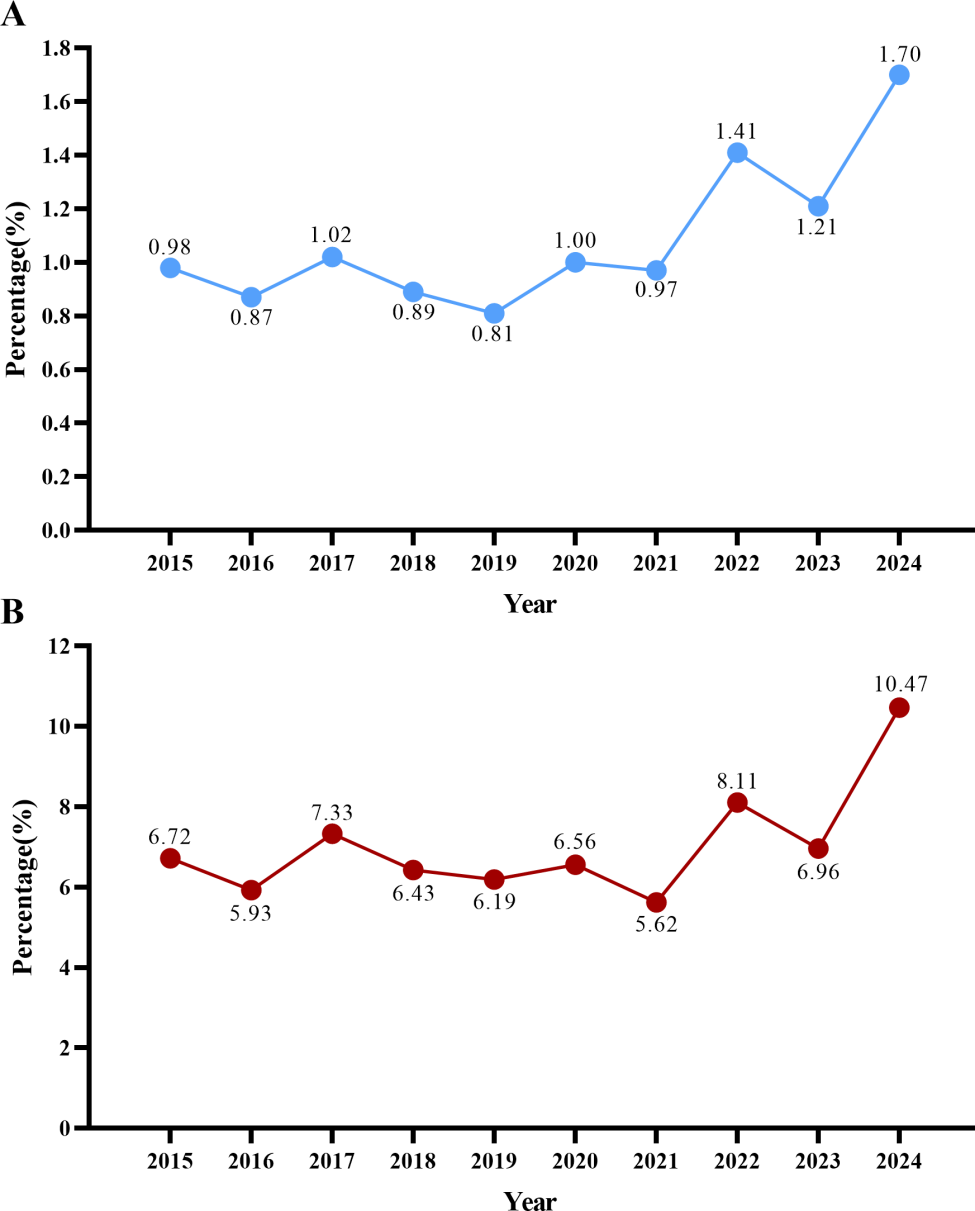
The positive rate of *Candida.* spp. (A) The positive rate of *Candida* spp. separating from bloodstream infections, (B) the proportion of *Candida* spp. in pathogens isolated from blood culture.

In 2015, 22 *Candida* spp. isolates separated from 2,245 blood culture samples, showing a positivity rate of 0.98% (22/2,245). In the subsequent years, the incidence continued to increase, reaching approximately 1.69% (50/2,959) by 2024. In 2015, a total of 327 pathogens were isolated from blood cultures, of which 22 were *Candida* strains, accounting for 6.72% (22/327). In recent years, the proportion of *Candida* spp. among blood culture-isolated pathogens has increased steadily, reaching nearly 11% by 2024 (Table 1, Fig. 1).

**Table 1:** The positive rate of *Candida spp* in blood culture.

Year	Blood culture (N)	Positive-Blood culture (N)	*Candida* spp. (N)	Candidemia of bloodstream infections rate (%)	*Candida*/pathogen isolated from blood culture (%)
2015	2,245	327	22	0.98	6.72
2016	2,184	320	19	0.87	5.93
2017	2,427	341	25	1.03	7.33
2018	2,472	342	22	0.89	6.43
2019	2,469	323	20	0.81	6.19
2020	2,626	396	26	0.99	6.56
2021	2,062	356	20	0.97	5.62
2022	2,766	481	39	1.41	8.11
2023	2,727	474	33	1.21	6.96
2024	2,959	478	50	1.69	10.47

### Distribution of *Candida* species isolated from blood cultures

Between January 2015 and December 2024, a total of 3,838 positive blood culture cases were recorded at our hospital, of which 276 non-duplicate *Candida* isolates were extracted, accounting for 7.2% of all blood culture-positive cases (Table 1). *C. parapsilosis* was the most frequently isolated species (89 isolates, 32.2%), followed by *C. albicans* (88 isolates, 31.9%), and *C. tropicalis* (59 isolates, 21.5%). *N. glabratus* ranked fourth (23 isolates, 8.3%). Other isolates included *C. famata* (*n* = 6, 2.2%), *Meyerozyma guilliermondii* (*n* = 4, 1.4%), *C. rugosa* (*n* = 2, 0.7%), *C. krusei* (*n* = 2, 0.7%), *C. intermedia* (*n* = 1, 0.4%), *C. dubliniensis* (*n* = 1, 0.4%), and *C. norvegensis* (*n* = 1, 0.4%). The details are presented in Table 2.

**Figure 2:**
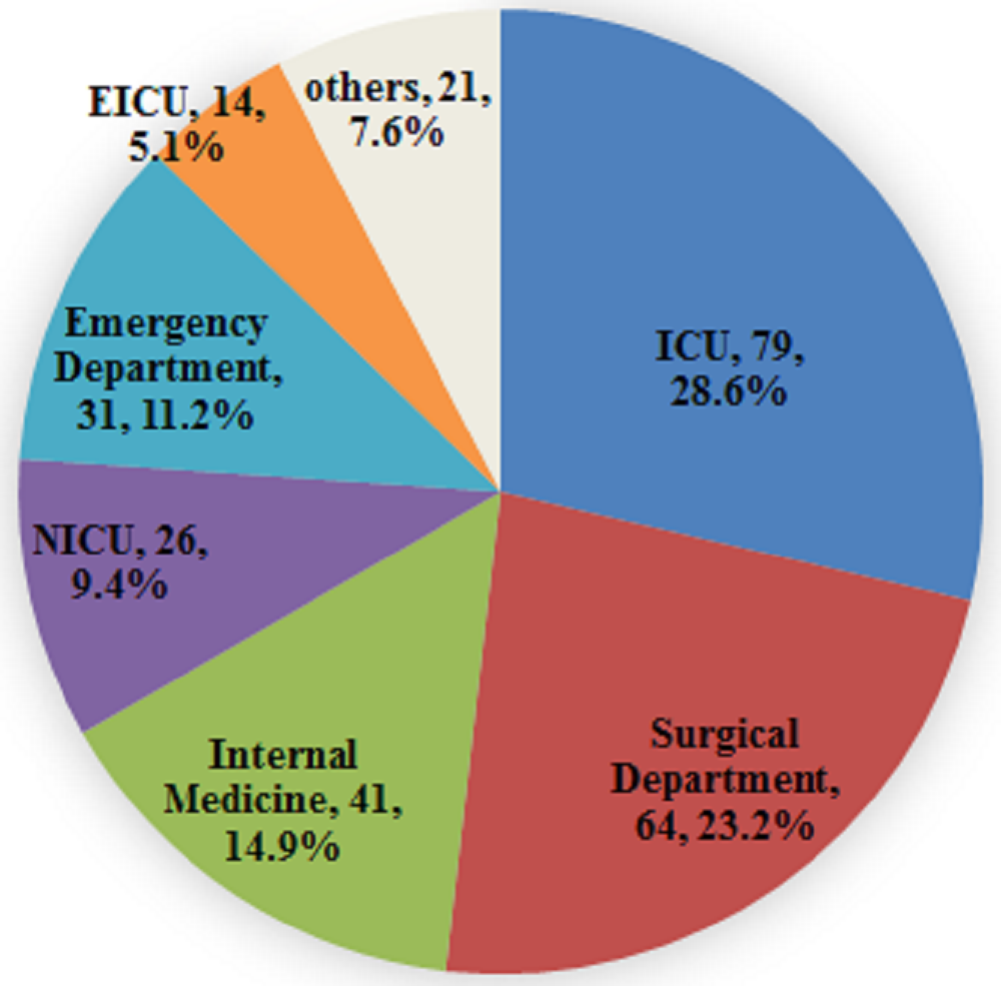
Distribution of candidemia patients by clinical service.

**Figure 3:**
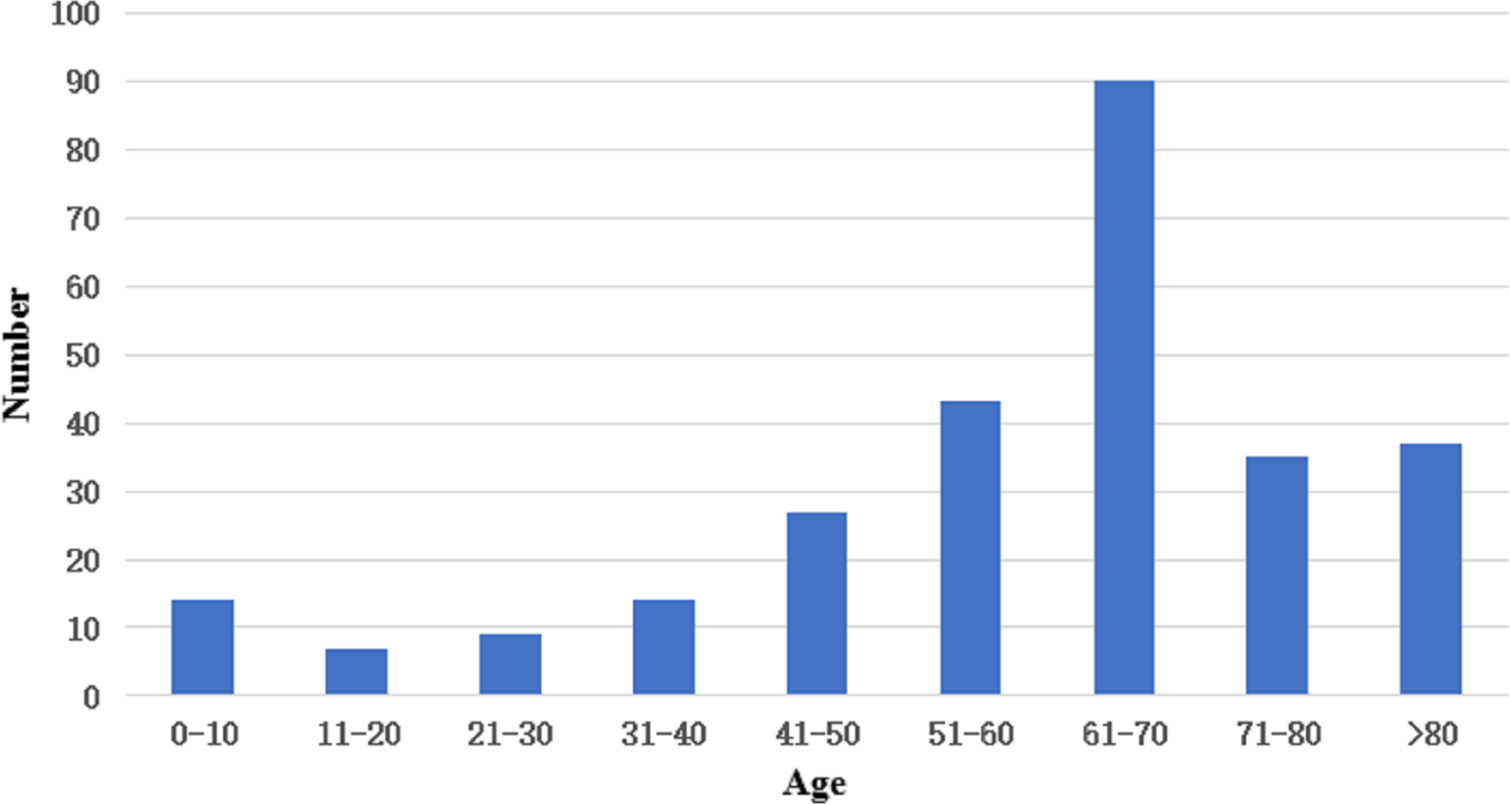
Distribution of candidemia patients by age.

Of the 276 cases of *Candida* infections identified through blood culture, isolates from the ICU accounted for 28.6% (79/276), followed by the Surgical Department (64 cases, 23.2%), Internal Medicine (41 cases, 14.9%), and the Emergency Department (31 cases, 11.2%). The Neurological ICU and Emergency ICU contributed 9.4% (26/276) and 5.1% (14/276), respectively. The remaining cases from other wards comprised 7.6% (21/276) (Fig. 2). Among the 276 patients with candidemia, 177 were male (64.1%), and 99 were female (35.9%). Candidemia occurred across a wide age range, from 20 days to 91 years, with a median age of 62 years (interquartile range, 50.5–72 years). Notably, 74.3% of patients were aged 50 years or older (Fig. 3).

**Table 2:** Univariate analysis of the factors influencing candidemia.

Factor	Candidemia group (N,%)	Control group (N,%)	OR	95% CI for Exp (B)	*P*-value
Gender (Female)	99 (35.9)	116 (42)	0.67	0.441–1.018	0.06
Underlying diseases					
Hypertension	179 (64.9)	129 (46.7)	2.352	1.553–3.562	<0.001
Diabetes Mellitus	84 (30.4)	65 (23.6)	1.629	1.017–2.608	0.042
Cardiovascular diseases	98 (35.5)	96 (34.8)	0.98	0.638–1.506	0.928
Chronic pulmonary diseases	107 (38.8)	75 (27.2)	1.687	1.084–2.626	0.021
Hematological malignancy	72 (26.1)	51 (18.5)	1.897	1.153–3.121	0.012
Solid organ tumor	87 (31.5)	56 (20.3)	1.689	1.05–2.717	0.031
Solid organ transplantation	11 (4.0)	7 (2.5)	2.053	0.64–6.586	0.226
Surgery	171 (62.0)	104 (37.8)	2.283	1.507–3.461	<0.001
Burns	3 (1.1)	2 (0.72)	2.117	0.254–17.672	0.488
Antibiotic administration prior to candidemia	246 (89.1)	201 (72.8)	2.887	1.679–4.962	<0.001
Antifungal administration prior to candidemia	72 (26.1)	57 (20.7)	1.538	0.947–2.499	0.082
Intravascular catheters	169 (61.2)	121 (43.8)	1.582	1.045–2.396	0.03
ICU admission	191 (69.2)	83 (30.10)	4.337	2.852–6.597	<0.001
Mechanical ventilation	166 (60.14%)	107 (38.8)	1.651	1.081–2.521	0.02
Total parenteral nutrition	120 (43.5%)	78 (28.3)	2.164	1.403–3.336	<0.001

**Notes.**

OR: Odds ratio; CI: Confidence Interval.

### Analysis of risk factors for Candidemia

Hypertension (64.9%), surgery (62.0%), chronic pulmonary disease (38.8%), and cardiovascular disease (35.5%) were the most common underlying diseases in candidemia patients. It is worth noting that 89.1% of patients had received antibiotics prior to developing candidemia, and 69.2% had been admitted to the ICU for at least 15 days before the blood culture results became positive. Surgical treatments had been performed in 171 (62.0%) patients. 169 (61.2%) patients used intravascular catheters, and 60.1% required mechanical ventilation. Nearly half of the patients with candidemia (43.5%) needed total parenteral nutrition. Additionally, 31.5% suffered from a solid organ tumor (Table 3).

**Table 3:** Distribution and antifungal susceptibility of *Candida* species causing candidemia.

Species	N (%)	Antifungal	MIC_50_	S/WT (%)	I/SDD (%)	R/n-WT (%)
*C. parapsilosis*	89 (32.2)	VRC	0.03	84 (94.4)	3 (3.4)	2 (2.3)
		FLC	0.5	81 (91.0)	3 (3.4)	5 (5.6)
		ITC	0.06	83 (93.3)	–	6 (6.7)
		AMB	0.25	89 (100)	0 (0)	0 (0)
		MCF	0.125	88 (98.9)	1 (1.1)	0
		CAS	0.125	88 (98.9)	1 (1.1)	0
*C. albicans*	88 (31.9)	VRC	0.03	81 (92.0)	5 (5.7)	2 (2.3)
		FLC	0.25	86 (97.7)	0 (0)	2 (2.3)
		ITC	0.06	–	–	–
		AMB	0.25	87 (98.9)	0 (0)	1 (1.1)
		MCF	0.03	88 (100)	0	0
		CAS	0.03	88 (100)	0	0
*C. tropicalis*	59 (21.5)	VRC	0.03	44 (74.6)	10 (16.9)	5 (8.5)
		FLC	1	46 (78.0)	2 (3.4)	11 (18.6)
		ITC	0.06	48 (81.4)	–	11 (18.6)
		AMB	0.25	59 (100)	–	–
		MCF	0.06	59 (100)	0	0
		CAS	0.06	59 (100)	0	0
*N. glabratus*	23 (8.3)	VRC	0.12	18 (78.3)	–	5 (21.7)
		FLC	32	–	16 (69.6)	7 (30.4)
		ITC	0.25	22 (95.7)	–	1 (13.0)
		AMB	0.25	23 (100)	–	0 (0)
		MCF	0.015	23 (100)	0	0
		CAS	0.03	22 (95.7)	1 (4.3)	0
*M. guilliermondii* (formerly *C. guilliermondii*)	4 (1.4)	VRC	–	–	–	–
		FLC	–	4	–	–
		ITC	–	4	–	–
		AMB	–	4	–	–
		MCF	–	4	0	0
		CAS	–	4	0	0
Candida spp.*	13 (4.7)	–	–	–	–	–

**Notes.**

*Candida* spp*.* ^∗^: *C. famata* (*n* = 6), *C. rugosa* (*n* = 2), *C. krusei* (*n* = 2), *C. intermedia* (*n* = 1), *C. dubliniensis* (*n* = 1), *C. norvegensis* (*n* = 1).

AMB Amphotericin B FLC fluconazole ITC itraconazole VRC voriconazole CAS caspofungin MCF micafungin WT wild type S susceptible “-” no breakpoint or ECV available

A comparative analysis of risk factors for candidemia was conducted. The infection group included patients with candidemia, while the control group consisted of patients from the same wards who did not develop candidemia. The results revealed that hypertension (*P* < 0.001), chronic pulmonary diseases (*P* = 0.021), hematological malignancy (*P* = 0.012), solid organ tumor (*P* = 0.031), surgery (*P* < 0.001), prior antibiotic administration >15 days (*P* < 0.001), and the use of intravascular catheters (*P* = 0.03), ICU admission (*P* < 0.001), mechanical ventilation (*P* = 0.02) and total parenteral nutrition (*P* < 0.001) were significantly more common in the infection group than in the control group. In contrast, no significant differences were observed in other factors (*P* > 0.05) (Table 2).

### Antifungal susceptibility testing results

The results of the antifungal susceptibility testing revealed that Amphotericin B (AMB) showed strong activity against common *Candida* isolates, with the rate of non-wild-type (nWT) or resistance being less than 2%. Except for one *N. glabrata* strain showing intermediate susceptibility to caspofungin (CAS), and one *C. parapsilosis* strain exhibiting intermediate susceptibility to both caspofungin (CAS) and micafungin (MCF), all other common *Candida* species were susceptible to echinocandins. Compared with *C. parapsilosis* and *C. albicans, C. tropicalis* and *N. glabratus* showed significantly higher resistance rates to voriconazole (8.5%, 21.7% *vs* 2.3%, 2.3%) and fluconazole (18.6%, 30.4% *vs* 5.6%, 2.3%), respectively. The resistance rates of *C. tropicalis* and *N. glabratus* to itraconazole (ITC) were also notable, at 18.6% (11/59) and 13.0% (1/23), respectively. For *C. parapsilosis,* the overall itraconazole nWT rate was only 6.7% (6/89) (Table 3).

### Outcomes of BDG assay in patients with candidemia

Among the 276 patients with candidemia, BDG assays were conducted on 118 patients (42.8%), within 7 days before to 14 days after the onset of symptoms. The assay yielded positive results in 84 cases, yielding a sensitivity of 71.2% and a specificity of 90%. Dynamic monitoring of BDG was implemented on 15 of the 49 positive individuals, revealing that antifungal therapy led to a marked decrease in BDG levels in 10 patients. This decrease was accompanied by improvement in clinical symptoms, indicating the effectiveness of the antifungal treatment and a favorable prognosis, leading to successful discharge. Conversely, five patients whose BDG levels initially decreased post-antifungal treatment but subsequently increased and remained elevated, presented a poor prognosis. These findings highlight the significant value of BDG assays for dynamic monitoring and for assessing the therapeutic outcomes in candidemia.

## Discussion

Our study found that the prevalence of candidemia has been steadily increasing over the years. The incidence rose from 0.98% in 2015 to 1.69% in 2024, which is slightly lower than the data in previous studies (Carbia et al., 2023). A study in Japan reported a candidemia incidence of 0.84% across over 2,000 hospitals between 2010 and 2019, which is marginally lower than our findings. This difference may be due to regional differences, hospital infection control strategies, and so on (Kajihara et al., 2022). The increase in candidemia incidences in recent years can be attributed to advances in diagnostic and therapeutic technologies, more invasive procedures, widespread use of broad-spectrum antibiotics, and immunocompromise caused by various underlying diseases (Tsay et al., 2017). Given the challenges in early diagnosis and the high mortality rate of candidemia, understanding the distribution of *Candida* species and their resistance patterns can provide essential guidance for empirical antifungal therapy.

In this study, we observed that non-albicans *Candida*, with *C. parapsilosis* surpassing *C. albicans*, emerged as the most prevalent species in bloodstream infections. This result is similar to the findings of several recent studies (Puig-Asensio et al., 2014a; Mamali et al., 2022). Conversely, a retrospective study spanning over 16 years at a center in Lebanon found that *C. albicans* had the highest isolation rate in bloodstream infections, followed by *C. tropicalis*. This finding was consistent with research data from the USA (Zakhem et al., 2021). A study from a hospital in China found that *C. tropicalis* accounted for the highest proportion of isolates (Li et al., 2015). Variation in species distribution may be attributed to differences in geographic regions, hospital settings, the patients studied, and the study time frames. Further analysis of species distribution across age groups showed that *C. parapsilosis* was particularly prevalent in patients under 18 years (Adam et al., 2021). Li et al. (2015) suggested that the increased isolation of non-albicans *Candida* might be associated with the widespread use of antifungal drugs. However, we did not find a significant association between antifungal use and species distribution. Future research should evaluate the impact of antifungal therapy on the incidence and species distribution of candidemia.

A majority (276) of *Candida* isolates collected in this study originated from the ICU, the Surgical Department, Internal Medicine, and the Emergency Department. The highest incidence of candidemia patients was observed in the ICU, potentially due to the severity of patients’ conditions, prolonged hospital stays, complex underlying diseases, frequent need for mechanical ventilation, extended use of broad-spectrum antibiotics, and intravenous catheterization. A previous study on 236 candidemia patients, with 172 cases (72.9%) being post-surgical, reported a significantly higher incidence of *C. parapsilosis* (Zhang et al., 2014). Similarly, the analysis of patients with post-surgical candidemia at our hospital revealed that *C. parapsilosis* was the dominant isolate (49.0% (50/102)). Patients with hematology, particularly those receiving immunosuppressive therapy or chemotherapy, are at heightened risk of candidemia. Moreover, *C. tropicalis* was the most commonly isolated species in the Hematology department (55.6% (15/27)), which is consistent with the findings in Brazil (Bergamasco et al., 2013). Our data further reveal that candidemia is more prevalent among the older population, especially those aged > 50 years, and is more common in males than in females. These findings align with the results of the CHIF-NET survey in China. Xiao et al. (2020) and other recent global studies (Pinto-Magalhaes et al., 2019; Aguilar et al., 2020). Notably, 89.1% of patients had received antibiotics prior to candidemia, and more than half of the patients suffered from hypertension and underwent surgery. Over 60% of patients had used intravascular catheters and were admitted to the ICU before developing a *Candida*-positive blood culture. Overall, the study shows that the well-established risk factors for candidemia include total parenteral nutrition, antibiotic exposure, intravascular catheters, mechanical ventilation, ICU admission, hematological malignancies, solid organ tumors, and surgery.

Regarding antifungal susceptibility, the present study evaluated six antifungal agents and found that *C. parapsilosis* and *C. albicans* were highly susceptible to all tested drugs. However, our results demonstrated that *C. tropicalis* and *N. glabratus* displayed reduced susceptibility to azoles. The susceptibility of *C. tropicalis* to fluconazole and itraconazole (78.0% and 81.4%, respectively) was consistent with the recently reported from CHIF-NET. However, its susceptibility to voriconazole (74.6%) was higher than that reported (49.2%) (Farmakiotis & Kontoyiannis, 2017). Bassetti et al. (2020) reported lower fluconazole resistance rates in *C. tropicalis* isolates from Latin America and North America, with rates of 1.1% and 5.5%, respectively (Bassetti et al., 2020). The resistance rates in other regions, such as China, were higher. The resistance rate of *C. tropicalis* to fluconazole was 22.2%, and it also showed 90.4% cross-resistance to voriconazole (Tan et al., 2016). Reports from Europe, such as those from Norway (Hesstvedt et al., 2015) and Switzerland, showed lower resistance rates to voriconazole (6.2% and 9%, respectively). These results suggest that the spread of azole-resistant *C. tropicalis* may be limited within the Asia-Pacific region. *N. glabratus* also exhibited reduced susceptibility to azoles, with 30.4% and 21.7% resistance and nWT to fluconazole and voriconazole, respectively. These findings are consistent with a multicenter study from China (Xiao et al., 2018). The increasing azole resistance among *Candida* species warrants concern, largely due to the overuse of azole drugs (Brescini et al., 2020). In response to the rising prevalence of azole resistance, the American Infectious Diseases Society (IDSA) recommended echinocandins as first-line agents for the treatment of *Candida* in 2016 (Pappas et al., 2016). Despite the emergence of echinocandin-resistant strains, it remains highly effective against fungal infections. Amphotericin B, a potent antifungal agent, has long been restricted in clinical application primarily owing to its substantial toxicity, which often manifests as severe nephrotoxicity and infusion-related adverse reactions.

The early symptoms of candidemia lack specificity, making differentiation from bacterial infections difficult. Blood culture is generally used as the gold standard for diagnosis. However, the culture process is time-consuming and may be interfered with by various factors, thus delaying early diagnosis. Given the long treatment durations, high recurrence rates, and elevated mortality associated with *Candida* infections, timely and accurate non-culturing diagnostic methods are needed. As a key component of fungal cell walls, BDG can be detected in serum and offers valuable assistance in diagnosing various fungal infections. Combining BDG assays with blood culture enhances early diagnostic and therapeutic monitoring capabilities. In this study, BDG testing was performed on 276 patients with candidemia, with a susceptibility of 41.5%. However, Yazdanpanah et al. (2024) reported a BDG test susceptibility rate of more than 90%. The disparity may be attributed to differences in the diagnostic reagents utilized. Similarly, Rouzé, Estella & Timsit (2022) reported a 45% BDG sensitivity in ICU patients, which aligned closely with our findings (Puig-Asensio et al., 2014b). Future studies, including larger sample sizes and additional regions, are needed to assess the diagnostic value of the BDG in candidemia. Though BDG-guided preemptive antifungal therapies did not significantly improve patients’ outcomes, they did contribute to a substantial reduction in antifungal dosages (Yoshida, 2021). Dynamic BDG monitoring in 10 patients showed a progressive decrease in BDG levels, which correlated with favorable clinical outcomes. However, five patients who initially experienced reduced BDG levels later saw levels rise again and had a poor prognosis. These findings highlight the utility of the BDG test in tracking the therapeutic response to antifungal therapy. Another notable observation in this study is that the β-D-glucan (BDG) assay is not useful for predicting the recurrence of dermatophyte foot infection. Superficial fungal infections usually cause fungal foot, while candidemia is an invasive fungal infection. Various factors may influence the sensitivity and specificity of BDG, so a comprehensive diagnostic approach that combines blood culture with clinical systems is necessary.

## Conclusions

In conclusion, this study provided valuable insights into the species distribution, risk factors, and antifungal susceptibility of *Candida* species causing candidemia in our region. It also highlights the potential role of BDG testing in early diagnosis and developing effective empirical treatment strategies for blood candidemia.The incidence of candidemia has increased in recent years, and *C. parapsilosis* was the most prevalent species. Thus, effective strategies are needed to help reduce morbidity and mortality among patients with candidemia.

##  Supplemental Information

10.7717/peerj.20832/supp-1 Supplemental Information 1 Clinical breakpoints (CBPs) and epidemiological cutoff values (ECVs)

10.7717/peerj.20832/supp-2 Supplemental Information 2 The ITS sequencing results

10.7717/peerj.20832/supp-3 Supplemental Information 3 Raw data

10.7717/peerj.20832/supp-4 Supplemental Information 4 The raw data related to the MALDI-TOF identification

10.7717/peerj.20832/supp-5 Supplemental Information 5 English translation codebook for raw data related to the MALDI-TOF identification

## Additional Information and Declarations

### Data Availability

The following information was supplied regarding data availability: The raw data is available in the Supplemental Files.
