# Supplementary material for: Candida species distribution, antifungal susceptibility and trends causing candidemia: a 10-year observation in eastern China
Source: PeerJ. 2026 Mar 5;14:e20832. doi: 10.7717/peerj.20832 (PMC12967414; doi:10.7717/peerj.20832)
Supplement: Supplemental Information 1 [file peerj-14-20832-s001.docx]

**Supplement 1. Clinical Break Points and Epidemiological Cutoff Values Applied in the Present Study**

| **Species** | **Antifungal agents** | **S**  **(ug/mL)** | **SDD (ug/mL)** | **I**  **(ug/mL)** | **R**  **(ug/mL)** | **ECV**  **(ug/mL)** | **Reference document** |
| --- | --- | --- | --- | --- | --- | --- | --- |
| *C. albicans* | Fluconazole | 2 | 4 | — | 8 | — | M27M44SED3 |
| *C. albicans* | Voriconazole | 0.12 | — | 0.25-0.5 | 1 | — | M27M44SED3 |
| *C. albicans* | Posaconazole | — | — | — | — | 0.06 | M57SED4 |
| *C. albicans* | Caspofungin | 0.25 | — | 0.5 | 1 | — | M27M44SED3 |
| *C. albicans* | Micafungin | 0.25 | — | 0.5 | 1 | — | M27M44SED3 |
| *C. albicans* | Amphotericin B | — | — | — | — | 2 | M57SED4 |
| *C. parapsilosis* | Fluconazole | 2 | 4 | — | 8 | — | M27M44SED3 |
| *C. parapsilosis* | Voriconazole | 0.12 | — | 0.25-0.5 | 1 | — | M27M44SED3 |
| *C. parapsilosis* | Itraconazole | — | — | — | — | 0.5 | M57SED4 |
| *C. parapsilosis* | Caspofungin | 2 | — | 4 | 8 | — | M27M44SED3 |
| *C. parapsilosis* | Micafungin | 2 | — | 4 | 8 | — | M27M44SED3 |
| *C. parapsilosis* | Amphotericin B | — | — | — | — | 1 | M57SED4 |
| *C. tropicalis* | Fluconazole | 2 | 4 | — | 8 | — | M27M44SED3 |
| *C. tropicalis* | Voriconazole | 0.12 | — | 0.25-0.5 | 1 | — | M27M44SED3 |
| *C. tropicalis* | Itraconazole | — | — | — | — | 0.5 | M57SED4 |
| *C. tropicalis* | Caspofungin | 0.25 | — | 0.5 | 1 | — | M27M44SED3 |
| *C. tropicalis* | Micafungin | 0.25 | — | 0.5 | 1 | — | M27M44SED3 |
| *C. tropicalis* | Amphotericin B | — | — | — | — | 2 | M57SED4 |
| *N. glabratus* | Fluconazole | — | 32 | — | 64 | — | M27M44SED3 |
| *N. glabratus* | Voriconazole | — | — | — | — | 0.25 | M57SED4 |
| *N. glabratus* | Itraconazole | — | — | — | — | 4 | M57SED4 |
| *N. glabratus* | Caspofungin | 0.12 | — | 0.25 | 0.5 | — | M27M44SED3 |
| *N. glabratus* | Micafungin | 0.06 | — | 0.12 | 0.25 | — | M27M44SED3 |
| *N. glabratus* | Amphotericin B | — | — | — | — | 2 | M57SED4 |
| *C. guilliermondii* | Fluconazole | — | — | — | — | 8 | M57SED4 |
| *C. guilliermondii* | Voriconazole | — | — | — | — | 0.5 | M57SED4 |
| *C. guilliermondii* | Itraconazole | — | — | — | — | 1 | M57SED4 |
| *C. guilliermondii* | Caspofungin | 2 | — | 4 | 8 | — | M27M44SED3 |
| *C. guilliermondii* | Micafungin | 2 | — | 4 | 8 | — | M27M44SED3 |
| *C. guilliermondii* | Amphotericin B | — | — | — | — | 2 | M57SED4 |
| *C. krusei* | Fluconazole | IR | IR | IR | IR | IR | M57SED4 |
| *C. krusei* | Voriconazole | 0.5 | — | 1 | 2 | — | M27M44SED3 |
| *C. krusei* | Itraconazole | — | — | — | — | 1 | M57SED4 |
| *C. krusei* | Caspofungin | 0.25 | — | 0.5 | 1 | — | M27M44SED3 |
| *C. krusei* | Micafungin | 0.25 | — | 0.5 | 1 | — | M27M44SED3 |
| *C. krusei* | Amphotericin B | — | — | — | — | 2 | M57SED4 |

Note: “—”: not available; IR: intrinsic resistance.
