# Supplementary material for: Candida species distribution, antifungal susceptibility and trends causing candidemia: a 10-year observation in eastern China
Source: PeerJ. 2026 Mar 5;14:e20832. doi: 10.7717/peerj.20832 (PMC12967414; doi:10.7717/peerj.20832)
Supplement: Supplemental Information 2 [file peerj-14-20832-s002.docx]

**There are some information about the methodology to extract DNA, PCR amplification, sequencing method and results.**

Genomic DNA was extracted using the Ezup Column Fungi Genomic DNA Purification Kit (Code: B518257, Sangon Biotech, Shanghai, China).

Primer sequence

| Primer | Primer sequence |
| --- | --- |
| ITS1 | 5’-TCCGTAGGTGAACCTGCGG-3’ |
| ITS4 | 5’-TCCTCCGCTTATTGATATGC-3’ |

PCR system

| **Reaction components** | **Volume (µl)** |
| --- | --- |
| 10 X PCR Buffer | 12.5 |
| dNTP (each 10 mM) |  |
| Taq Plus DNA Polymerase（5 U/μl） |  |
| 50mM MgSO_4_ |  |
| Primer F (10 µM) | 1 |
| Primer R (10 µM) | 1 |
| Template (DNA) | 1 |
| ddH_2_O | 9.5 |
| Total | 25 |

PCR Reaction Conditions

| **Temperature（°C）** | **Time** | **Cycle** |
| --- | --- | --- |
| 95 | 5 min |  |
| 94 | 30 s | 30cycle |
| 57 | 30 s |  |
| 72 | 90 s |  |
| 72 | 10min |  |

Results:

CGGAACATTTCATCCTTACACACCTGTGAATTTTTAAATGATACTTTGCTTTGGTCAGACTTTATAATTAGTCTGGCCAGAGGTATACAAACTCCAAATTTATTTTAAACATGAGTCTGAATTGAAAAAGAATAAATTATTCAAAACTTTCAACAACGGATCTCTTGGTTCTCGCATCGATGAAGAACGCAGCGAAATGCGATAAGTAATGTGAATTGCAGAATTTCGTGAATCATCGAATCTTTGAACGCACATTGCGCCCCTTGGTATTCCAGGGGGCATACGTGTATGAGCGTCATTTCACTCTTAAACCCTCGGGTTTAGTGTTGAACCTTTCCTTCGATCTTTTTTCGAAGGATGGCTCGAAATGAAATGGCAAGGCAATCCAGTCTAAAGCTTACACAGTGTCTTAGGTTTTACCAATTACGCTGGGCAAAGCTTGCTTGGATGAGCCGGGCGGTACAAACTTGATTTAAAATTTTCATCTC

| Description | Score | Query  Cover | E  value | Per.  Ident | Accession |
| --- | --- | --- | --- | --- | --- |
| Trichomonascus sp. strain SW143 internal transcribed spacer 1, partial sequence; 5.8S ribosomal RNA gene and internal transcribed spacer 2, complete sequence; and large subunit ribosomal RNA gene, partial sequence | 885 | 99% | 0.0 | 99.79% | KY065349 |
| Trichomonascus ciferrii genes for 18S rRNA, ITS1, 5.8S rRNA, ITS2, 28S rRNA, partial and complete sequence, strain: HKU-SZH 3591 | 885 | 99% | 0.0 | 99.79% | LC158127 |
| Trichomonascus ciferrii strain UOA/HCPF 16498 small subunit ribosomal RNA gene, partial sequence; internal transcribed spacer 1 and 5.8S ribosomal RNA gene, complete sequence; and internal transcribed spacer 2, partial sequence | 885 | 99% | 0.0 | 99.79% | ON182014 |
| Trichomonascus ciferrii strain KU60020.118 small subunit ribosomal RNA gene, partial sequence; internal transcribed spacer 1, 5.8S ribosomal RNA gene, and internal transcribed spacer 2, complete sequence; and large subunit ribosomal RNA gene, partial sequence | 885 | 99% | 0.0 | 99.79% | MW824439 |
| Trichomonascus ciferrii strain KU60020.120 small subunit ribosomal RNA gene, partial sequence; internal transcribed spacer 1, 5.8S ribosomal RNA gene, and internal transcribed spacer 2, complete sequence; and large subunit ribosomal RNA gene, partial sequence | 885 | 99% | 0.0 | 99.79% | MW819644 |
| Trichomonascus ciferrii strain CBS 215.79 18S ribosomal RNA gene, partial sequence; internal transcribed spacer 1, 5.8S ribosomal RNA gene, and internal transcribed spacer 2, complete sequence; and 28S ribosomal RNA gene, partial sequence | 883 | 98% | 0.0 | 100.0% | KX590833 |
| Trichomonascus ciferrii isolate M5164 small subunit ribosomal RNA gene, partial sequence; internal transcribed spacer 1 and 5.8S ribosomal RNA gene, complete sequence; and internal transcribed spacer 2, partial sequence | 883 | 98% | 0.0 | 100.0% | MW040038 |
| Trichomonascus sp. strain SW115 18S ribosomal RNA gene, partial sequence; internal transcribed spacer 1, 5.8S ribosomal RNA gene, and internal transcribed spacer 2, complete sequence; and 28S ribosomal RNA gene, partial sequence | 872 | 99% | 0.0 | 99.38% | KY260668 |
| [Candida] sp. (uncertain placement) strain SW114 small subunit ribosomal RNA gene, partial sequence; internal transcribed spacer 1, 5.8S ribosomal RNA gene, and internal transcribed spacer 2, complete sequence; and large subunit ribosomal RNA gene, partial sequence | 872 | 99% | 0.0 | 99.38% | KY065365 |
| Aspergillus sp. isolate 212 internal transcribed spacer 1, partial sequence; 5.8S ribosomal RNA gene and internal transcribed spacer 2, complete sequence; and large subunit ribosomal RNA gene, partial sequence | 872 | 99% | 0.0 | 99.38% | PP070047 |
