# Supplementary figures and images for: Candida species distribution, antifungal susceptibility and trends causing candidemia: a 10-year observation in eastern China (part 2 of 2)
Source: PeerJ. 2026 Mar 5;14:e20832. doi: 10.7717/peerj.20832 (PMC12967414; doi:10.7717/peerj.20832)

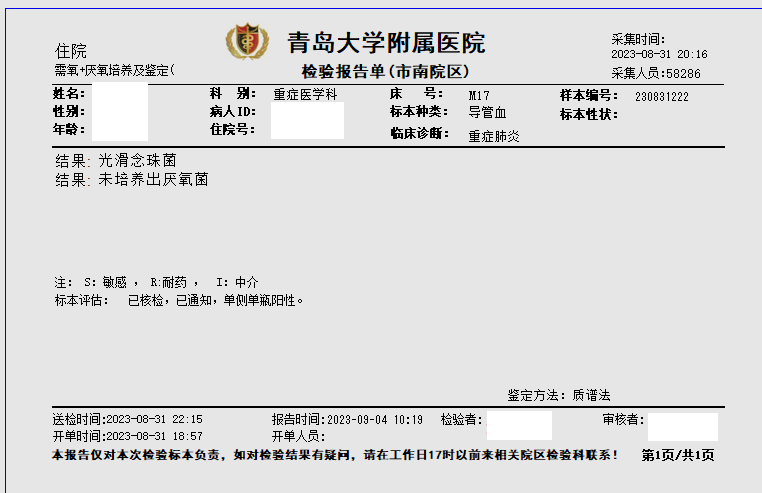

Supplement: Supplemental Information 4 [file peerj-14-20832-s004.zip › Supplement 4/19.png]

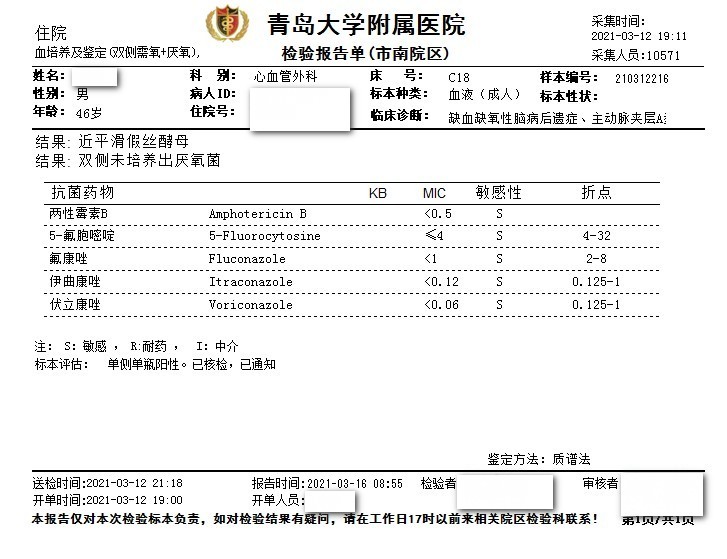

Supplement: Supplemental Information 4 [file peerj-14-20832-s004.zip › Supplement 4/190┴⌡.jpg]

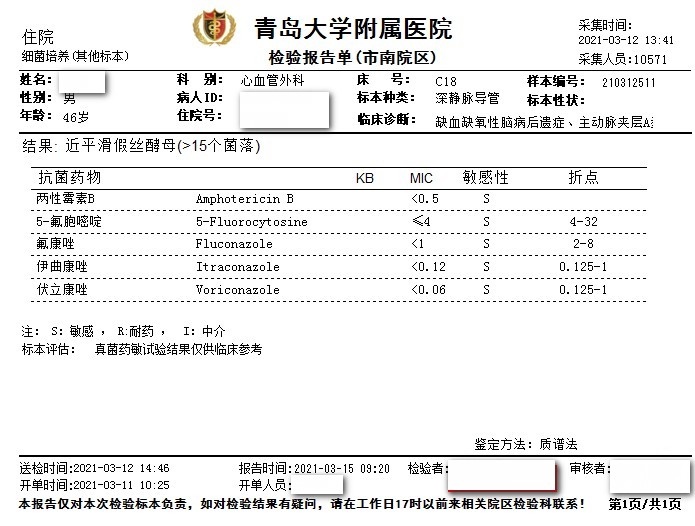

Supplement: Supplemental Information 4 [file peerj-14-20832-s004.zip › Supplement 4/191┴⌡.jpg]

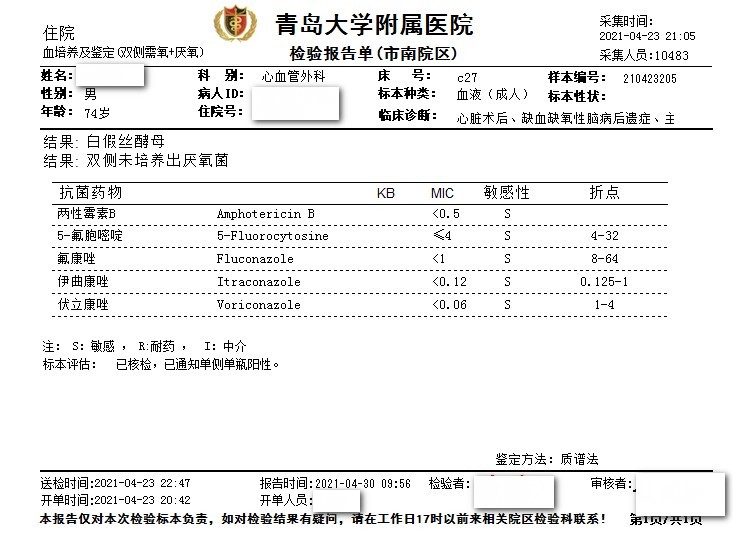

Supplement: Supplemental Information 4 [file peerj-14-20832-s004.zip › Supplement 4/192└ε.jpg]

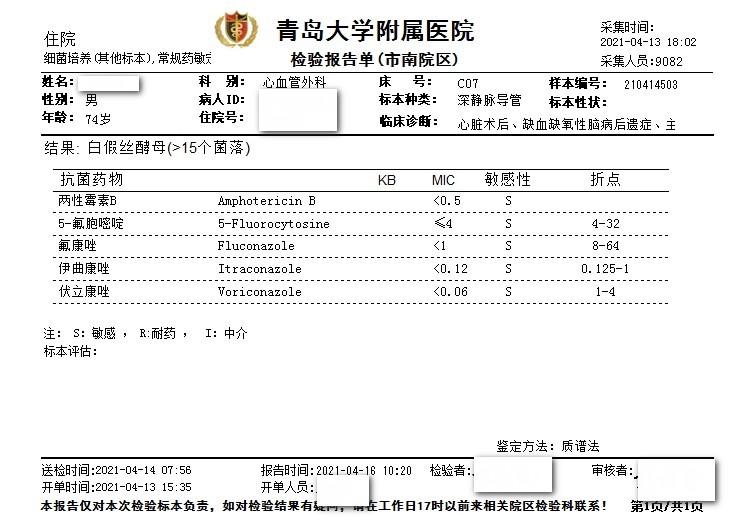

Supplement: Supplemental Information 4 [file peerj-14-20832-s004.zip › Supplement 4/193└ε.jpg]

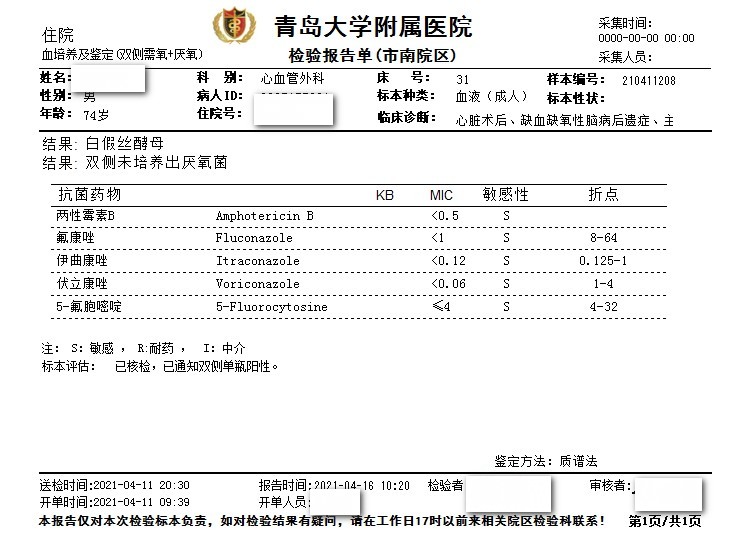

Supplement: Supplemental Information 4 [file peerj-14-20832-s004.zip › Supplement 4/194└ε.jpg]

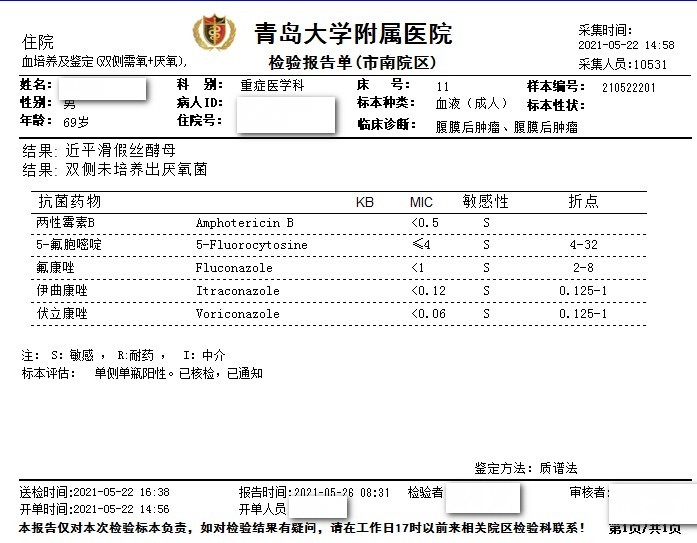

Supplement: Supplemental Information 4 [file peerj-14-20832-s004.zip › Supplement 4/195┼╦.jpg]

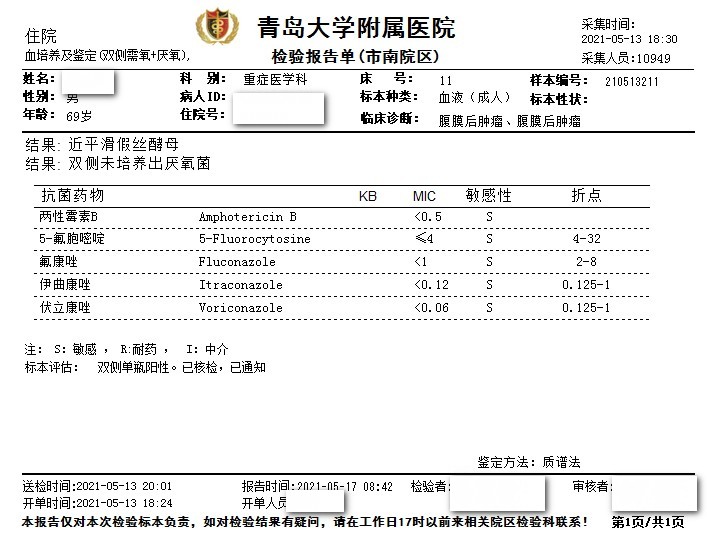

Supplement: Supplemental Information 4 [file peerj-14-20832-s004.zip › Supplement 4/196┼╦.jpg]

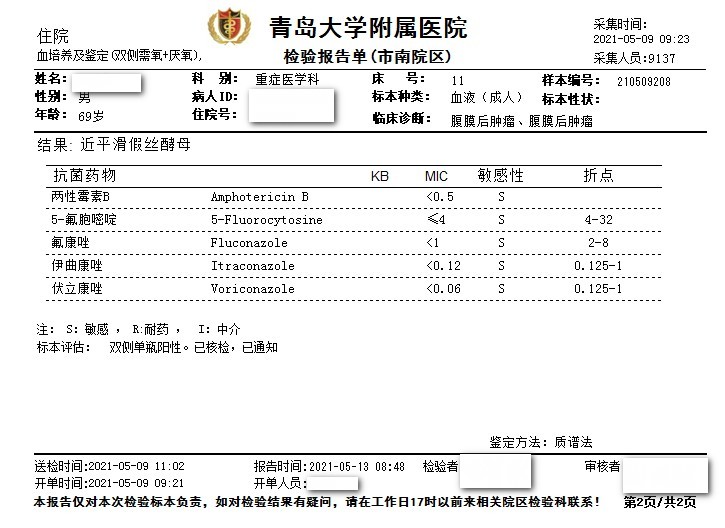

Supplement: Supplemental Information 4 [file peerj-14-20832-s004.zip › Supplement 4/197┼╦.jpg]

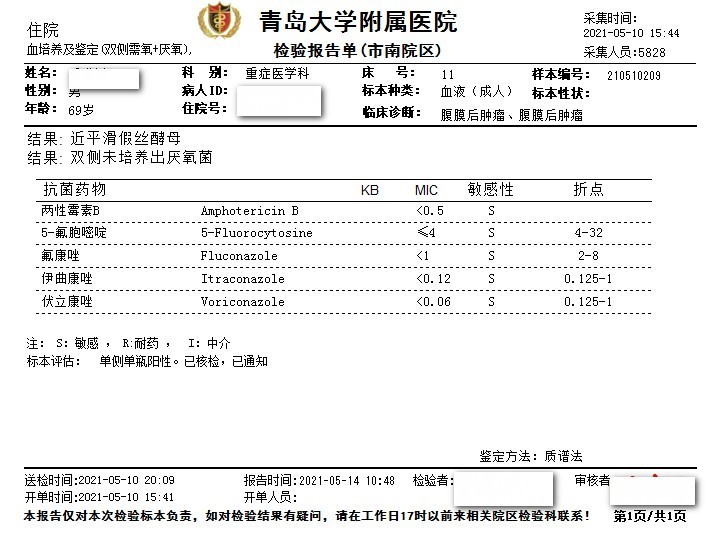

Supplement: Supplemental Information 4 [file peerj-14-20832-s004.zip › Supplement 4/198┼╦.jpg]

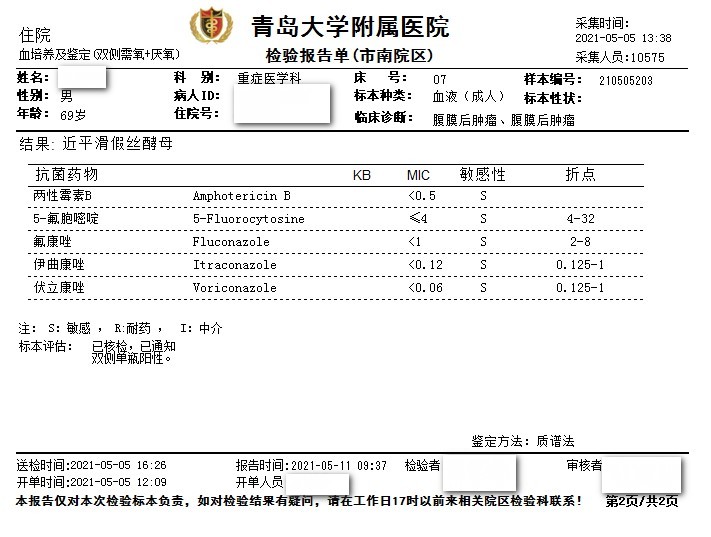

Supplement: Supplemental Information 4 [file peerj-14-20832-s004.zip › Supplement 4/199┼╦.jpg]

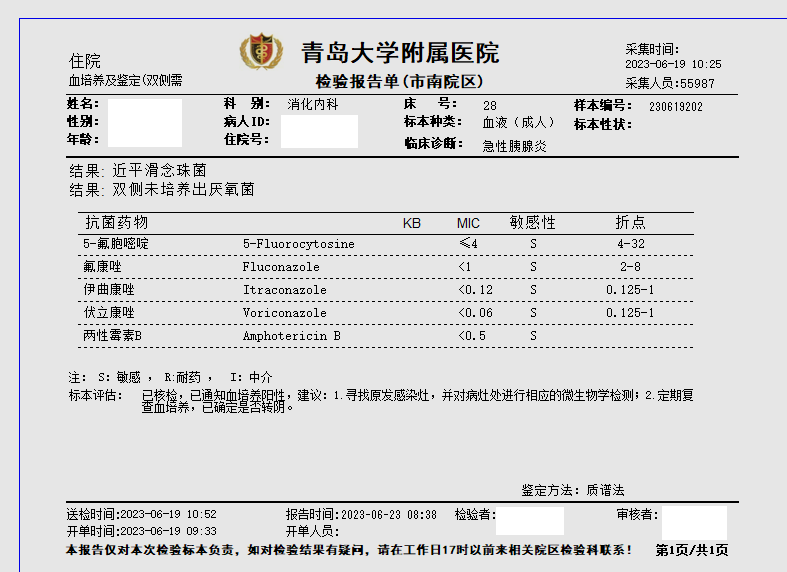

Supplement: Supplemental Information 4 [file peerj-14-20832-s004.zip › Supplement 4/2.png]

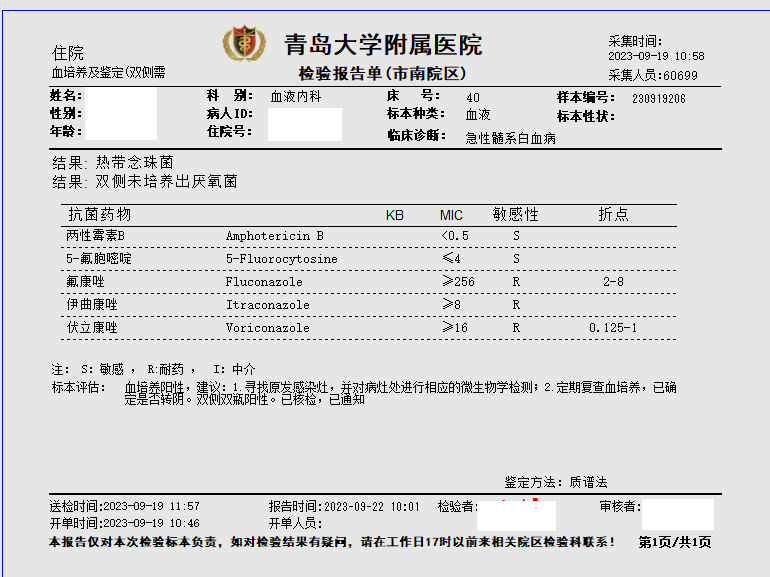

Supplement: Supplemental Information 4 [file peerj-14-20832-s004.zip › Supplement 4/20.png]

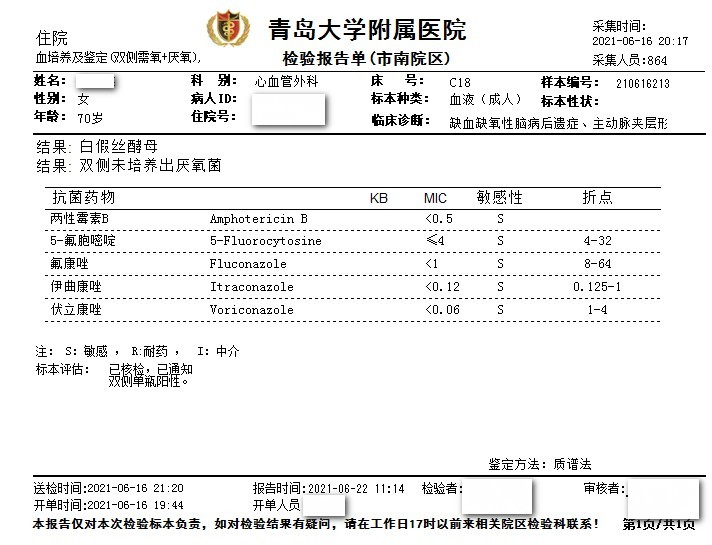

Supplement: Supplemental Information 4 [file peerj-14-20832-s004.zip › Supplement 4/200╩ó.jpg]

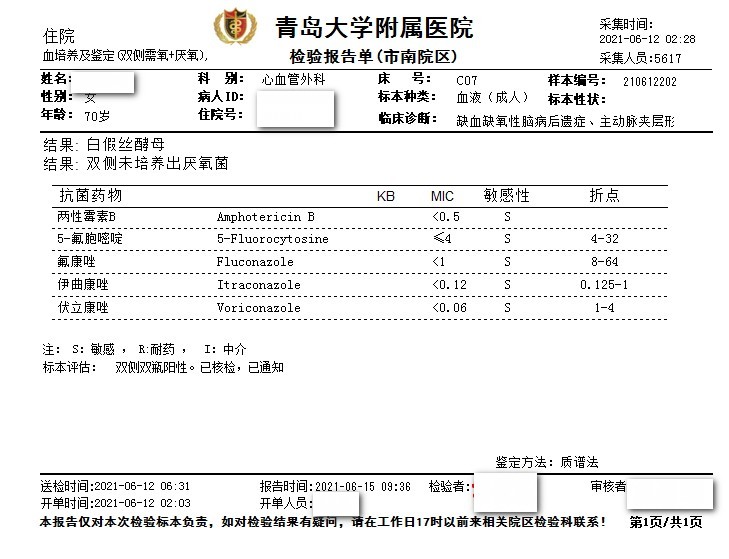

Supplement: Supplemental Information 4 [file peerj-14-20832-s004.zip › Supplement 4/201╩ó.jpg]

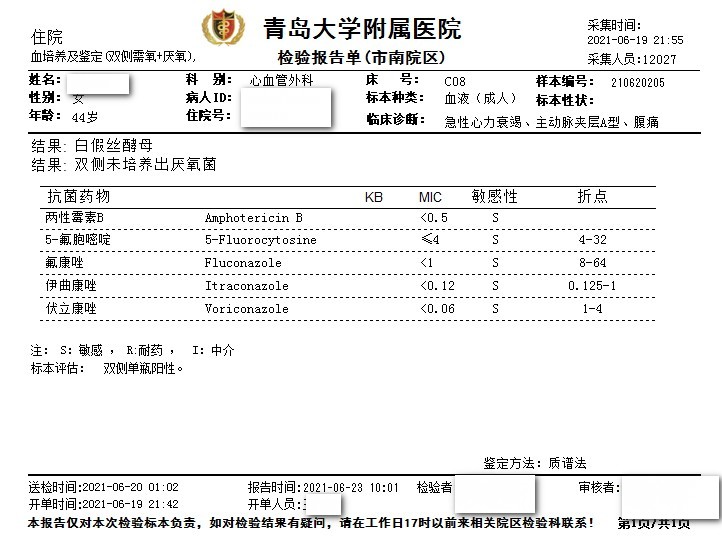

Supplement: Supplemental Information 4 [file peerj-14-20832-s004.zip › Supplement 4/202└ε.jpg]

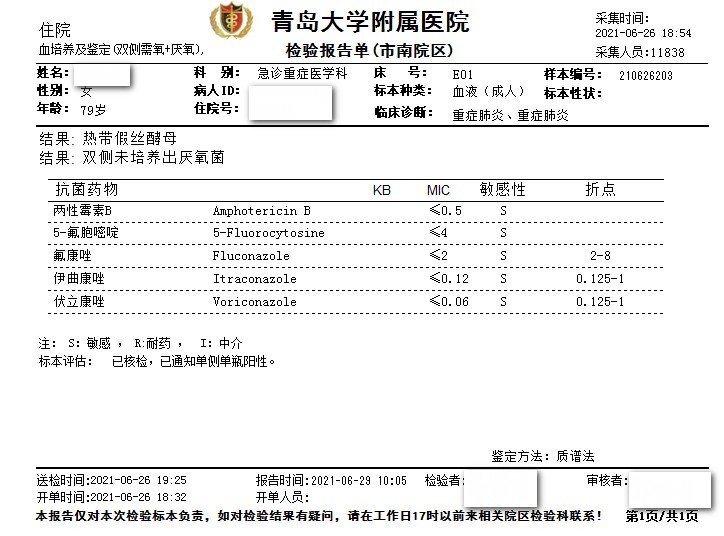

Supplement: Supplemental Information 4 [file peerj-14-20832-s004.zip › Supplement 4/203╠¿.jpg]

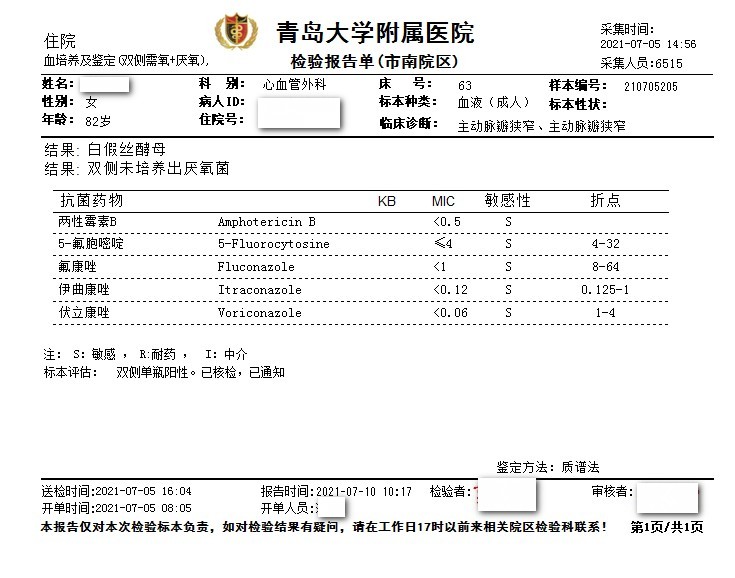

Supplement: Supplemental Information 4 [file peerj-14-20832-s004.zip › Supplement 4/204║╪.jpg]

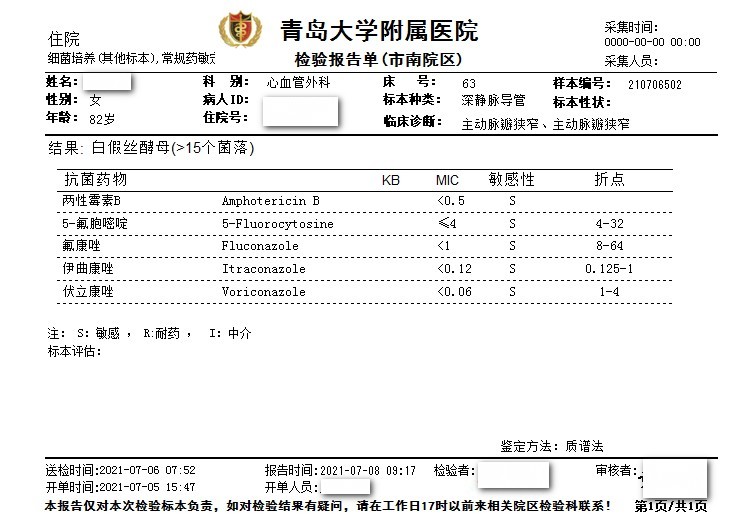

Supplement: Supplemental Information 4 [file peerj-14-20832-s004.zip › Supplement 4/205║╪.jpg]

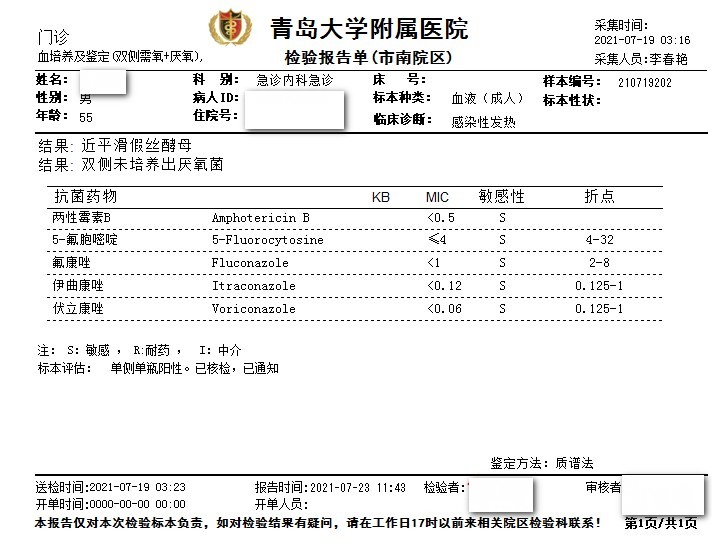

Supplement: Supplemental Information 4 [file peerj-14-20832-s004.zip › Supplement 4/206╚╬.jpg]

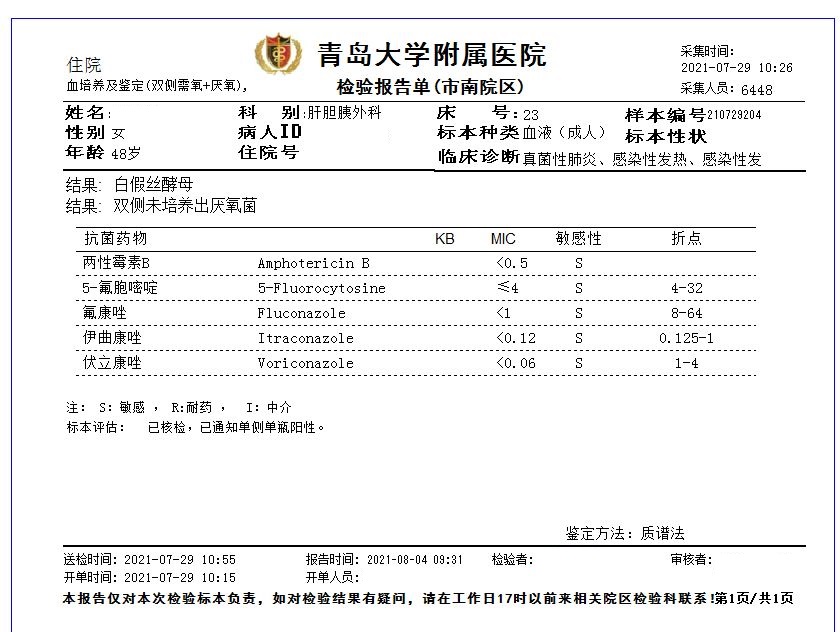

Supplement: Supplemental Information 4 [file peerj-14-20832-s004.zip › Supplement 4/207│┬.jpg]

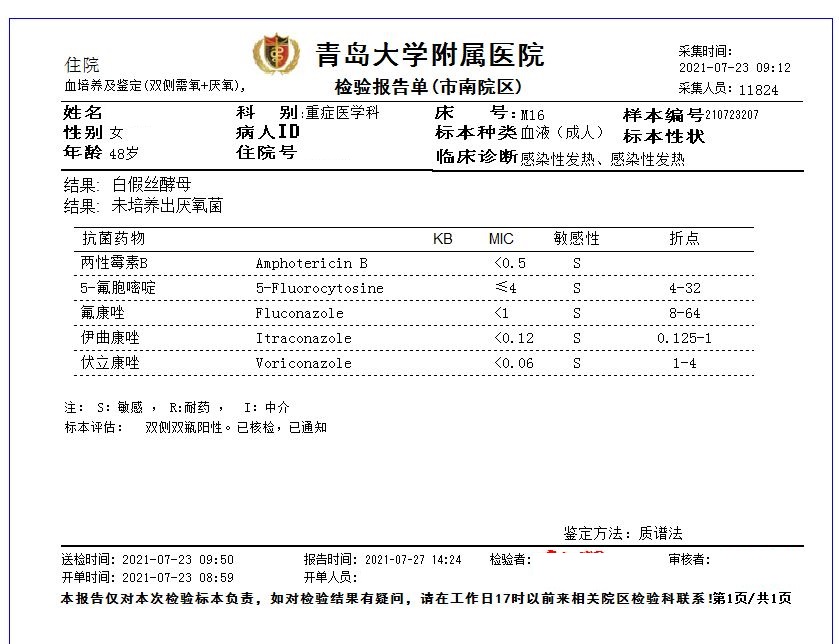

Supplement: Supplemental Information 4 [file peerj-14-20832-s004.zip › Supplement 4/208│┬.jpg]

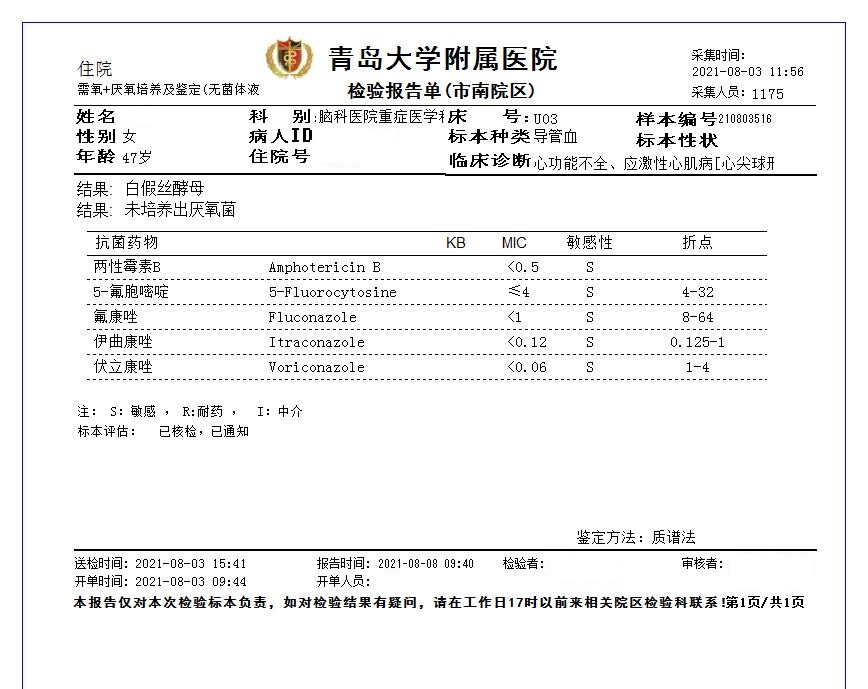

Supplement: Supplemental Information 4 [file peerj-14-20832-s004.zip › Supplement 4/209.jpg]

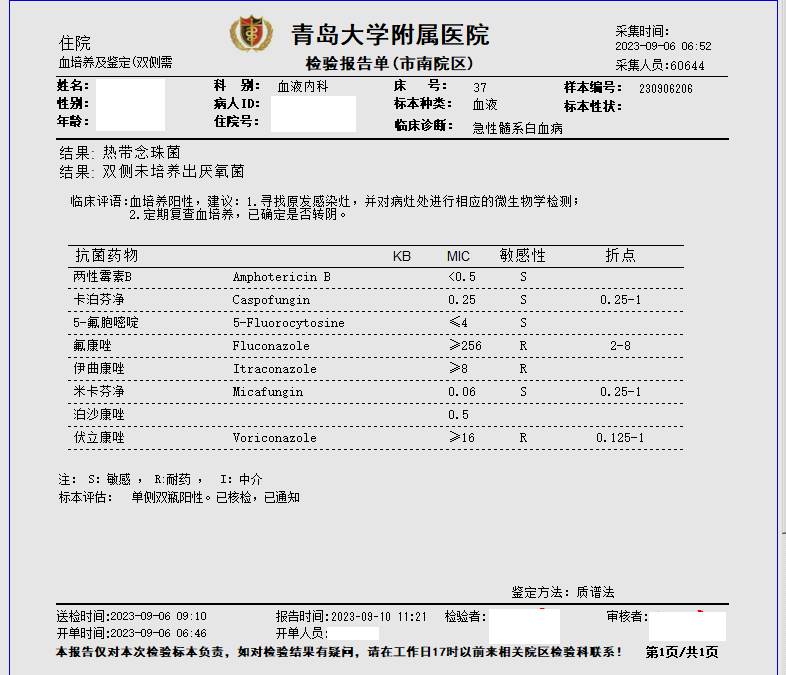

Supplement: Supplemental Information 4 [file peerj-14-20832-s004.zip › Supplement 4/21.png]

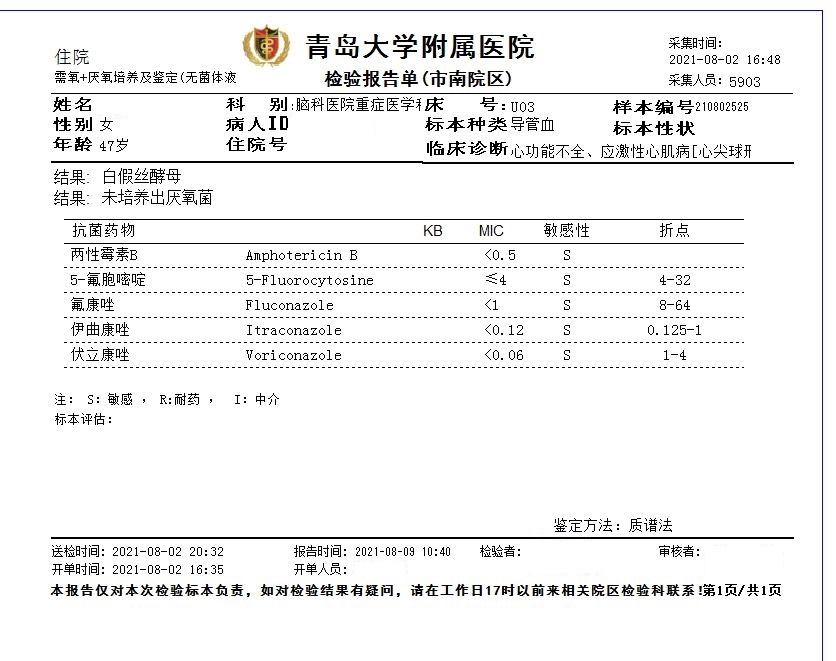

Supplement: Supplemental Information 4 [file peerj-14-20832-s004.zip › Supplement 4/210.jpg]

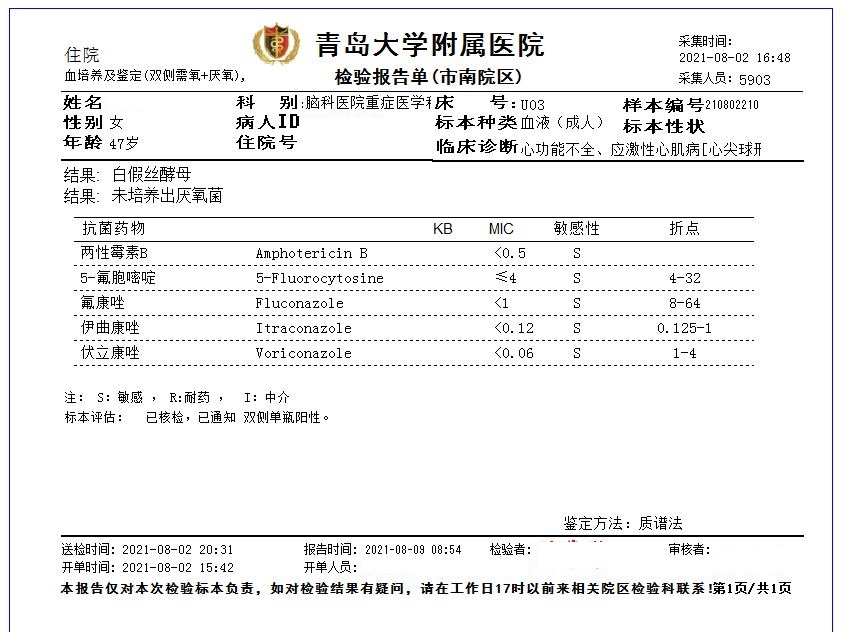

Supplement: Supplemental Information 4 [file peerj-14-20832-s004.zip › Supplement 4/211.jpg]

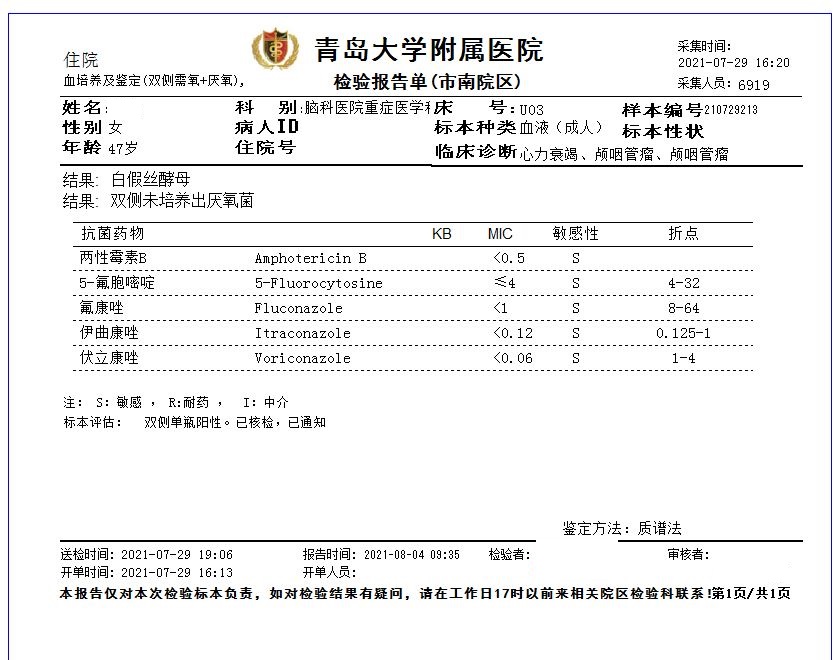

Supplement: Supplemental Information 4 [file peerj-14-20832-s004.zip › Supplement 4/212.jpg]

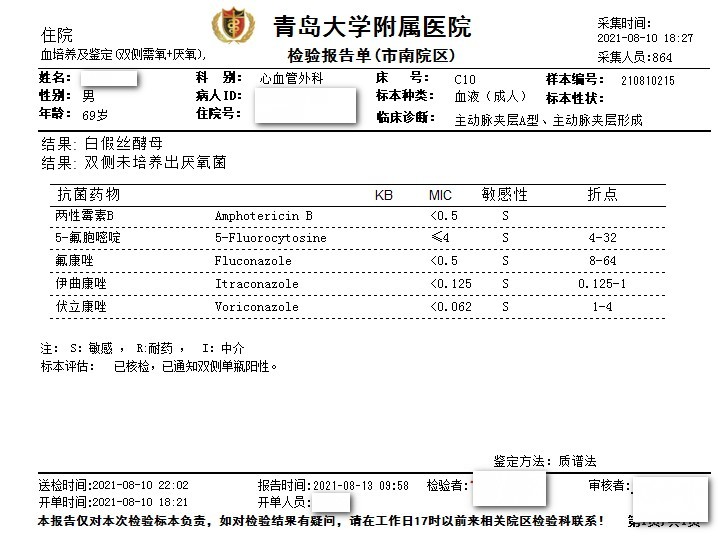

Supplement: Supplemental Information 4 [file peerj-14-20832-s004.zip › Supplement 4/213╟·.jpg]

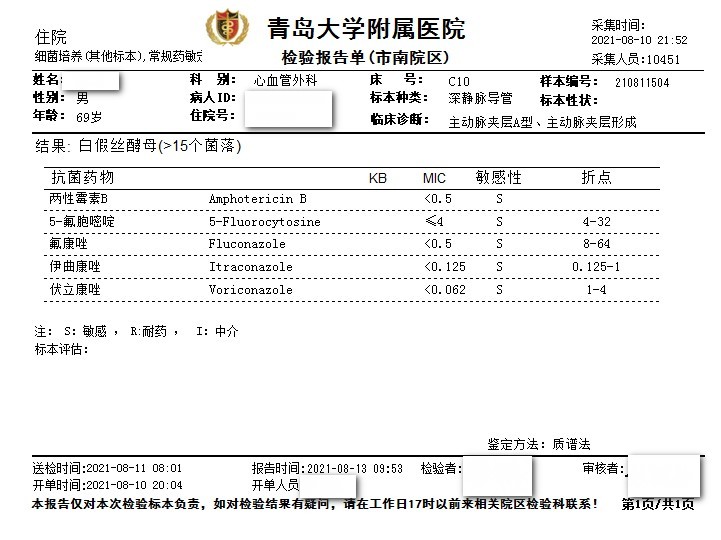

Supplement: Supplemental Information 4 [file peerj-14-20832-s004.zip › Supplement 4/214╟·.jpg]

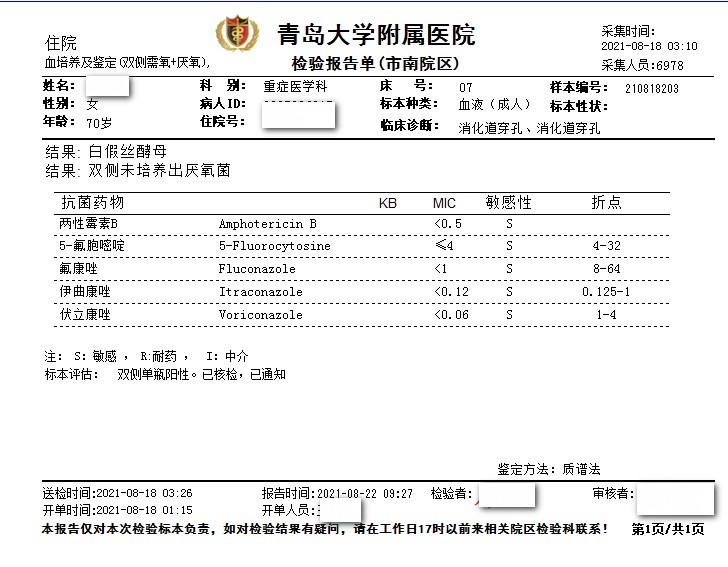

Supplement: Supplemental Information 4 [file peerj-14-20832-s004.zip › Supplement 4/215┤≈.jpg]

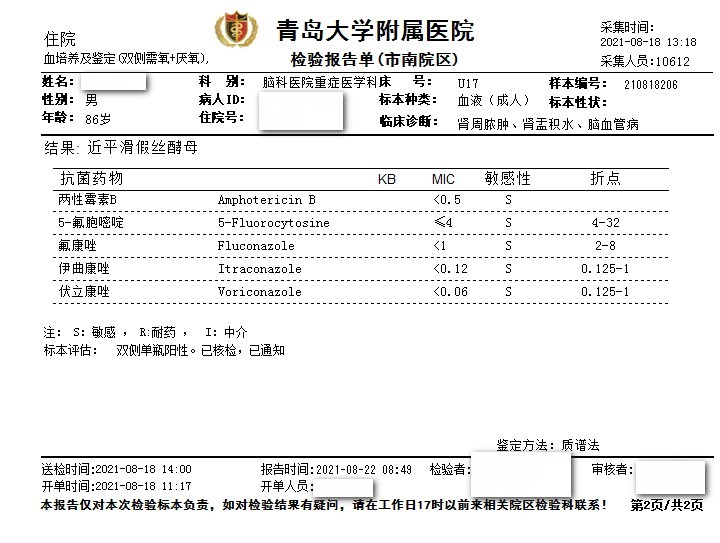

Supplement: Supplemental Information 4 [file peerj-14-20832-s004.zip › Supplement 4/216║╪.jpg]

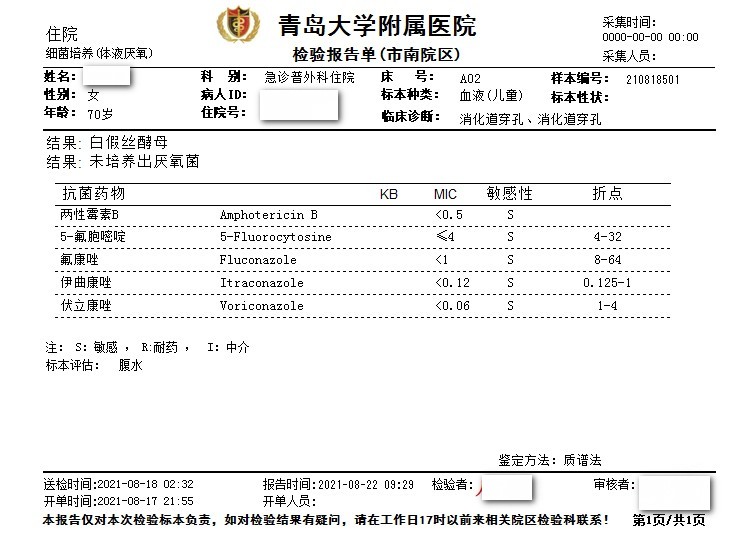

Supplement: Supplemental Information 4 [file peerj-14-20832-s004.zip › Supplement 4/217┤≈.jpg]

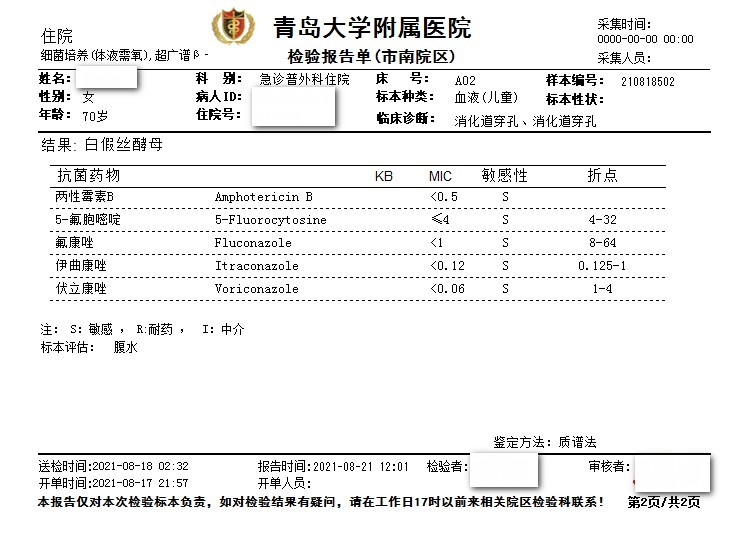

Supplement: Supplemental Information 4 [file peerj-14-20832-s004.zip › Supplement 4/218┤≈.jpg]

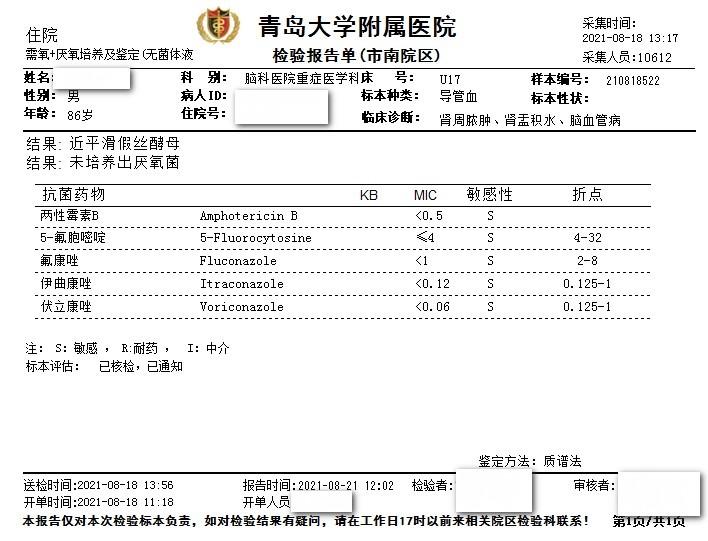

Supplement: Supplemental Information 4 [file peerj-14-20832-s004.zip › Supplement 4/219║╪.jpg]

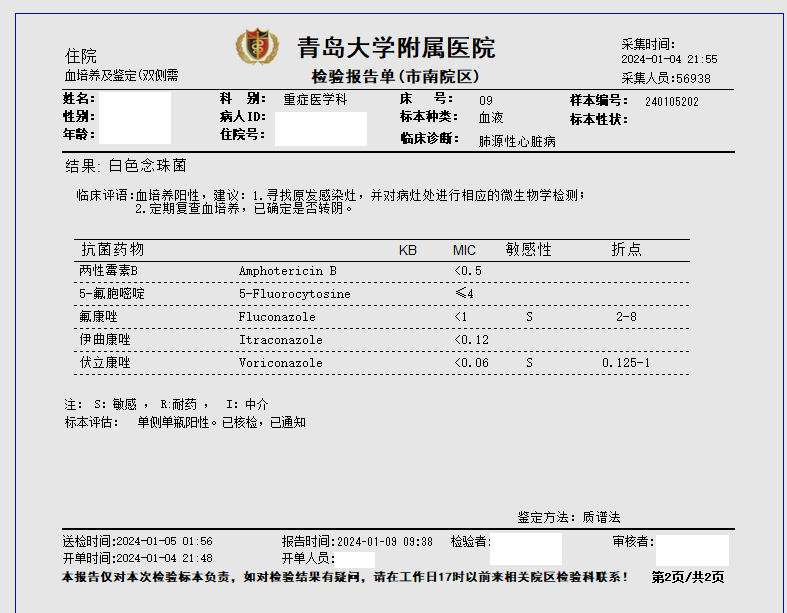

Supplement: Supplemental Information 4 [file peerj-14-20832-s004.zip › Supplement 4/22.png]

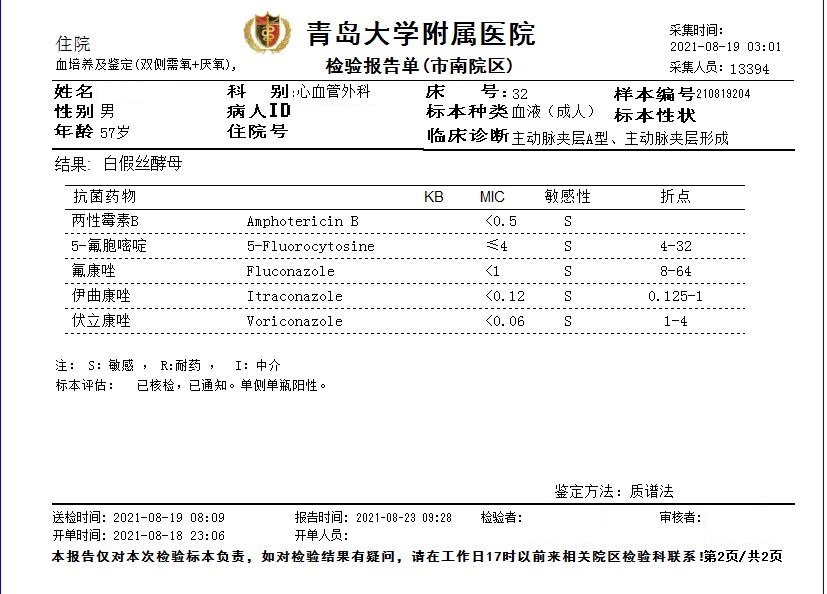

Supplement: Supplemental Information 4 [file peerj-14-20832-s004.zip › Supplement 4/220░╫.jpg]

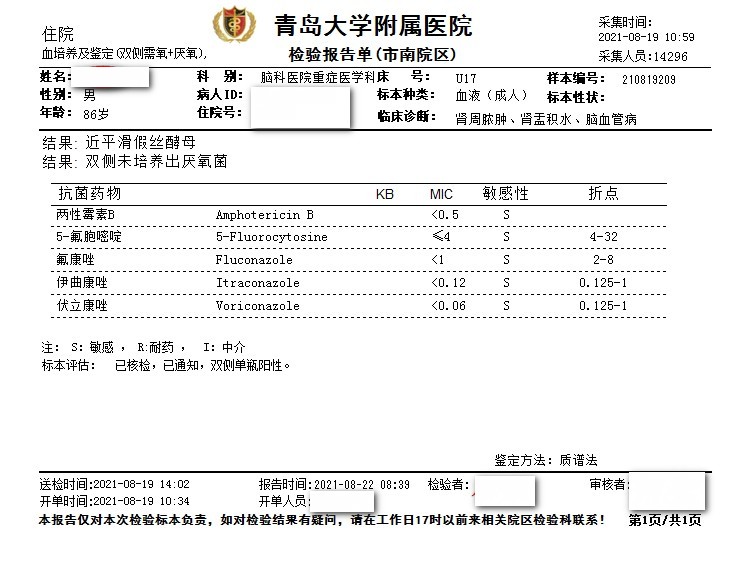

Supplement: Supplemental Information 4 [file peerj-14-20832-s004.zip › Supplement 4/221║╪.jpg]

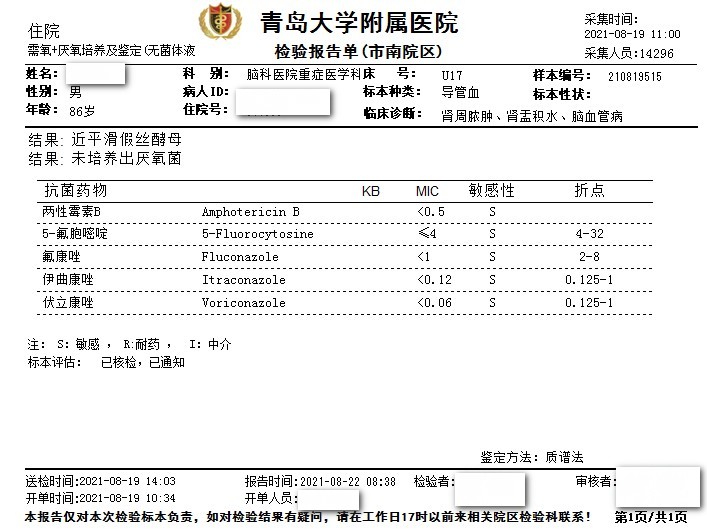

Supplement: Supplemental Information 4 [file peerj-14-20832-s004.zip › Supplement 4/222║╪.jpg]

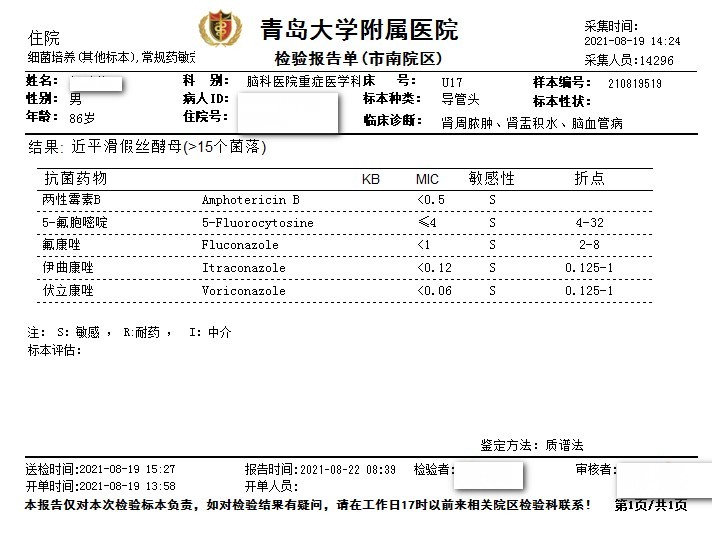

Supplement: Supplemental Information 4 [file peerj-14-20832-s004.zip › Supplement 4/223║╪jpg.jpg]

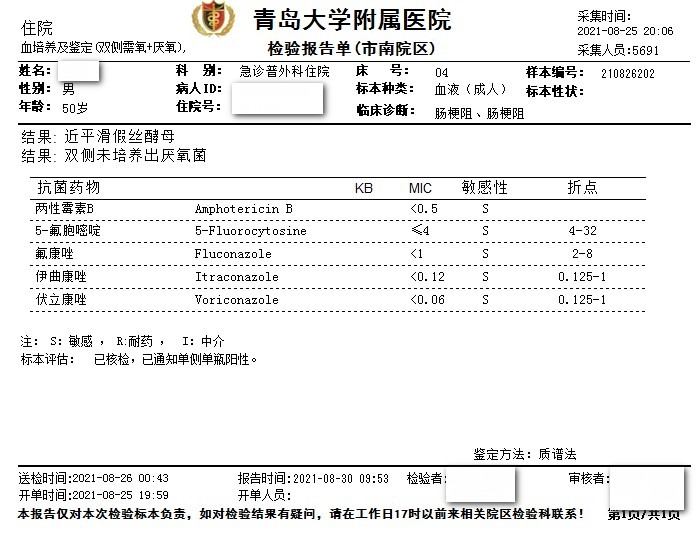

Supplement: Supplemental Information 4 [file peerj-14-20832-s004.zip › Supplement 4/224═⌡.jpg]

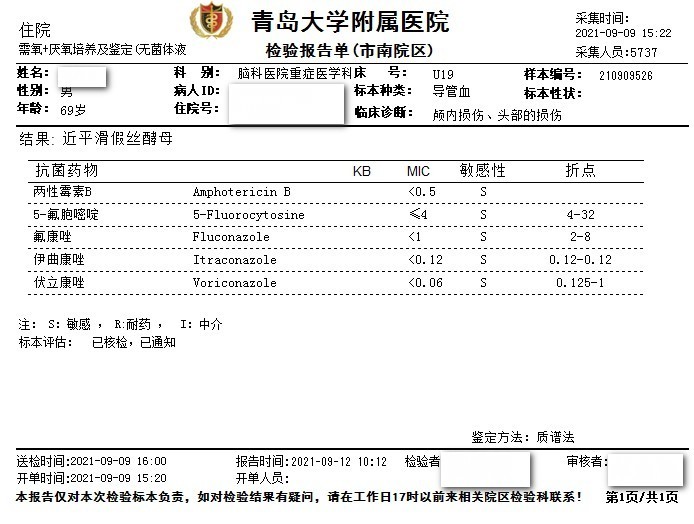

Supplement: Supplemental Information 4 [file peerj-14-20832-s004.zip › Supplement 4/225┴⌡.jpg]

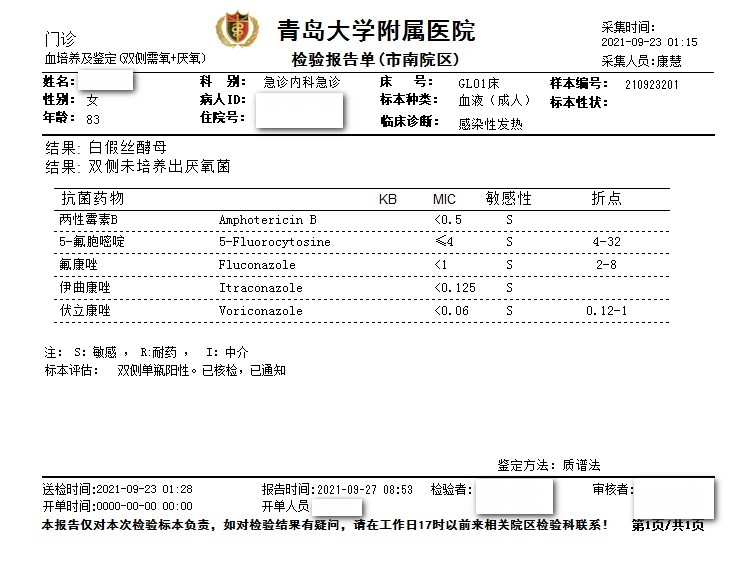

Supplement: Supplemental Information 4 [file peerj-14-20832-s004.zip › Supplement 4/226║╪.jpg]

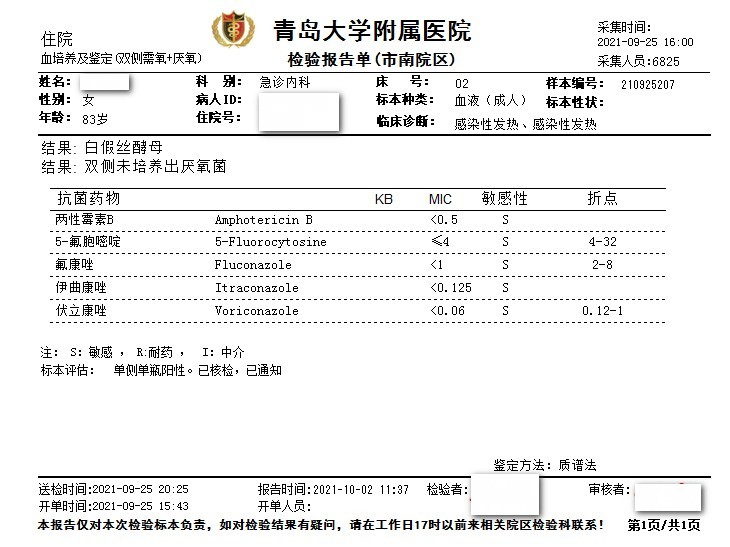

Supplement: Supplemental Information 4 [file peerj-14-20832-s004.zip › Supplement 4/227║╪.jpg]

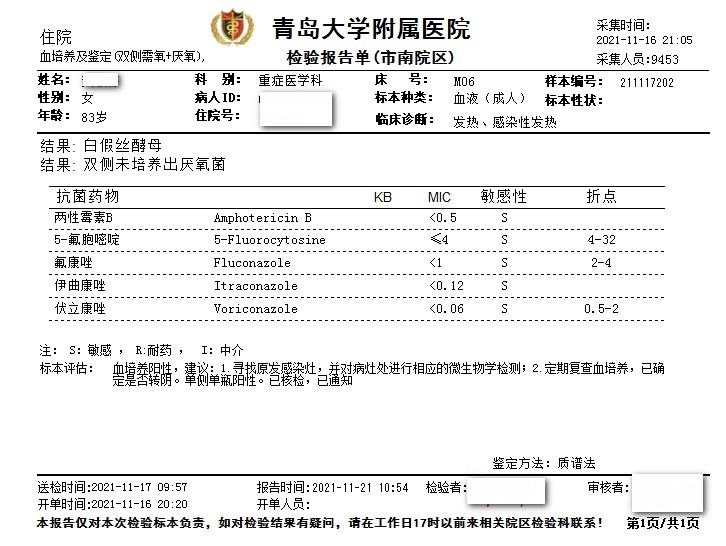

Supplement: Supplemental Information 4 [file peerj-14-20832-s004.zip › Supplement 4/228║╪.jpg]

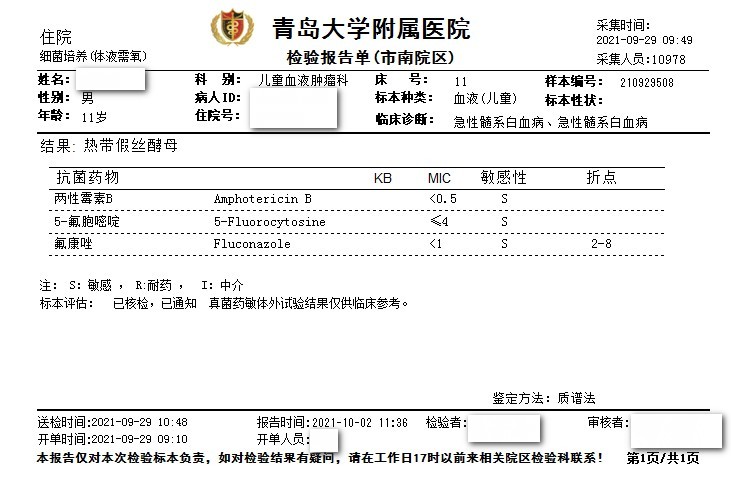

Supplement: Supplemental Information 4 [file peerj-14-20832-s004.zip › Supplement 4/229└╫.jpg]

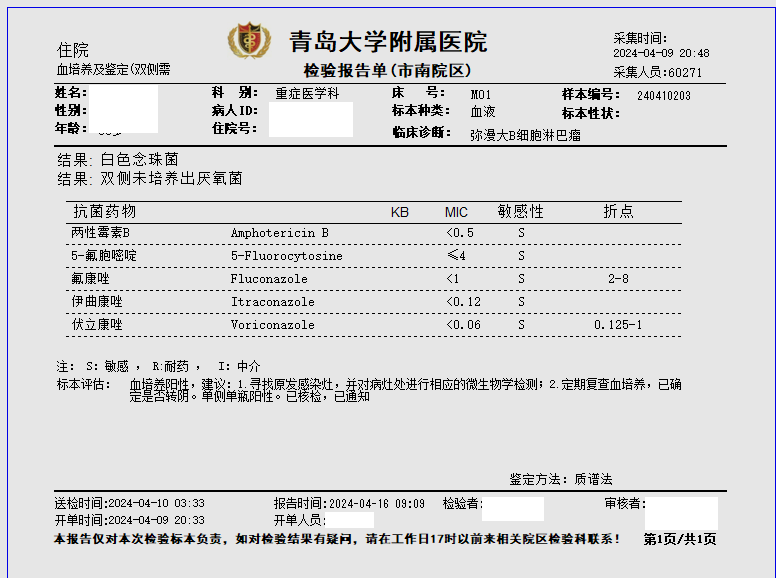

Supplement: Supplemental Information 4 [file peerj-14-20832-s004.zip › Supplement 4/23.png]

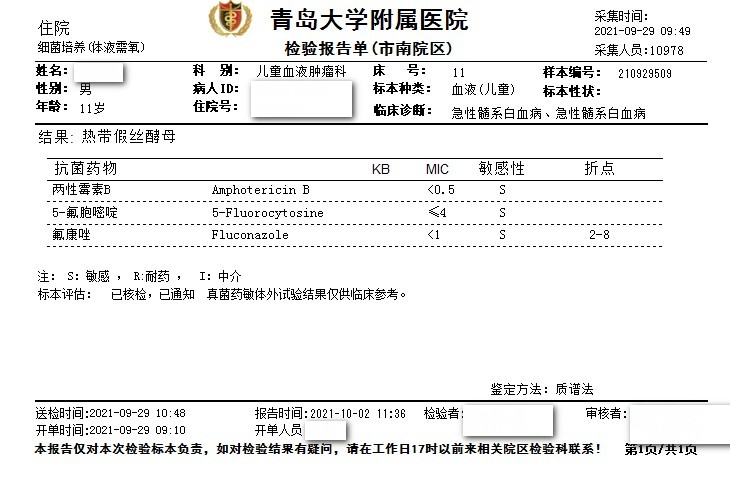

Supplement: Supplemental Information 4 [file peerj-14-20832-s004.zip › Supplement 4/230└╫.jpg]

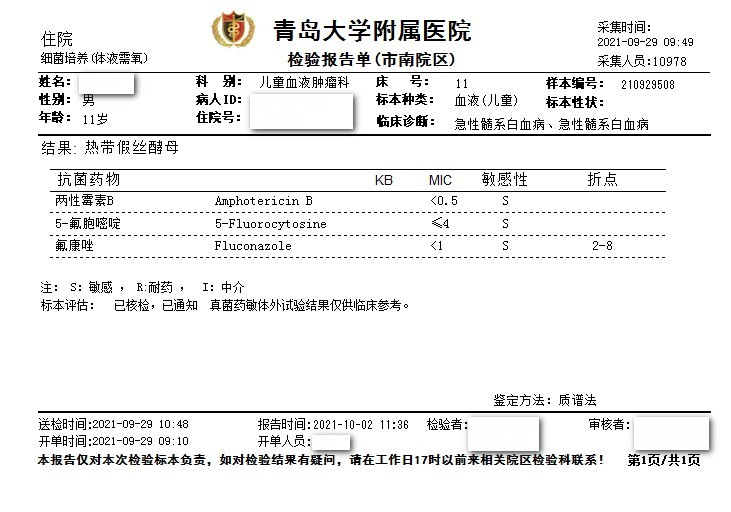

Supplement: Supplemental Information 4 [file peerj-14-20832-s004.zip › Supplement 4/231└╫.jpg]

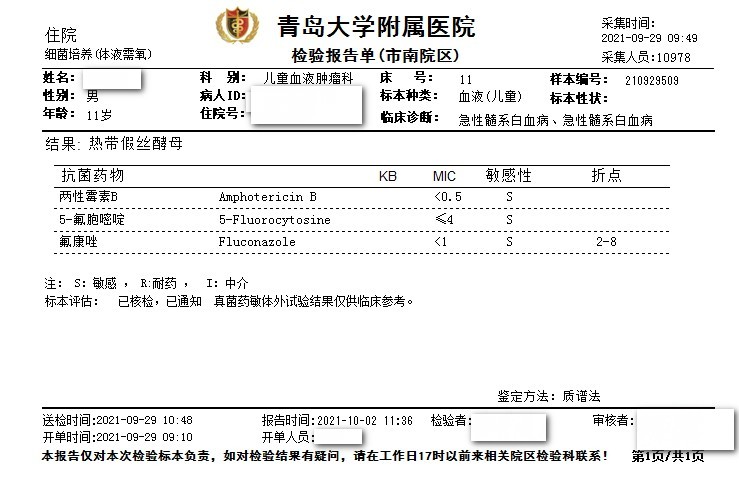

Supplement: Supplemental Information 4 [file peerj-14-20832-s004.zip › Supplement 4/232└╫.jpg]

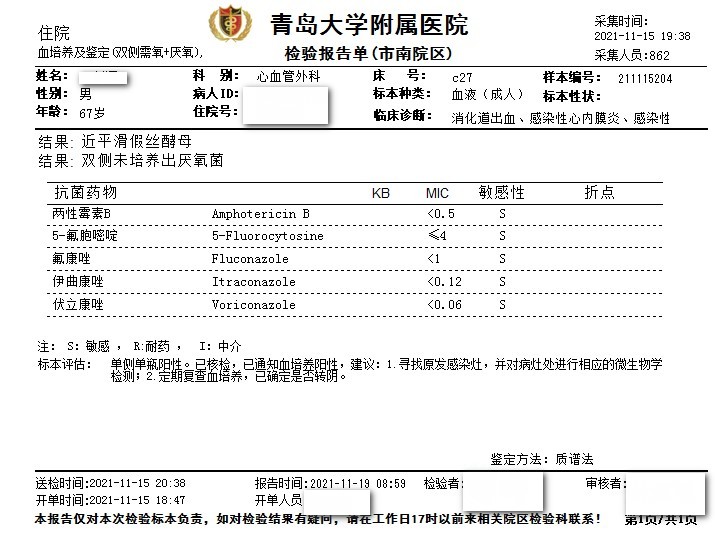

Supplement: Supplemental Information 4 [file peerj-14-20832-s004.zip › Supplement 4/233═⌡.jpg]

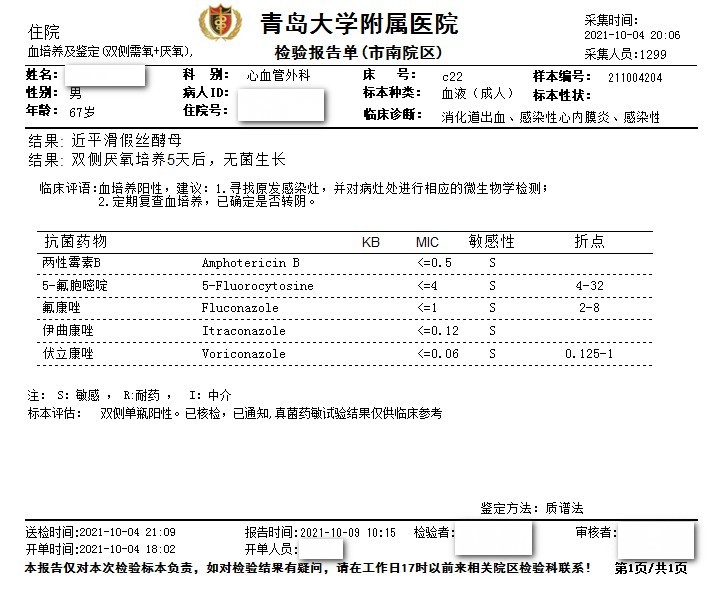

Supplement: Supplemental Information 4 [file peerj-14-20832-s004.zip › Supplement 4/234═⌡.jpg]

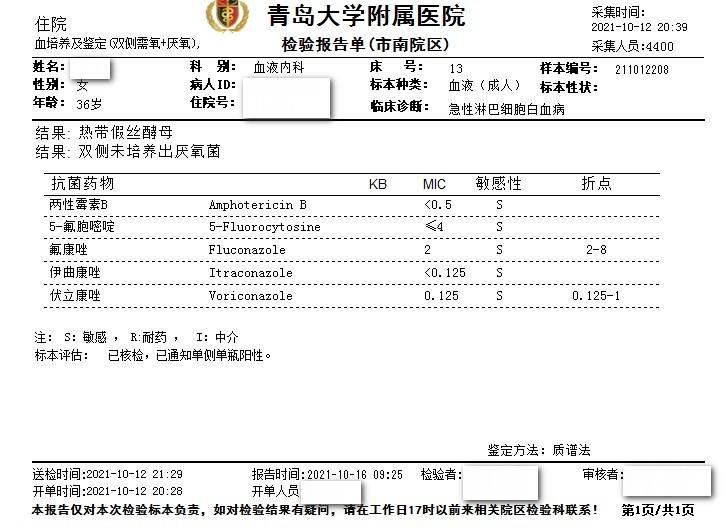

Supplement: Supplemental Information 4 [file peerj-14-20832-s004.zip › Supplement 4/235╓▄.jpg]

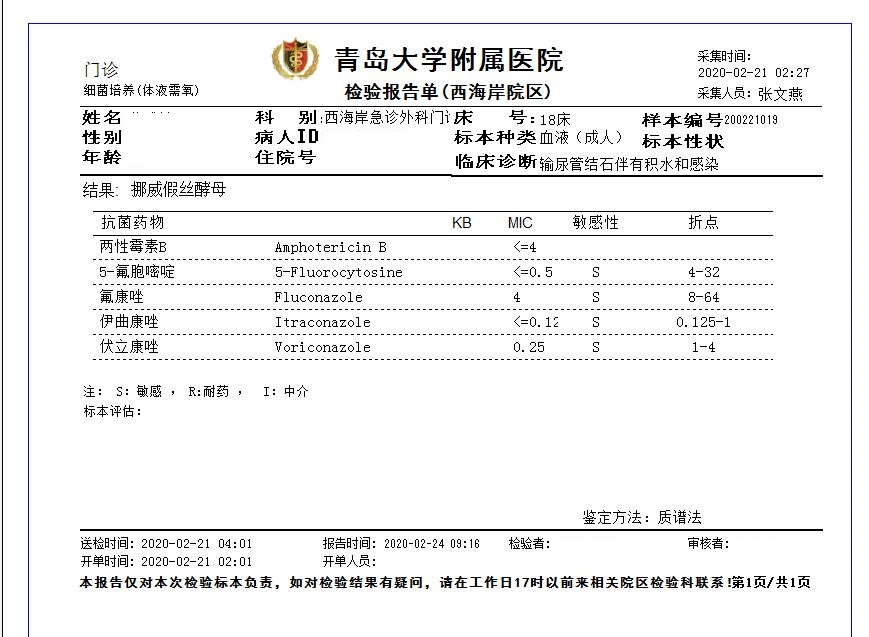

Supplement: Supplemental Information 4 [file peerj-14-20832-s004.zip › Supplement 4/236.JPG]

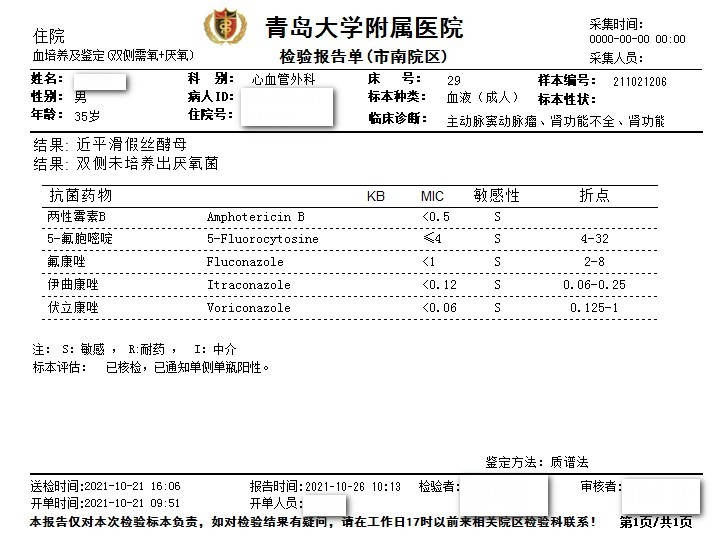

Supplement: Supplemental Information 4 [file peerj-14-20832-s004.zip › Supplement 4/237└ε.jpg]

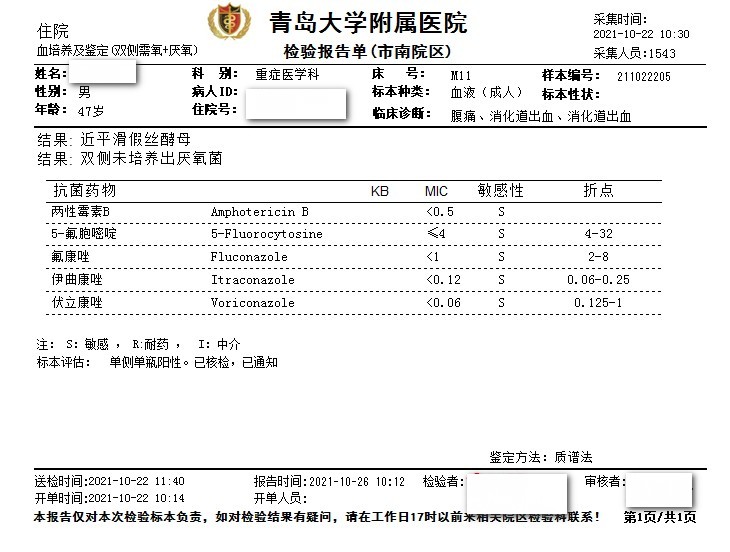

Supplement: Supplemental Information 4 [file peerj-14-20832-s004.zip › Supplement 4/238╨∞.jpg]

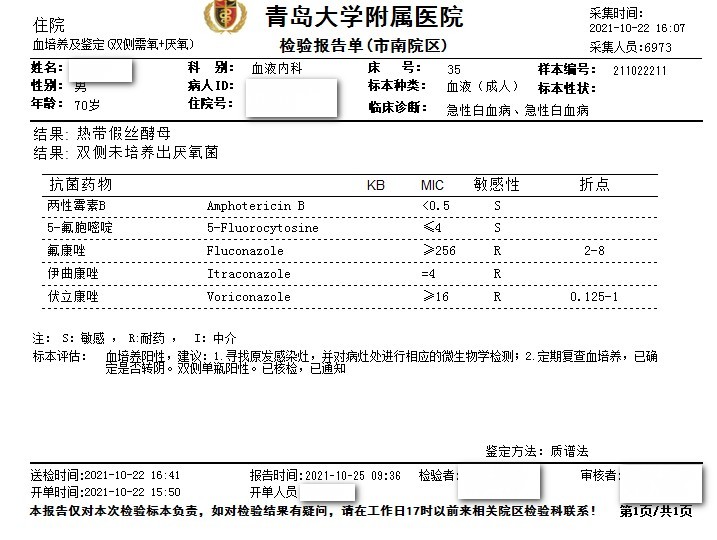

Supplement: Supplemental Information 4 [file peerj-14-20832-s004.zip › Supplement 4/239╠╒.jpg]

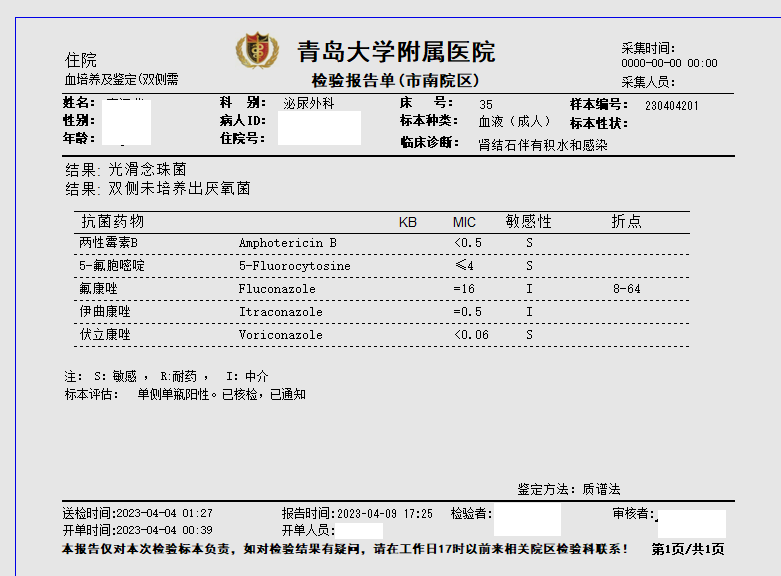

Supplement: Supplemental Information 4 [file peerj-14-20832-s004.zip › Supplement 4/24.png]

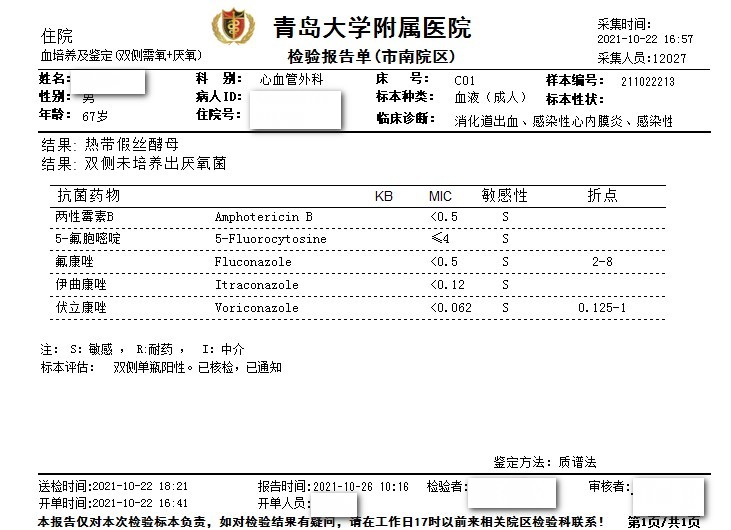

Supplement: Supplemental Information 4 [file peerj-14-20832-s004.zip › Supplement 4/240═⌡.jpg]

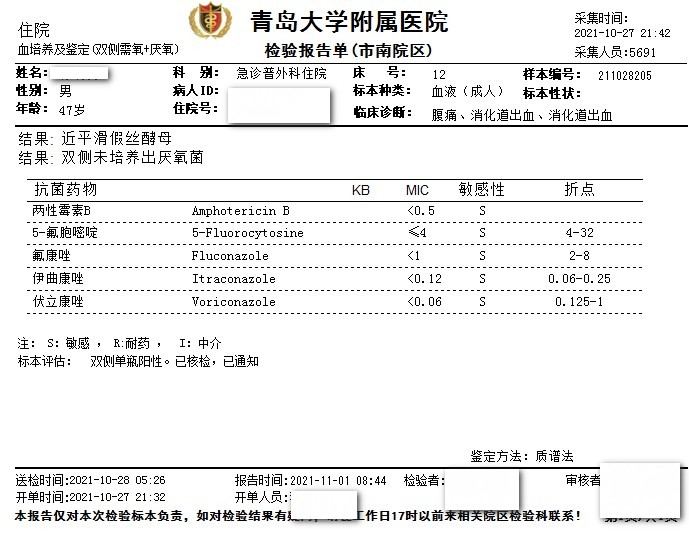

Supplement: Supplemental Information 4 [file peerj-14-20832-s004.zip › Supplement 4/241╨∞.jpg]

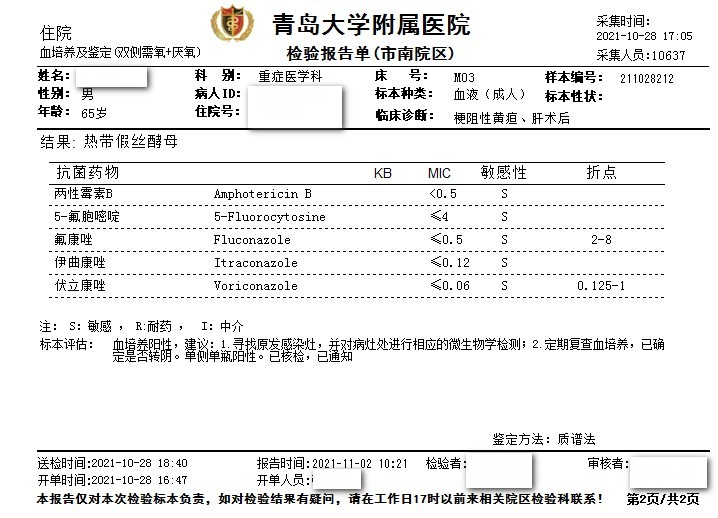

Supplement: Supplemental Information 4 [file peerj-14-20832-s004.zip › Supplement 4/242╙┌.jpg]

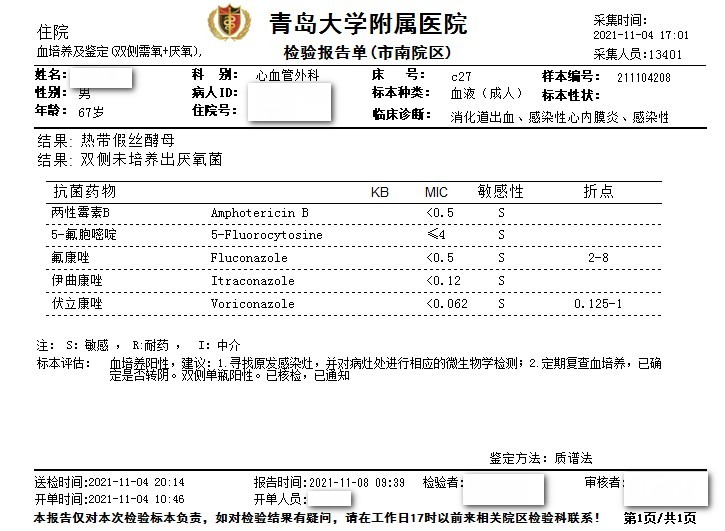

Supplement: Supplemental Information 4 [file peerj-14-20832-s004.zip › Supplement 4/243═⌡.jpg]

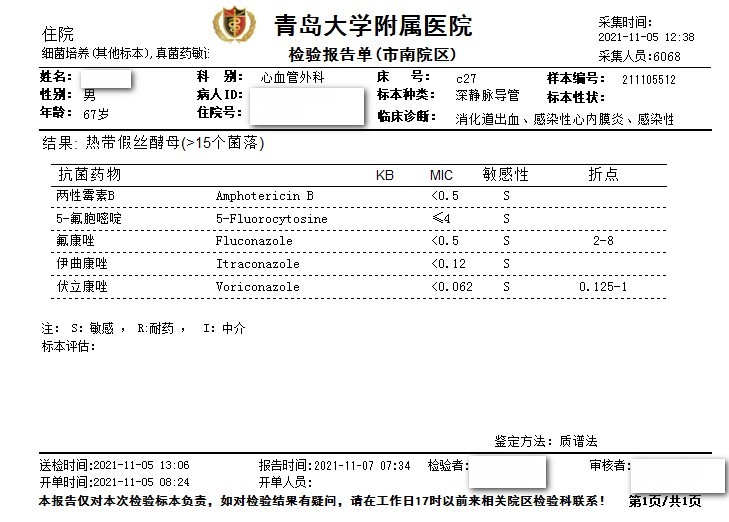

Supplement: Supplemental Information 4 [file peerj-14-20832-s004.zip › Supplement 4/244═⌡.jpg]

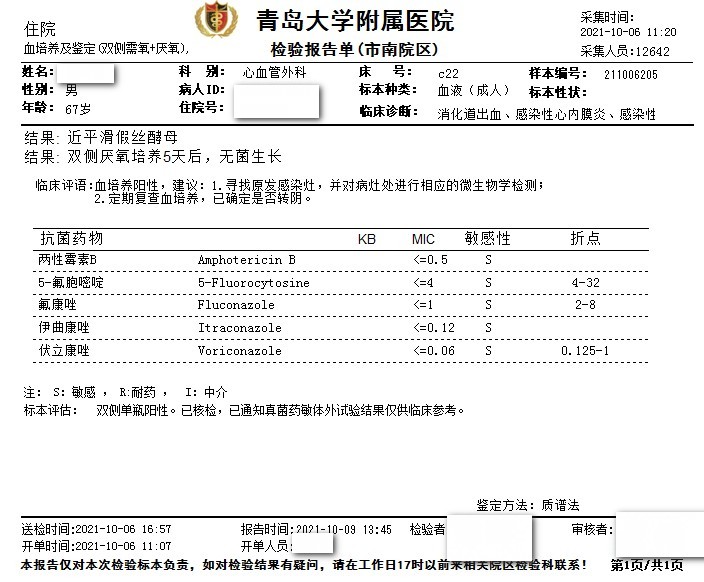

Supplement: Supplemental Information 4 [file peerj-14-20832-s004.zip › Supplement 4/245═⌡.jpg]

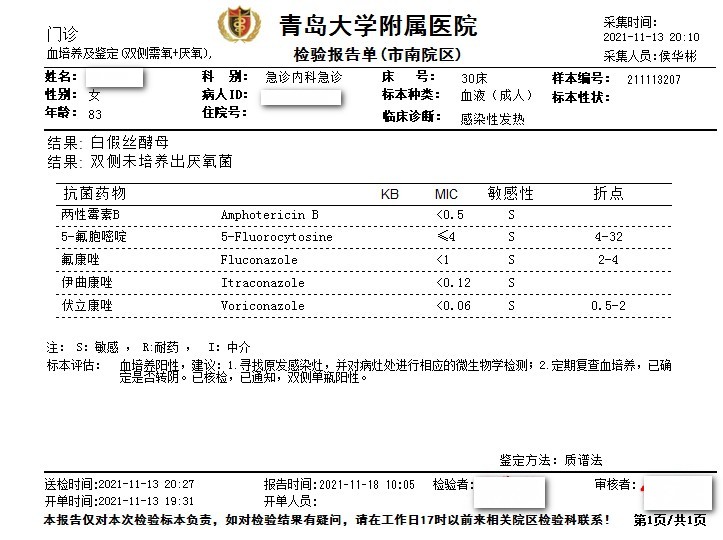

Supplement: Supplemental Information 4 [file peerj-14-20832-s004.zip › Supplement 4/246║╪.jpg]

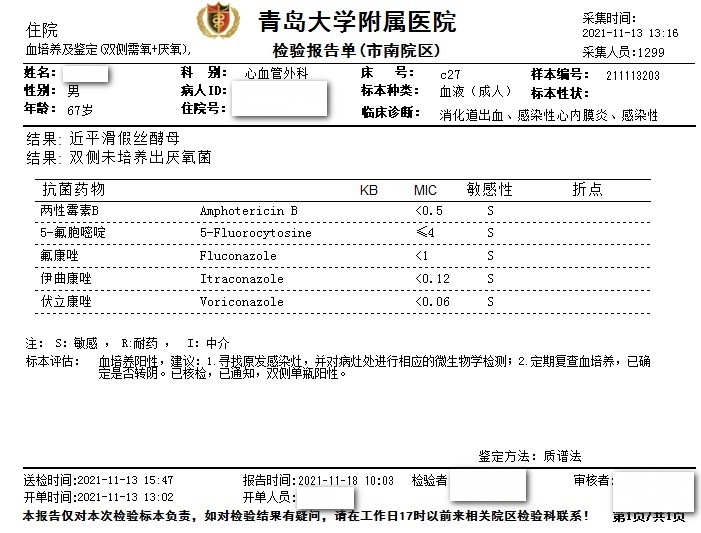

Supplement: Supplemental Information 4 [file peerj-14-20832-s004.zip › Supplement 4/247═⌡.jpg]

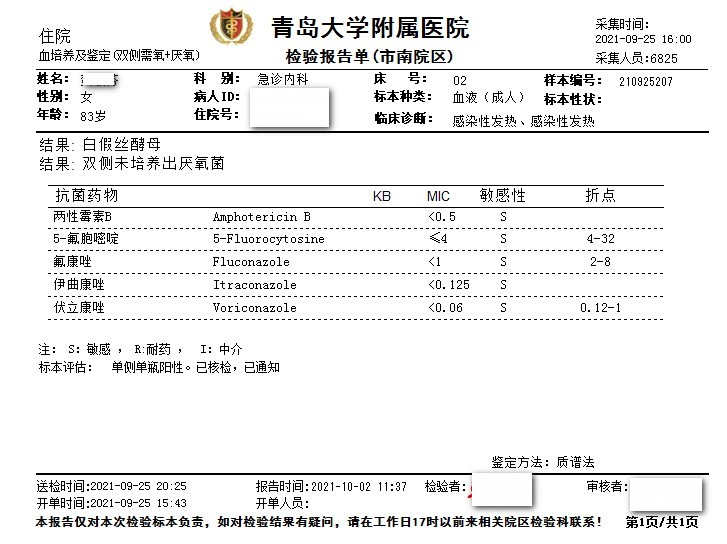

Supplement: Supplemental Information 4 [file peerj-14-20832-s004.zip › Supplement 4/248║╪.jpg]

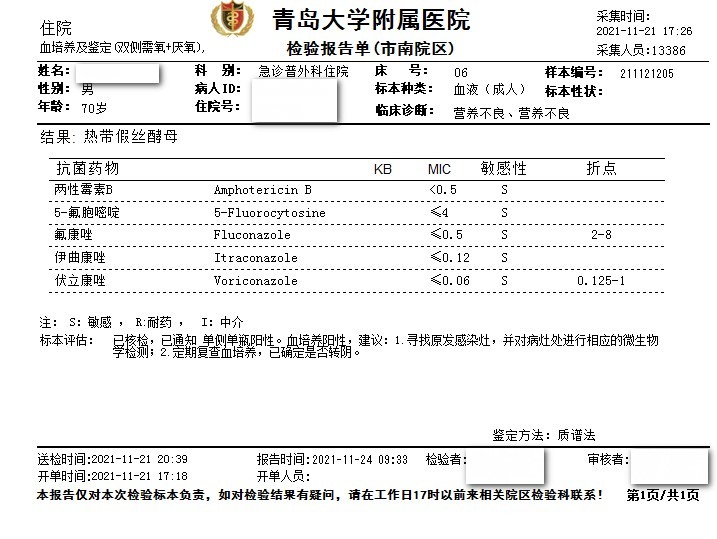

Supplement: Supplemental Information 4 [file peerj-14-20832-s004.zip › Supplement 4/249╦╬.jpg]

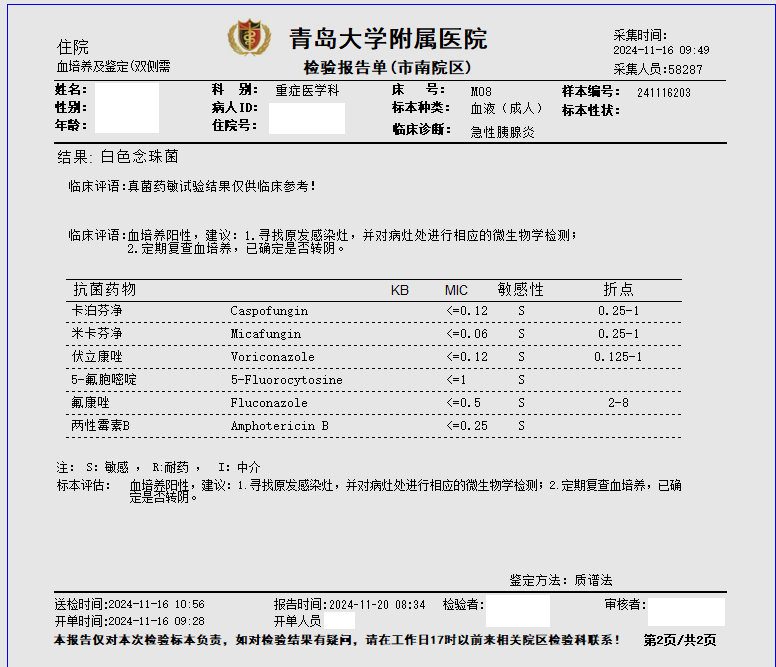

Supplement: Supplemental Information 4 [file peerj-14-20832-s004.zip › Supplement 4/25.png]

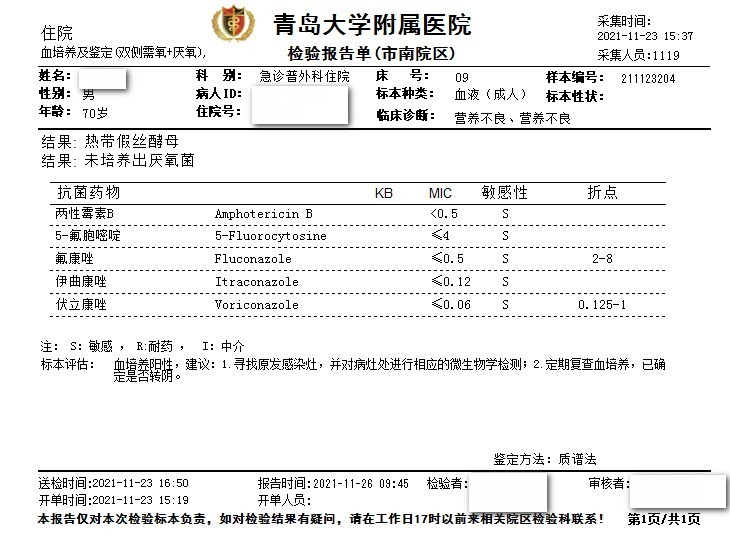

Supplement: Supplemental Information 4 [file peerj-14-20832-s004.zip › Supplement 4/250╦╬.jpg]

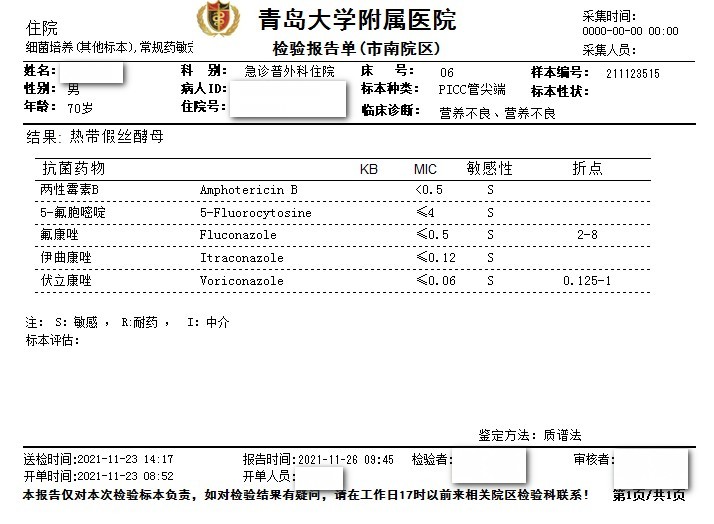

Supplement: Supplemental Information 4 [file peerj-14-20832-s004.zip › Supplement 4/251╦╬.jpg]

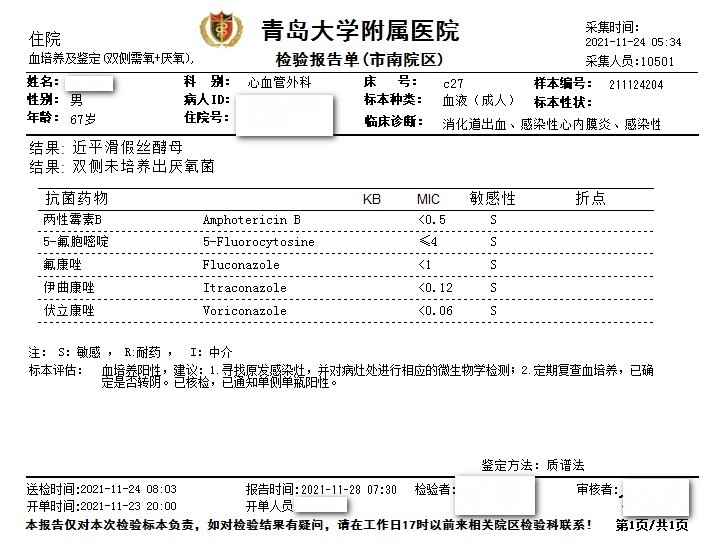

Supplement: Supplemental Information 4 [file peerj-14-20832-s004.zip › Supplement 4/252═⌡.jpg]

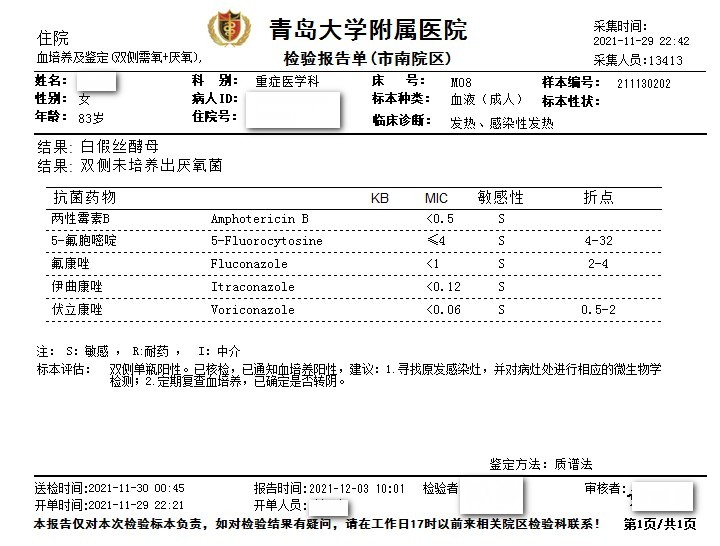

Supplement: Supplemental Information 4 [file peerj-14-20832-s004.zip › Supplement 4/253║╪.jpg]

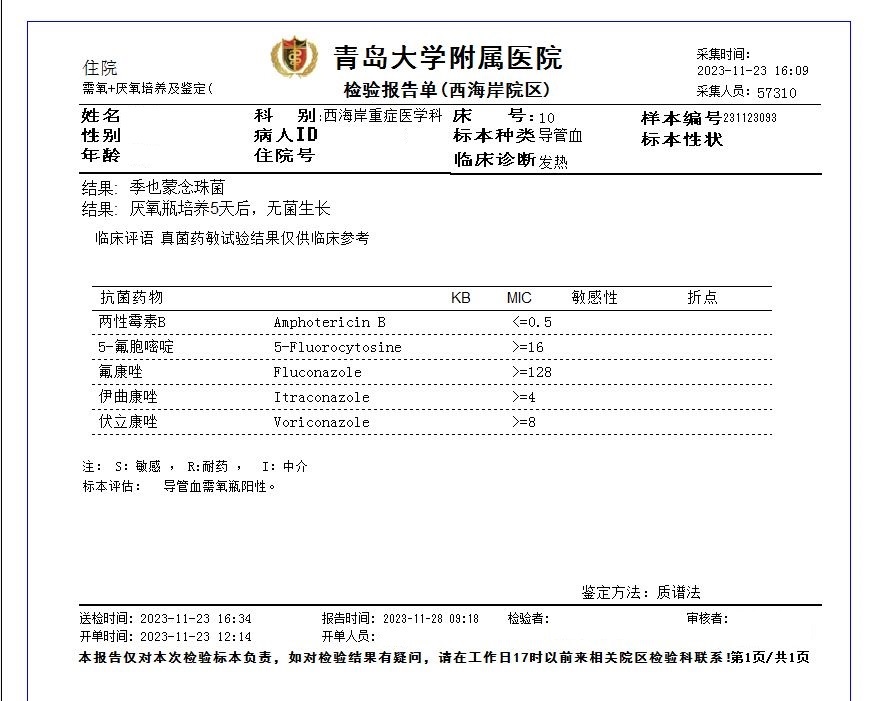

Supplement: Supplemental Information 4 [file peerj-14-20832-s004.zip › Supplement 4/254.╨φ.JPG]

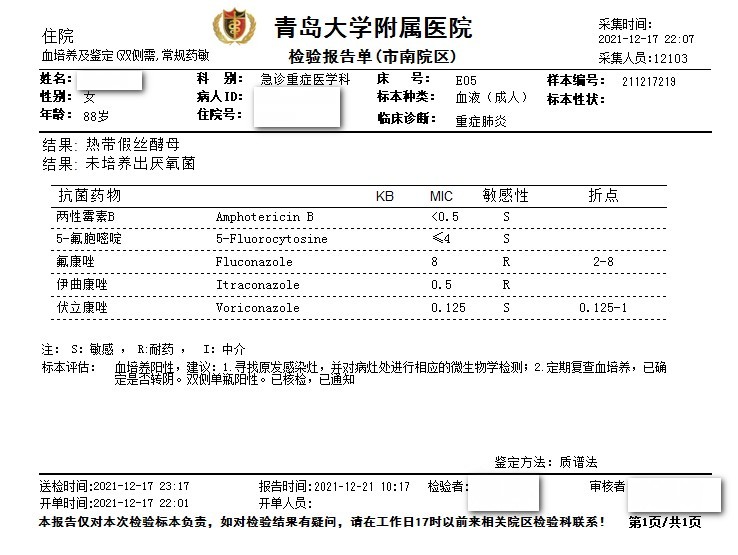

Supplement: Supplemental Information 4 [file peerj-14-20832-s004.zip › Supplement 4/255╖╢.jpg]

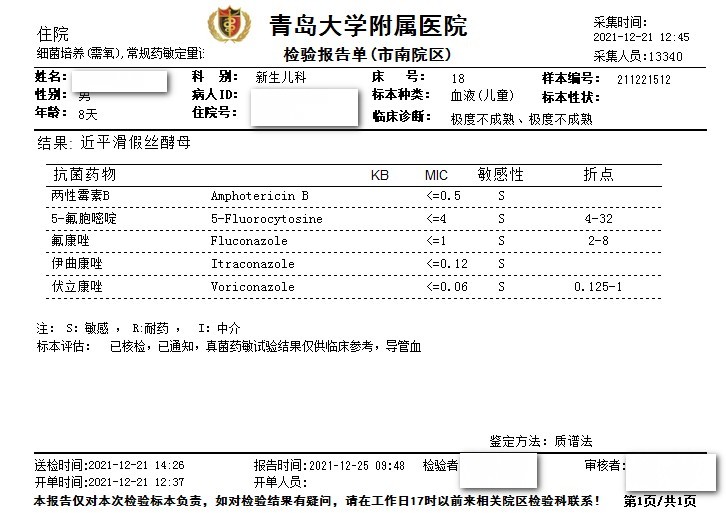

Supplement: Supplemental Information 4 [file peerj-14-20832-s004.zip › Supplement 4/256┬└.jpg]

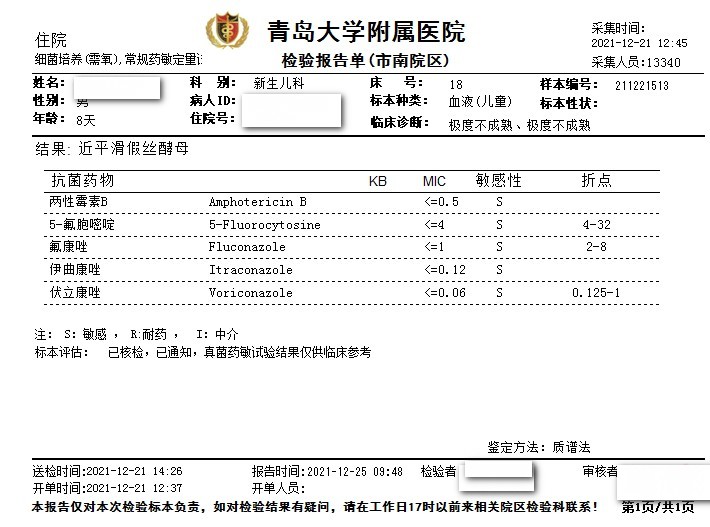

Supplement: Supplemental Information 4 [file peerj-14-20832-s004.zip › Supplement 4/257┬└.jpg]

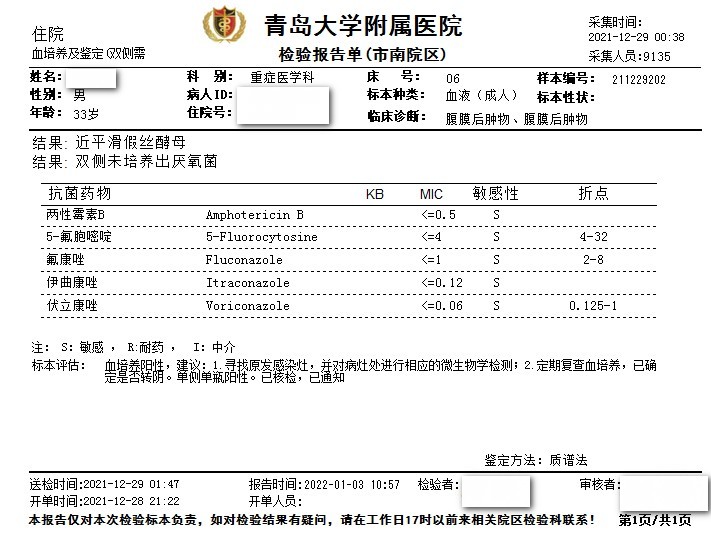

Supplement: Supplemental Information 4 [file peerj-14-20832-s004.zip › Supplement 4/258╔█.jpg]

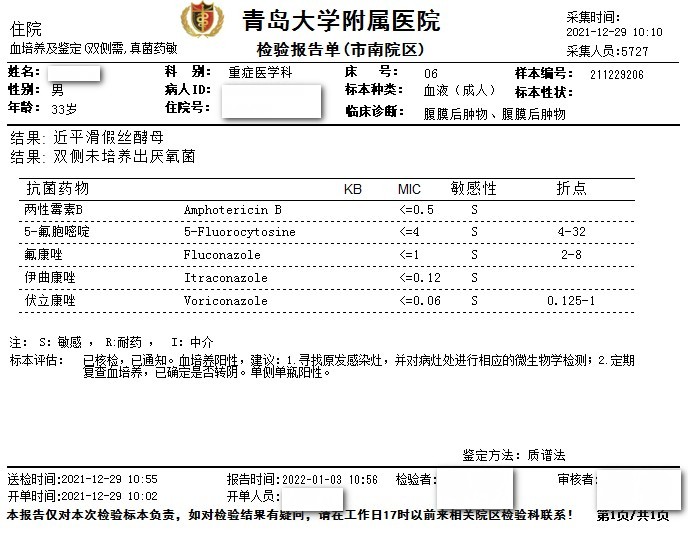

Supplement: Supplemental Information 4 [file peerj-14-20832-s004.zip › Supplement 4/259╔█.jpg]

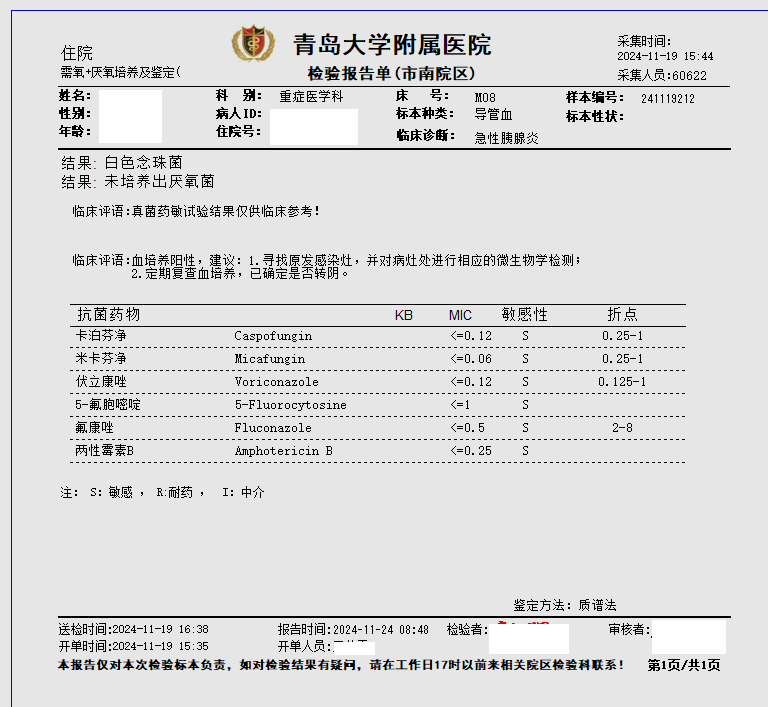

Supplement: Supplemental Information 4 [file peerj-14-20832-s004.zip › Supplement 4/26.png]

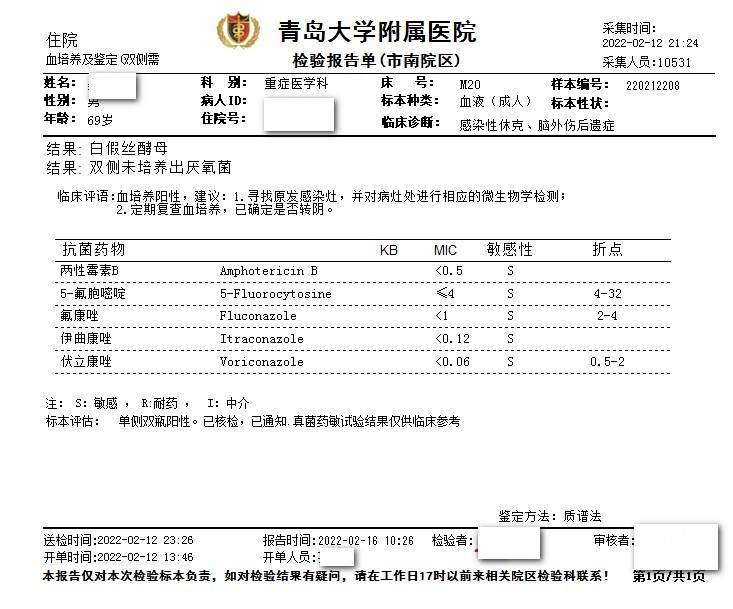

Supplement: Supplemental Information 4 [file peerj-14-20832-s004.zip › Supplement 4/260╜¬.jpg]

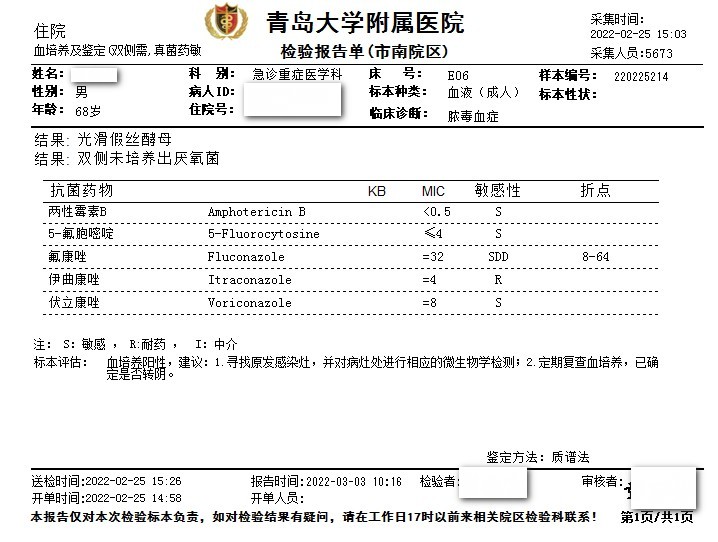

Supplement: Supplemental Information 4 [file peerj-14-20832-s004.zip › Supplement 4/261├╧.jpg]

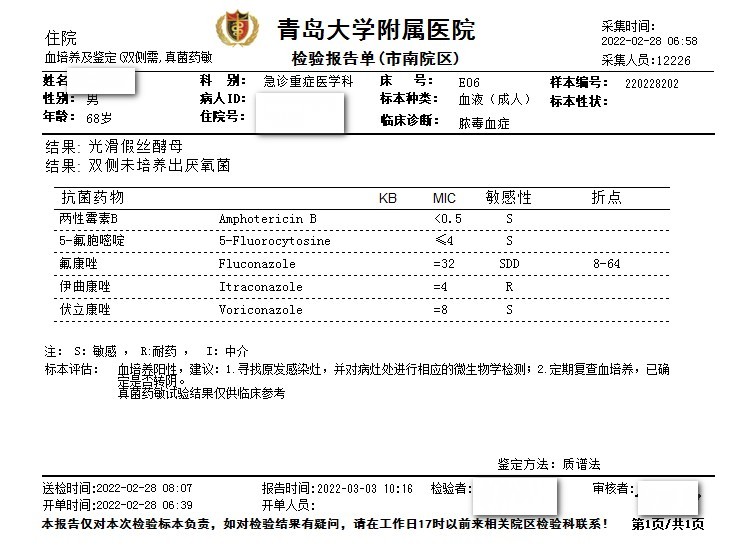

Supplement: Supplemental Information 4 [file peerj-14-20832-s004.zip › Supplement 4/262├╧.jpg]

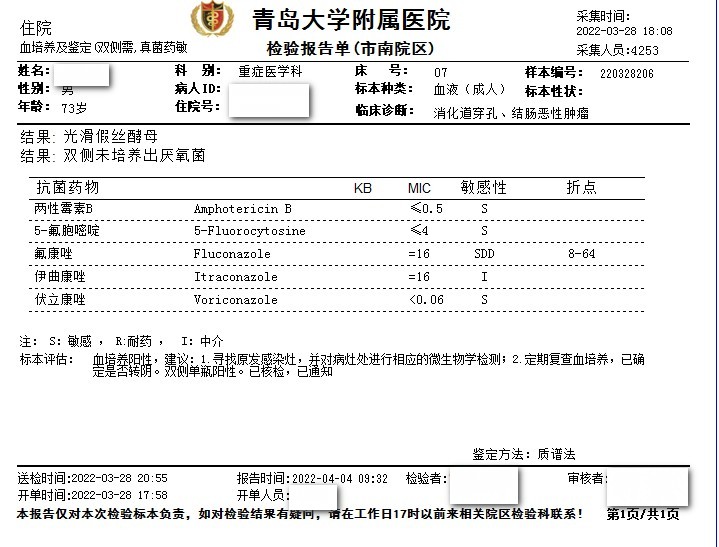

Supplement: Supplemental Information 4 [file peerj-14-20832-s004.zip › Supplement 4/263└ε.jpg]

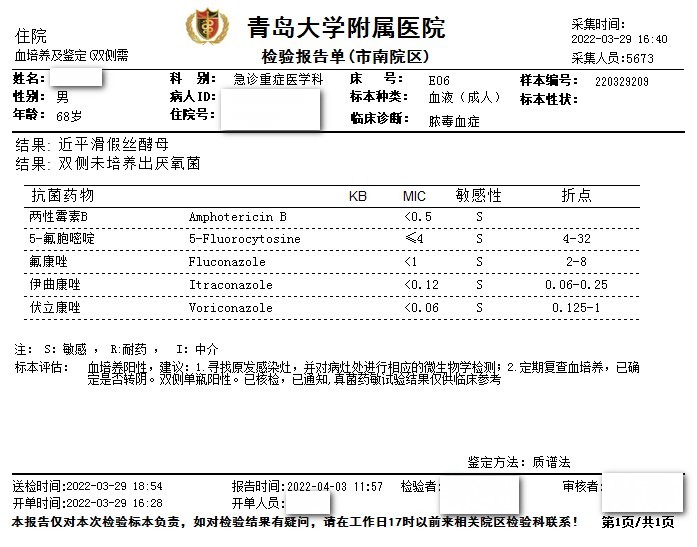

Supplement: Supplemental Information 4 [file peerj-14-20832-s004.zip › Supplement 4/264├╧.jpg]

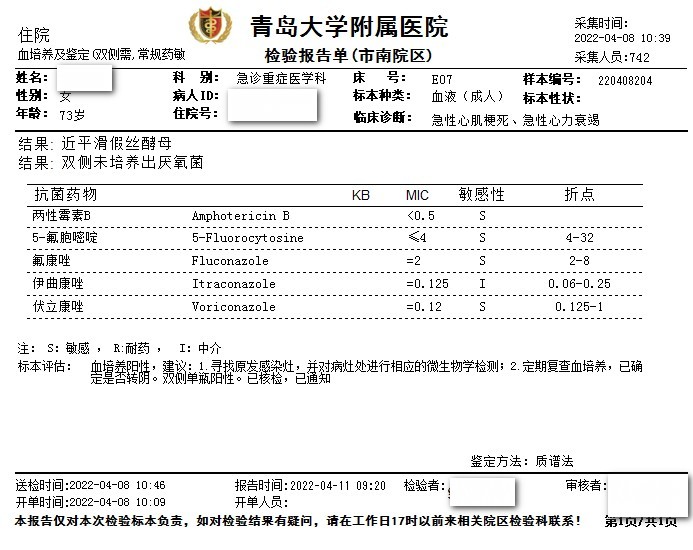

Supplement: Supplemental Information 4 [file peerj-14-20832-s004.zip › Supplement 4/265└ε.jpg]

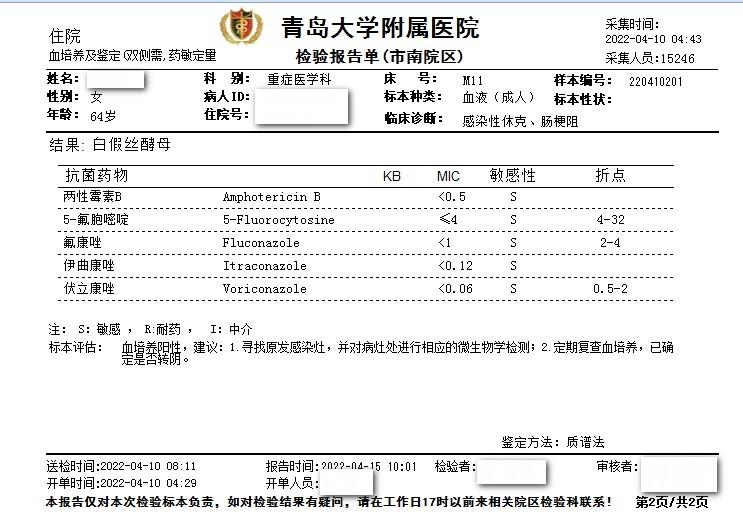

Supplement: Supplemental Information 4 [file peerj-14-20832-s004.zip › Supplement 4/266┴⌡.jpg]

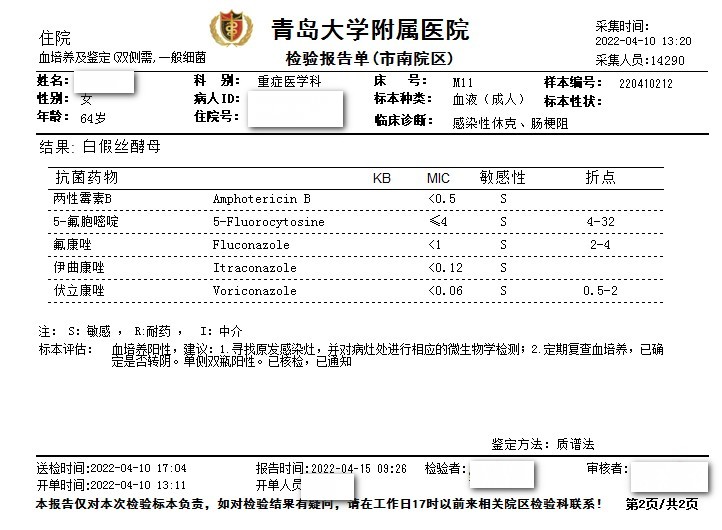

Supplement: Supplemental Information 4 [file peerj-14-20832-s004.zip › Supplement 4/267┴⌡jpg.jpg]

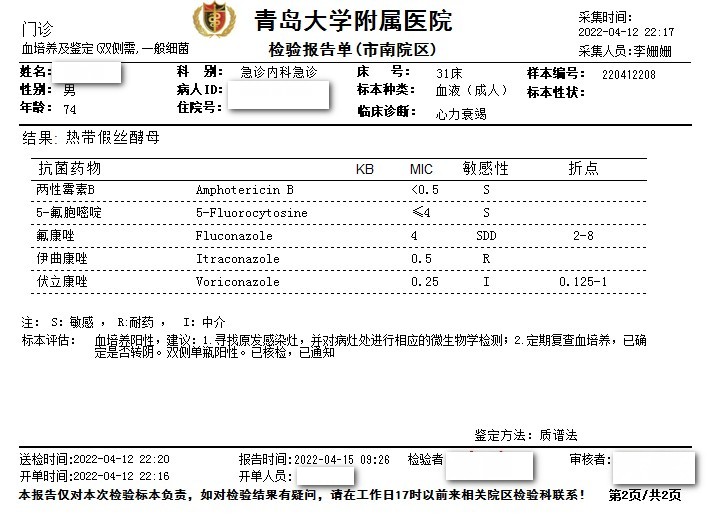

Supplement: Supplemental Information 4 [file peerj-14-20832-s004.zip › Supplement 4/268╚╬.jpg]

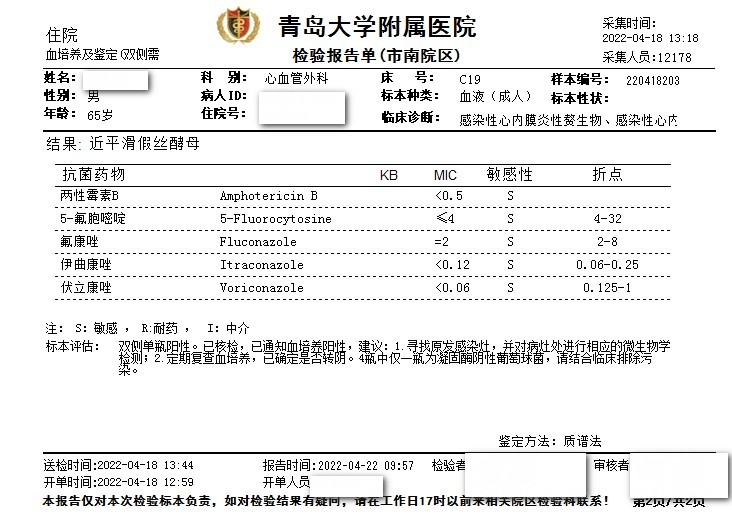

Supplement: Supplemental Information 4 [file peerj-14-20832-s004.zip › Supplement 4/269═⌡.jpg]

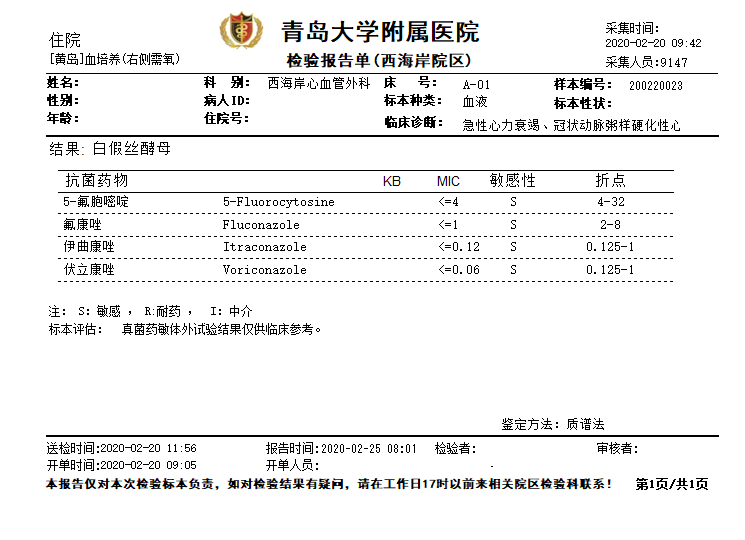

Supplement: Supplemental Information 4 [file peerj-14-20832-s004.zip › Supplement 4/27.png]

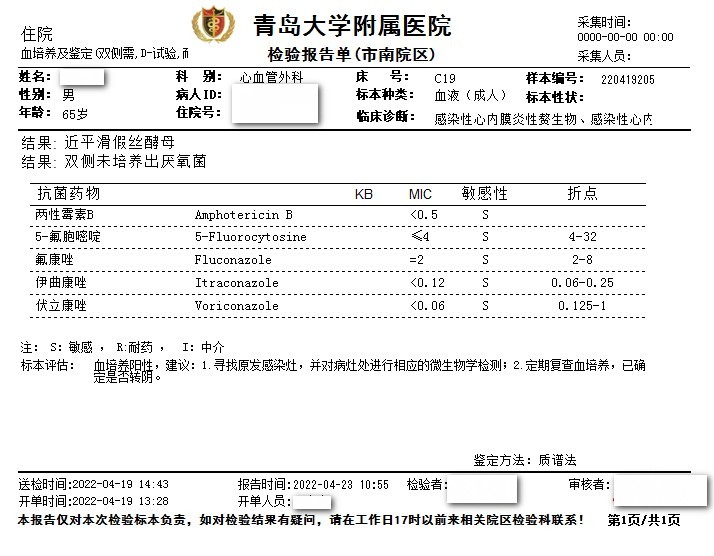

Supplement: Supplemental Information 4 [file peerj-14-20832-s004.zip › Supplement 4/270═⌡.jpg]

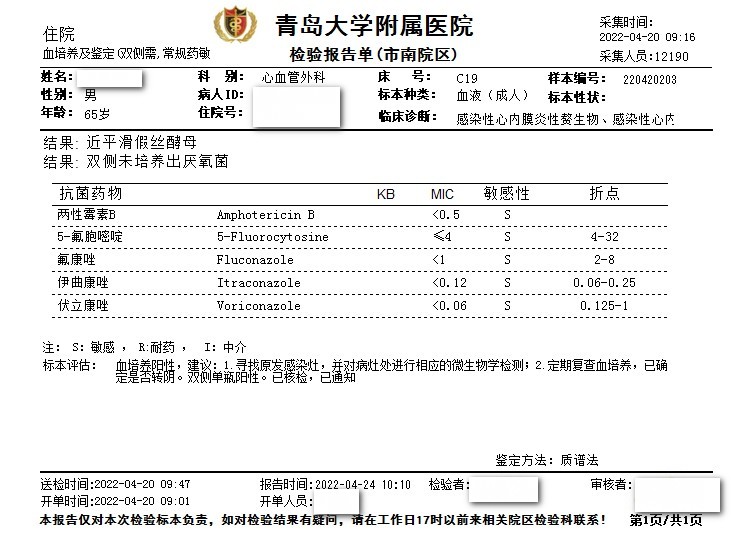

Supplement: Supplemental Information 4 [file peerj-14-20832-s004.zip › Supplement 4/271═⌡.jpg]

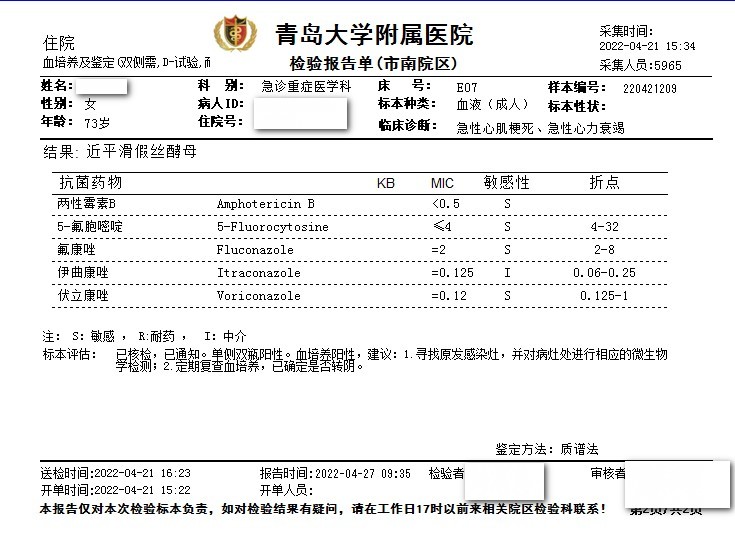

Supplement: Supplemental Information 4 [file peerj-14-20832-s004.zip › Supplement 4/272└ε.jpg]

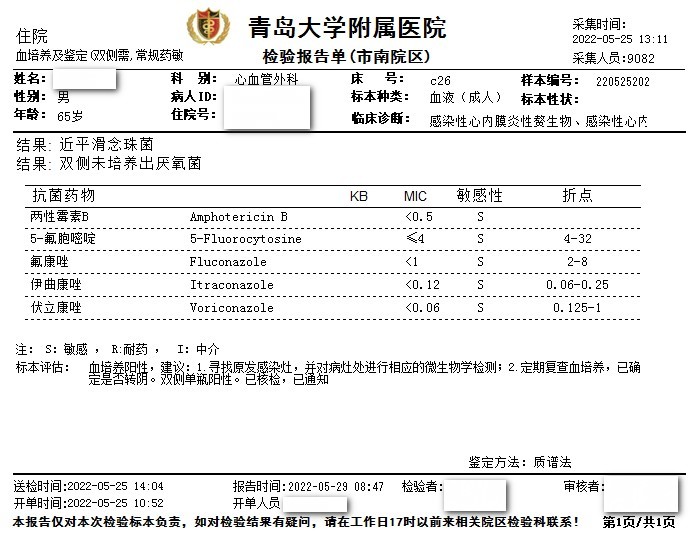

Supplement: Supplemental Information 4 [file peerj-14-20832-s004.zip › Supplement 4/273.jpg]

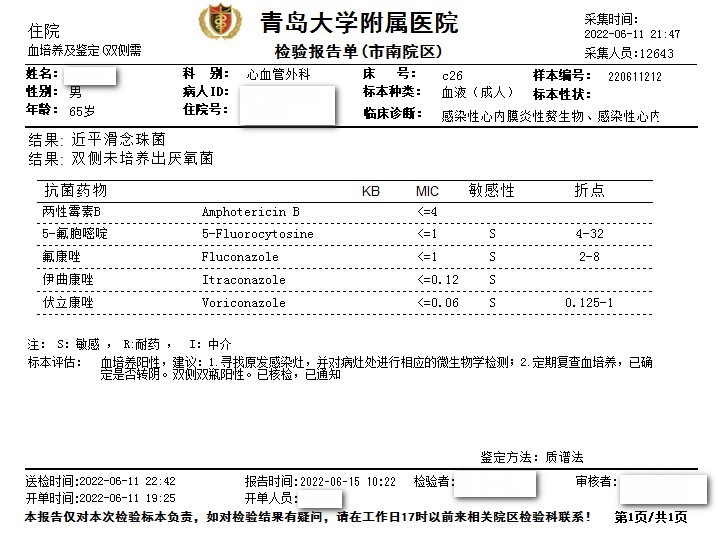

Supplement: Supplemental Information 4 [file peerj-14-20832-s004.zip › Supplement 4/274.jpg]

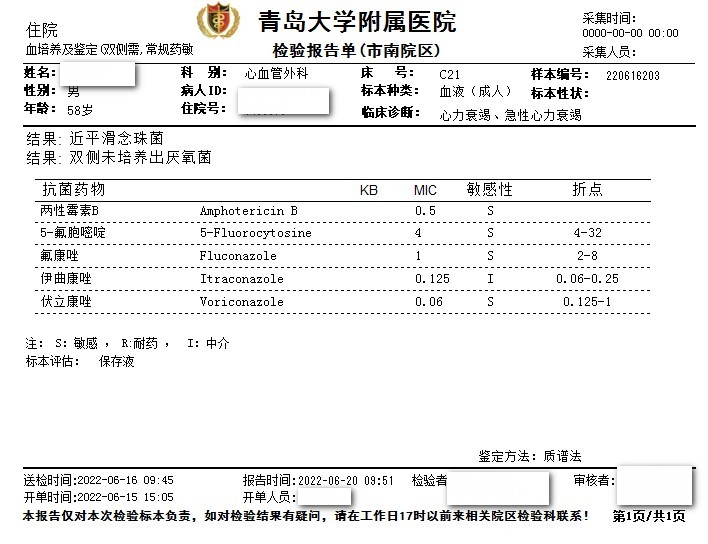

Supplement: Supplemental Information 4 [file peerj-14-20832-s004.zip › Supplement 4/275.jpg]

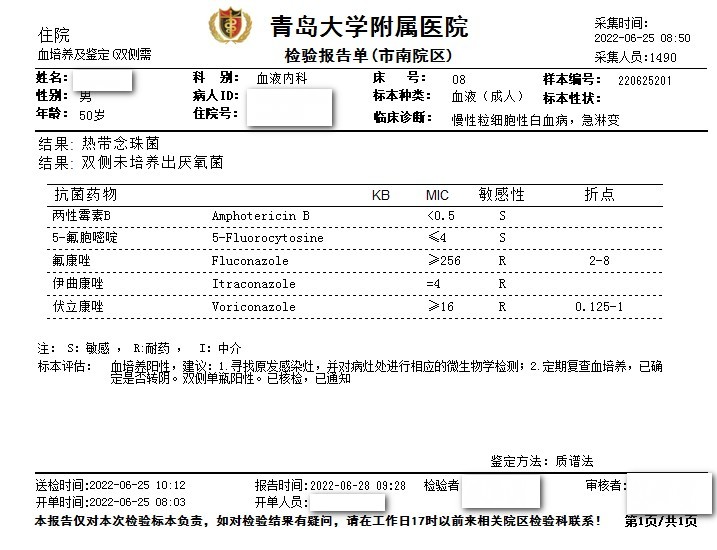

Supplement: Supplemental Information 4 [file peerj-14-20832-s004.zip › Supplement 4/276.jpg]

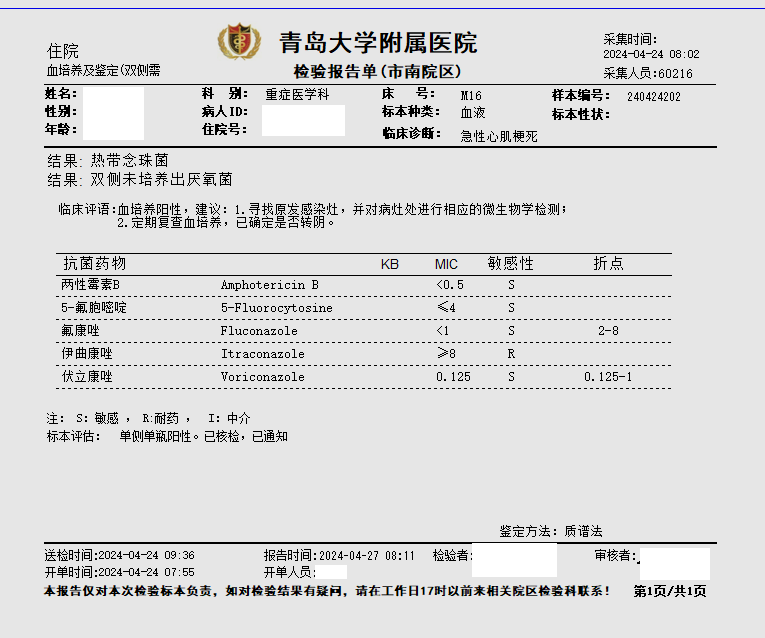

Supplement: Supplemental Information 4 [file peerj-14-20832-s004.zip › Supplement 4/28.png]

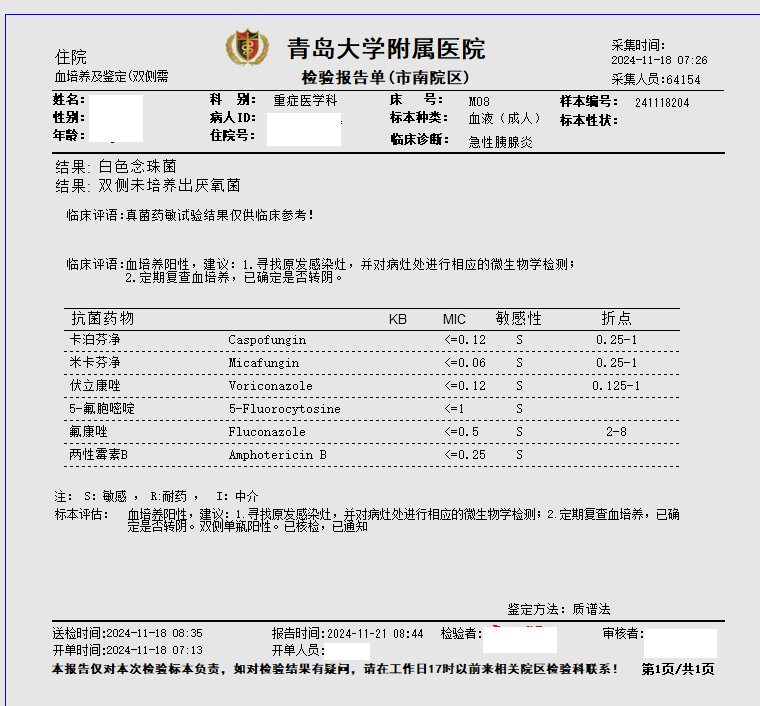

Supplement: Supplemental Information 4 [file peerj-14-20832-s004.zip › Supplement 4/29.png]

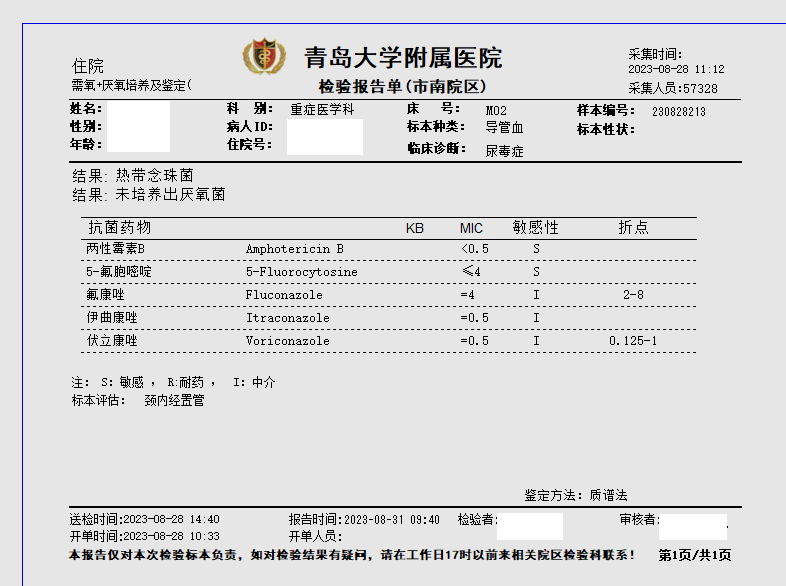

Supplement: Supplemental Information 4 [file peerj-14-20832-s004.zip › Supplement 4/3.png]
