# Supplementary figures and images for: Candida species distribution, antifungal susceptibility and trends causing candidemia: a 10-year observation in eastern China (part 1 of 2)
Source: PeerJ. 2026 Mar 5;14:e20832. doi: 10.7717/peerj.20832 (PMC12967414; doi:10.7717/peerj.20832)

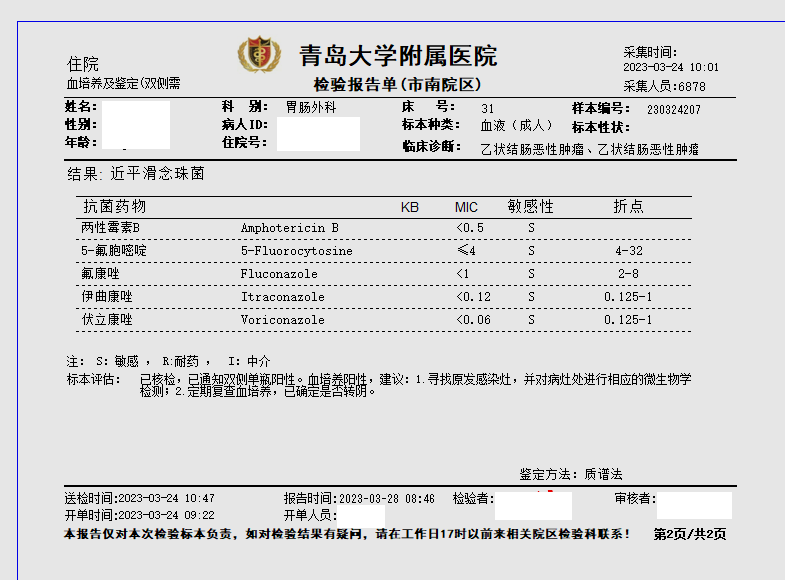

Supplement: Supplemental Information 4 [file peerj-14-20832-s004.zip › Supplement 4/1.png]

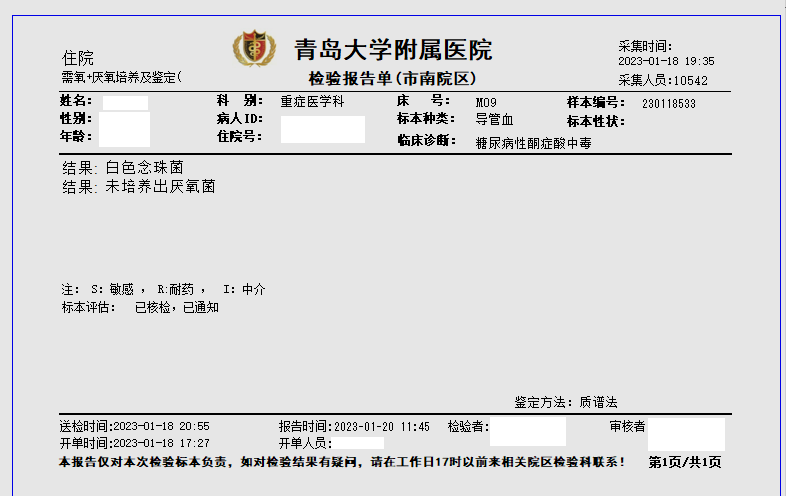

Supplement: Supplemental Information 4 [file peerj-14-20832-s004.zip › Supplement 4/10.PNG]

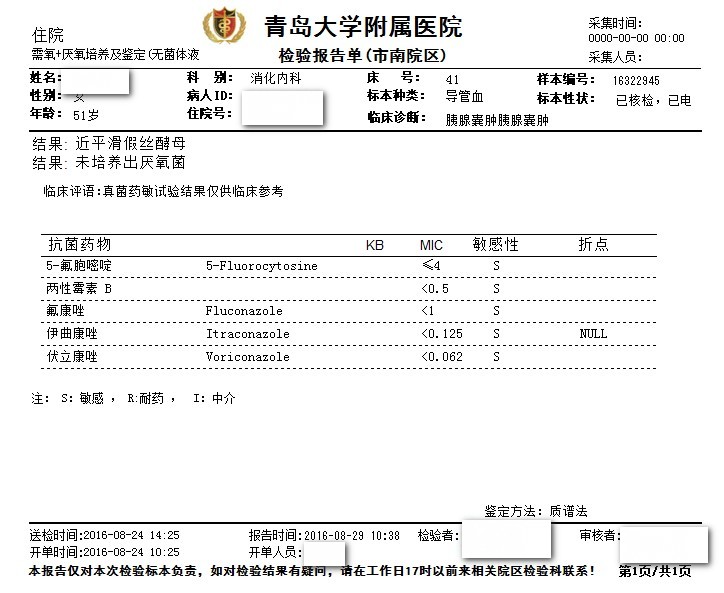

Supplement: Supplemental Information 4 [file peerj-14-20832-s004.zip › Supplement 4/100║·.jpg]

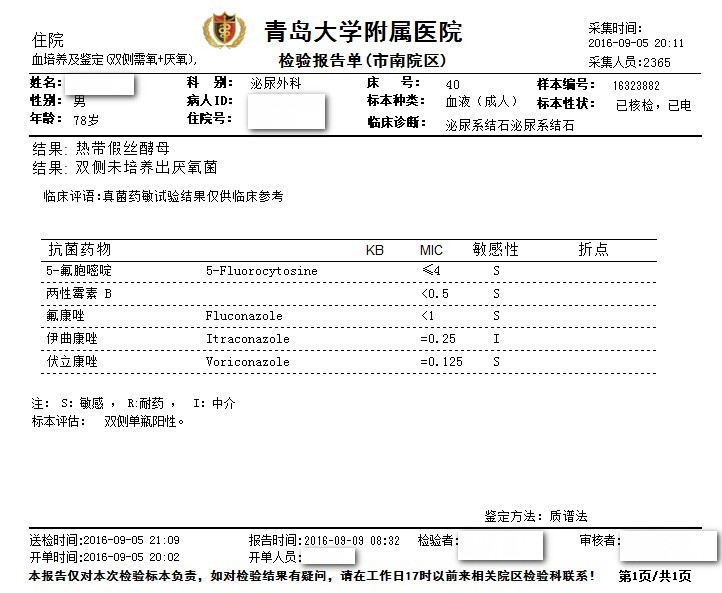

Supplement: Supplemental Information 4 [file peerj-14-20832-s004.zip › Supplement 4/101╬Γ.jpg]

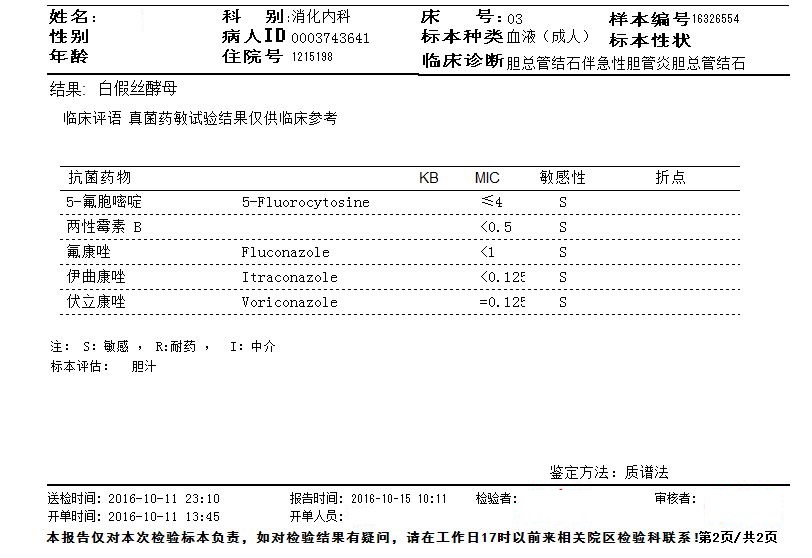

Supplement: Supplemental Information 4 [file peerj-14-20832-s004.zip › Supplement 4/102╒┼.jpg]

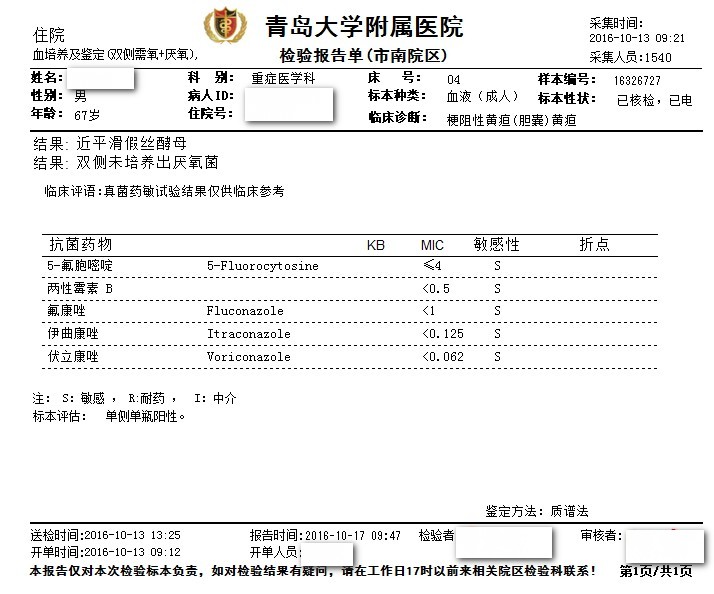

Supplement: Supplemental Information 4 [file peerj-14-20832-s004.zip › Supplement 4/103╓▄.jpg]

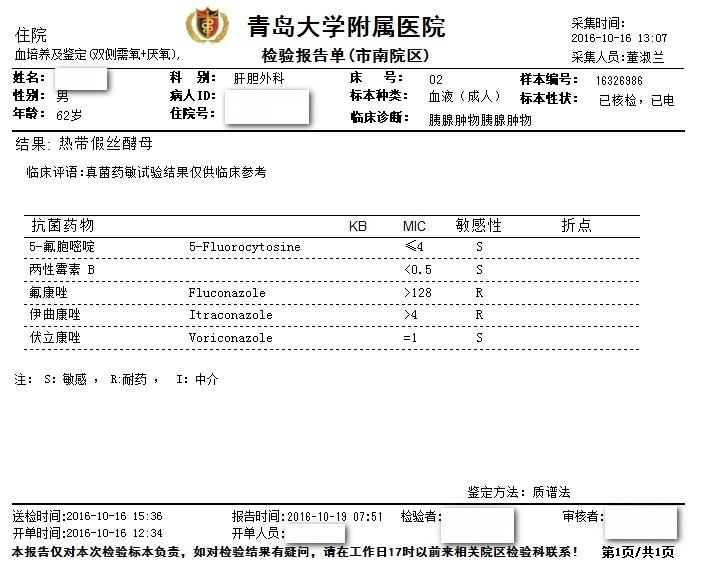

Supplement: Supplemental Information 4 [file peerj-14-20832-s004.zip › Supplement 4/104└ε.jpg]

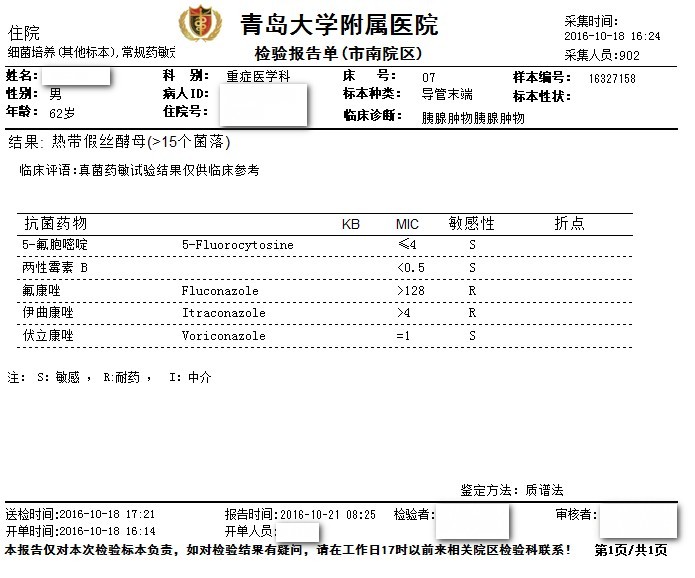

Supplement: Supplemental Information 4 [file peerj-14-20832-s004.zip › Supplement 4/105└ε.jpg]

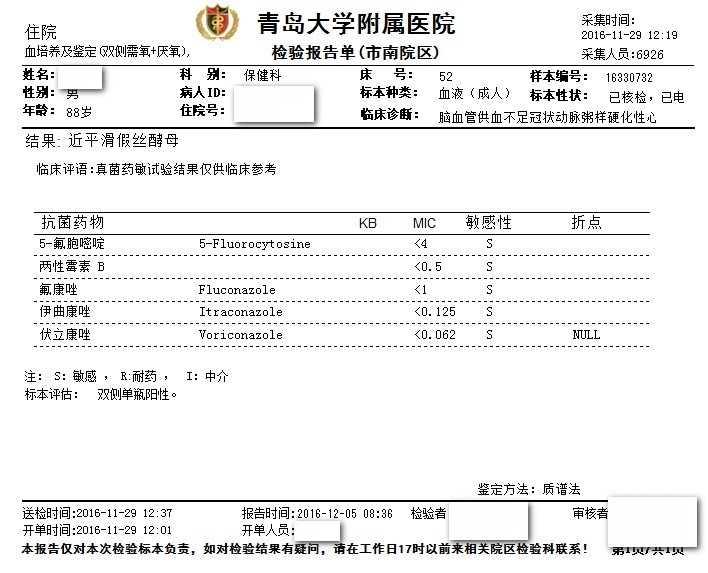

Supplement: Supplemental Information 4 [file peerj-14-20832-s004.zip › Supplement 4/106╙┌.jpg]

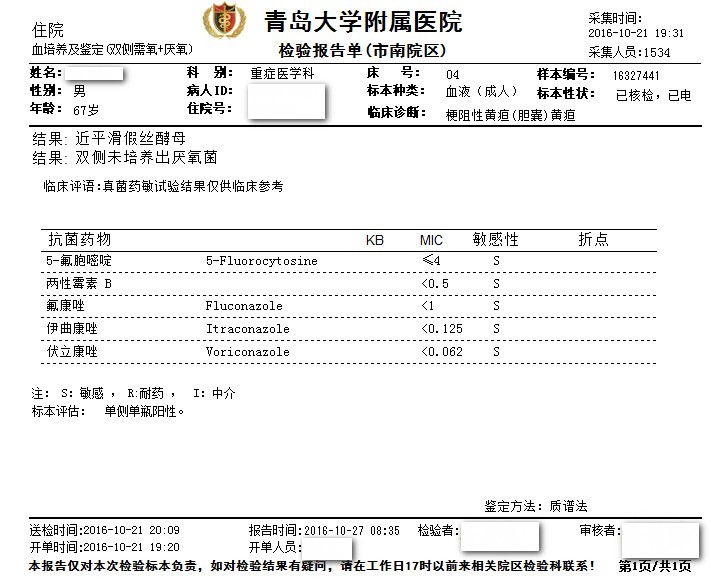

Supplement: Supplemental Information 4 [file peerj-14-20832-s004.zip › Supplement 4/107╓▄.jpg]

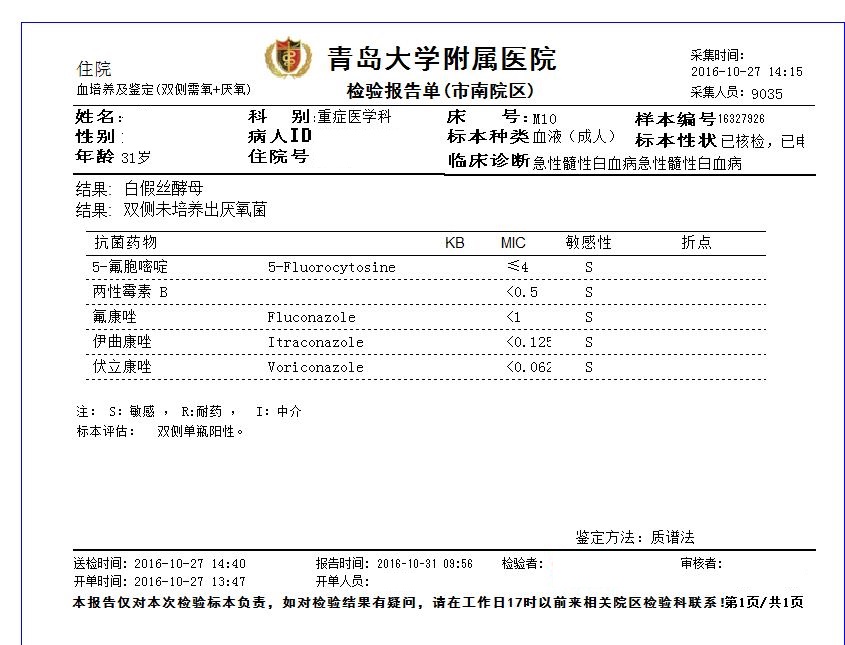

Supplement: Supplemental Information 4 [file peerj-14-20832-s004.zip › Supplement 4/108╙┌.jpg]

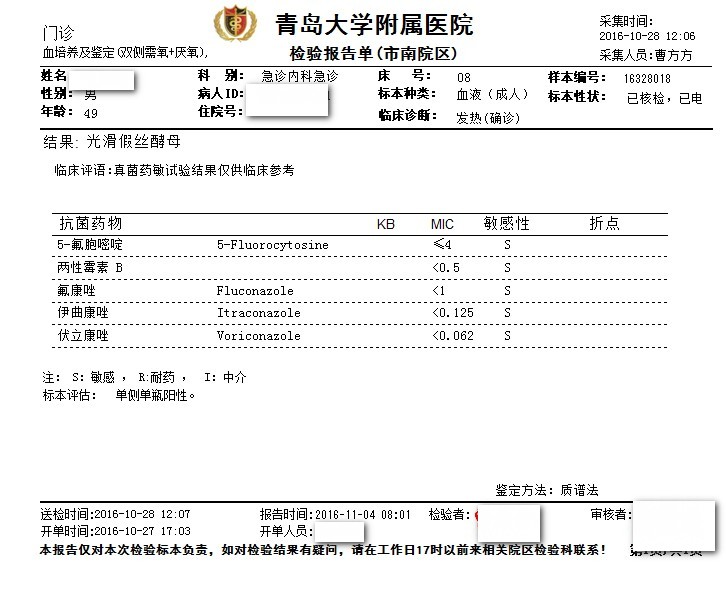

Supplement: Supplemental Information 4 [file peerj-14-20832-s004.zip › Supplement 4/109└ε.jpg]

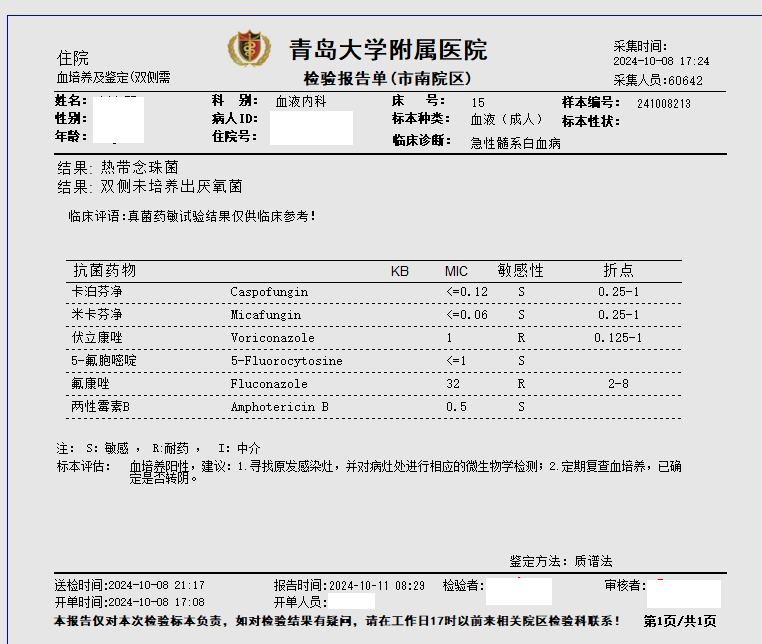

Supplement: Supplemental Information 4 [file peerj-14-20832-s004.zip › Supplement 4/11.png]

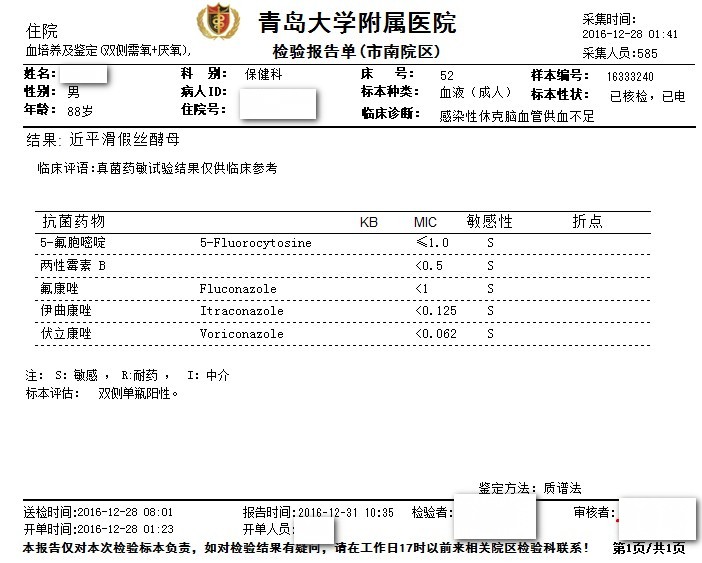

Supplement: Supplemental Information 4 [file peerj-14-20832-s004.zip › Supplement 4/110╙┌.jpg]

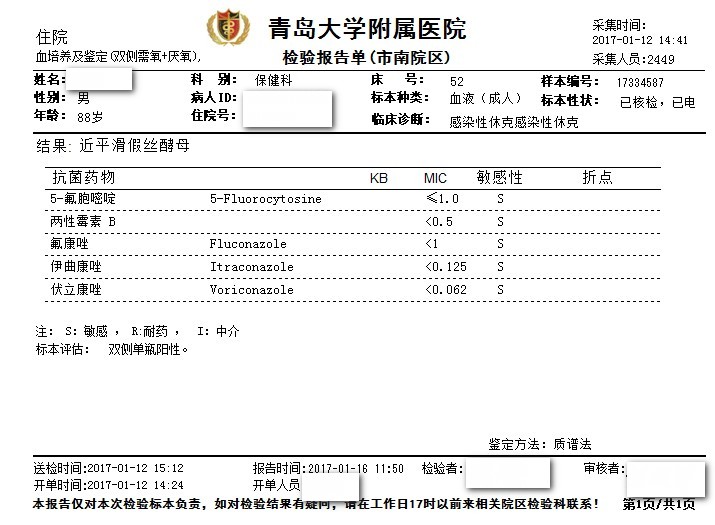

Supplement: Supplemental Information 4 [file peerj-14-20832-s004.zip › Supplement 4/111╙┌.jpg]

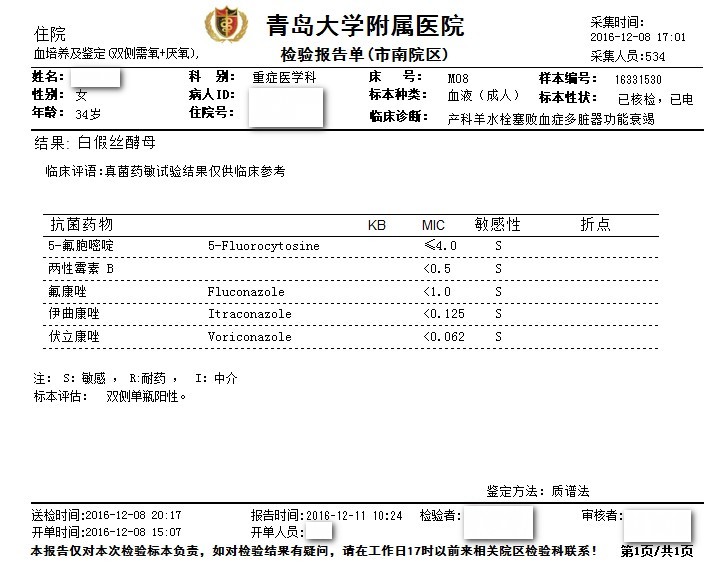

Supplement: Supplemental Information 4 [file peerj-14-20832-s004.zip › Supplement 4/112│┬.jpg]

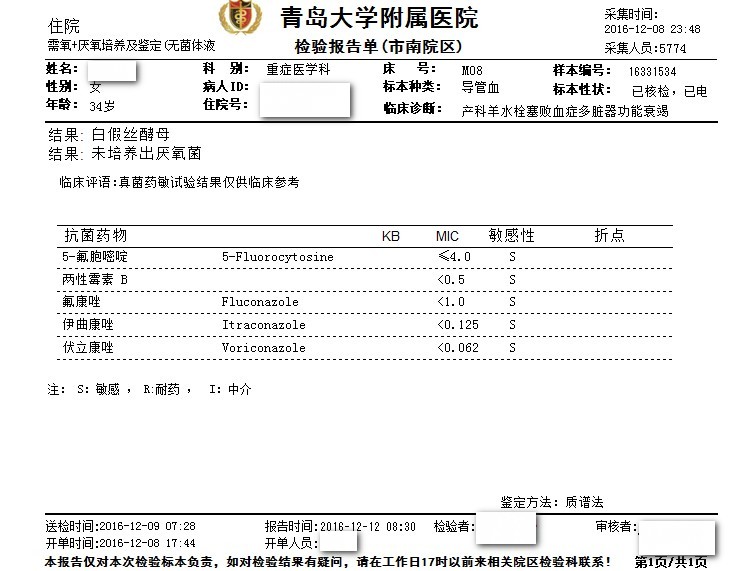

Supplement: Supplemental Information 4 [file peerj-14-20832-s004.zip › Supplement 4/113│┬.jpg]

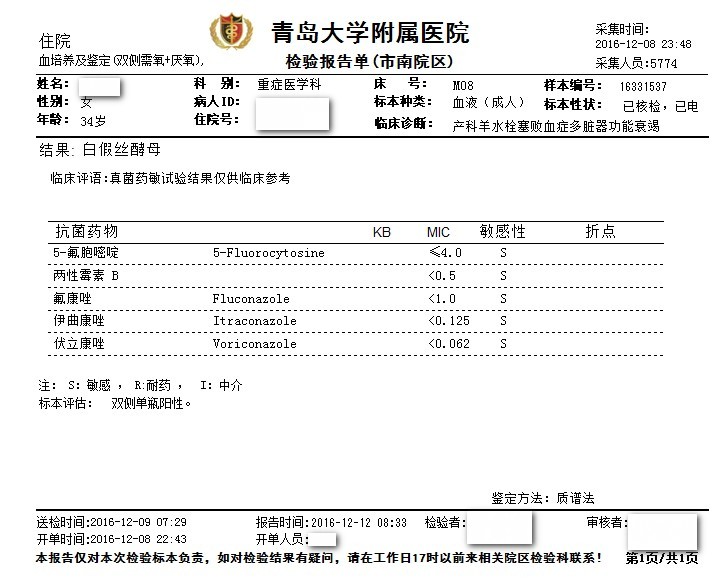

Supplement: Supplemental Information 4 [file peerj-14-20832-s004.zip › Supplement 4/114│┬.jpg]

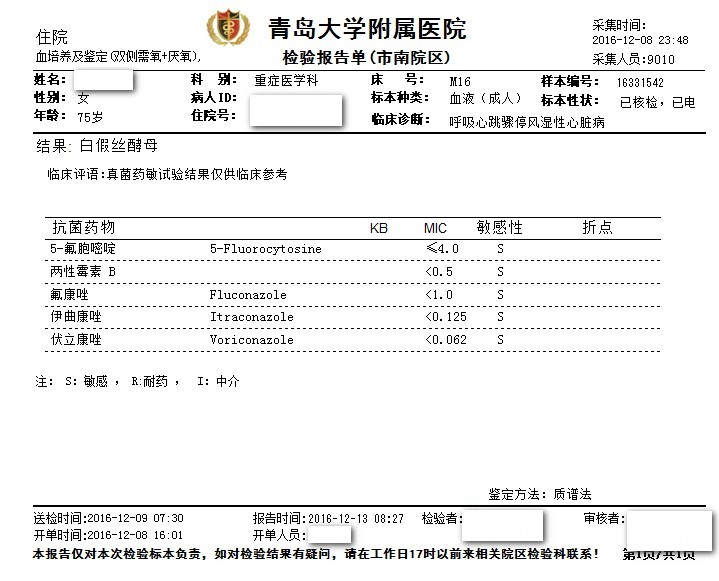

Supplement: Supplemental Information 4 [file peerj-14-20832-s004.zip › Supplement 4/115└ε.jpg]

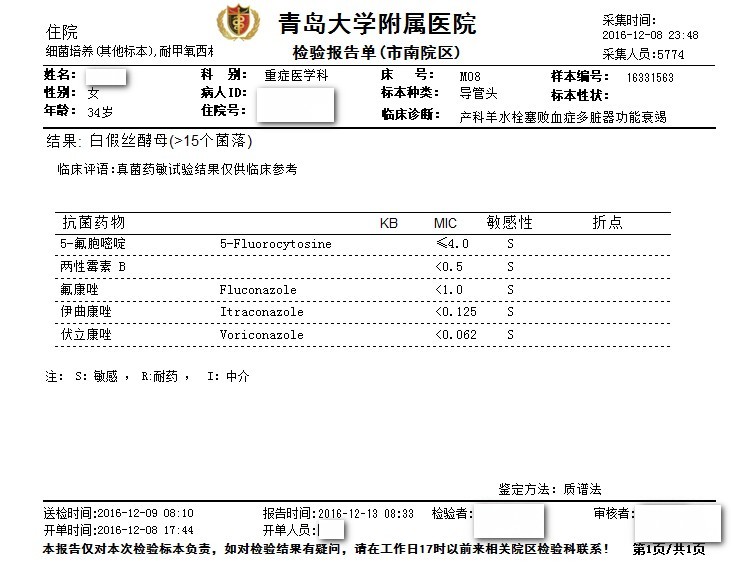

Supplement: Supplemental Information 4 [file peerj-14-20832-s004.zip › Supplement 4/116│┬.jpg]

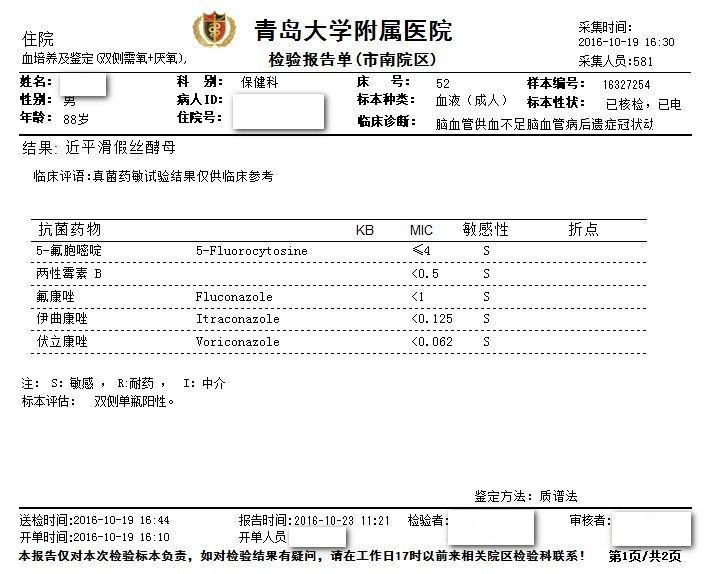

Supplement: Supplemental Information 4 [file peerj-14-20832-s004.zip › Supplement 4/117╙┌.jpg]

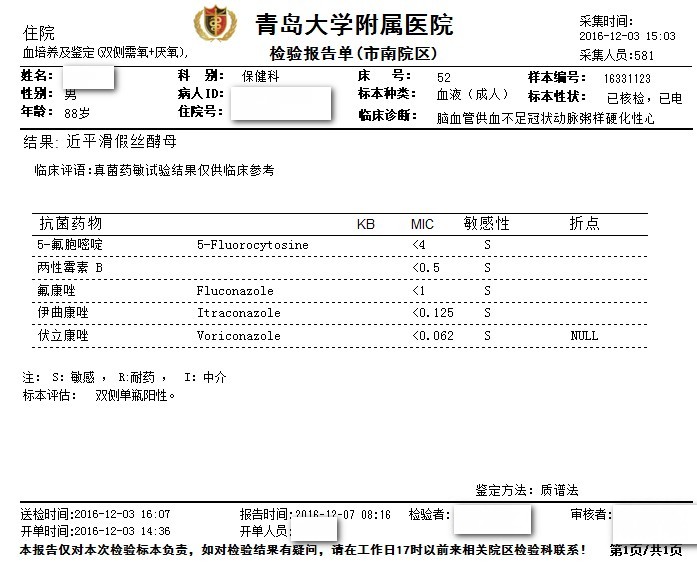

Supplement: Supplemental Information 4 [file peerj-14-20832-s004.zip › Supplement 4/118╙┌.jpg]

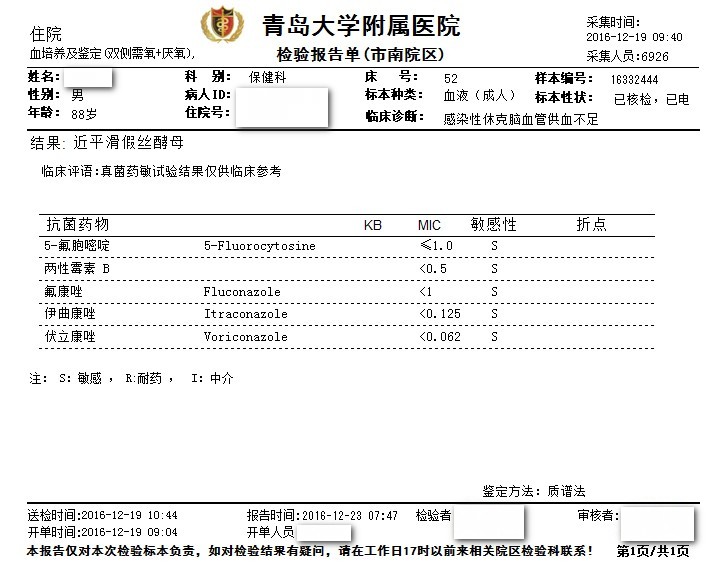

Supplement: Supplemental Information 4 [file peerj-14-20832-s004.zip › Supplement 4/119╙┌.jpg]

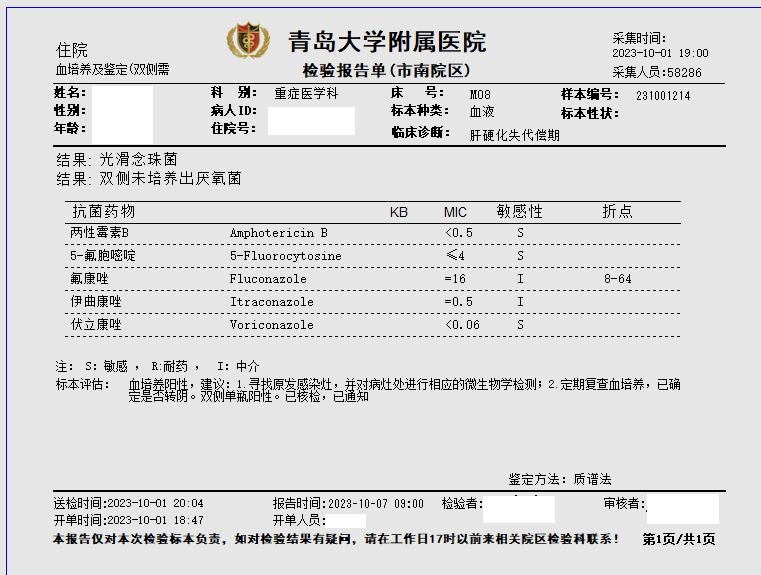

Supplement: Supplemental Information 4 [file peerj-14-20832-s004.zip › Supplement 4/12.png]

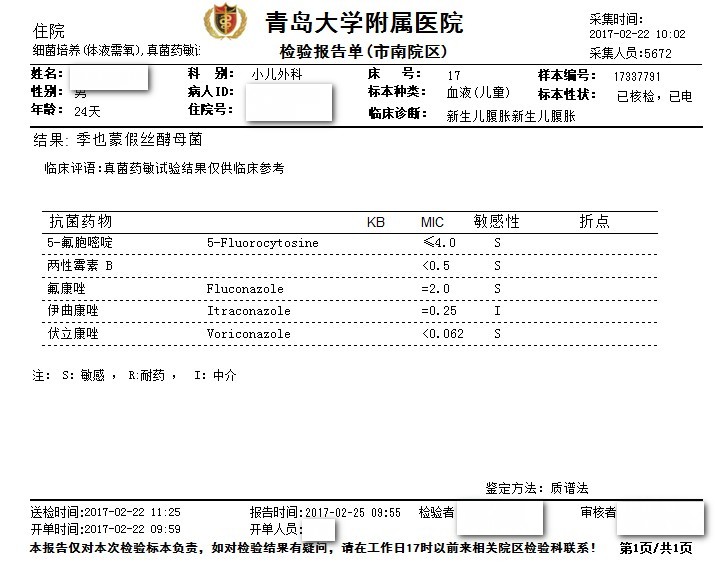

Supplement: Supplemental Information 4 [file peerj-14-20832-s004.zip › Supplement 4/120═⌡.jpg]

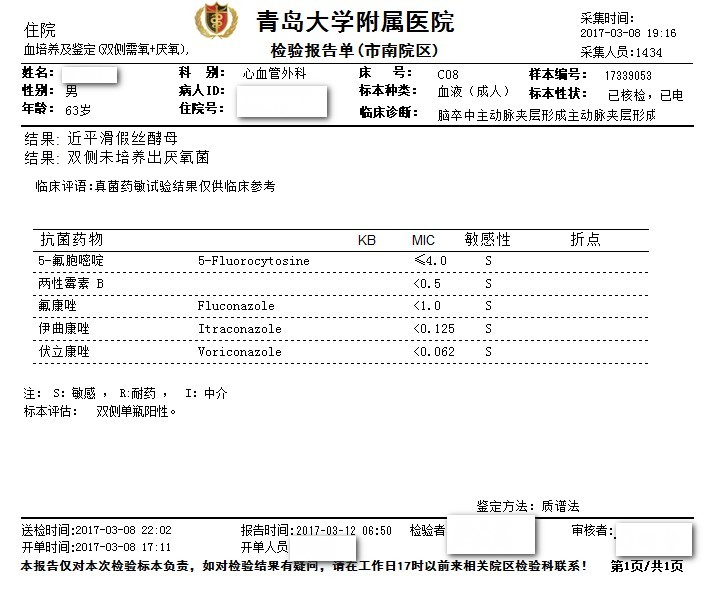

Supplement: Supplemental Information 4 [file peerj-14-20832-s004.zip › Supplement 4/121╦╒jpg.jpg]

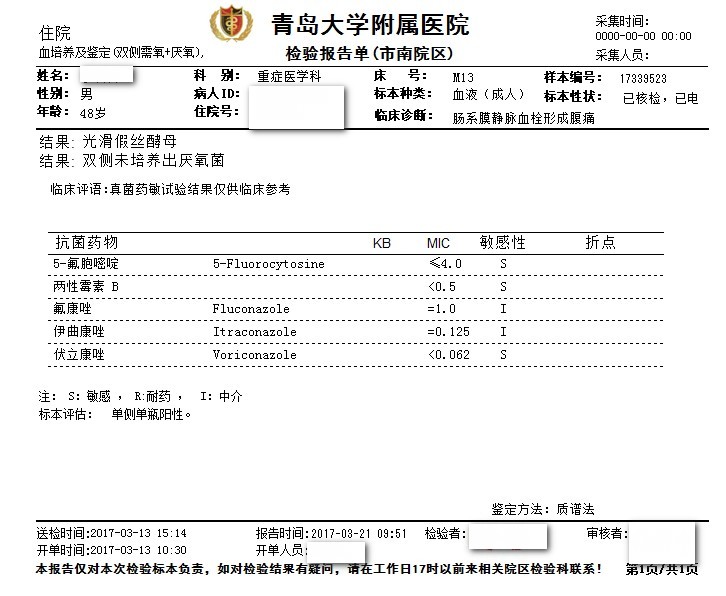

Supplement: Supplemental Information 4 [file peerj-14-20832-s004.zip › Supplement 4/122└εjpg.jpg]

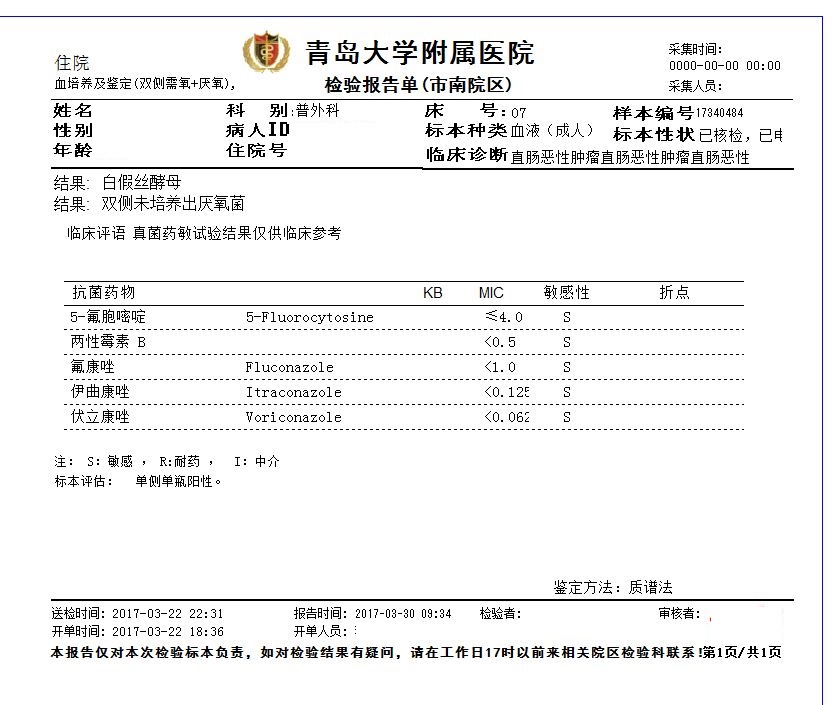

Supplement: Supplemental Information 4 [file peerj-14-20832-s004.zip › Supplement 4/123═⌡╒╫.jpg]

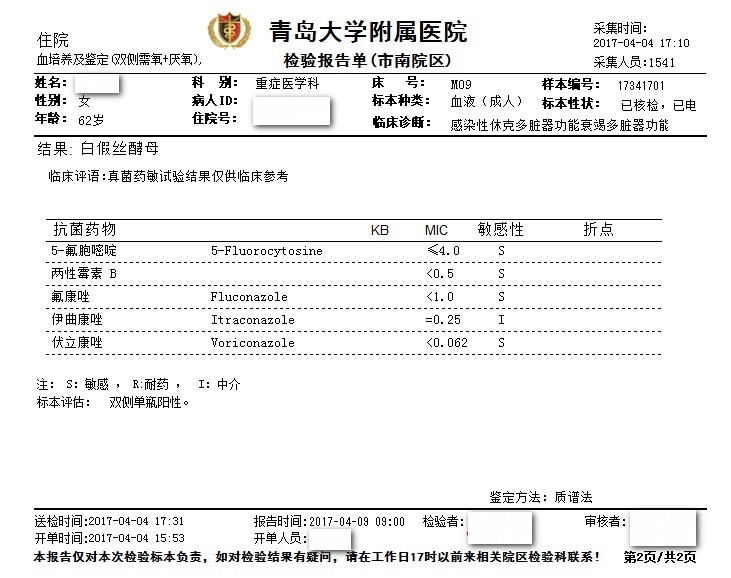

Supplement: Supplemental Information 4 [file peerj-14-20832-s004.zip › Supplement 4/124.╛╧.jpg]

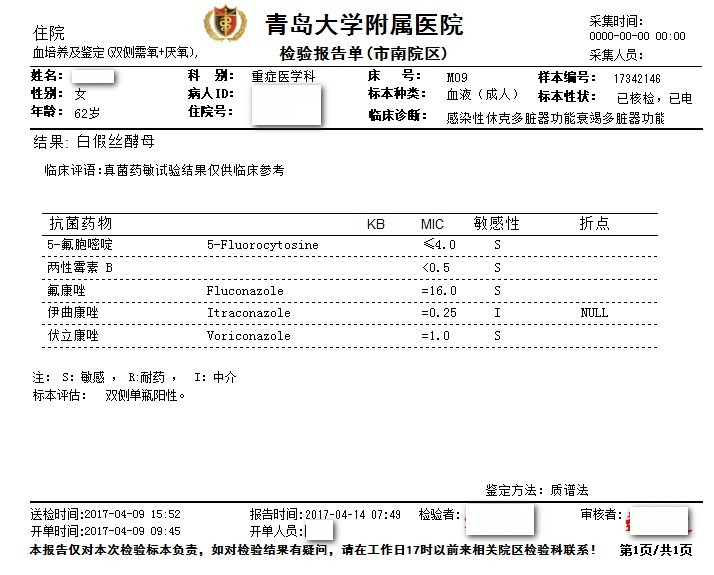

Supplement: Supplemental Information 4 [file peerj-14-20832-s004.zip › Supplement 4/125╛╧.jpg]

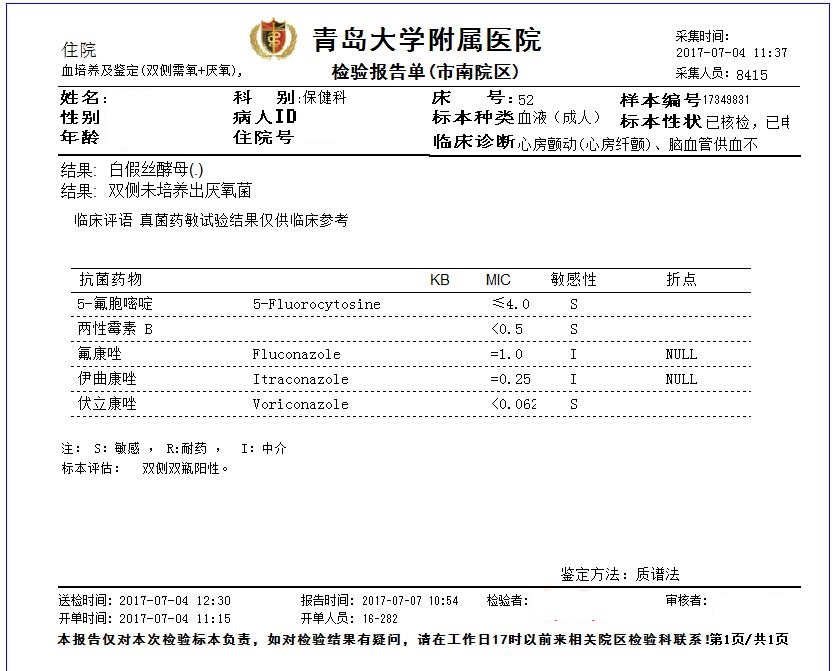

Supplement: Supplemental Information 4 [file peerj-14-20832-s004.zip › Supplement 4/126═⌡.jpg]

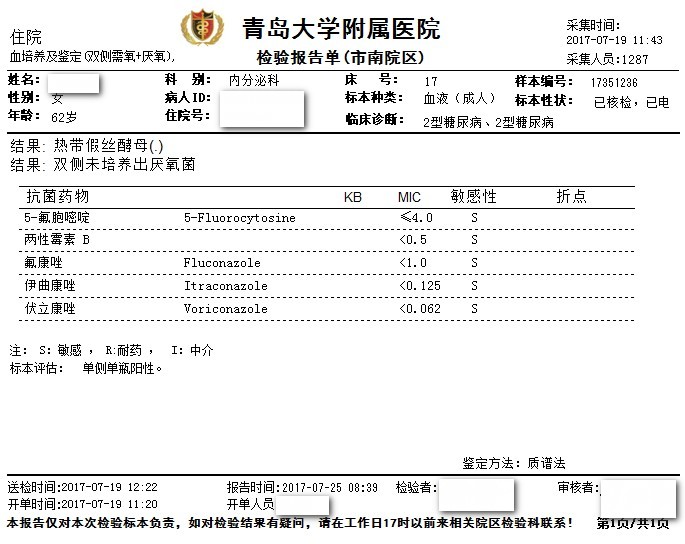

Supplement: Supplemental Information 4 [file peerj-14-20832-s004.zip › Supplement 4/127╨∞.jpg]

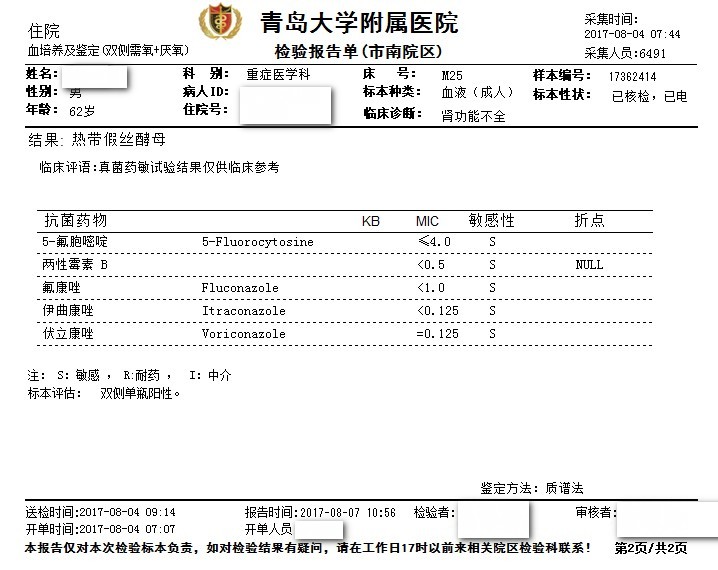

Supplement: Supplemental Information 4 [file peerj-14-20832-s004.zip › Supplement 4/128═⌡.jpg]

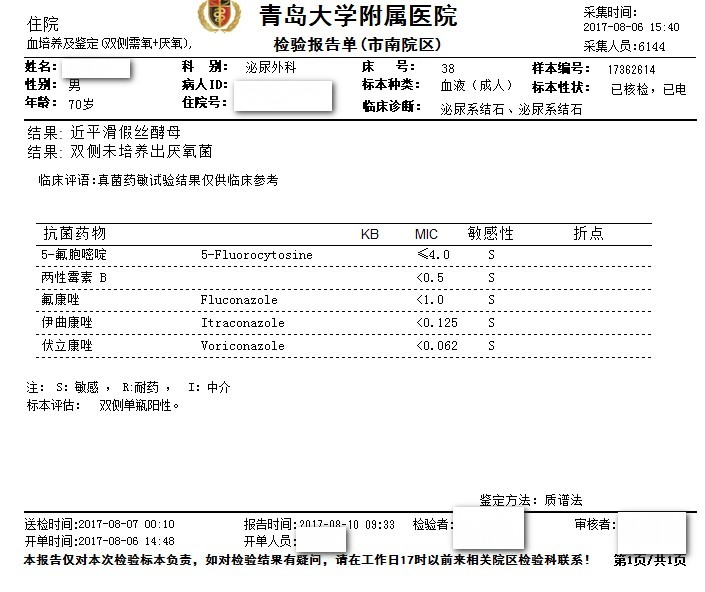

Supplement: Supplemental Information 4 [file peerj-14-20832-s004.zip › Supplement 4/129╤ε.jpg]

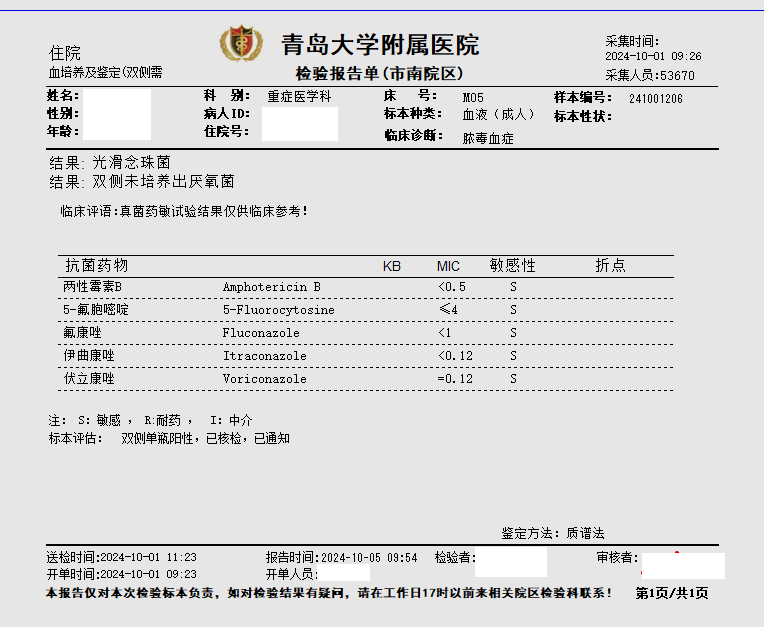

Supplement: Supplemental Information 4 [file peerj-14-20832-s004.zip › Supplement 4/13.png]

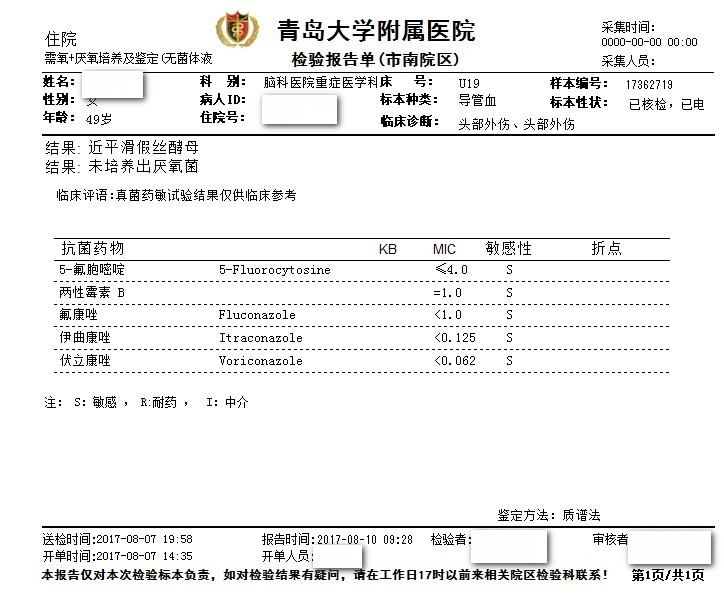

Supplement: Supplemental Information 4 [file peerj-14-20832-s004.zip › Supplement 4/130╜¬.jpg]

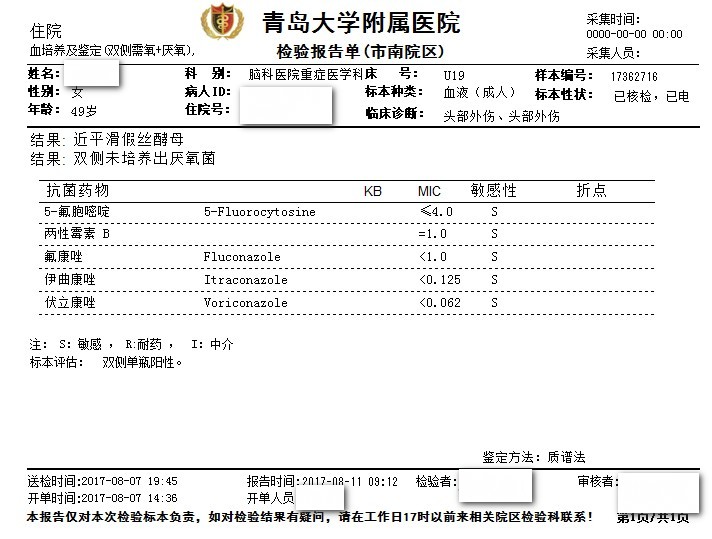

Supplement: Supplemental Information 4 [file peerj-14-20832-s004.zip › Supplement 4/131╜¬.jpg]

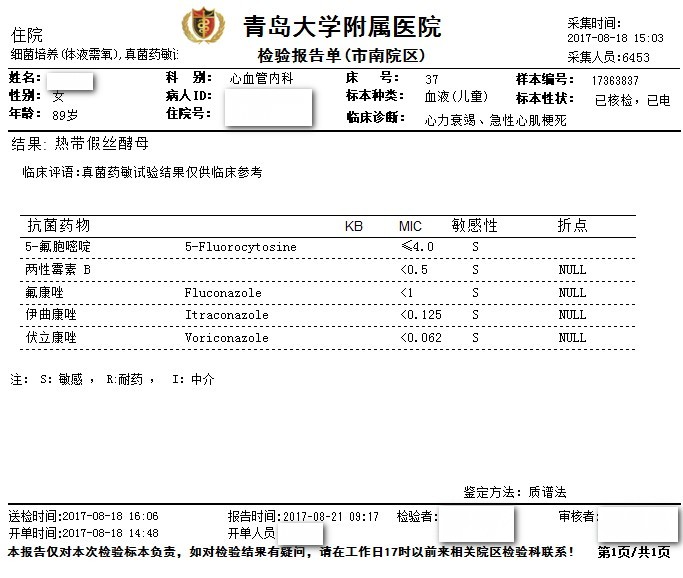

Supplement: Supplemental Information 4 [file peerj-14-20832-s004.zip › Supplement 4/132┴⌡.jpg]

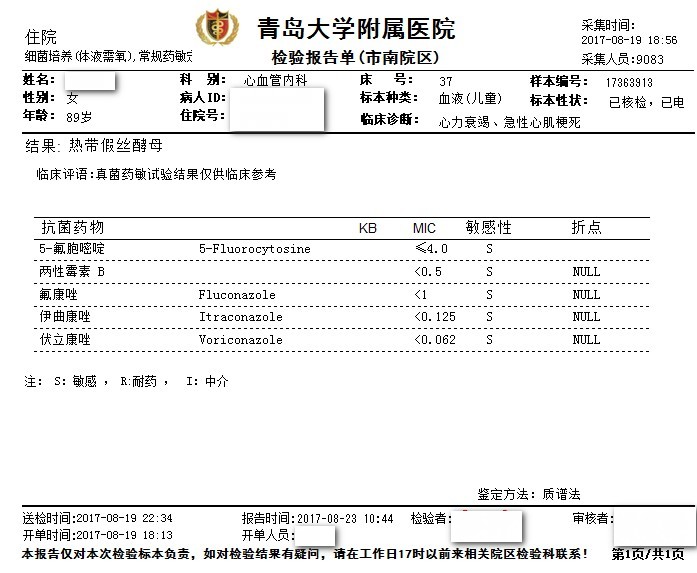

Supplement: Supplemental Information 4 [file peerj-14-20832-s004.zip › Supplement 4/133┴⌡.jpg]

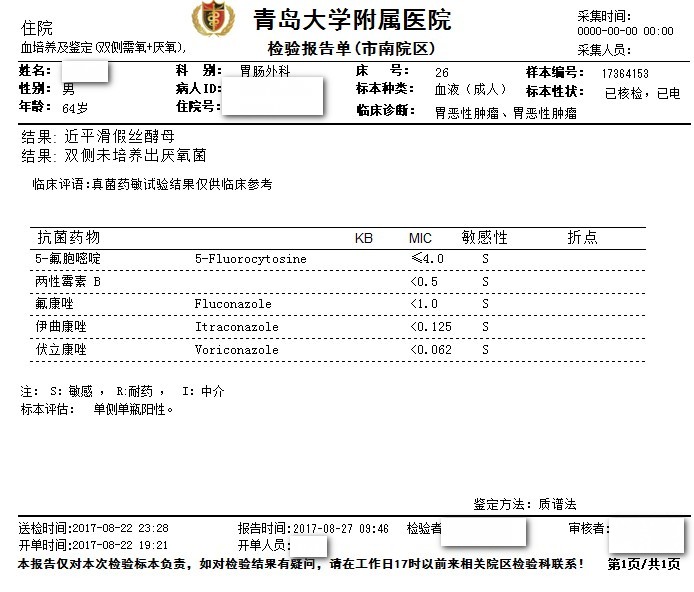

Supplement: Supplemental Information 4 [file peerj-14-20832-s004.zip › Supplement 4/134╣∙.jpg]

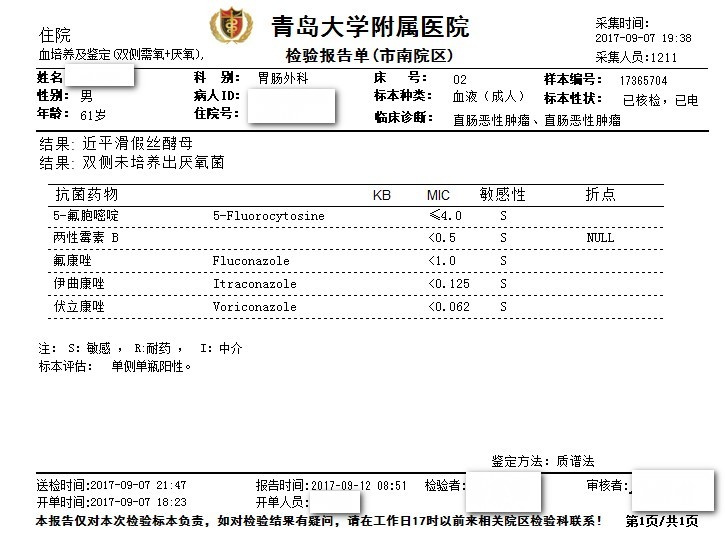

Supplement: Supplemental Information 4 [file peerj-14-20832-s004.zip › Supplement 4/135╬║.jpg]

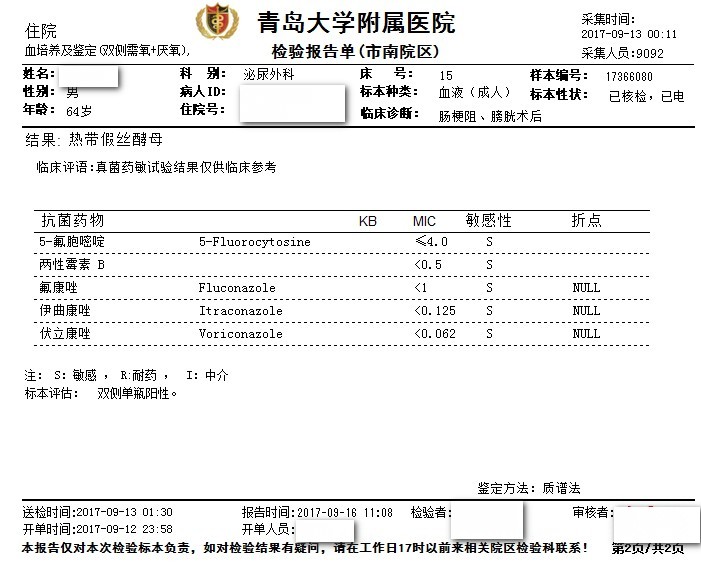

Supplement: Supplemental Information 4 [file peerj-14-20832-s004.zip › Supplement 4/136└ε.jpg]

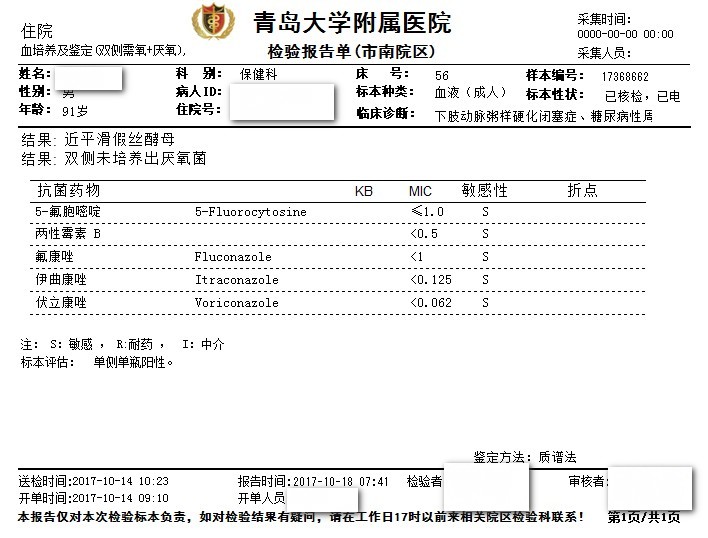

Supplement: Supplemental Information 4 [file peerj-14-20832-s004.zip › Supplement 4/137╥n.jpg]

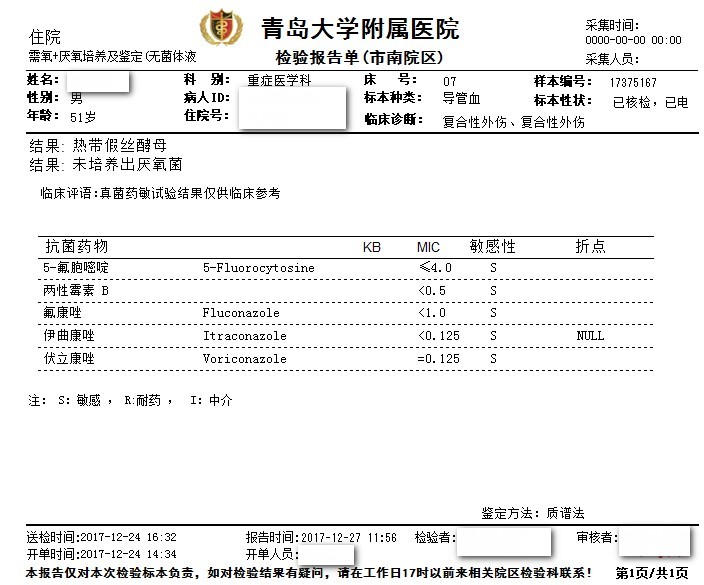

Supplement: Supplemental Information 4 [file peerj-14-20832-s004.zip › Supplement 4/138╠ß.jpg]

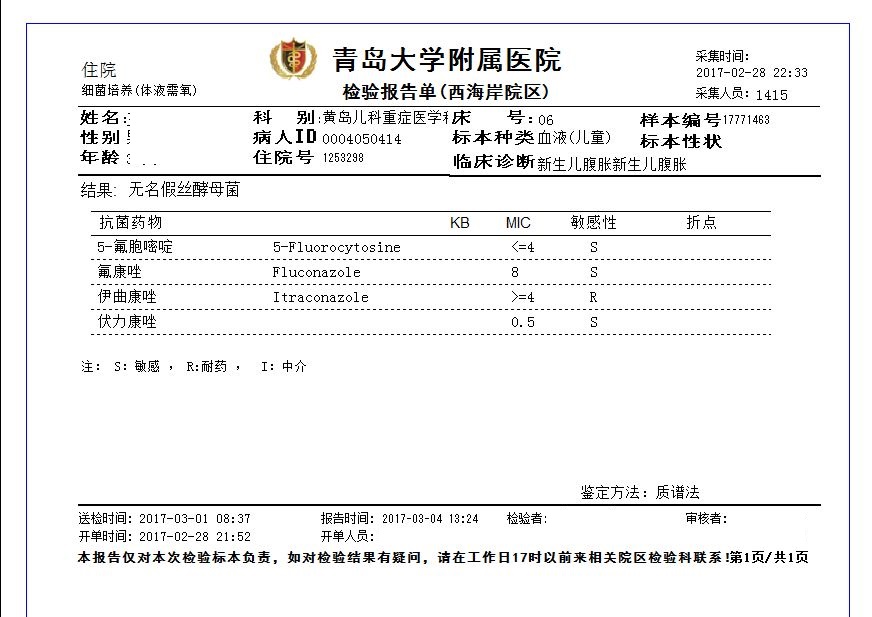

Supplement: Supplemental Information 4 [file peerj-14-20832-s004.zip › Supplement 4/139.JPG]

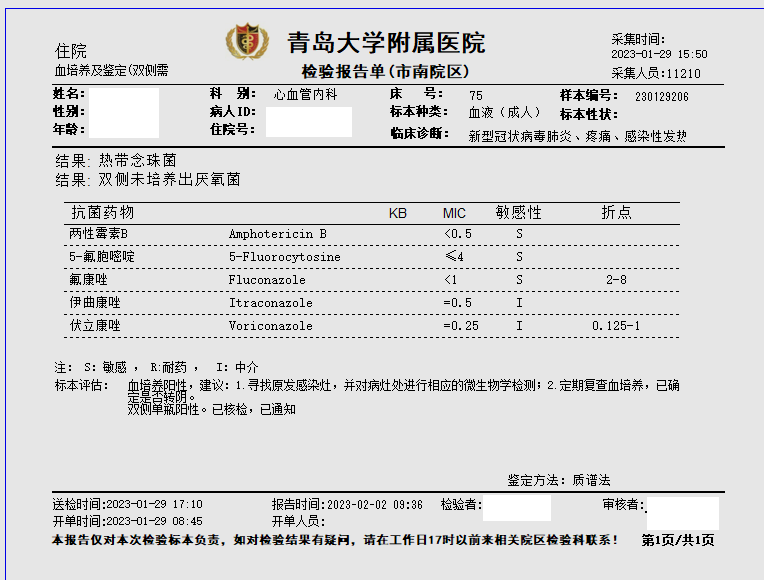

Supplement: Supplemental Information 4 [file peerj-14-20832-s004.zip › Supplement 4/14.png]

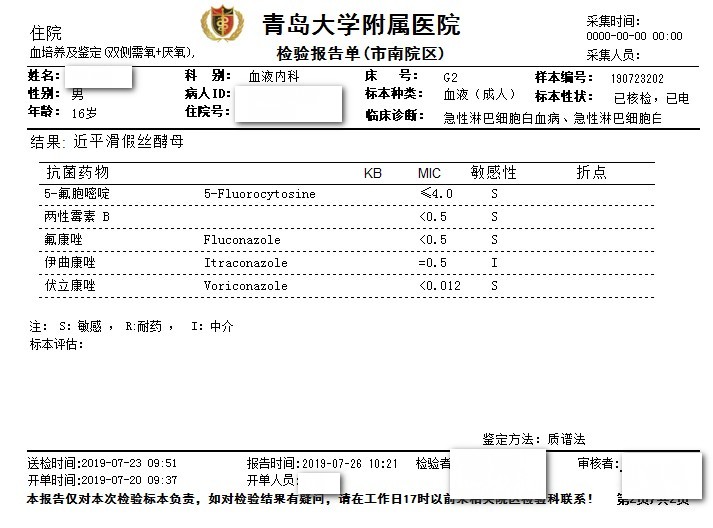

Supplement: Supplemental Information 4 [file peerj-14-20832-s004.zip › Supplement 4/140║1⁄2.jpg]

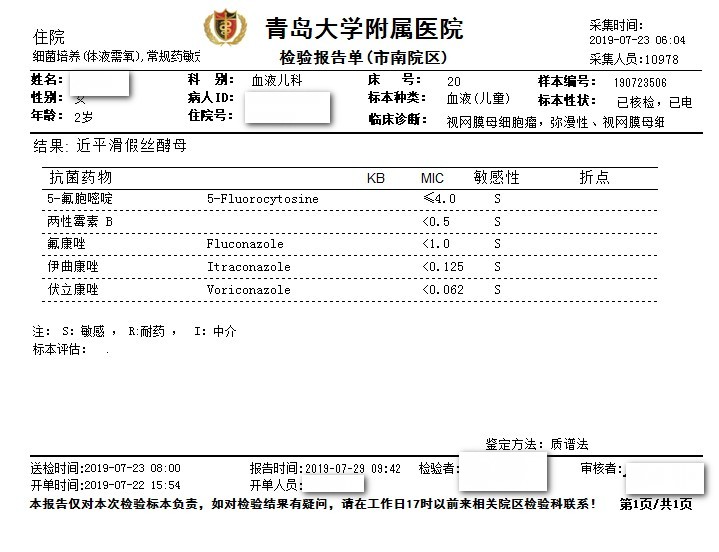

Supplement: Supplemental Information 4 [file peerj-14-20832-s004.zip › Supplement 4/141═≥.jpg]

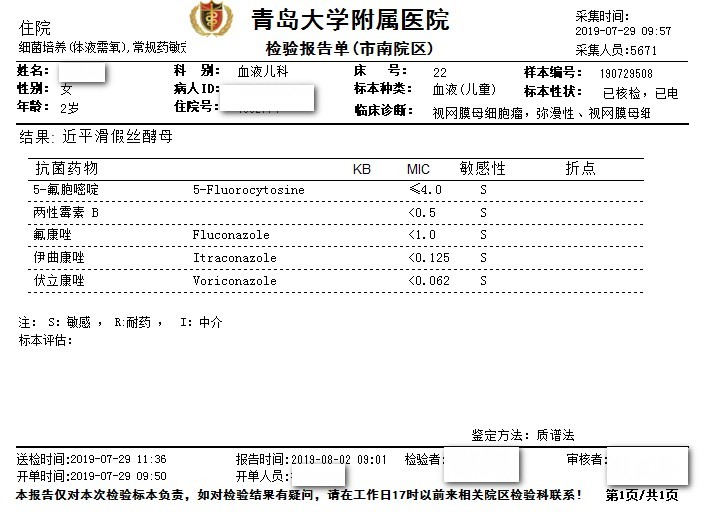

Supplement: Supplemental Information 4 [file peerj-14-20832-s004.zip › Supplement 4/142═≥.jpg]

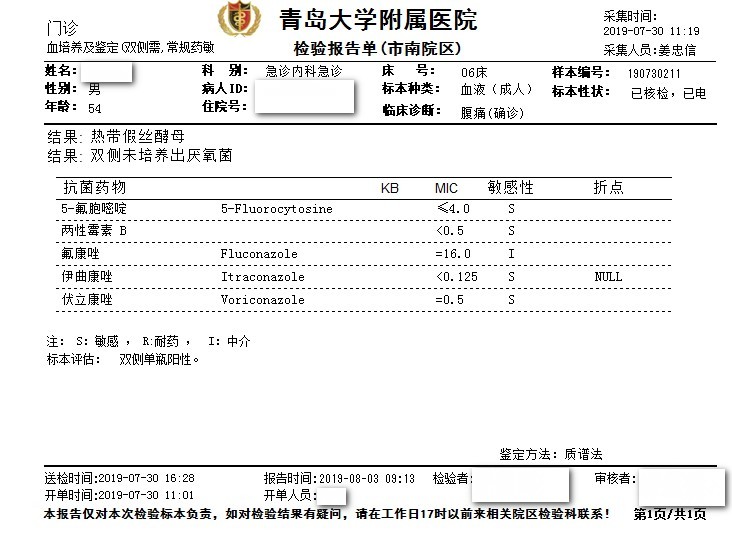

Supplement: Supplemental Information 4 [file peerj-14-20832-s004.zip › Supplement 4/143╦∩.jpg]

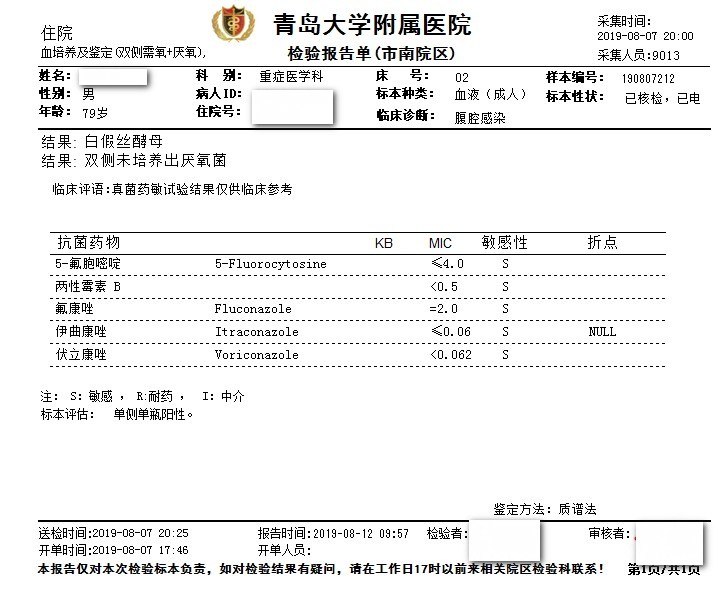

Supplement: Supplemental Information 4 [file peerj-14-20832-s004.zip › Supplement 4/144─▓.jpg]

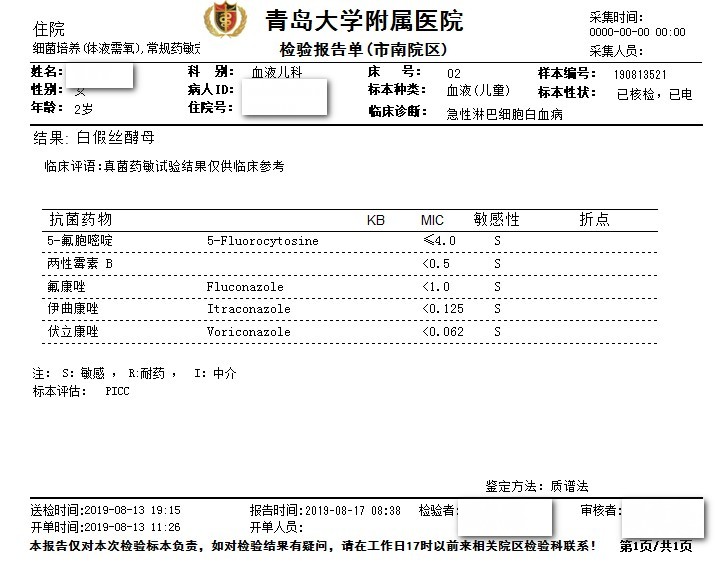

Supplement: Supplemental Information 4 [file peerj-14-20832-s004.zip › Supplement 4/145═⌡.jpg]

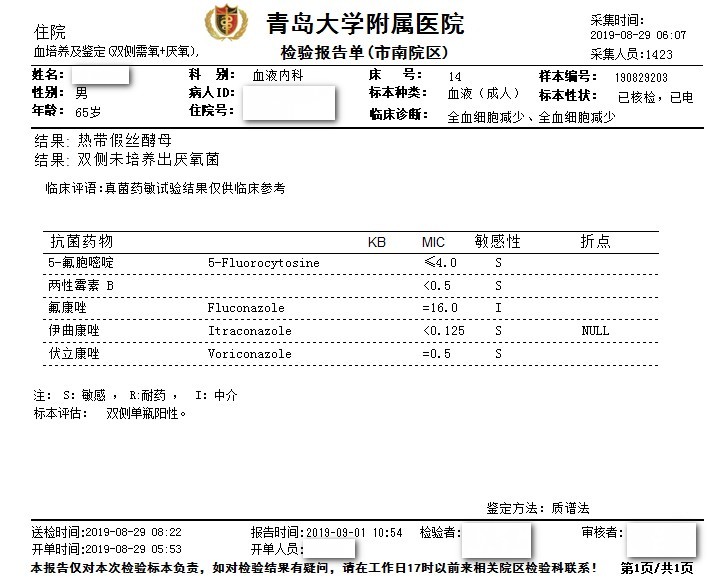

Supplement: Supplemental Information 4 [file peerj-14-20832-s004.zip › Supplement 4/146┬└.jpg]

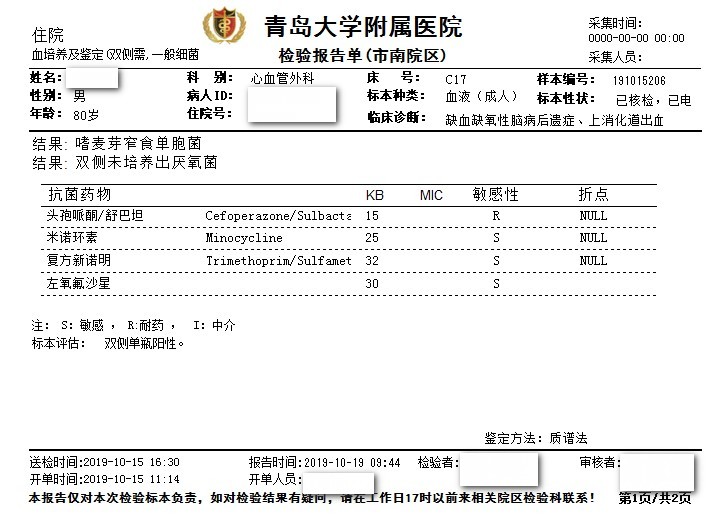

Supplement: Supplemental Information 4 [file peerj-14-20832-s004.zip › Supplement 4/147┴⌡.jpg]

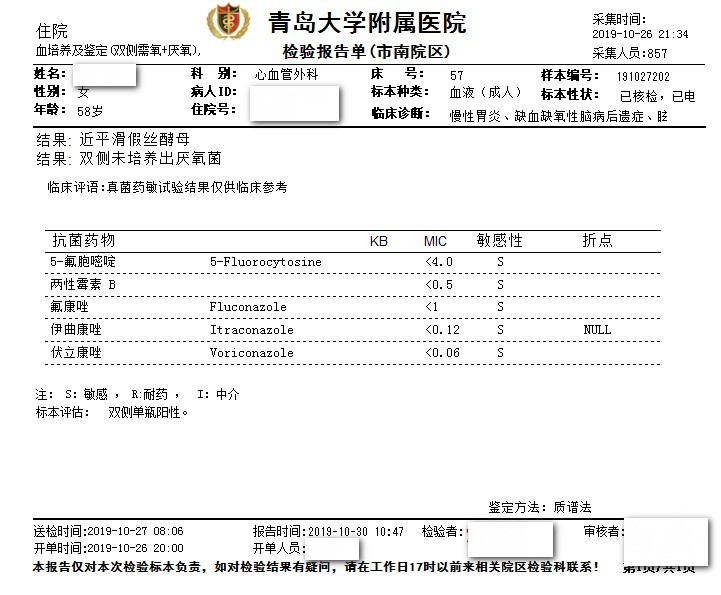

Supplement: Supplemental Information 4 [file peerj-14-20832-s004.zip › Supplement 4/148│┘.jpg]

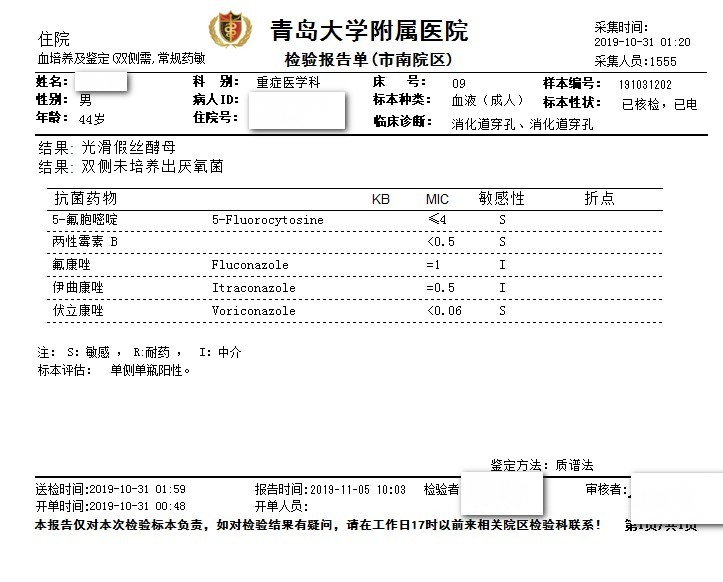

Supplement: Supplemental Information 4 [file peerj-14-20832-s004.zip › Supplement 4/149┤·.jpg]

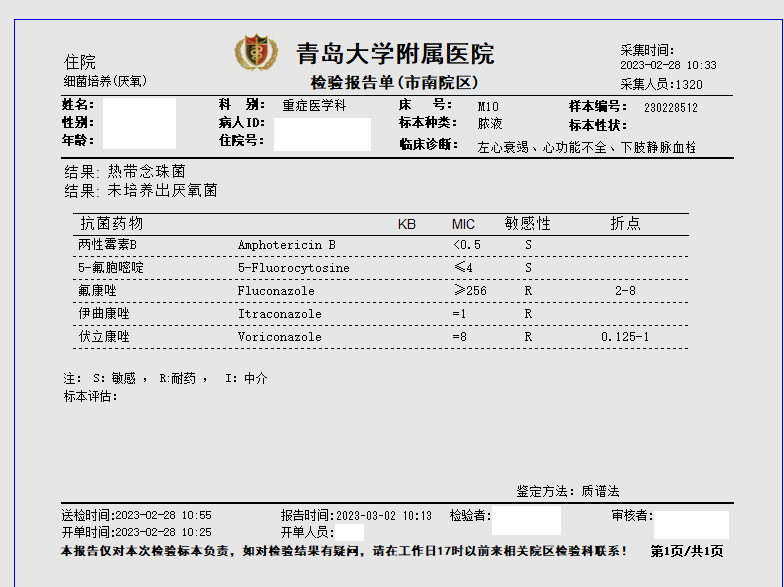

Supplement: Supplemental Information 4 [file peerj-14-20832-s004.zip › Supplement 4/15.png]

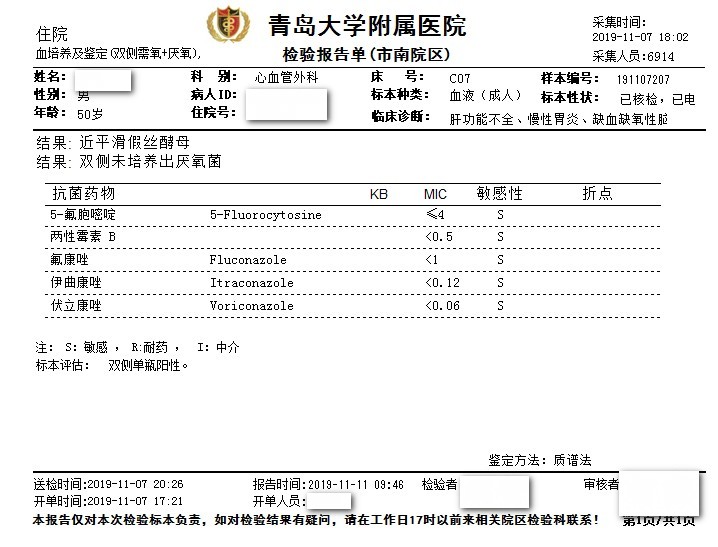

Supplement: Supplemental Information 4 [file peerj-14-20832-s004.zip › Supplement 4/150╦∩.jpg]

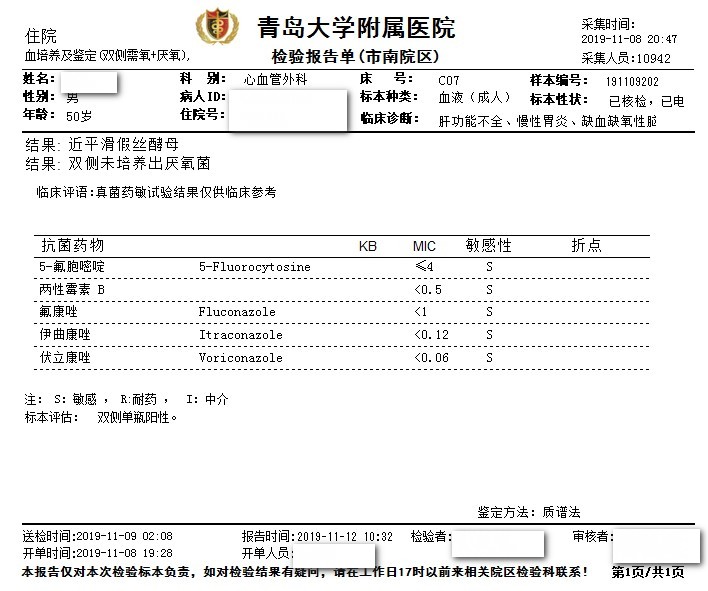

Supplement: Supplemental Information 4 [file peerj-14-20832-s004.zip › Supplement 4/151╦∩jpg.jpg]

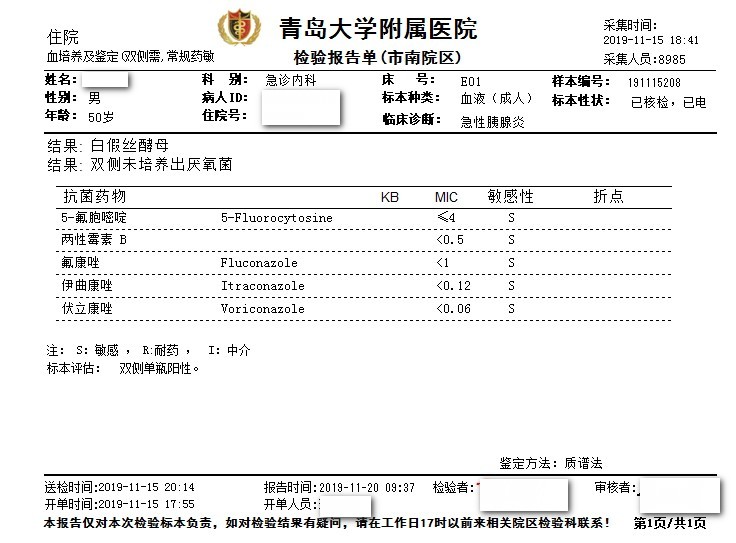

Supplement: Supplemental Information 4 [file peerj-14-20832-s004.zip › Supplement 4/152┴⌡.jpg]

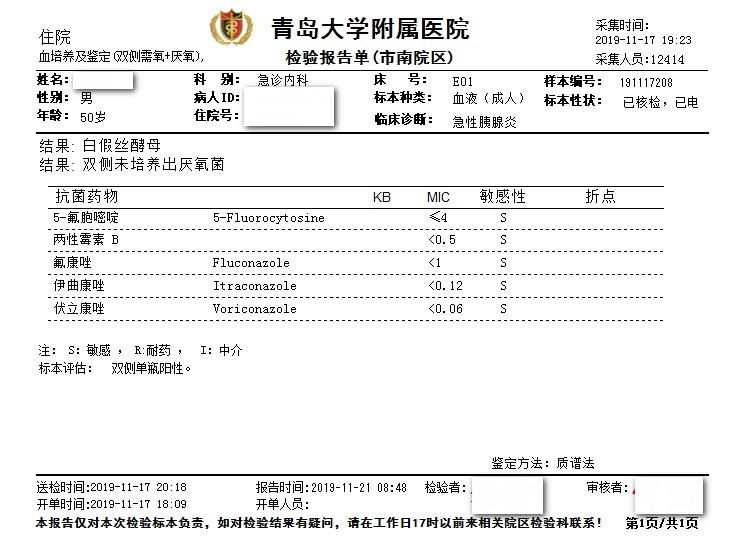

Supplement: Supplemental Information 4 [file peerj-14-20832-s004.zip › Supplement 4/153┴⌡.jpg]

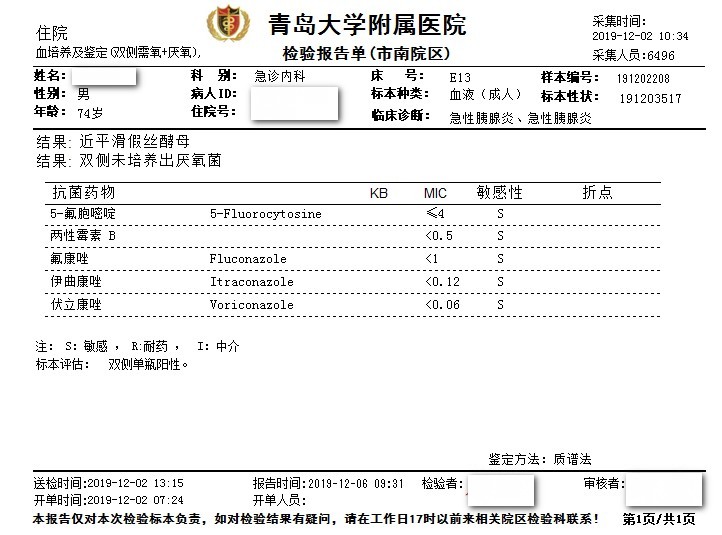

Supplement: Supplemental Information 4 [file peerj-14-20832-s004.zip › Supplement 4/154┤▐.jpg]

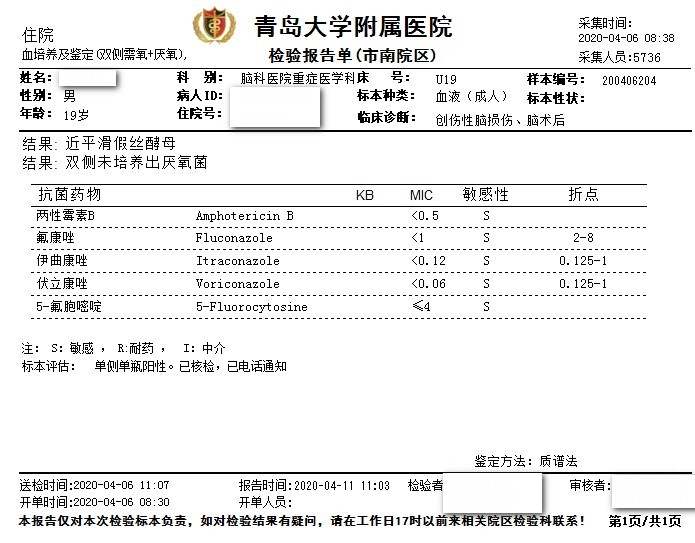

Supplement: Supplemental Information 4 [file peerj-14-20832-s004.zip › Supplement 4/155╖╢.jpg]

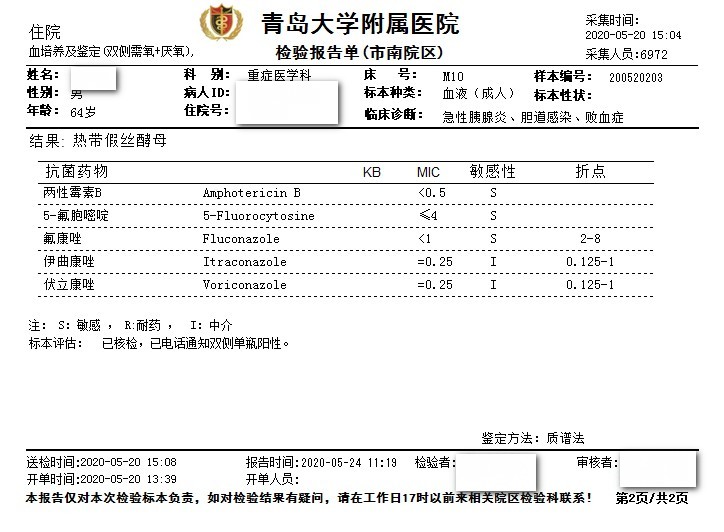

Supplement: Supplemental Information 4 [file peerj-14-20832-s004.zip › Supplement 4/156└ε.jpg]

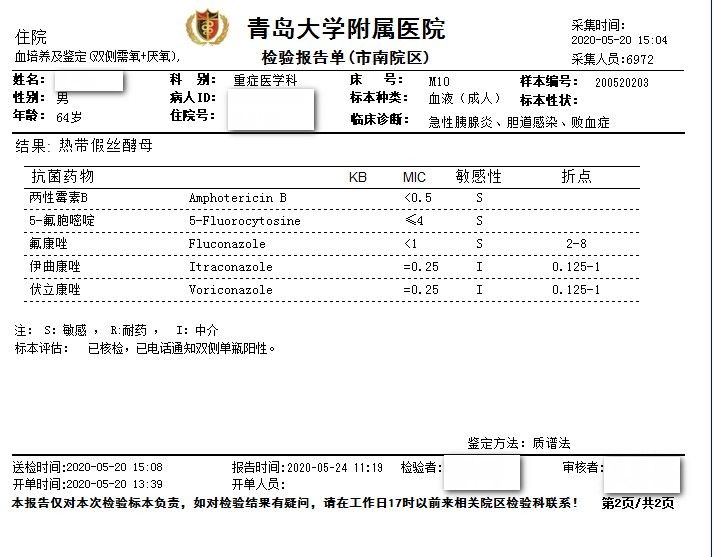

Supplement: Supplemental Information 4 [file peerj-14-20832-s004.zip › Supplement 4/157└ε.jpg]

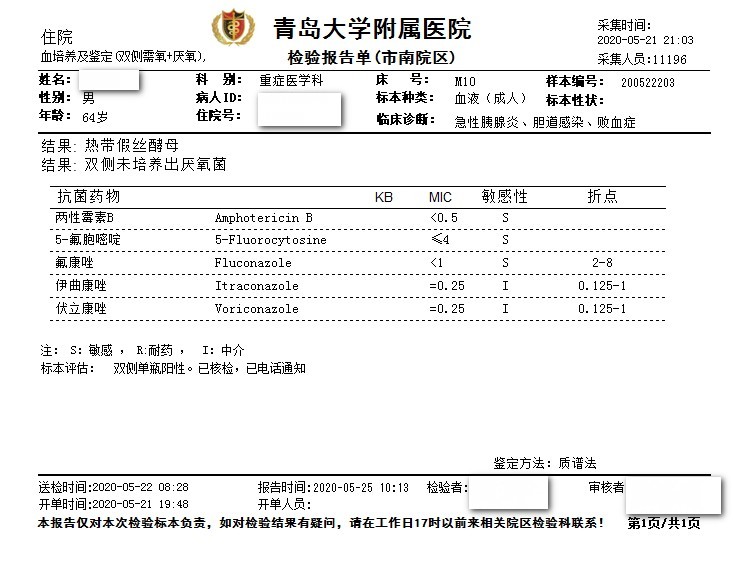

Supplement: Supplemental Information 4 [file peerj-14-20832-s004.zip › Supplement 4/158└ε.jpg]

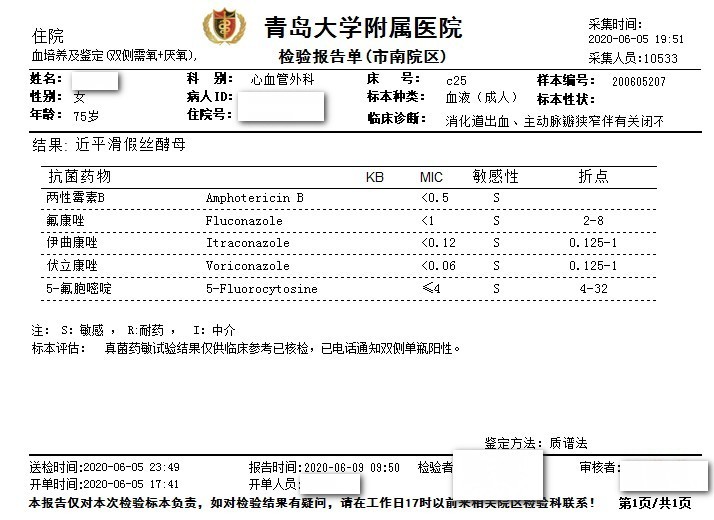

Supplement: Supplemental Information 4 [file peerj-14-20832-s004.zip › Supplement 4/159╔█.jpg]

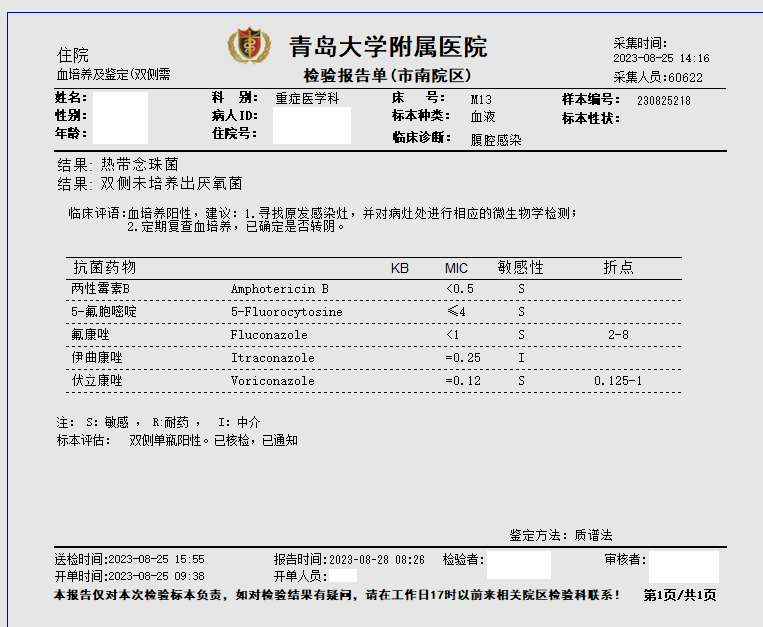

Supplement: Supplemental Information 4 [file peerj-14-20832-s004.zip › Supplement 4/16.png]

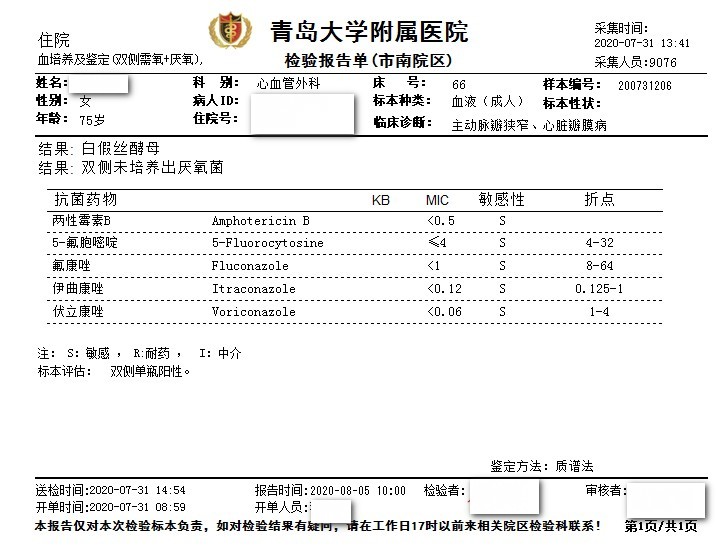

Supplement: Supplemental Information 4 [file peerj-14-20832-s004.zip › Supplement 4/160╦∩.jpg]

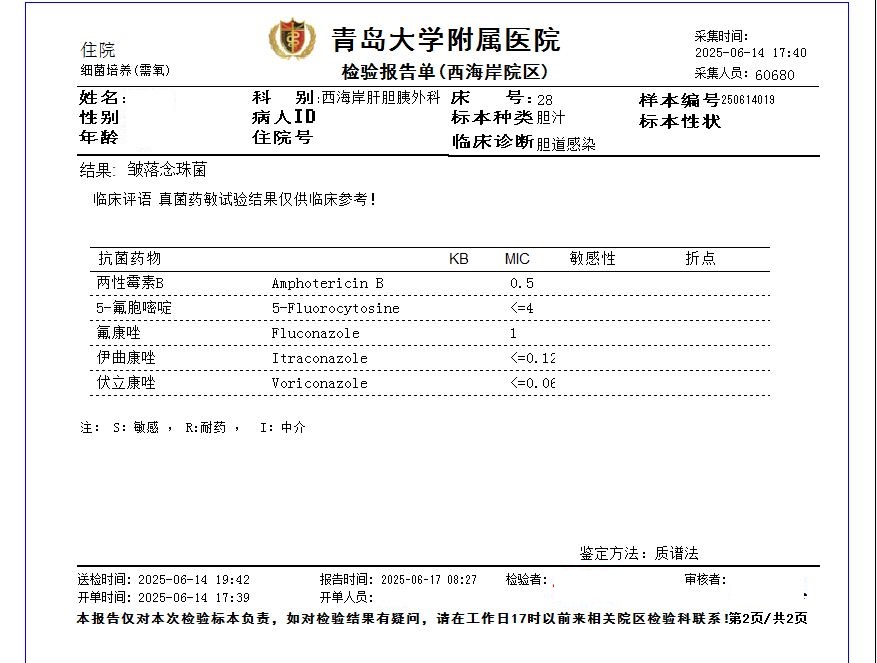

Supplement: Supplemental Information 4 [file peerj-14-20832-s004.zip › Supplement 4/161.└ε.JPG]

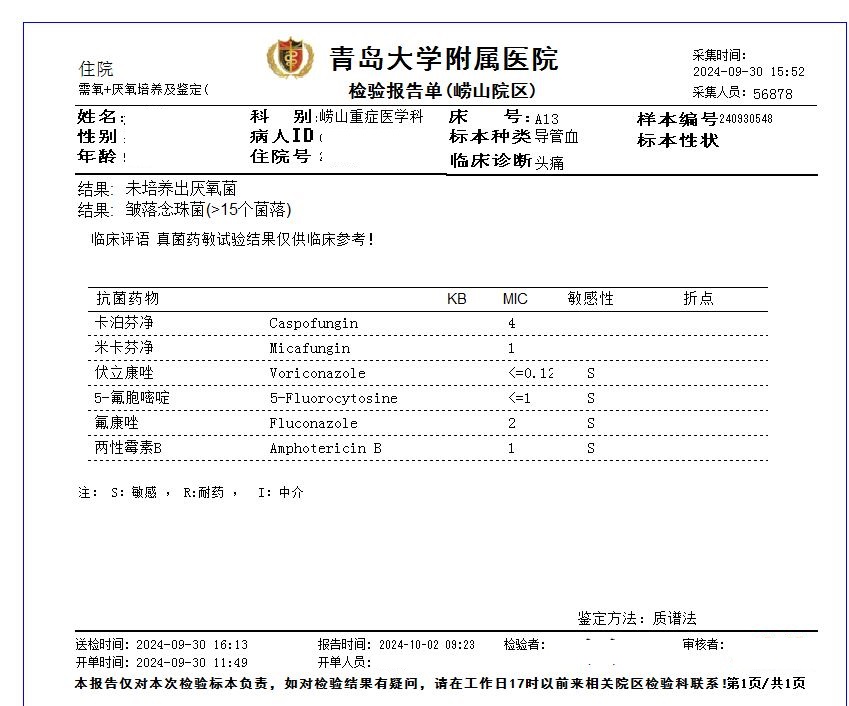

Supplement: Supplemental Information 4 [file peerj-14-20832-s004.zip › Supplement 4/162.└ε.JPG]

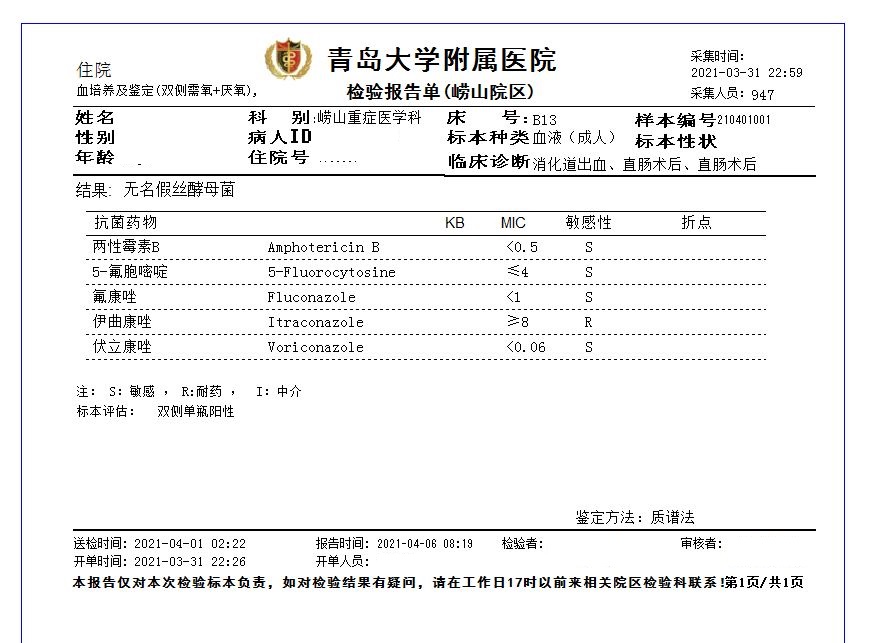

Supplement: Supplemental Information 4 [file peerj-14-20832-s004.zip › Supplement 4/163└ε.JPG]

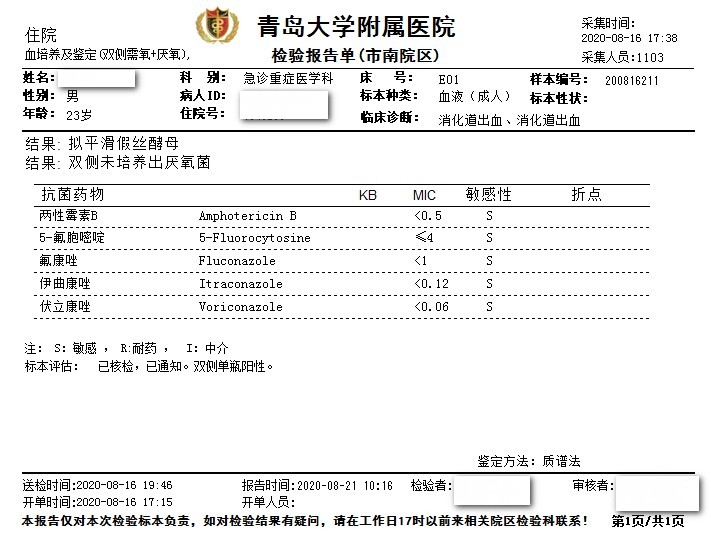

Supplement: Supplemental Information 4 [file peerj-14-20832-s004.zip › Supplement 4/164δ°.jpg]

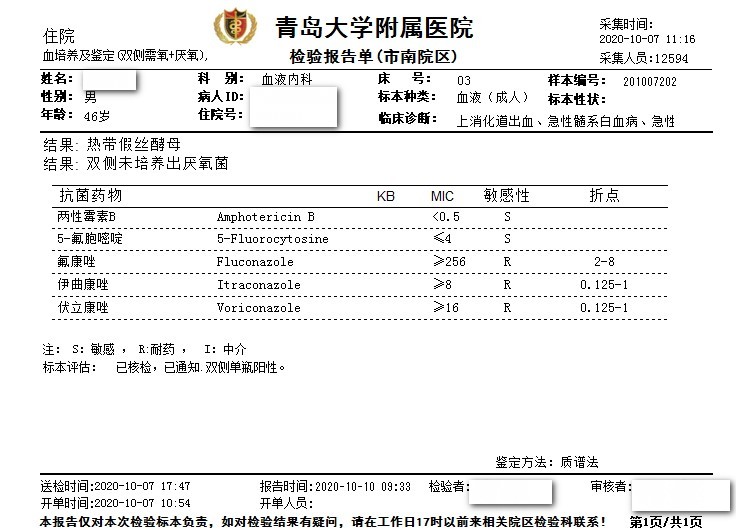

Supplement: Supplemental Information 4 [file peerj-14-20832-s004.zip › Supplement 4/165┤▐.jpg]

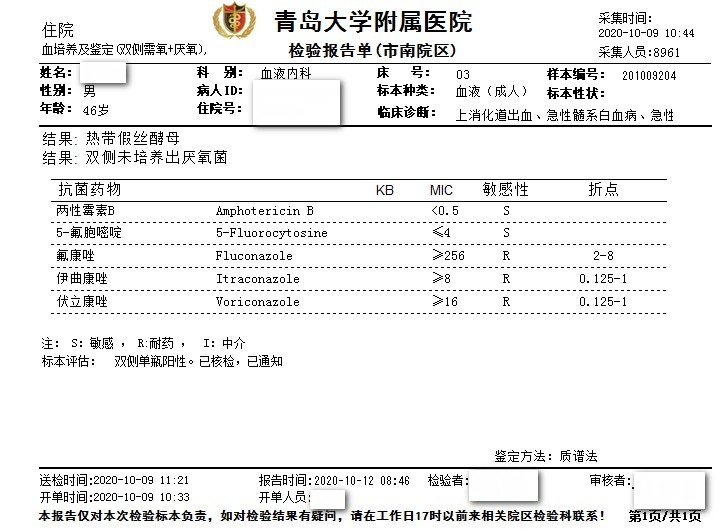

Supplement: Supplemental Information 4 [file peerj-14-20832-s004.zip › Supplement 4/166┤▐.jpg]

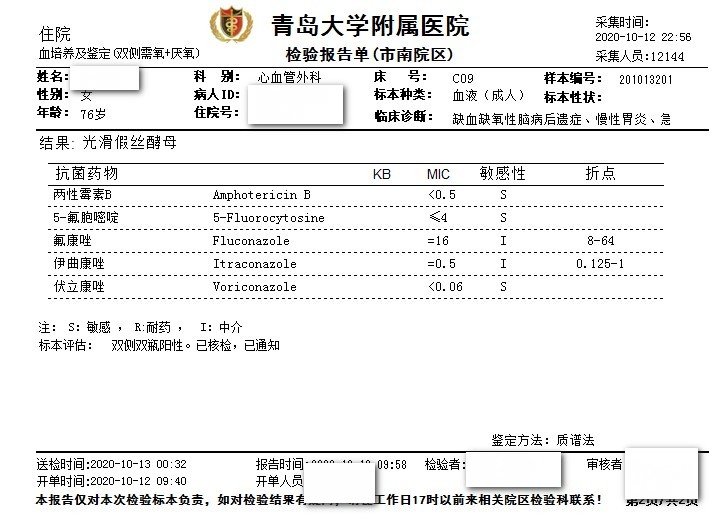

Supplement: Supplemental Information 4 [file peerj-14-20832-s004.zip › Supplement 4/167╓╙.jpg]

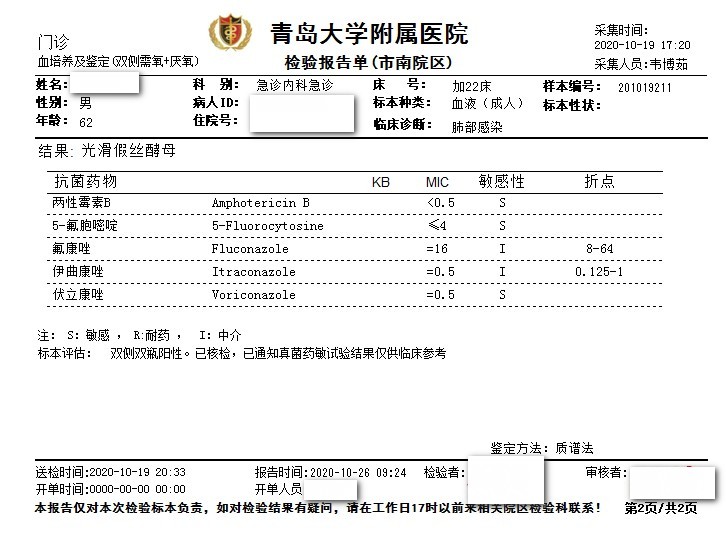

Supplement: Supplemental Information 4 [file peerj-14-20832-s004.zip › Supplement 4/168╦∩.jpg]

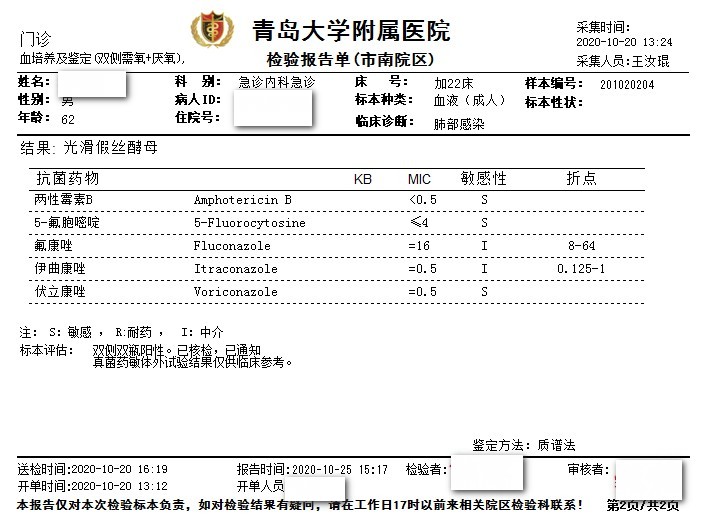

Supplement: Supplemental Information 4 [file peerj-14-20832-s004.zip › Supplement 4/169╦∩.jpg]

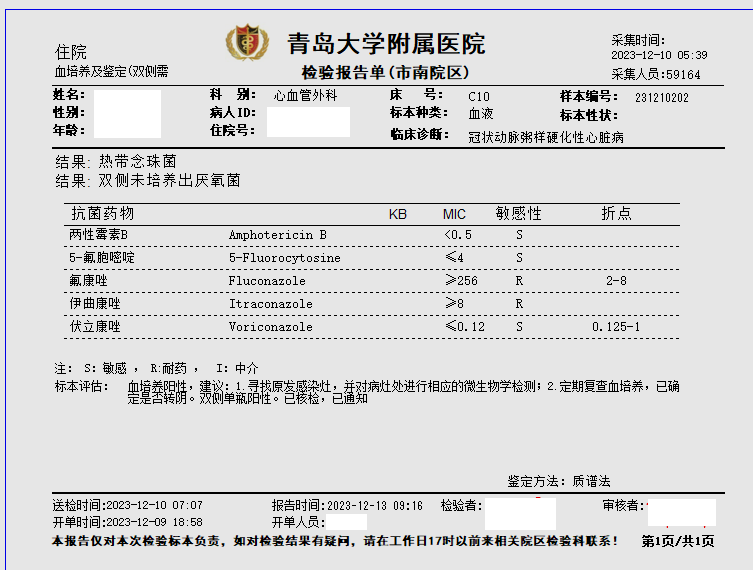

Supplement: Supplemental Information 4 [file peerj-14-20832-s004.zip › Supplement 4/17.png]

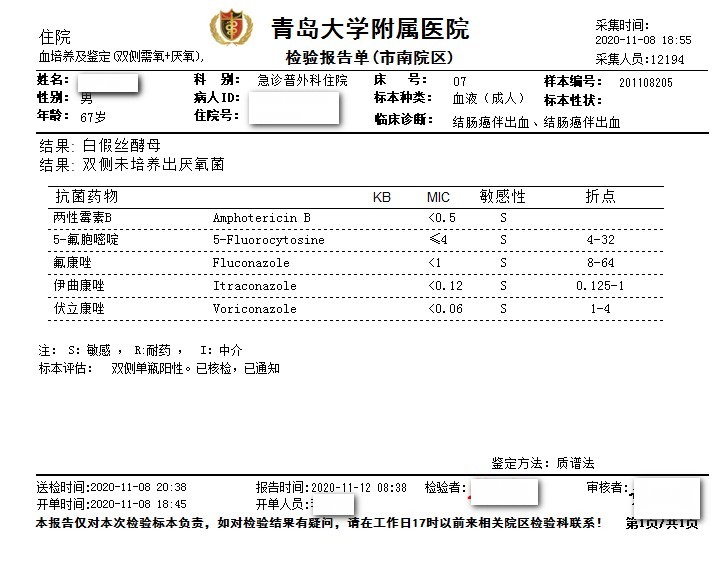

Supplement: Supplemental Information 4 [file peerj-14-20832-s004.zip › Supplement 4/170═≥.jpg]

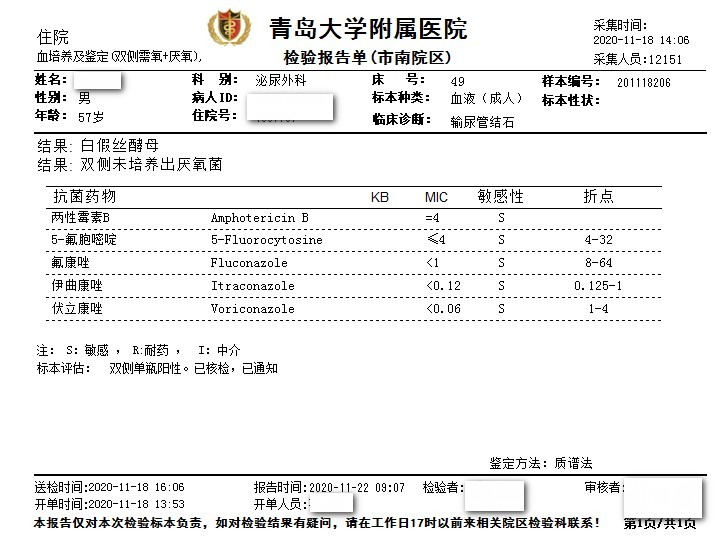

Supplement: Supplemental Information 4 [file peerj-14-20832-s004.zip › Supplement 4/171╨∞.jpg]

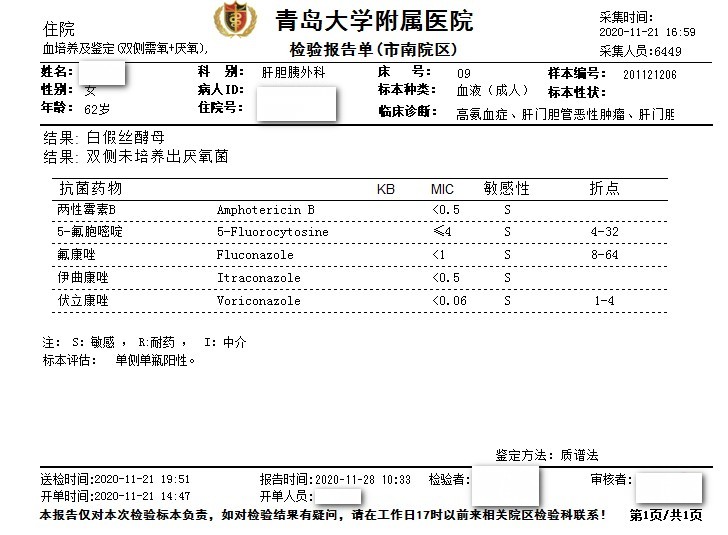

Supplement: Supplemental Information 4 [file peerj-14-20832-s004.zip › Supplement 4/172.jpg]

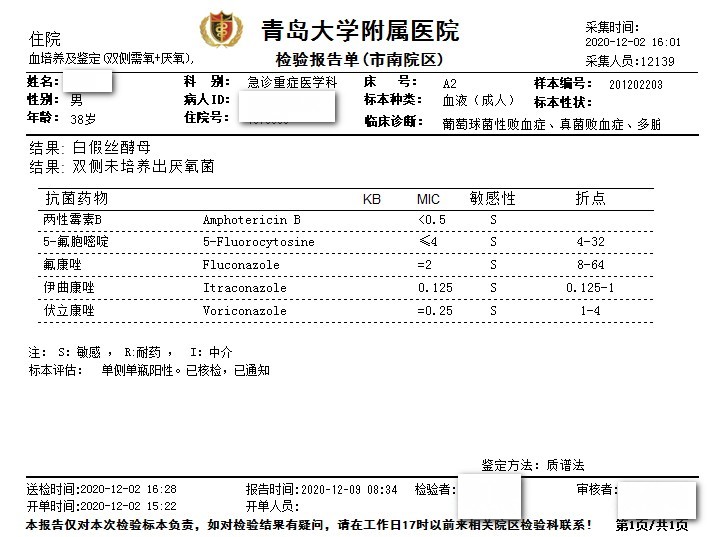

Supplement: Supplemental Information 4 [file peerj-14-20832-s004.zip › Supplement 4/173╒┼.jpg]

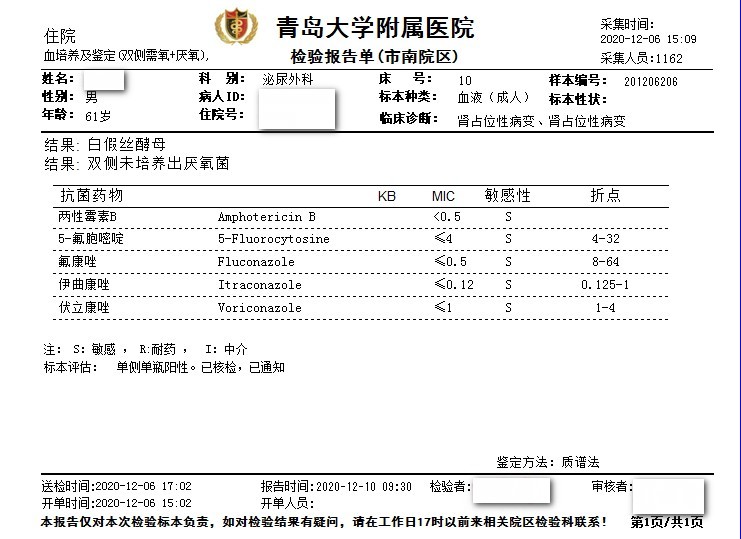

Supplement: Supplemental Information 4 [file peerj-14-20832-s004.zip › Supplement 4/174╕▀.jpg]

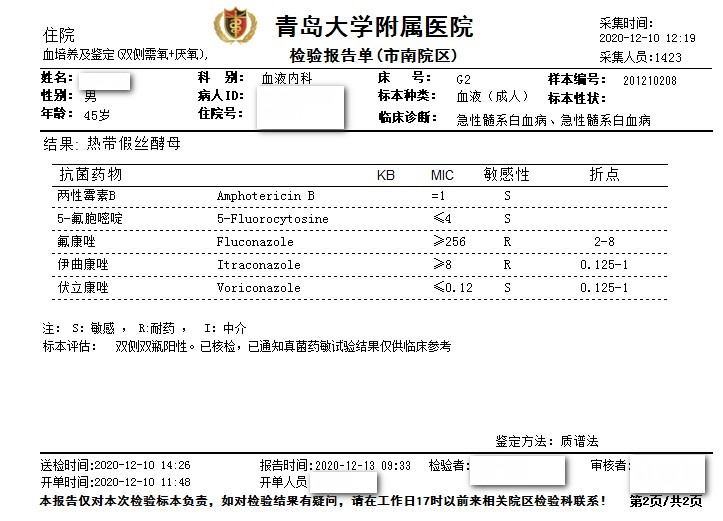

Supplement: Supplemental Information 4 [file peerj-14-20832-s004.zip › Supplement 4/175┴⌡.jpg]

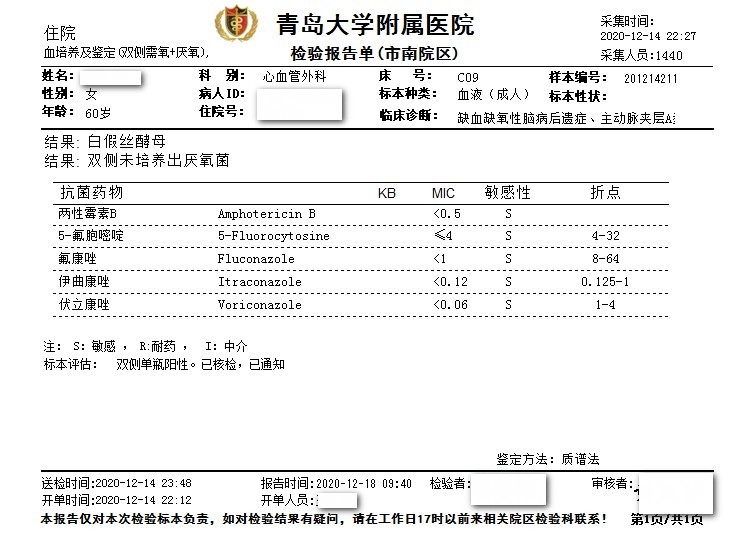

Supplement: Supplemental Information 4 [file peerj-14-20832-s004.zip › Supplement 4/176╜Γ.jpg]

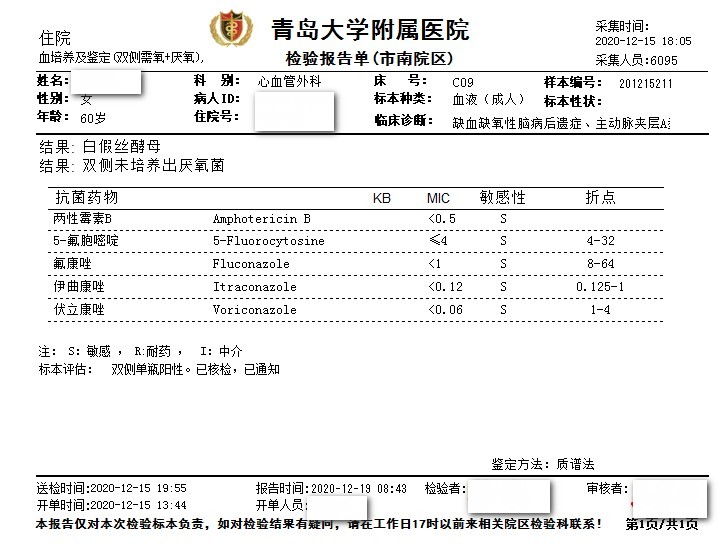

Supplement: Supplemental Information 4 [file peerj-14-20832-s004.zip › Supplement 4/177╜Γ.jpg]

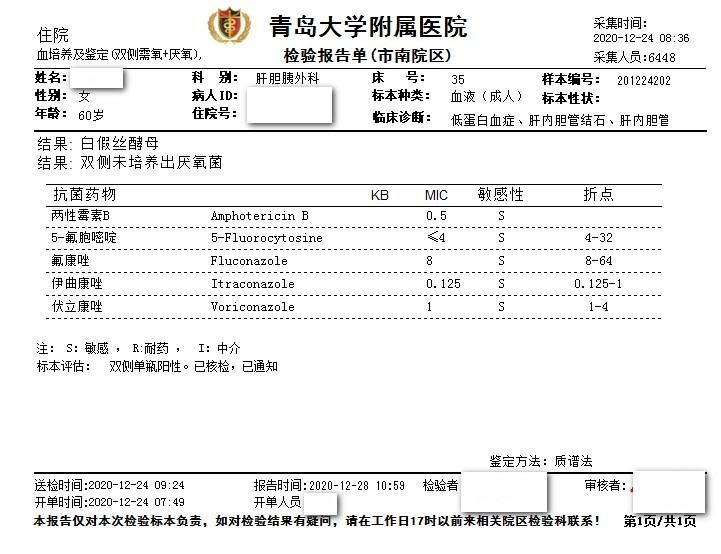

Supplement: Supplemental Information 4 [file peerj-14-20832-s004.zip › Supplement 4/178╤╒.jpg]

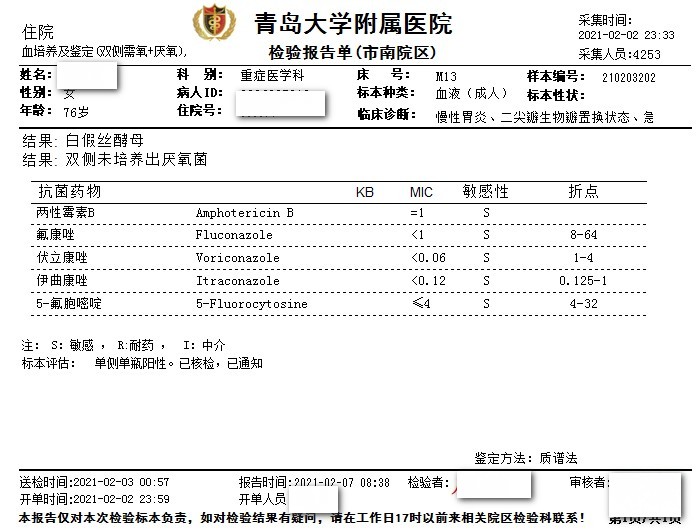

Supplement: Supplemental Information 4 [file peerj-14-20832-s004.zip › Supplement 4/179╙┌.jpg]

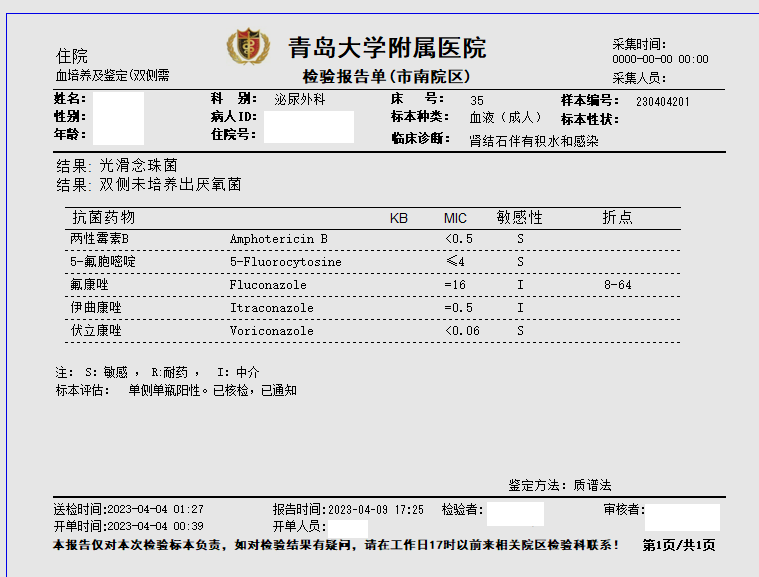

Supplement: Supplemental Information 4 [file peerj-14-20832-s004.zip › Supplement 4/18.png]

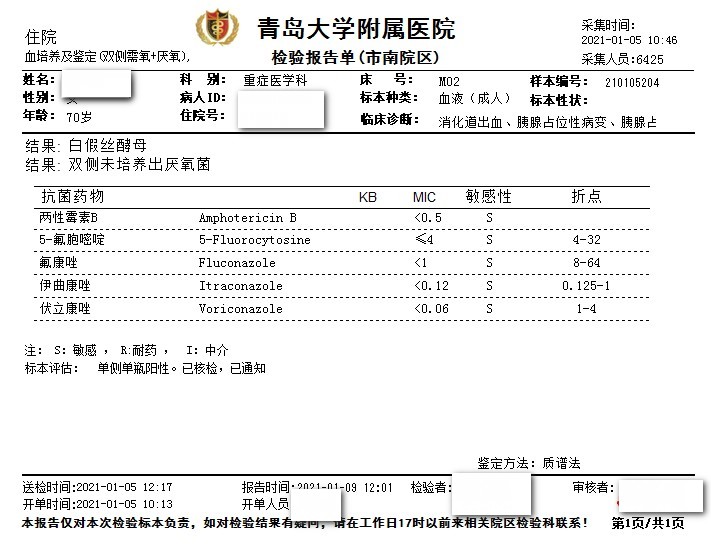

Supplement: Supplemental Information 4 [file peerj-14-20832-s004.zip › Supplement 4/180╤ε.jpg]

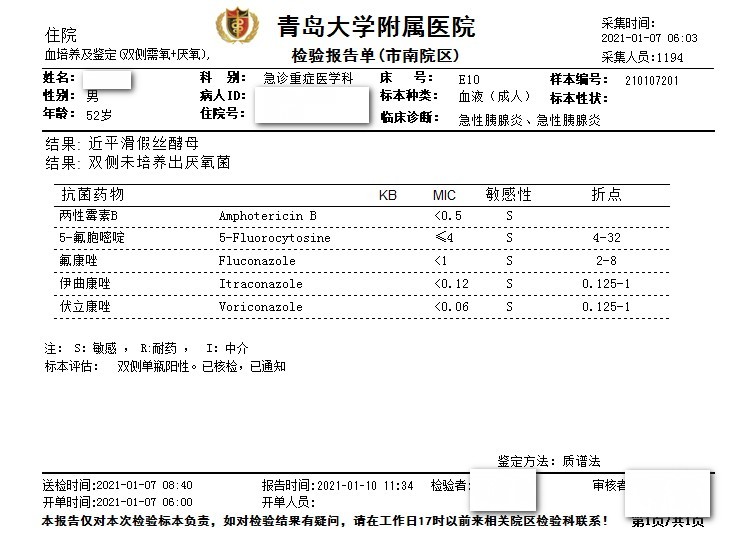

Supplement: Supplemental Information 4 [file peerj-14-20832-s004.zip › Supplement 4/181═⌡.jpg]

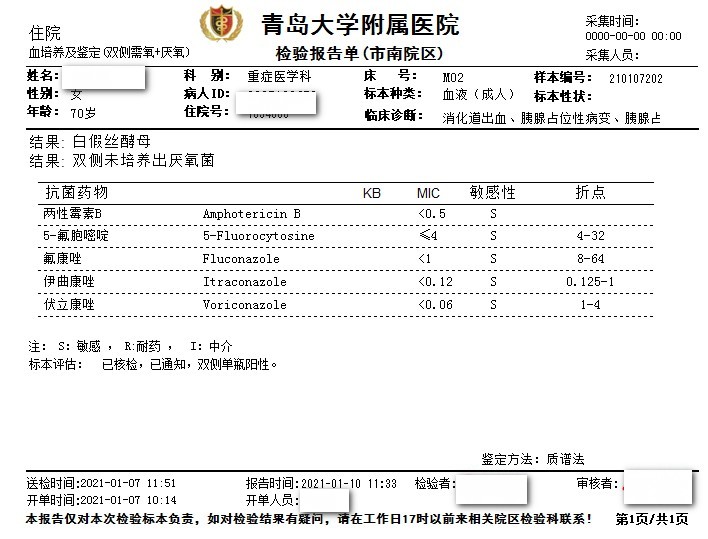

Supplement: Supplemental Information 4 [file peerj-14-20832-s004.zip › Supplement 4/182╤ε.jpg]

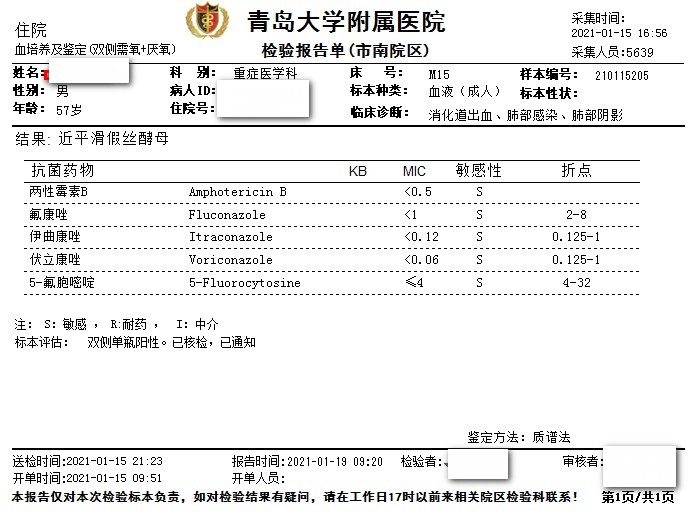

Supplement: Supplemental Information 4 [file peerj-14-20832-s004.zip › Supplement 4/183╘1⁄4.jpg]

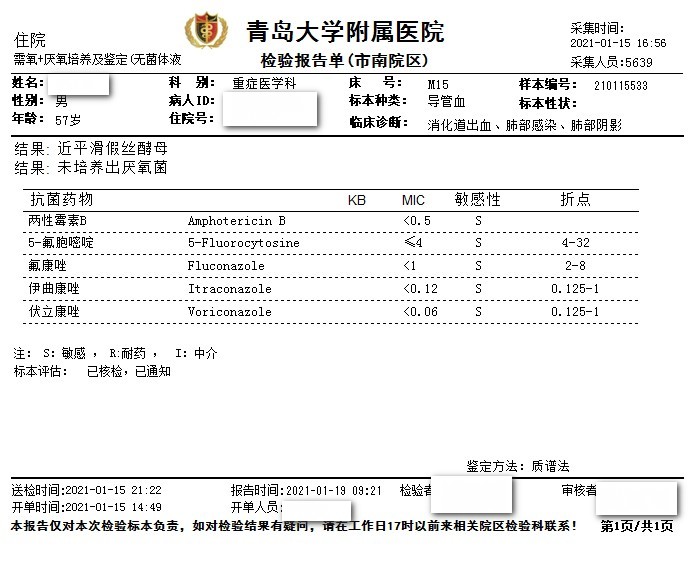

Supplement: Supplemental Information 4 [file peerj-14-20832-s004.zip › Supplement 4/184╘1⁄4.jpg]

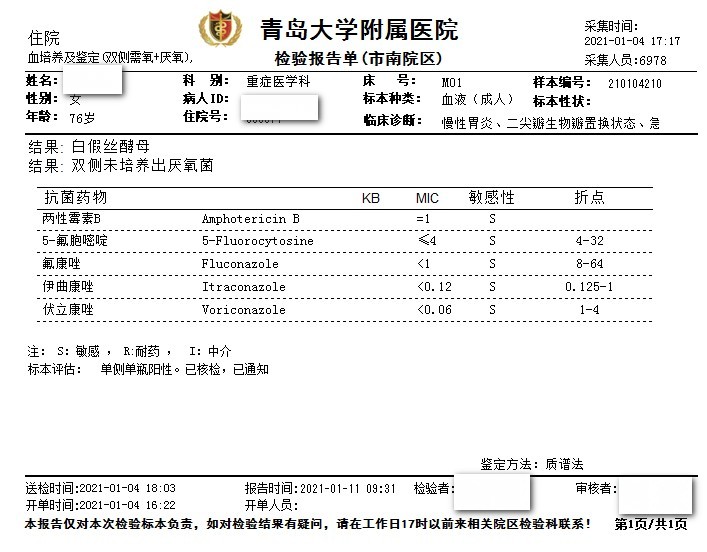

Supplement: Supplemental Information 4 [file peerj-14-20832-s004.zip › Supplement 4/185╙┌.jpg]

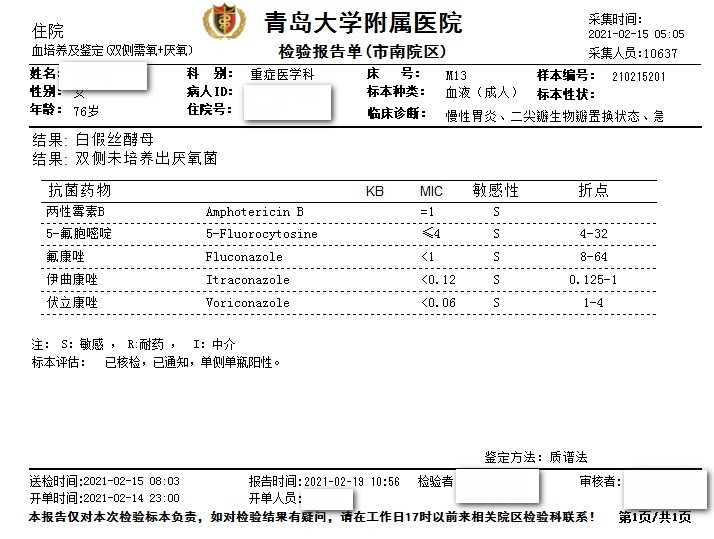

Supplement: Supplemental Information 4 [file peerj-14-20832-s004.zip › Supplement 4/186╙┌.jpg]

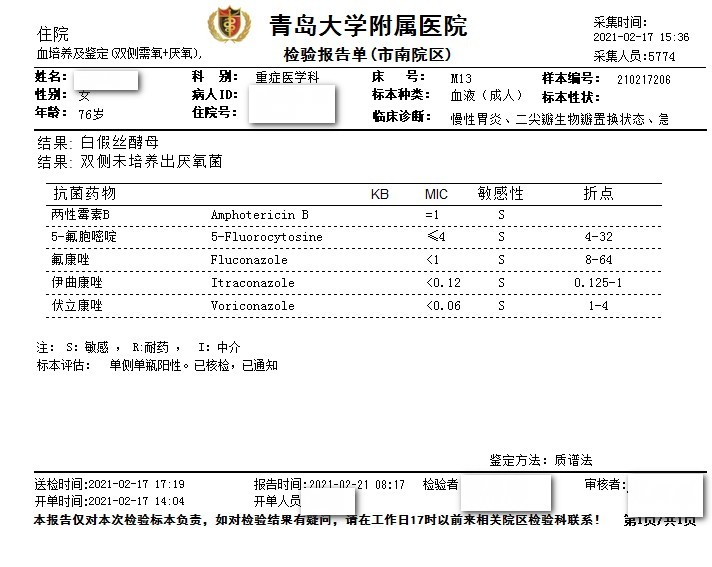

Supplement: Supplemental Information 4 [file peerj-14-20832-s004.zip › Supplement 4/187╙┌.jpg]

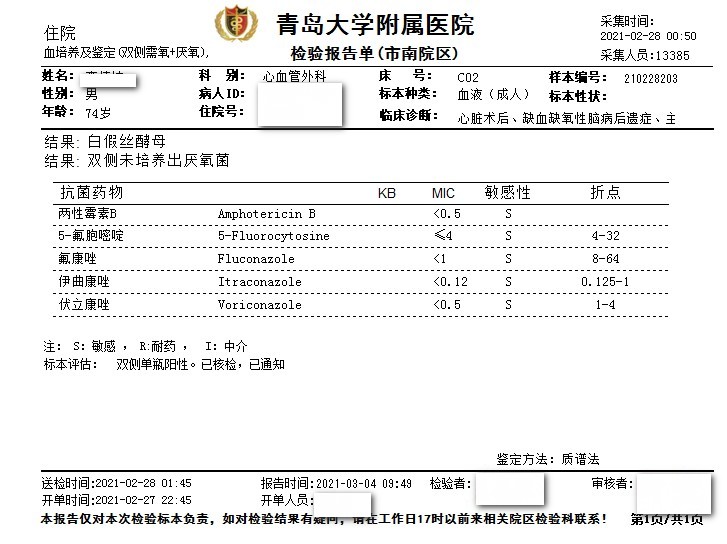

Supplement: Supplemental Information 4 [file peerj-14-20832-s004.zip › Supplement 4/188└ε.jpg]

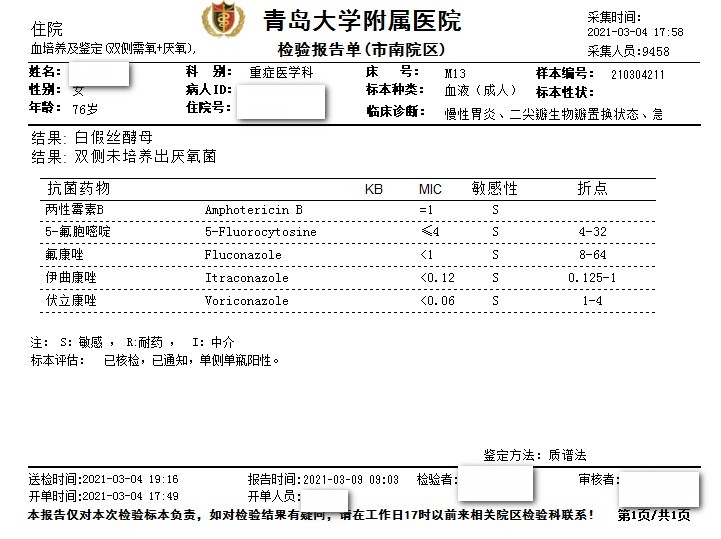

Supplement: Supplemental Information 4 [file peerj-14-20832-s004.zip › Supplement 4/189╙┌.jpg]
