# Supplementary material for: Candida species distribution, antifungal susceptibility and trends causing candidemia: a 10-year observation in eastern China
Source: PeerJ. 2026 Mar 5;14:e20832. doi: 10.7717/peerj.20832 (PMC12967414; doi:10.7717/peerj.20832)
Supplement: Supplemental Information 5 [file peerj-14-20832-s005.docx]

**Name: Gender:** m

**Age:** 64

**Department:** ICU　 **Patient ID:**

**Inpatient No.:**

**Bed No.:**

**Specimen Type:** blood

**Clinical Diagnosis:**

**Sample No.:** 230324207

**Result:** ***Candida parapsilosis***

Antibiotic MIC

Amphotericin B <0.5

Fluconazole <1

Itraconazole 8

Voriconazole <0.06

AST Result Interpretation

WT

S

nWT

S

Breakpoint

2

2-8

0.5

0.12-1

Note: S: Sensitive,R: Resistant, I: Intermediat, nWT: non-Wild Type, WT: Wild Type

Identification Method: MOLDI TOF

Specimen submission time: 2023.3.24 10:47 Report Release Time: 2023.3.28 8:46 Laboratory Technician： Report Reviewer ：

Laboratory test order time: 2023.3.24 9:22 Ordering Physician：

**This report applies solely to the submitted specimen. For inquiries regarding test results, please contact the Laboratory prior to 17:00 on working days.**

**Name: Gender:** **m**

**Age:** 64

**Department:** ICU　 **Patient ID:**

**Inpatient No.:**

**Bed No.:**

**Specimen Type:** blood

**Clinical Diagnosis:**

**Sample No.:** 230619202

**Result:** ***Candida parapsilosis***

Antibiotic MIC

Amphotericin B <0.5

Fluconazole <1

Itraconazole <0.12

Voriconazole <0.06

AST Result Interpretation

WT

S

WT

S

Breakpoint

1

2-8

0.5

0.12-1

Note: S: Sensitive,R: Resistant, I: Intermediat, nWT: non-Wild Type, WT: Wild Type

Identification Method: MOLDI TOF

Specimen submission time: 2023.6.19 10:52

Laboratory test order time: 2023.6.19 9:33

Report Release Time: 2023.6.238:38 Laboratory Technician: Report Reviewer:

Ordering Physician:

**This report applies solely to the submitted specimen. For inquiries regarding test results, please contact the Laboratory prior to 17:00 on working days.**

**Name:**

**Gender:** **m**

**Age:** 59

**Department:**

**Patient ID:**

**Inpatient No.:**

Internal Medicine

**Bed No.:**

**Specimen Type:** blood

**Clinical Diagnosis:**

**Sample No.:** 230828213

**Result:** ***Candida tropicalis***

Antibiotic MIC

Amphotericin B <0.5

Fluconazole 4

Itraconazole 0.5

Voriconazole 0.5

AST Result Interpretation

WT

I

nWT

I

Breakpoint

2

2-8

0.5

0.12-1

Note: S: Sensitive,R: Resistant, I: Intermediat, nWT: non-Wild Type, WT: Wild Type

Identification Method: MOLDI TOF

Specimen submission time: 2023.8.28 14:40 Reviewer:

Laboratory test order time: 2023.8.28 10:33

Report Release Time: 2023.8.31 9:40 Laboratory Technician: Report

Ordering Physician:

**This report applies solely to the submitted specimen. For inquiries regarding test results, please contact the Laboratory prior to 17:00 on working days.**

**Name:**

**Gender:** **m**

**Age:** 59

**Department:**

**Patient ID:**

**Inpatient No.:**

Internal Medicine

**Bed No.:**

**Specimen Type:** blood

**Clinical Diagnosis:**

**Sample No.:** 230617207

**Result:** ***Candida tropicalis***

Antibiotic MIC

Amphotericin B <0.5

Fluconazole 2

Itraconazole <0.125

Voriconazole 0.062

AST Result Interpretation

WT

S

WT

S

Breakpoint

2

2-8

0.5

0.12-1

Note: S: Sensitive,R: Resistant, I: Intermediat, nWT: non-Wild Type, WT: Wild Type

Identification Method: MOLDI TOF

Specimen submission time: 2023.6.17 20:59

Laboratory test order time: 2023.6.17 18:54

Report Release Time: 2023.6.20 9:08 Laboratory Technician: Report Reviewer:

Ordering Physician:

**This report applies solely to the submitted specimen. For inquiries regarding test results, please contact the Laboratory prior to 17:00 on working days.**

**Name: Gender:** **m**

**Age:** 59

**Department: Patient ID:**

**Inpatient No.:**

Internal Medicine

**Bed No.:**

**Specimen Type:** blood

**Clinical Diagnosis:**

**Sample No.:** 240106210

**Result:** ***Candida tropicalis***

Antibiotic MIC

Amphotericin B <0.5

Fluconazole <1

Itraconazole <0.12

Voriconazole 0.12

AST Result Interpretation

WT

S

WT

S

Breakpoint

2

2-8

0.5

0.12-1

Note: S: Sensitive,R: Resistant, I: Intermediat, nWT: non-Wild Type, WT: Wild Type

Identification Method: MOLDI TOF

Specimen submission time: 2024.1.6 23:57

Laboratory test order time: 2024.1.6 22:00

Report Release Time: 2024.1.10 11:02 Laboratory Technician: Report Reviewer:

Ordering Physician:

**This report applies solely to the submitted specimen. For inquiries regarding test results, please contact the Laboratory prior to 17:00 on working days.**

**Name:**

**Gender:** **m**

**Age:** 49

**Department:**

**Patient ID:**

**Inpatient No.:**

Emergency Department

**Bed No.:**

**Specimen Type:** blood

**Clinical Diagnosis:**

**Sample No.:** 240716201

**Result:** ***Candida glabrata***

Antibiotic MIC

Amphotericin B 0.5

Caspofungin 0.25

Micafungin <=0.06

Voriconazole <=0.12

AST Result Interpretation

WT

S

S

WT

Breakpoint

2

0.12-0.5

0.06-0.25

0.25

Note: S: Sensitive,R: Resistant, I: Intermediat, nWT: non-Wild Type, WT: Wild Type

Identification Method: MOLDI TOF

Specimen submission time: 2024.7.16 6:26

Laboratory test order time: 2024.7.16 4:13

Report Release Time: 2024.7.21 7:51 Laboratory Technician: Report Reviewer:

Ordering Physician:

**This report applies solely to the submitted specimen. For inquiries regarding test results, please contact the Laboratory prior to 17:00 on working days.**

**Name: Gender:** **f**

**Age:** 37

**Department:** ICU **Patient ID:**

**Inpatient No.:**

**Bed No.:**

**Specimen Type:** blood

**Clinical Diagnosis:**

**Sample No.:** 230412213

**Result:** ***Candida albicans***

Antibiotic MIC

Amphotericin B <0.5

Fluconazole <1

AST Result Interpretation

WT

S

Breakpoint

2

2-8

Itraconazole <0.12

Voriconazole <=0.06 S 0.12-1

Note: S: Sensitive,R: Resistant, I: Intermediat, nWT: non-Wild Type, WT: Wild Type

Identification Method: MOLDI TOF

Specimen submission time: 2023.4.12 12:03 Reviewer:

Laboratory test order time: 2023.4.12 10:56

Report Release Time: 2023.4.16 14:00 Laboratory Technician: Report

Ordering Physician:

**This report applies solely to the submitted specimen. For inquiries regarding test results, please contact the Laboratory prior to 17:00 on working days.**

**Name: Gender:** **f**

**Age:** 37

**Department:** ICU **Patient ID:**

**Inpatient No.:**

**Bed No.:**

**Specimen Type:** blood

**Clinical Diagnosis:**

**Sample No.:** 240821201

**Result:** ***Candida albicans***

Antibiotic MIC

Caspofungin <=0.12

Micafungin <=0.06

Voriconazole <=0.12

Fluconazole <=0.5

Amphotericin B <=0.25

AST Result Interpretation

S

S

S

S

WT

Breakpoint

0.25-1

0.25-1

0.12-1

2-8

2

Note: S: Sensitive,R: Resistant, I: Intermediat, nWT: non-Wild Type, WT: Wild Type

Identification Method: MOLDI TOF

Specimen submission time: 2024.8.21 0:13

Laboratory test order time: 2024.8.20 19:17

Report Release Time: 2024.8.24 11:00 Laboratory Technician: Report Reviewer:

Ordering Physician:

**This report applies solely to the submitted specimen. For inquiries regarding test results, please contact the Laboratory prior to 17:00 on working days.**

**Name:** 　 **Gender: m**

**Age:** 68

**Department:** **ICU Patient ID:**

**Inpatient No.:**

**Bed No.:** 　 **Specimen Type:** **Blood**

**Clinical Diagnosis:**

**Sample No.:** **230216206**

**Result:** ***Candida albicans***

Note: S: Sensitive,R: Resistant, I: Intermediat

Identification Method: MOLDI TOF

Specimen submission time: 2023.2.16 21:42 Report Release Time: 2023.2.16 19:20 Laboratory Technician: Report Reviewer: Laboratory test order time: 2023.2.20 8:16 Ordering Physician: xxx

**This report applies solely to the submitted specimen. For inquiries regarding test results, please contact the Laboratory prior to 17:00 on working days.**

**Name:** 　 **Gender: m**

**Age:** 68

**Department:** ICU **Patient ID:**

**Inpatient No.:**

**Bed No.:**

**Specimen Type:** **Blood**

**Clinical Diagnosis:**

**Sample No.:** **230118533**

**Result:** ***Candida albicans***

Note: S: Sensitive,R: Resistant, I: Intermediat

Identification Method: MOLDI TOF

Specimen submission time:2023.1.18 20:55 Report Release Time: 2023.1.20 11:45 Laboratory Technician: Report Reviewer: Laboratory test order time: 2023.1.18 21:40 Ordering Physician:

**This report applies solely to the submitted specimen. For inquiries regarding test results, please contact the Laboratory prior to 17:00 on working days.**

**Name:** 　 **Gender: m**

**Age:** 60

**Department: Patient ID:**

**Inpatient No.:**

internal Medicine

**Bed No.: Specimen Type:**

**Clinical Diagnosis:**

**Sample No.:** 24100821**3 Blood**

**Result:** ***Candida tropicalis***

Antibiotic MIC

Caspofungin ≤0.12 Micafungin ≤0.06

Voriconazole 1

Fluconazole 32

Amphotericin B 0.5

AST Result Interpretation

S S

R

R

WT

Breakpoint

0.25-1 0.25-1

0.125-1

2-8

2

Note: S: Sensitive, R: Resistant, I: Intermediat, nWT: non-Wild Type, WT: Wild Type

Identification Method: MOLDI TOF

Specimen submission time:2024.10.8 21:17 Report Release Time: 2024.10.11 8:29 Laboratory Technician: Report Reviewer: Laboratory test order time: 2024.10.8 17:08 Ordering Physician:

**This report applies solely to the submitted specimen. For inquiries regarding test results, please contact the Laboratory prior to 17:00 on working days.**

**Name:** 　 **Gender: m**

**Age:** 81

**Department:** **ICU Patient ID:**

**Inpatient No.:**

**Bed No.:** 　 **Specimen Type:** **Blood**

**Clinical Diagnosis:**

**Sample No.:** **231001214**

**Result:** ***Candida glabrata***

Antibiotic

　Amphotericin B

Fluconazole

Itraconazole

Voriconazole

MIC AST Result Interpretation

<0.5 WT

=16 S

=0.5 WT

<0.06 WT

Breakpoint

2

32-64

4

0.25

Note: S: Sensitive, R: Resistant, I: Intermediat, nWT: non-Wild Type, WT: Wild Type

Identification Method: MOLDI TOF

Specimen submission time: 2023.10.1 20:04 Report Release Time: 2023.10.7 9:00 Laboratory Technician: Report Reviewer: Laboratory test order time: 2023.10.1 18:47 Ordering Physician:

**This report applies solely to the submitted specimen. For inquiries regarding test results, please contact the Laboratory prior to 17:00 on working days.**

**Name:** 　 **Gender: m**

**Age:** 81

**Department:** **ICU Patient ID:**

**Inpatient No.:**

**Bed No.:** 　 **Specimen Type:** **Blood**

**Clinical Diagnosis:**

**Sample No.:** **241001206**

**Result:** ***Candida glabrata***

Antibiotic

　Amphotericin B

Fluconazole

Itraconazole

Voriconazole

MIC AST Result Interpretation

<0.5 WT

<1 S

<0.12 WT

=0.12 WT

Breakpoint

2

32-64

4

0.25

Note: S: Sensitive, R: Resistant, I: Intermediat, nWT: non-Wild Type, WT: Wild Type

Identification Method: MOLDI TOF

Specimen submission time: 2024.10.1 11:23 Report Release Time: 2024.10.5 9:54 Laboratory Technician: Report Reviewer: Laboratory test order time: 2024.10.1 9:23 Ordering Physician:

**This report applies solely to the submitted specimen. For inquiries regarding test results, please contact the Laboratory prior to 17:00 on working days.**

**Name:** 　 **Gender:** **f**

**Age:** 68

**Department:** ICU **Patient ID:**

**Inpatient No.:**

**Bed No.:** 　 **Specimen Type:** **Blood**

**Clinical Diagnosis:**

**Sample No.:** **230129206**

**Result:** ***Candida tropicalis***

Antibiotic

　Amphotericin B

Fluconazole

Itraconazole

Voriconazole

MIC AST Result Interpretation

<0.5 WT

<1 S

=0.5 nWT

=0.25 I

Breakpoint

2

2-8

0.5

0.125-1

Note: S: Sensitive,R: Resistant, I: Intermediat, nWT: non-Wild Type, WT: Wild Type

Identification Method: MOLDI TOF

Specimen submission time:2023.1.29 17:10 Report Release Time: 2023.2.2 9:36 Laboratory Technician: Report Reviewer: Laboratory test order time: 2023.1.29 8:45 Ordering Physician:

**This report applies solely to the submitted specimen. For inquiries regarding test results, please contact the Laboratory prior to 17:00 on working days.**

**Name:** 　 **Gender:** **F**

**Age:** 68

**Department:** ICU **Patient ID:**

**Inpatient No.:**

**Bed No.:** 　 **Specimen Type:** **Blood**

**Clinical Diagnosis:**

**Sample No.:** **230228512**

**Result:** ***Candida tropicalis***

Antibiotic

　Amphotericin B

Fluconazole

Itraconazole

Voriconazole

MIC AST Result Interpretation

<0.5 WT

≥256 R

=1 nWT

=8 R

Breakpoint

2

2-8

0.5

0.125-1

Note: S: Sensitive,R: Resistant, I: Intermediat, nWT: non-Wild Type, WT: Wild Type

Identification Method: MOLDI TOF

Specimen submission time: 2023.2.28 10:55 Report Release Time: 20230302 10:13 Laboratory Technician: Report Reviewer Laboratory test order time: 2023.2.28 10:25 Ordering Physician

**This report applies solely to the submitted specimen. For inquiries regarding test results, please contact the Laboratory prior to 17:00 on working days.**

**Name:** 　 **Gender:** **f**

**Age:** 68

**Department:** **ICU Patient ID:**

**Inpatient No.:**

**Bed No.:** 　 **Specimen Type:** **Blood**

**Clinical Diagnosis:**

**Sample No.:** **230825218**

**Result:** ***Candida tropicalis***

Antibiotic

　Amphotericin B

Fluconazole

Itraconazole

Voriconazole

MIC AST Result Interpretation

<0.5 WT

<1 S

=0.25 WT

=0.12 S

Breakpoint

2

2-8

0.5

0.125-1

Note: S: Sensitive,R: Resistant, I: Intermediat, nWT: non-Wild Type, WT: Wild Type

Identification Method: MOLDI TOF

Specimen submission time:2023.8.25 15:55 Report Release Time: 2023.8.28 8:26 Laboratory Technician: Report Reviewer Laboratory test order time: 2023.8.25 9:38 Ordering Physician:

**This report applies solely to the submitted specimen. For inquiries regarding test results, please contact the Laboratory prior to 17:00 on working days.**

**Name:** 　 **Gender:** **f**

**Age:** 70

**Department:** NICU **Patient ID:**

**Inpatient No.:**

**Bed No.:** 　 **Specimen Type:** **Blood**

**Clinical Diagnosis:**

**Sample No.:** **231210202**

**Result:** ***Candida tropicalis***

Antibiotic

　Amphotericin B

Fluconazole

Itraconazole

Voriconazole

MIC AST Result Interpretation

<0.5 WT

≥256 R

≥8 nWT

≤0.12 S

Breakpoint

2

2-8

0.5

0.125-1

Note: S: Sensitive,R: Resistant, I: Intermediat, nWT: non-Wild Type, WT: Wild Type

Identification Method: MOLDI TOF

Specimen submission time: 2023.12.10 7:07 Report Release Time: 2023.12.13 9:16 Laboratory Technician: Report Reviewer: Laboratory test order time: 2023.12.9 18:58 Ordering Physician:

**This report applies solely to the submitted specimen. For inquiries regarding test results, please contact the Laboratory prior to 17:00 on working days.**

**Name:** 　 **Gender: m**

**Age:** 81

**Department:** ICU **Patient ID:**

**Inpatient No.:**

**Bed No.:** 　 **Specimen Type:** **Blood**

**Clinical Diagnosis:**

**Sample No.:** **230404201**

**Result:** ***Candida glabrata***

Antibiotic

　Amphotericin B

Fluconazole

Itraconazole

Voriconazole

MIC AST Result Interpretation

<0.5 WT

=16 S

=0.5 WT

<0.06 WT

Breakpoint

2

32-64

4

0.25

Note: S: Sensitive,R: Resistant, I: Intermediat, nWT: non-Wild Type, WT: Wild Type

Identification Method: MOLDI TOF

Specimen submission time: 2023.4.4.1:27 Report Release Time: 2023.4.9 17:25 Laboratory Technician: Report Reviewer: Laboratory test order time: 2023.4.4 0:39 Ordering Physician:

**This report applies solely to the submitted specimen. For inquiries regarding test results, please contact the Laboratory prior to 17:00 on working days.**

**Name:** 　 **Gender: M**

**Age:** 81

**Department:** **ICU Patient ID:**

**Inpatient No.:**

**Bed No.:** 　 **Specimen Type:** **Blood**

**Clinical Diagnosis:**

**Sample No.:** **230831222**

**Result:** ***Candida glabrata***

Note: S: Sensitive,R: Resistant, I: Intermediat, nWT: non-Wild Type, WT: Wild Type

Identification Method: MOLDI TOF

Specimen submission time: 2023.8.31 22:15 Report Release Time: 2023.9.4 10:19 Laboratory Technician: Report Reviewer: Laboratory test order time: 2023.8.3118:57 Ordering Physician:

**This report applies solely to the submitted specimen. For inquiries regarding test results, please contact the Laboratory prior to 17:00 on working days.**

**Name:** 　 **Gender:** **F**

**Age:** 70

**Department:** ICU **Patient ID:**

**Inpatient No.:**

**Bed No.:** 　 **Specimen Type:** **Blood**

**Clinical Diagnosis:**

**Sample No.:** **230919206**

**Result:** ***Candida tropicalis***

Antibiotic

　Amphotericin B

Fluconazole

Itraconazole

Voriconazole

MIC AST Result Interpretation

<0.5 WT

≥256 R

≥8 nWT

≥16 R

Breakpoint

2

2-8

0.5

0.125-1

Note: S: Sensitive,R: Resistant, I: Intermediat, nWT: non-Wild Type, WT: Wild Type

Identification Method: MOLDI TOF

Specimen submission time: 2023.9.19 11:57 Report Release Time: 2023.9.22 10:01 Laboratory Technician: Report Reviewer: Laboratory test order time: 2023.9.19 10:46 Ordering Physician:

**This report applies solely to the submitted specimen. For inquiries regarding test results, please contact the Laboratory prior to 17:00 on working days.**

**Name:** 　 **Gender:** **f**

**Age:** 70

**Department:** ICU **Patient ID:**

**Inpatient No.:**

**Bed No.:** 　 **Specimen Type: Blood**

**Clinical Diagnosis:**

**Sample No.:** **230906206**

**Result:** ***Candida tropicalis***

Antibiotic MIC

Amphotericin B <0.5 Caspofungin 0.25

Fluconazole ≥256

Itraconazole ≥8 Micafungin 0.06

Voriconazole <0.06

AST Result Interpretation

WT S

R

nWT S

R

Breakpoint

　2 0.25-1

2-8

0.5 0.25-1

0.125-1

Note: S: Sensitive,R: Resistant, I: Intermediat, nWT: non-Wild Type, WT: Wild Type

Identification Method: MOLDI TOF

Specimen submission time: 2023.9.6 9:10 Report Release Time: 2023.9.10 11:21 Laboratory Technician: Report Reviewer: Laboratory test order time: 2023.9.6 6:46 Ordering Physician

**This report applies solely to the submitted specimen. For inquiries regarding test results, please contact the Laboratory prior to 17:00 on working days.**

**Name:** 　 **Gender:** **F**

**Age:** 70

**Department:** **ICU Patient ID:**

**Inpatient No.:**

**Bed No.:** 　 **Specimen Type: Blood**

**Clinical Diagnosis:**

**Sample No.:** **240105202**

**Result:** ***Candida albicans***

Antibiotic

　Amphotericin B

Fluconazole

MIC AST Result Interpretation

<0.5 WT

<1 S

Breakpoint

　2

2-8

Itraconazole <0.12

Voriconazole <0.06 S 0.125-1

Note: S: Sensitive,R: Resistant, I: Intermediat, nWT: non-Wild Type, WT: Wild Type

Identification Method: MOLDI TOF

Specimen submission time: 2024.1.5 1:56 Report Release Time: 2024.1.9 9:38 Laboratory Technician: Report Reviewer: Laboratory test order time: 2024.1.4 21:48 Ordering Physician:

**This report applies solely to the submitted specimen. For inquiries regarding test results, please contact the Laboratory prior to 17:00 on working days.**

**Name:** 　 **Gender:** **f**

**Age:** 70

**Department:** **ICU Patient ID:**

**Inpatient No.:**

**Bed No.:** 　 **Specimen Type: Blood**

**Clinical Diagnosis:**

**Sample No.:** **240410203**

**Result:** ***Candida albicans***

Antibiotic MIC

Amphotericin B <0.5

Fluconazole <1

AST Result Interpretation

WT

S

Breakpoint

2

2-8

Itraconazole <0.12

Voriconazole <0.06 S 0.125-1

Note: S: Sensitive,R: Resistant, I: Intermediat, nWT: non-Wild Type, WT: Wild Type

Identification Method: MOLDI TOF

Specimen submission time: 2024.4.10 3:33 Report Release Time: 2024.4.16 9:09 Laboratory Technician Report Reviewer Laboratory test order time: 2024.4.9 20:33 Ordering Physician:

**This report applies solely to the submitted specimen. For inquiries regarding test results, please contact the Laboratory prior to 17:00 on working days.**

**Name:** 　 **Gender: m**

**Age:** 81

**Department:** ICU **Patient ID:**

**Inpatient No.:**

**Bed No.:** 　 **Specimen Type: Blood**

**Clinical Diagnosis:**

**Sample No.:** **230404201**

**Result:** ***Candida glabrata***

Antibiotic

　Amphotericin B

Fluconazole

Itraconazole

Voriconazole

MIC AST Result Interpretation

<0.5 WT

=16 S

=0.5 WT

<0.06 WT

Breakpoint

2

32-64

4

0.25

Note: S: Sensitive,R: Resistant, I: Intermediat, nWT: non-Wild Type, WT: Wild Type

Identification Method: MOLDI TOF

Specimen submission time: 2023.4.4 1:27 Report Release Time: 2023.4.9 17:25 Laboratory Technician: Report Reviewer Laboratory test order time: 2023.4.4 0:39 Ordering Physician:

**This report applies solely to the submitted specimen. For inquiries regarding test results, please contact the Laboratory prior to 17:00 on working days.**

**Name:** 　 **Gender:** **f**

**Age:** 70

**Department:** **ICU Patient ID:**

**Inpatient No.:**

**Bed No.:** 　 **Specimen Type: Blood**

**Clinical Diagnosis:**

**Sample No.:** **241116203**

**Result:** ***Candida albicans***

Antibiotic MIC

Caspofungin ≤0.12 Micafungin　 ≤0.06

Voriconazole ≤0.12

Fluconazole ≤0.5

Amphotericin B ≤0.25

AST Result Interpretation

S S

S

S

WT

Breakpoint

0.25-1 0.25-1

0.125-1

2-8

2

Note: S: Sensitive,R: Resistant, I: Intermediat, nWT: non-Wild Type, WT: Wild Type

Identification Method: MOLDI TOF

Specimen submission time: 2024.11.16 10:56 Report Release Time: 2024.11.20 8:34 Laboratory Technician: Report Reviewer Laboratory test order time: 2021.11.16 9:28 Ordering Physician:

**This report applies solely to the submitted specimen. For inquiries regarding test results, please contact the Laboratory prior to 17:00 on working days.**

**Name:** 　 **Gender:** **f**

**Age:** 70

**Department:** **ICU Patient ID:**

**Inpatient No.:**

**Bed No.:** 　 **Specimen Type: Blood**

**Clinical Diagnosis:**

**Sample No.:** **241119212**

**Result:** ***Candida albicans***

Antibiotic MIC

Caspofungin ≤0.12 Micafungin　 ≤0.06

Voriconazole ≤0.12

Fluconazole ≤0.5

Amphotericin B ≤0.25

AST Result Interpretation

S S

S

S

WT

Breakpoint

0.25-1 0.25-1

0.125-1

2-8

2

Note: S: Sensitive,R: Resistant, I: Intermediat, nWT: non-Wild Type, WT: Wild Type

Identification Method: MOLDI TOF

Specimen submission time: 2024.11.19 16:38 Report Release Time: 2024.11.14 8:48 Laboratory Technician: Report Reviewer: Laboratory test order time: 2024.11.19 15:35 Ordering Physician:

**This report applies solely to the submitted specimen. For inquiries regarding test results, please contact the Laboratory prior to 17:00 on working days.**

**Name:** 　 **Gender:** **F**

**Age:** 70

**Department:** ICU **Patient ID:**

**Inpatient No.:**

**Bed No.:** 　 **Specimen Type: Blood**

**Clinical Diagnosis:**

**Sample No.:** **200110023**

**Result:** ***Candida albicans***

Antibiotic MIC Fluconazole <1

Itraconazole ≤0.12

Voriconazole ≤0.06

AST Result Interpretation

S

S

S

Breakpoint

2-8

0.125-1

0.125-1

Note: S: Sensitive,R: Resistant, I: Intermediat

Identification Method: MOLDI TOF

Specimen submission time: 2020.2.20 11:56 Report Release Time: 2020.2.25 8:01 Laboratory Technician: Report Reviewer Laboratory test order time: 2020.2.20 9:05 Ordering Physician:

**This report applies solely to the submitted specimen. For inquiries regarding test results, please contact the Laboratory prior to 17:00 on working days.**

**Name:** 　 **Gender: m**

**Age:** 65

**Department: Patient ID:**

**Inpatient No.:**

Internal Medicine

**Bed No.:** 　 **Specimen Type: Blood**

**Clinical Diagnosis:**

**Sample No.:** **240424202**

**Result:** ***Candida tropicalis***

Antibiotic

　Amphotericin B

Fluconazole

Itraconazole

Voriconazole

MIC AST Result Interpretation

<0.5 WT

<1 S

≥8 nWT

0.125 S

Breakpoint

2

2-8

0.5

0.125-1

Note: S: Sensitive,R: Resistant, I: Intermediat, nWT: non-Wild Type, WT: Wild Type

Identification Method: MOLDI TOF

Specimen submission time: 2024.4.24 9:36 Report Release Time: 2024.4.27 8:11 Laboratory Technician: Report Reviewer: Laboratory test order time: 2024.4.24 7:55 Ordering Physician:

**This report applies solely to the submitted specimen. For inquiries regarding test results, please contact the Laboratory prior to 17:00 on working days.**

**Name:** 　 **Gender:** **f**

**Age:** 70

**Department:** **ICU Patient ID:**

**Inpatient No.:**

**Bed No.:** 　 **Specimen Type: Blood**

**Clinical Diagnosis:**

**Sample No.:** **241118204**

**Result:** ***Candida albicans***

Antibiotic MIC

Caspofungin ≤0.12 Micafungin　 ≤0.06

Voriconazole ≤0.12

Fluconazole ≤0.5

Amphotericin B ≤0.25

AST Result Interpretation

S S

S

S

WT

Breakpoint

0.25-1 0.25-1

0.125-1

2-8

2

Note: S: Sensitive,R: Resistant, I: Intermediat, nWT: non-Wild Type, WT: Wild Type

Identification Method: MOLDI TOF

Specimen submission time: 2024.11.18 Report Release Time: 2024.11.21 Laboratory Technician: Report Reviewer: Laboratory test order time: 2024.11.18 Ordering Physician:

**This report applies solely to the submitted specimen. For inquiries regarding test results, please contact the Laboratory prior to 17:00 on working days.**

**Name:** 　 **Gender:** **f**

**Age:** 70

**Department:** ICU **Patient ID:**

**Inpatient No.:**

**Bed No.:** 　 **Specimen Type: Blood**

**Clinical Diagnosis:**

**Sample No.:** **240511215**

**Result:** ***Candida albicans***

Antibiotic

　Amphotericin B

Fluconazole

MIC AST Result Interpretation

<0.5 WT

<1 S

Breakpoint

2

2-8

Itraconazole <0.12

Voriconazole <0.06 S 0.125-1

Note: S: Sensitive,R: Resistant, I: Intermediat, nWT: non-Wild Type, WT: Wild Type

Identification Method: MOLDI TOF

Specimen submission time: 2024.5.11

Laboratory test order time: 2024.5.11

Report Release Time: 2024.5.17 Laboratory Technician: Report Reviewer:

Ordering Physician:

**This report applies solely to the submitted specimen. For inquiries regarding test results, please contact the Laboratory prior to 17:00 on working days.**

**Name:** 　 **Gender: m**

**Age:** 16

**Department: Patient ID:**

**Inpatient No.:**

internal Medicine

**Bed No.:** 　 **Specimen Type: Blood**

**Clinical Diagnosis:**

**Sample No.:** **241028204**

**Result:** ***Candida tropicalis***

Antibiotic MIC

Caspofungin ≤0.12 Micafungin　 ≤0.06

Voriconazole ≤0.12

Fluconazole ≤0.5

Amphotericin B ≤0.25

AST Result Interpretation

S S

S

S

WT

Breakpoint

0.25-1 0.25-1

0.12-1

2-8

2

Note: S: Sensitive,R: Resistant, I: Intermediat, nWT: non-Wild Type, WT: Wild Type

Identification Method: MOLDI TOF

Specimen submission time: 2024.10.28

Laboratory test order time: 2024.10.28

Report Release Time: 2024.10.31 Laboratory Technician: Report Reviewer:

Ordering Physician:

**This report applies solely to the submitted specimen. For inquiries regarding test results, please contact the Laboratory prior to 17:00 on working days.**

**Name:** 　 **Gender: M**

**Age:** 60

**Department: Patient ID:**

**Inpatient No.:**

Internal Medicine

**Bed No.:** 　 **Specimen Type: Blood**

**Clinical Diagnosis:**

**Sample No.:** **240106210**

**Result:** ***Candida tropicalis***

Antibiotic

　Amphotericin B

Fluconazole

Itraconazole

Voriconazole

MIC AST Result Interpretation

<0.5 WT

<1 S

<0.12 WT

=0.12 S

Breakpoint

2

2-8 0.5

0.125-1

Note: S: Sensitive,R: Resistant, I: Intermediat, nWT: non-Wild Type, WT: Wild Type

Identification Method: MOLDI TOF

Specimen submission time: 2024.1.6

Laboratory test order time: 2024.1.6

Report Release Time: 2024.1.10 Laboratory Technician: Report Reviewer:

Ordering Physician:

**This report applies solely to the submitted specimen. For inquiries regarding test results, please contact the Laboratory prior to 17:00 on working days.**

**Name:** 　 **Gender: m**

**Age:** 60

**Department: Patient ID:**

**Inpatient No.:**

internal Medicine **Bed No.:** 　 **Specimen Type: Blood**

**Clinical Diagnosis:**

**Sample No.:** **241020204**

**Result:** ***Candida tropicalis***

Antibiotic MIC

Caspofungin ≤0.12 Micafungin　 ≤0.06

Voriconazole ≤0.12

Fluconazole 1

Amphotericin B ≤0.25

AST Result Interpretation

S S

S

S

WT

Breakpoint

0.25-1 0.25-1

0.125-1

2-8

2

Note: S: Sensitive,R: Resistant, I: Intermediat

Identification Method: MOLDI TOF

Specimen submission time: 2024.10.20

Laboratory test order time: 2024.10.20

Report Release Time: 2024.10.24 Laboratory Technician: Report Reviewer:

Ordering Physician:

**This report applies solely to the submitted specimen. For inquiries regarding test results, please contact the Laboratory prior to 17:00 on working days.**

**Name:** 　 **Gender: M**

**Age:** 74

**Department:** ICU **Patient ID:**

**Inpatient No.:**

**Bed No.:** 　 **Specimen Type: Blood**

**Clinical Diagnosis:**

**Sample No.:** **231022209**

**Result:** ***Candida glabrata***

Antibiotic

　Amphotericin B

Fluconazole

Itraconazole

Voriconazole

MIC AST Result Interpretation

<0.5 WT

<1 S

<0.12 WT

<0.06 WT

Breakpoint

2

32-64

4

0.25

Note: S: Sensitive,R: Resistant, I: Intermediat, nWT: non-Wild Type, WT: Wild Type

Identification Method: MOLDI TOF

Specimen submission time: 2023.10.22

Laboratory test order time: 2023.10.22

Report Release Time: 2023.10.25 Laboratory Technician: Report Reviewer:

Ordering Physician:

**This report applies solely to the submitted specimen. For inquiries regarding test results, please contact the Laboratory prior to 17:00 on working days.**

**Name:** 　 **Gender:** **F**

**Age:** 85

**Department:** NICU **Patient ID:**

**Inpatient No.:**

**Bed No.:** 　 **Specimen Type: Blood**

**Clinical Diagnosis:**

**Sample No.:** **15378695**

**Result:** ***Candida albicans***

Antibiotic MIC AST Result Interpretation Breakpoint

Amphotericin B <0.5 Fluconazole <1

Itraconazole <0.125

Voriconazole <0.062

Note: S: Sensitive,R: Resistant, I: Intermediat

Identification Method: MOLDI TOF

Specimen submission time: 2015.1.28

Laboratory test order time: 2015.1.28

Report Release Time: 2015.2.1 Laboratory Technician: Report Reviewer:

Ordering Physician:

**This report applies solely to the submitted specimen. For inquiries regarding test results, please contact the Laboratory prior to 17:00 on working days.**

**Name:** 　 **Gender:** **f**

**Age:** 85

**Department:** **NICU Patient ID:**

**Inpatient No.:**

**Bed No.:** 　 **Specimen Type: Blood**

**Clinical Diagnosis:**

**Sample No.:** **15378820**

**Result:** ***Candida albicans***

Antibiotic MIC AST Result Interpretation Breakpoint

Amphotericin B <0.5 Fluconazole <1

Itraconazole <0.125

Voriconazole <0.062

Note: S: Sensitive,R: Resistant, I: Intermediat

Identification Method: MOLDI TOF

Specimen submission time: 2015.1.31

Laboratory test order time: 2015.1.31

Report Release Time: 2015.2.4 Laboratory Technician: Report Reviewer:

Ordering Physician:

**This report applies solely to the submitted specimen. For inquiries regarding test results, please contact the Laboratory prior to 17:00 on working days.**

**Name:**

**Gender:** **M**

**Age:** 68

**Department:**

**Patient ID:**

**Inpatient No.:**

emergency department

**Bed No.:**

**Specimen Type: Blood**

**Clinical Diagnosis:**

**Sample No.:** **15383166**

**Result:** ***Candida glabrata***

Antibiotic MIC AST Result Interpretation Breakpoint

Amphotericin B <0.5 Fluconazole <1

Itraconazole <0.125

Voriconazole <0.062

Note: S: Sensitive,R: Resistant, I: Intermediat

Identification Method: MOLDI TOF

Specimen submission time: 2015.4.4

Laboratory test order time: 2015.4.4

Report Release Time: 2015.4.7 Laboratory Technician: Report Reviewer:

Ordering Physician:

**This report applies solely to the submitted specimen. For inquiries regarding test results, please contact the Laboratory prior to 17:00 on working days.**

**Name:** 　 **Gender:** **F**

**Age:** 73

**Department:** **ICU Patient ID:**

**Inpatient No.:**

**Bed No.:** 　 **Specimen Type: Blood**

**Clinical Diagnosis:**

**Sample No.:** **15383928**

**Result:** ***Candida albicans***

Antibiotic MIC AST Result Interpretation Breakpoint

Amphotericin B <0.5 Fluconazole =8

Itraconazole <0.125

Voriconazole =0.25

Note: S: Sensitive,R: Resistant, I: Intermediat

Identification Method: MOLDI TOF

Specimen submission time: 2015.4.15

Laboratory test order time: 2015.4.15

Report Release Time: 2015.4.19 Laboratory Technician: Report Reviewer:

Ordering Physician:

**This report applies solely to the submitted specimen. For inquiries regarding test results, please contact the Laboratory prior to 17:00 on working days.**

**Name:** 　 **Gender:** **f**

**Age:** 73

**Department:** **ICU Patient ID:**

**Inpatient No.:**

**Bed No.:** 　 **Specimen Type: Blood**

**Clinical Diagnosis:**

**Sample No.:** **15383929**

**Result:** ***Candida albicans***

Antibiotic MIC AST Result Interpretation Breakpoint

Amphotericin B =1 Fluconazole <1

Itraconazole <0.125

Voriconazole <0.062

Note: S: Sensitive,R: Resistant, I: Intermediat

Identification Method: MOLDI TOF

Specimen submission time: 2015.4.15

Laboratory test order time: 2015.4.15

Report Release Time: 2015.4.15 Laboratory Technician: Report Reviewer:

Ordering Physician:

**This report applies solely to the submitted specimen. For inquiries regarding test results, please contact the Laboratory prior to 17:00 on working days.**

**Name:** 　 **Gender:** **f**

**Age:** 42

**Department: Patient ID:**

**Inpatient No.:**

internal Medicine

**Bed No.:** 　 **Specimen Type: Blood**

**Clinical Diagnosis:**

**Sample No.:** **15285028**

**Result:** ***Candida tropicalis***

Antibiotic MIC AST Result Interpretation Breakpoint

Amphotericin B <0.5 Fluconazole >128

Itraconazole >4

Voriconazole >8

Note: S: Sensitive,R: Resistant, I: Intermediat

Identification Method: MOLDI TOF

Specimen submission time: 2015.5.2

Laboratory test order time: 2015.4.30

Report Release Time: 2015.5.9 Laboratory Technician: Report Reviewer:

Ordering Physician:

**This report applies solely to the submitted specimen. For inquiries regarding test results, please contact the Laboratory prior to 17:00 on working days.**

**Name:**

**Gender:** **f**

**Age:** 66

**Department:**

**Patient ID:**

**Inpatient No.:**

surgical department

**Bed No.:**

**Specimen Type: Blood**

**Clinical Diagnosis:**

**Sample No.:** **15388252**

**Result:** ***Candida krusei***

Antibiotic MIC AST Result Interpretation Breakpoint

Amphotericin B <0.5 Fluconazole =16

Itraconazole =1

Voriconazole =0.5

Note: S: Sensitive,R: Resistant, I: Intermediat

Identification Method: MOLDI TOF

Specimen submission time: 2015.6.16

Laboratory test order time: 2015.6.16

Report Release Time: 2015.6.16 Laboratory Technician: Report Reviewer:

Ordering Physician:

**This report applies solely to the submitted specimen. For inquiries regarding test results, please contact the Laboratory prior to 17:00 on working days.**

**Name:** 　 **Gender: m**

**Age:** 20d

**Department: Patient ID:**

**Inpatient No.:**

Neonatology

**Bed No.:** 　 **Specimen Type: Blood**

**Clinical Diagnosis:**

**Sample No.:** **15388919**

**Result:** ***Candida albicans***

Antibiotic

5-Fluorocytosine 　 Amphotericin B

Fluconazole Itraconazole

Voriconazole

MIC AST Result Interpretation

≤4 S

<0.5 S =2 S

=0.25 I

=0.125 S

Breakpoint

Note: S: Sensitive,R: Resistant, I: Intermediat

Identification Method: MOLDI TOF

Specimen submission time: 2015.6.26

Laboratory test order time: 2015.6.26

Report Release Time: 2015.7.1 Laboratory Technician: Report Reviewer:

Ordering Physician

**This report applies solely to the submitted specimen. For inquiries regarding test results, please contact the Laboratory prior to 17:00 on working days.**

**Name:** 　 **Gender: m**

**Age:** 23d

**Department: Patient ID:**

**Inpatient No.:**

Neonatology

**Bed No.:** 　 **Specimen Type: Blood**

**Clinical Diagnosis:**

**Sample No.:** **24121305**

**Result:** ***Candida albicans***

Antibiotic MIC

Caspofungin ≤0.12 Micafungin　 ≤0.06

Voriconazole ≤0.12

Fluconazole ≤0.5

Amphotericin B 1

AST Result Interpretation

S S

S

S

WT

Breakpoint

0.25-1 0.25-1

0.125-1

2-8

2

Note: S: Sensitive,R: Resistant, I: Intermediat, nWT: non-Wild Type, WT: Wild Type

Identification Method: MOLDI TOF

Specimen submission time: 2024.12.13

Laboratory test order time: 2024.12.13

Report Release Time: 2024.12.19 Laboratory Technician: Report Reviewer:

Ordering Physician:

**This report applies solely to the submitted specimen. For inquiries regarding test results, please contact the Laboratory prior to 17:00 on working days.**

**Name:** 　 **Gender:** **f**

**Age:** 76

**Department: Patient ID:**

**Inpatient No.:**

internal Medicine

**Bed No.:** 　 **Specimen Type: Blood**

**Clinical Diagnosis:**

**Sample No.:** **15389211**

**Result:** ***Candida parapsilosis***

Antibiotic MIC AST Result Interpretation Breakpoint

Amphotericin B <0.5 Fluconazole <1

Itraconazole <0.125

Voriconazole <0.062

Note: S: Sensitive,R: Resistant, I: Intermediat

Identification Method: MOLDI TOF

Specimen submission time: 2015.6.30

Laboratory test order time: 2015.6.30

Report Release Time: 2015.7.7 Laboratory Technician: Report Reviewer:

Ordering Physician:

**This report applies solely to the submitted specimen. For inquiries regarding test results, please contact the Laboratory prior to 17:00 on working days.**

**Name:** 　 **Gender: m**

**Age:** 26

**Department:** **ICU Patient ID:**

**Inpatient No.:**

**Bed No.:** 　 **Specimen Type: Blood**

**Clinical Diagnosis:**

**Sample No.:** **15389371**

**Result:** ***Candida albicans***

Antibiotic MIC AST Result Interpretation Breakpoint

Amphotericin B <0.5 Fluconazole <1

Itraconazole <0.125

Voriconazole <0.062

Note: S: Sensitive,R: Resistant, I: Intermediat

Identification Method: MOLDI TOF

Specimen submission time: 2015.7.2

Laboratory test order time: 2015.7.2

Report Release Time: 2015.7.2 Laboratory Technician: Report Reviewer:

Ordering Physician:

**This report applies solely to the submitted specimen. For inquiries regarding test results, please contact the Laboratory prior to 17:00 on working days.**

**Name:** 　 **Gender: m**

**Age:** 55

**Department: Patient ID:**

**Inpatient No.:**

internal Medicine

**Bed No.:** 　 **Specimen Type: Blood**

**Clinical Diagnosis:**

**Sample No.:** **231127051**

**Result:** ***Candida guilliermondii***

Antibiotic

　Amphotericin B

Fluconazole

MIC AST Result Interpretation

0.5

128

Breakpoint

Itraconazole 2

Voriconazole 4

Note: S: Sensitive,R: Resistant, I: Intermediat

Identification Method: MOLDI TOF

Specimen submission time: 2023.11.27

Laboratory test order time: 2023.11.27

Report Release Time: 2023.12.1 Laboratory Technician: Report Reviewer:

Ordering Physician:

**This report applies solely to the submitted specimen. For inquiries regarding test results, please contact the Laboratory prior to 17:00 on working days.**

**Name:** 　 **Gender: m**

**Age:** 54

**Department:** EICU **Patient ID:**

**Inpatient No.:**

**Bed No.:** 　 **Specimen Type: Blood**

**Clinical Diagnosis:**

**Sample No.:** **15396204**

**Result:** ***Candida famata***

Note: S: Sensitive,R: Resistant, I: Intermediat

Identification Method: MOLDI TOF

Specimen submission time: 2015.9.29

Laboratory test order time: 2015.9.29

Report Release Time: 2015.10.2 Laboratory Technician: Report Reviewer:

Ordering Physician

**This report applies solely to the submitted specimen. For inquiries regarding test results, please contact the Laboratory prior to 17:00 on working days.**

**Name:** 　 **Gender: m**

**Age:** 54

**Department: Patient ID:**

**Inpatient No.:**

Internal Medicine

**Bed No.:** 　 **Specimen Type: Blood**

**Clinical Diagnosis:**

**Sample No.:** **160820029**

**Result:** ***Candida intermedia***

Antibiotic Amphotericin B Fluconazole

Itraconazole

MIC AST Result Interpretation Breakpoint

<0.5 =2

<0.125

Voriconazole <0.062

Note: S: Sensitive,R: Resistant, I: Intermediat

Identification Method: MOLDI TOF

Specimen submission time: 2016.8.20

Laboratory test order time: 2016.8.20

Report Release Time: 2016.8.24 Laboratory Technician: Report Reviewer:

Ordering Physician:

**This report applies solely to the submitted specimen. For inquiries regarding test results, please contact the Laboratory prior to 17:00 on working days.**

**Name:** 　 **Gender: m**

**Age:** 54

**Department: Patient ID:**

**Inpatient No.:**

Internal Medicine

**Bed No.:** 　 **Specimen Type: Blood**

**Clinical Diagnosis:**

**Sample No.:** **210311004**

**Result:** ***Candida famata***

Antibiotic

　Amphotericin B

Fluconazole

MIC AST Result Interpretation

=1

<1

Breakpoint

Itraconazole <0.12

Voriconazole <0.06

Note: S: Sensitive,R: Resistant, I: Intermediat

Identification Method: MOLDI TOF

Specimen submission time: 2021.3.11

Laboratory test order time: 2021.3.11

Report Release Time: 2021.3.14 Laboratory Technician: Report Reviewer:

Ordering Physician:

**This report applies solely to the submitted specimen. For inquiries regarding test results, please contact the Laboratory prior to 17:00 on working days.**

**Name:** 　 **Gender:** **f**

**Age:** 46

**Department:** **ICU Patient ID:**

**Inpatient No.:**

**Bed No.:** 　 **Specimen Type: Blood**

**Clinical Diagnosis:**

**Sample No.:** **15392891**

**Result:** ***Candida tropicalis***

Antibiotic MIC AST Result Interpretation Breakpoint

Amphotericin B <0.5 Fluconazole >128

Itraconazole >4

Voriconazole <0.062

Note: S: Sensitive,R: Resistant, I: Intermediat

Identification Method: MOLDI TOF

Specimen submission time: 2015.8.18

Laboratory test order time: 2015.8.18

Report Release Time: 2015.8.22 Laboratory Technician: Report Reviewer:

Ordering Physician:

**This report applies solely to the submitted specimen. For inquiries regarding test results, please contact the Laboratory prior to 17:00 on working days.**

**Name:** 　 **Gender:** **f**

**Age:** 46

**Department:** ICU **Patient ID:**

**Inpatient No.:**

**Bed No.:** 　 **Specimen Type: Blood**

**Clinical Diagnosis:**

**Sample No.:** **15392894**

**Result:** ***Candida tropicalis***

Antibiotic MIC AST Result Interpretation Breakpoint

Amphotericin B <0.5 Fluconazole >128

Itraconazole >4

Voriconazole <0.062

Note: S: Sensitive,R: Resistant, I: Intermediat

Identification Method: MOLDI TOF

Specimen submission time: 2015.8.19

Laboratory test order time: 2015.8.19

Report Release Time: 2015.8.22 Laboratory Technician: Report Reviewer:

Ordering Physician:

**This report applies solely to the submitted specimen. For inquiries regarding test results, please contact the Laboratory prior to 17:00 on working days.**

**Name:** 　 **Gender:** **f**

**Age:** 66

**Department: Patient ID:**

**Inpatient No.:**

gynecology

**Bed No.:** 　 **Specimen Type: Blood**

**Clinical Diagnosis:**

**Sample No.:** **15393785**

**Result:** ***Candida parapsilosis***

Antibiotic MIC AST Result Interpretation Breakpoint

Amphotericin B <0.5 Fluconazole <1

Itraconazole <0.125

Voriconazole <0.062

Note: S: Sensitive,R: Resistant, I: Intermediat

Identification Method: MOLDI TOF

Specimen submission time: 2015.8.29

Laboratory test order time: 2015.8.29

Report Release Time: 2015.9.3 Laboratory Technician: Report Reviewer:

Ordering Physician:

**This report applies solely to the submitted specimen. For inquiries regarding test results, please contact the Laboratory prior to 17:00 on working days.**

**Name:**

**Gender:** **f**

**Age:** 56

**Department:**

**Patient ID:**

**Inpatient No.:**

emergency department

**Bed No.:**

**Specimen Type: Blood**

**Clinical Diagnosis:**

**Sample No.:** **15393802**

**Result:** ***Candida parapsilosis***

Antibiotic MIC AST Result Interpretation Breakpoint

Amphotericin B <0.5 Fluconazole <1

Itraconazole <0.125

Voriconazole <0.062

Note: S: Sensitive,R: Resistant, I: Intermediat

Identification Method: MOLDI TOF

Specimen submission time: 2015.8.29

Laboratory test order time: 2015.8.29

Report Release Time: 2015.9.3 Laboratory Technician: Report Reviewer:

Ordering Physician

**This report applies solely to the submitted specimen. For inquiries regarding test results, please contact the Laboratory prior to 17:00 on working days.**

**Name:** 　 **Gender: m**

**Age:** 47

**Department:** **ICU Patient ID:**

**Inpatient No.:**

**Bed No.:** 　 **Specimen Type: Blood**

**Clinical Diagnosis:**

**Sample No.:** **15396545**

**Result:** ***Candida tropicalis***

Antibiotic MIC AST Result Interpretation Breakpoint

Amphotericin B <0.5 Fluconazole <1

Itraconazole <0.125

Voriconazole =0.125

Note: S: Sensitive,R: Resistant, I: Intermediat

Identification Method: MOLDI TOF

Specimen submission time: 2015.10.5

Laboratory test order time: 2015.10.5

Report Release Time: 2015.10.9 Laboratory Technician: Report Reviewer:

Ordering Physician

**This report applies solely to the submitted specimen. For inquiries regarding test results, please contact the Laboratory prior to 17:00 on working days.**

**Name:**

**Gender:** **m**

**Age:** 86

**Department:**

**Patient ID:**

**Inpatient No.:**

emergency department

**Bed No.:**

**Specimen Type: Blood**

**Clinical Diagnosis:**

**Sample No.:** **15396867**

**Result:** ***Candida albicans***

Antibiotic MIC AST Result Interpretation Breakpoint

Amphotericin B <0.5 Fluconazole <1

Itraconazole <0.125

Voriconazole <0.062

Note: S: Sensitive,R: Resistant, I: Intermediat

Identification Method: MOLDI TOF

Specimen submission time: 2015.10.9

Laboratory test order time: 2015.10.9

Report Release Time: 2015.10.12 Laboratory Technician: Report Reviewer:

Ordering Physician:

**This report applies solely to the submitted specimen. For inquiries regarding test results, please contact the Laboratory prior to 17:00 on working days.**

**Name:**

**Gender:** **m**

**Age:** 48

**Department:**

**Patient ID:**

**Inpatient No.:**

emergency department

**Bed No.:**

**Specimen Type: Blood**

**Clinical Diagnosis:**

**Sample No.:** **15397437**

**Result:** ***Candida parapsilosis***

Antibiotic MIC AST Result Interpretation Breakpoint

Amphotericin B <0.5 Fluconazole <1

Itraconazole <0.125

Voriconazole <0.062

Note: S: Sensitive,R: Resistant, I: Intermediat

Identification Method: MOLDI TOF

Specimen submission time: 2015.10.17

Laboratory test order time: 2015.10.17

Report Release Time: 2015.10.22 Laboratory Technician: Report Reviewer:

Ordering Physician

**This report applies solely to the submitted specimen. For inquiries regarding test results, please contact the Laboratory prior to 17:00 on working days.**

**Name:** 　 **Gender: m**

**Age:** 42

**Department:** NICU **Patient ID:**

**Inpatient No.:**

**Bed No.:** 　 **Specimen Type: Blood**

**Clinical Diagnosis:**

**Sample No.:** **15002057**

**Result:** ***Candida albicans***

Antibiotic MIC AST Result Interpretation Breakpoint

Amphotericin B <0.5 Fluconazole =2

Itraconazole =0.5

Voriconazole =0.25

Note: S: Sensitive,R: Resistant, I: Intermediat

Identification Method: MOLDI TOF

Specimen submission time: 2015.1.25 Report Release Time: 2015.1.30 Laboratory Technician: Report Reviewer: Laboratory test order time: 2015.1.25 Ordering Physician:

**This report applies solely to the submitted specimen. For inquiries regarding test results, please contact the Laboratory prior to 17:00 on working days.**

**Name:** 　 **Gender: M**

**Age:** 85

**Department: Patient ID:**

**Inpatient No.:**

internal Medicine

**Bed No.:** 　 **Specimen Type: Blood**

**Clinical Diagnosis:**

**Sample No.:** **15003818**

**Result:** ***Candida parapsilosis***

Antibiotic Amphotericin B Fluconazole

Itraconazole

MIC AST Result Interpretation Breakpoint

<0.5 <1

<0.125

Voriconazole <0.062

Note: S: Sensitive,R: Resistant, I: Intermediat

Identification Method: MOLDI TOF

Specimen submission time: 2015.2.15

Laboratory test order time: 2015.2.15

Report Release Time: 2015.2.19 Laboratory Technician: Report Reviewer:

Ordering Physician:

**This report applies solely to the submitted specimen. For inquiries regarding test results, please contact the Laboratory prior to 17:00 on working days.**

**Name:**

**Gender:** **F**

**Age:** 64

**Department:**

**Patient ID:**

**Inpatient No.:**

surgical department

**Bed No.:**

**Specimen Type: Blood**

**Clinical Diagnosis:**

**Sample No.:** **15004951**

**Result:** ***Candida tropicalis***

Antibiotic MIC AST Result Interpretation Breakpoint

Amphotericin B >16 Fluconazole <1

Itraconazole >4

Voriconazole =0.25

Note: S: Sensitive,R: Resistant, I: Intermediat

Identification Method: MOLDI TOF

Specimen submission time: 2015.3.6

Laboratory test order time: 2015.3.5

Report Release Time: 2015.3.6 Laboratory Technician: Report Reviewer:

Ordering Physician:

**This report applies solely to the submitted specimen. For inquiries regarding test results, please contact the Laboratory prior to 17:00 on working days.**

**Name:** 　 **Gender: m**

**Age:** 73

**Department: Patient ID:**

**Inpatient No.:**

internal Medicine

**Bed No.:** 　 **Specimen Type: Blood**

**Clinical Diagnosis:**

**Sample No.:** **15008344**

**Result:** ***Candida tropicalis***

Antibiotic MIC AST Result Interpretation Breakpoint

Amphotericin B =1 Fluconazole <1

Itraconazole <0.125

Voriconazole <0.062

Note: S: Sensitive,R: Resistant, I: Intermediat

Identification Method: MOLDI TOF

Specimen submission time: 2015.4.18

Laboratory test order time: 2015.4.18

Report Release Time: 2015.4.18 Laboratory Technician: Report Reviewer:

Ordering Physician:

**This report applies solely to the submitted specimen. For inquiries regarding test results, please contact the Laboratory prior to 17:00 on working days.**

**Name:** 　 **Gender: m**

**Age:** 74

**Department: Patient ID:**

**Inpatient No.:**

internal Medicine

**Bed No.:** 　 **Specimen Type: Blood**

**Clinical Diagnosis:**

**Sample No.:** **15007377**

**Result:** ***Candida tropicalis***

Antibiotic MIC AST Result Interpretation Breakpoint

Amphotericin B =1 Fluconazole <1

Itraconazole <0.125

Voriconazole <0.062

Note: S: Sensitive,R: Resistant, I: Intermediat

Identification Method: MOLDI TOF

Specimen submission time: 2015.4.6

Laboratory test order time: 2015.4.6

Report Release Time: 2015.4.10 Laboratory Technician: Report Reviewer:

Ordering Physician:

**This report applies solely to the submitted specimen. For inquiries regarding test results, please contact the Laboratory prior to 17:00 on working days.**

**Name:** 　 **Gender: m**

**Age:** 86

**Department:** **NICU Patient ID:**

**Inpatient No.:**

**Bed No.:** 　 **Specimen Type: Blood**

**Clinical Diagnosis:**

**Sample No.:** **15009157**

**Result:** ***Candida parapsilosis***

Antibiotic MIC AST Result Interpretation Breakpoint

Amphotericin B <0.5 Fluconazole <1

Itraconazole <0.125

Voriconazole <0.062

Note: S: Sensitive,R: Resistant, I: Intermediat

Identification Method: MOLDI TOF

Specimen submission time: 2015.4.30

Laboratory test order time: 2015.4.30

Report Release Time: 2015.4.30 Laboratory Technician: Report Reviewer:

Ordering Physician:

**This report applies solely to the submitted specimen. For inquiries regarding test results, please contact the Laboratory prior to 17:00 on working days.**

**Name:** 　 **Gender: m**

**Age:** 86

**Department:** **NICU Patient ID:**

**Inpatient No.:**

**Bed No.:** 　 **Specimen Type: Blood**

**Clinical Diagnosis:**

**Sample No.:** **18009002**

**Result:** ***Candida parapsilosis***

Antibiotic MIC AST Result Interpretation Breakpoint

Amphotericin B <0.5 Fluconazole <1

Itraconazole <0.125

Voriconazole <0.062

Note: S: Sensitive,R: Resistant, I: Intermediat

Identification Method: MOLDI TOF

Specimen submission time: 2018.4.28

Laboratory test order time: 2018.4.28

Report Release Time: 2018.4.28 Laboratory Technician: Report Reviewer:

Ordering Physician:

**This report applies solely to the submitted specimen. For inquiries regarding test results, please contact the Laboratory prior to 17:00 on working days.**

**Name:** 　 **Gender: m**

**Age:** 86

**Department:** N**ICU Patient ID:**

**Inpatient No.:**

**\**

**Bed No.:** 　 **Specimen Type: Blood**

**Clinical Diagnosis:**

**Sample No.:** **16009158**

**Result:** ***Candida parapsilosis***

Antibiotic MIC AST Result Interpretation Breakpoint

Amphotericin B <0.5 Fluconazole <1

Itraconazole <0.125

Voriconazole <0.062

Note: S: Sensitive,R: Resistant, I: Intermediat

Identification Method: MOLDI TOF

Specimen submission time: 2016.4.30

Laboratory test order time: 2016.4.30

Report Release Time: 2016.5.4 Laboratory Technician: Report Reviewer:

Ordering Physician: xxx

**This report applies solely to the submitted specimen. For inquiries regarding test results, please contact the Laboratory prior to 17:00 on working days.**

**Name:**

**Gender:** **m**

**Age:** 57

**Department:**

**Patient ID:**

**Inpatient No.:**

surgical department

**Bed No.:**

**Specimen Type: Blood**

**Clinical Diagnosis:**

**Sample No.:** **16014586**

**Result:** ***Candida dubliniensis***

Antibiotic MIC AST Result Interpretation Breakpoint

Amphotericin B =8 Fluconazole <1

Itraconazole <0.125

Voriconazole <0.062

Note: S: Sensitive,R: Resistant, I: Intermediat

Identification Method: MOLDI TOF

Specimen submission time: 2016.7.22

Laboratory test order time: 2016.7.22

Report Release Time: 2016.7.29 Laboratory Technician: Report Reviewer:

Ordering Physician: xxx

**This report applies solely to the submitted specimen. For inquiries regarding test results, please contact the Laboratory prior to 17:00 on working days.**

**Name:**

**Gender:** **f**

**Age:** 50

**Department:**

**Patient ID:**

**Inpatient No.:**

emergency department

**Bed No.:**

**Specimen Type: Blood**

**Clinical Diagnosis:**

**Sample No.:** **16016119**

**Result:** ***Candida parapsilosis***

Antibiotic MIC AST Result Interpretation Breakpoint

Amphotericin B <0.5 Fluconazole <1

Itraconazole <0.125

Voriconazole <0.062

Note: S: Sensitive,R: Resistant, I: Intermediat

Identification Method: MOLDI TOF

Specimen submission time: 2016.8.9

Laboratory test order time: 2016.8.9

Report Release Time: 2016.8.14 Laboratory Technician: Report Reviewer:

Ordering Physician:

**This report applies solely to the submitted specimen. For inquiries regarding test results, please contact the Laboratory prior to 17:00 on working days.**

**Name:** 　 **Gender:** **f**

**Age:** 69

**Department:** EICU **Patient ID:**

**Inpatient No.:**

**Bed No.:** 　 **Specimen Type: Blood**

**Clinical Diagnosis:**

**Sample No.:** **16016936**

**Result:** ***Candida famata***

Antibiotic MIC AST Result Interpretation Breakpoint

Amphotericin B <0.5 Fluconazole <1

Itraconazole <0.125

Voriconazole <0.062

Note: S: Sensitive,R: Resistant, I: Intermediat

Identification Method: MOLDI TOF

Specimen submission time: 2016.8.18

Laboratory test order time: 2016.8.18

Report Release Time: 2016.8.22 Laboratory Technician: Report Reviewer:

Ordering Physician：xxx

**This report applies solely to the submitted specimen. For inquiries regarding test results, please contact the Laboratory prior to 17:00 on working days.**

**Name:** 　 **Gender: m**

**Age:** 73

**Department:** ICU **Patient ID:**

**Inpatient No.:**

**Bed No.:** 　 **Specimen Type: Blood**

**Clinical Diagnosis:**

**Sample No.:** **18018447**

**Result:** ***Candida glabrata***

Antibiotic MIC AST Result Interpretation Breakpoint

Amphotericin B <0.5 Fluconazole =2

Itraconazole =0.25

Voriconazole =0.125

Note: S: Sensitive,R: Resistant, I: Intermediat

Identification Method: MOLDI TOF

Specimen submission time: 2018.9.6

Laboratory test order time: 2018.9.6

Report Release Time: 2018.9.11 Laboratory Technician: Report Reviewer:

Ordering Physician: xxx

**This report applies solely to the submitted specimen. For inquiries regarding test results, please contact the Laboratory prior to 17:00 on working days.**

**Name:** 　 **Gender: m**

**Age:** 73

**Department:** **ICU Patient ID:**

**Inpatient No.:**

**Bed No.:** 　 **Specimen Type: Blood**

**Clinical Diagnosis:**

**Sample No.:** **16018572**

**Result:** ***Candida glabrata***

Antibiotic MIC AST Result Interpretation Breakpoint

Amphotericin B <0.5 Fluconazole =2

Itraconazole =0.25

Voriconazole =0.125

Note: S: Sensitive,R: Resistant, I: Intermediat

Identification Method: MOLDI TOF

Specimen submission time: 2016.9.7

Laboratory test order time: 2016.9.7

Report Release Time: 2016.9.12 Laboratory Technician: Report Reviewer:

Ordering Physician:

**This report applies solely to the submitted specimen. For inquiries regarding test results, please contact the Laboratory prior to 17:00 on working days.**

**Name:**

**Gender:** **m**

**Age:** 61

**Department:**

**Patient ID:**

**Inpatient No.:**

surgical department

**Bed No.:**

**Specimen Type: Blood**

**Clinical Diagnosis:**

**Sample No.:** **16019905**

**Result:** ***Candida albicans***

Antibiotic MIC AST Result Interpretation Breakpoint

Amphotericin B <0.5 Fluconazole <1

Itraconazole <0.125

Voriconazole <0.062

Note: S: Sensitive,R: Resistant, I: Intermediat

Identification Method: MOLDI TOF

Specimen submission time:2016.09.23 15:32Report Release Time: 2016.09.28 10:29 Laboratory Technician: Report Reviewer: Laboratory test order time: 2016.09.23.12:44 Ordering Physician: xxx

**This report applies solely to the submitted specimen. For inquiries regarding test results, please contact the Laboratory prior to 17:00 on working days.**

**Name:** 　 **Gender:** **f**

**Age:** 61

**Department: Patient ID:**

**Inpatient No.:**

gynecology

**Bed No.:** 　 **Specimen Type: Blood**

**Clinical Diagnosis:**

**Sample No.:** **16019828**

**Result:** ***Candida albicans***

Antibiotic MIC AST Result Interpretation Breakpoint

Amphotericin B <0.5 Fluconazole <1

Itraconazole <0.125

Voriconazole <0.062

Note: S: Sensitive,R: Resistant, I: Intermediat

Identification Method: MOLDI TOF

Specimen submission time:2016.09.22 20:55Report Release Time: 2016.09.28 10:30 Laboratory Technician: Report Reviewer: Laboratory test order time: 2016.09.22.16:23 Ordering Physician:xxx

**This report applies solely to the submitted specimen. For inquiries regarding test results, please contact the Laboratory prior to 17:00 on working days.**

**Name:**

**Gender:** **m**

**Age:** 30

**Department:**

**Patient ID:**

**Inpatient No.:**

surgical department

**Bed No.:**

**Specimen Type: Blood**

**Clinical Diagnosis:**

**Sample No.:** **18297723**

**Result:** ***Candida parapsilosis***

Antibiotic MIC AST Result Interpretation Breakpoint

Amphotericin B <0.5 Fluconazole <1

Itraconazole <0.125

Voriconazole <0.062

Note: S: Sensitive,R: Resistant, I: Intermediat

Identification Method: MOLDI TOF

Specimen submission time:2018.10.12 10:50Report Release Time: 2018.10.16 09:05 Laboratory Technician: Report Reviewer: Laboratory test order time: 2018.10.12.09:19 Ordering Physician:xxx

**This report applies solely to the submitted specimen. For inquiries regarding test results, please contact the Laboratory prior to 17:00 on working days.**

**Name:**

**Gender:** **m**

**Age:** 65

**Department:**

**Patient ID:**

**Inpatient No.:**

day-care unit

**Bed No.:**

**Specimen Type: Blood**

**Clinical Diagnosis:**

**Sample No.:** **16298226**

**Result:** ***Candida guilliermondii***

Antibiotic MIC AST Result Interpretation Breakpoint

Amphotericin B <0.5 Fluconazole =2

Itraconazole <0.125

Voriconazole <0.062

Note: S: Sensitive,R: Resistant, I: Intermediat

Identification Method: MOLDI TOF

Specimen submission time:2016.10.18 09:49Report Release Time: 2016.10.24 08:28 Laboratory Technician: Report Reviewer: Laboratory test order time: 2016.10.18.09:41 Ordering Physician: xxx

**This report applies solely to the submitted specimen. For inquiries regarding test results, please contact the Laboratory prior to 17:00 on working days.**

**Name:**

**Gender:** **m**

**Age:** 58

**Department:**

**Patient ID:**

**Inpatient No.:**

emergency department

**Bed No.:**

**Specimen Type: Blood**

**Clinical Diagnosis:**

**Sample No.:** **18299696**

**Result:** ***Candida glabrata***

Antibiotic MIC AST Result Interpretation Breakpoint

Amphotericin B <0.5 Fluconazole <1

Itraconazole <0.125

Voriconazole <0.062

Note: S: Sensitive,R: Resistant, I: Intermediat

Identification Method: MOLDI TOF

Specimen submission time:2018.11.06 07:40Report Release Time: 2018.11.09 09:14 Laboratory Technician: Report Reviewer: Laboratory test order time: 2018.11.04.20:05 Ordering Physician: xxx

**This report applies solely to the submitted specimen. For inquiries regarding test results, please contact the Laboratory prior to 17:00 on working days.**

**Name:** 　 **Gender: m**

**Age:** 58

**Department:** **ICU Patient ID:**

**Inpatient No.:**

**Bed No.:** 　 **Specimen Type: Blood**

**Clinical Diagnosis:**

**Sample No.:** **16300014**

**Result:** ***Candida glabrata***

Antibiotic MIC AST Result Interpretation Breakpoint

Amphotericin B <0.5 Fluconazole =4

Itraconazole =0.5

Voriconazole =0.25

Note: S: Sensitive,R: Resistant, I: Intermediat

Identification Method: MOLDI TOF

Specimen submission time:2016.11.10 11:43Report Release Time: 2016.11.13 12:09 Laboratory Technician: Report Reviewer: Laboratory test order time: 2016.11.10.09:06 Ordering Physician:xxx

**This report applies solely to the submitted specimen. For inquiries regarding test results, please contact the Laboratory prior to 17:00 on working days.**

**Name:** 　 **Gender: m**

**Age:** 78

**Department:** N**ICU Patient ID:**

**Inpatient No.:**

**Bed No.:** 　 **Specimen Type: Blood**

**Clinical Diagnosis:**

**Sample No.:** **16600260**

**Result:** ***Candida parapsilosis***

Antibiotic MIC AST Result Interpretation Breakpoint

Amphotericin B <0.5 Fluconazole <1

Itraconazole <0.125

Voriconazole <0.062

Note: S: Sensitive,R: Resistant, I: Intermediat

Identification Method: MOLDI TOF

Specimen submission time:2016.11.13 12:09Report Release Time: 2016.11.17 09:22 Laboratory Technician: Report Reviewer: Laboratory test order time: 2016.11.13.09:32 Ordering Physician: xxx

**This report applies solely to the submitted specimen. For inquiries regarding test results, please contact the Laboratory prior to 17:00 on working days.**

**Name:** 　 **Gender: M**

**Age:** 78

**Department:** **NICU Patient ID:**

**Inpatient No.:**

**Bed No.:** 　 **Specimen Type:Blood**

**Clinical Diagnosis:**

**Sample No.:** **16300261**

**Result:** ***Candida parapsilosis***

Antibiotic MIC AST Result Interpretation Breakpoint

Amphotericin B <0.5 Fluconazole <1

Itraconazole <0.125

Voriconazole <0.062

Note: S: Sensitive,R: Resistant, I: Intermediat

Identification Method: MOLDI TOF

Specimen submission time:2016.11.13 12:10Report Release Time: 2016.11.16 09:14 Laboratory Technician: Report Reviewer: Laboratory test order time: 2016.11.13.09:33 Ordering Physician:xxx

**This report applies solely to the submitted specimen. For inquiries regarding test results, please contact the Laboratory prior to 17:00 on working days.**

**Name:** 　 **Gender: M**

**Age:** 73

**Department: Patient ID:**

**Inpatient No.:**

internal Medicine

**Bed No.:** 　 **Specimen Type: Blood**

**Clinical Diagnosis:**

**Sample No.:** **210310523**

**Result:** ***Candida famata***

Antibiotic

　Amphotericin B

Fluconazole

MIC AST Result Interpretation

=1

<1

Breakpoint

Itraconazole <0.12

Voriconazole <0.06

Note: S: Sensitive,R: Resistant, I: Intermediat

Identification Method: MOLDI TOF

Specimen submission time:2021.03.10 10:29Report Release Time: 2021.03.14 09:26 Laboratory Technician: Report Reviewer: Laboratory test order time: 2021.03.10.09:11 Ordering Physician:xxx

**This report applies solely to the submitted specimen. For inquiries regarding test results, please contact the Laboratory prior to 17:00 on working days.**

**Name:**

**Gender:** **M**

**Age:** 27

**Department:**

**Patient ID:**

**Inpatient No.:**

emergency department

**Bed No.:**

**Specimen Type: Blood**

**Clinical Diagnosis:**

**Sample No.:** **18301786**

**Result:** ***Candida parapsilosis***

Antibiotic MIC AST Result Interpretation Breakpoint

Amphotericin B <0.5 Fluconazole <1

Itraconazole <0.125

Voriconazole <0.062

Note: S: Sensitive,R: Resistant, I: Intermediat

Identification Method: MOLDI TOF

Specimen submission time:2018.12.03 13:55Report Release Time: 2018.12.06 09:29 Laboratory Technician: Report Reviewer: Laboratory test order time: 2018.12.03.07:48 Ordering Physician:xxx

**This report applies solely to the submitted specimen. For inquiries regarding test results, please contact the Laboratory prior to 17:00 on working days.**

**Name:** 　 **Gender:** **F**

**Age:** 31

**Department: Patient ID:**

**Inpatient No.:**

internal Medicine

**Bed No.:** 　 **Specimen Type: Blood**

**Clinical Diagnosis:**

**Sample No.:** **18302119**

**Result:** ***Candida tropicalis***

Antibiotic MIC AST Result Interpretation Breakpoint

Amphotericin B <0.5 Fluconazole <1

Itraconazole =0.25

Voriconazole =0.25

Note: S: Sensitive,R: Resistant, I: Intermediat

Identification Method: MOLDI TOF

Specimen submission time:2018.12.08 05:31Report Release Time: 2018.12.11 08:54 Laboratory Technician: Report Reviewer: Laboratory test order time: 2018.12.08.03:57 Ordering Physician:xxx

**This report applies solely to the submitted specimen. For inquiries regarding test results, please contact the Laboratory prior to 17:00 on working days.**

**Name:**

**Gender:** **F**

**Age:** 74

**Department:**

**Patient ID:**

**Inpatient No.:**

Surgical Depatment

**Bed No.:**

**Specimen Type: Blood**

**Clinical Diagnosis:**

**Sample No.:** **18304781**

**Result:** ***Candida krusei***

Antibiotic MIC AST Result Interpretation Breakpoint

Amphotericin B =1 Fluconazole =32

Itraconazole =1

Voriconazole =1

Note: S: Sensitive,R: Resistant, I: Intermediat

Identification Method: MOLDI TOF

Specimen submission time:2018.01.08 20:48Report Release Time: 2018.01.12 08:50 Laboratory Technician: Report Reviewer: Laboratory test order time: 2018.01.08.19:07 Ordering Physician:xxx

**This report applies solely to the submitted specimen. For inquiries regarding test results, please contact the Laboratory prior to 17:00 on working days.**

**Name:**

**Gender:** **M**

**Age:** 27

**Department:**

**Patient ID:**

**Inpatient No.:**

emergency department

**Bed No.:**

**Specimen Type: Blood**

**Clinical Diagnosis:**

**Sample No.:** **18304912**

**Result:** ***Candida parapsilosis***

Antibiotic MIC AST Result Interpretation Breakpoint

Amphotericin B <0.5 Fluconazole <1

Itraconazole <0.125

Voriconazole <0.062

Note: S: Sensitive,R: Resistant, I: Intermediat

Identification Method: MOLDI TOF

Specimen submission time:2018.01.10 18:32Report Release Time: 2018.01.13 09:16 Laboratory Technician: Report Reviewer: Laboratory test order time: 2018.01.10.15:46 Ordering Physician:xxx

**This report applies solely to the submitted specimen. For inquiries regarding test results, please contact the Laboratory prior to 17:00 on working days.**

**Name:** 　 **Gender:** **F**

**Age:** 1M

**Department: Patient ID:**

**Inpatient No.:**

Neonatology

**Bed No.:** 　 **Specimen Type: Blood**

**Clinical Diagnosis:**

**Sample No.:** **16308938**

**Result:** ***Candida glabrata***

Antibiotic MIC AST Result Interpretation Breakpoint

Amphotericin B <0.5 Fluconazole =2

Itraconazole =0.25

Voriconazole <0.062

Note: S: Sensitive,R: Resistant, I: Intermediat

Identification Method: MOLDI TOF

Specimen submission time:2016.03.03 13:41Report Release Time: 2016.03.08 09:23 Laboratory Technician: Report Reviewer: Laboratory test order time: 2016.03.03.12:45 Ordering Physician:xxx

**This report applies solely to the submitted specimen. For inquiries regarding test results, please contact the Laboratory prior to 17:00 on working days.**

**Name:** 　 **Gender:** **F**

**Age:** 1M

**Department: Patient ID:**

**Inpatient No.:**

Neonatology

**Bed No.:** 　 **Specimen Type: Blood**

**Clinical Diagnosis:**

**Sample No.:** **16309155**

**Result:** ***Candida glabrata***

Antibiotic MIC AST Result Interpretation Breakpoint

Amphotericin B <0.5 Fluconazole =2

Itraconazole =0.25

Voriconazole <0.062

Note: S: Sensitive,R: Resistant, I: Intermediat

Identification Method: MOLDI TOF

Specimen submission time:2016.03.06 16:28Report Release Time: 2016.03.10 09:10 Laboratory Technician: Report Reviewer: Laboratory test order time: 2016.03.06.10:44 Ordering Physician:xxx

**This report applies solely to the submitted specimen. For inquiries regarding test results, please contact the Laboratory prior to 17:00 on working days.**

**Name:** 　 **Gender:** **f**

**Age:** 37

**Department:** **NICU Patient ID:**

**Inpatient No.:**

**Bed No.:** 　 **Specimen Type: Blood**

**Clinical Diagnosis:**

**Sample No.:** **16312508**

**Result:** ***Candida parapsilosis***

Antibiotic MIC AST Result Interpretation Breakpoint

Amphotericin B <0.5 Fluconazole <1

Itraconazole <0.125

Voriconazole <0.062

Note: S: Sensitive,R: Resistant, I: Intermediat

Identification Method: MOLDI TOF

Specimen submission time:2016.04.18 01:15Report Release Time: 2016.04.22 10:21 Laboratory Technician: Report Reviewer: Laboratory test order time: 2016.04.17.15:05 Ordering Physician:xxx

**This report applies solely to the submitted specimen. For inquiries regarding test results, please contact the Laboratory prior to 17:00 on working days.**

**Name:**

**Gender:** **m**

**Age:** 49

**Department:**

**Patient ID:**

**Inpatient No.:**

**Surgical Department**

**Bed No.:**

**Specimen Type: Blood**

**Clinical Diagnosis:**

**Sample No.:** **16313579**

**Result:** ***Candida parapsilosis***

Antibiotic MIC AST Result Interpretation Breakpoint

Amphotericin B <0.5 Fluconazole <1

Itraconazole <0.125

Voriconazole <0.062

Note: S: Sensitive,R: Resistant, I: Intermediat

Identification Method: MOLDI TOF

Specimen submission time:2016.04.29 20:02Report Release Time: 2016.05.05 09:25 Laboratory Technician: Report Reviewer: Laboratory test order time: 2016.04.29.18:06 Ordering Physician:xxx

**This report applies solely to the submitted specimen. For inquiries regarding test results, please contact the Laboratory prior to 17:00 on working days.**

**Name:**

**Gender:** **m**

**Age:** 29

**Department:**

**Patient ID:**

**Inpatient No.:**

**Internal Department**

**Bed No.:**

**Specimen Type: Blood**

**Clinical Diagnosis:**

**Sample No.:** **16317278**

**Result:** ***Candida albicans***

Antibiotic MIC AST Result Interpretation Breakpoint

Amphotericin B <0.5 Fluconazole <1

Itraconazole <0.125

Voriconazole <0.062

Note: S: Sensitive,R: Resistant, I: Intermediat

Identification Method: MOLDI TOF

Specimen submission time:2016.06.17 15:41Report Release Time: 2016.06.20 09:02 Laboratory Technician: Report Reviewer: Laboratory test order time: 2016.06.17.10:22 Ordering Physician:xxx

**This report applies solely to the submitted specimen. For inquiries regarding test results, please contact the Laboratory prior to 17:00 on working days.**

**Name:** 　 **Gender: M**

**Age:** 29

**Department:** **EICU Patient ID:**

**Inpatient No.:**

**Bed No.:** 　 **Specimen Type: Blood**

**Clinical Diagnosis:**

**Sample No.:** **16317280**

**Result:** ***Candida albicans***

Antibiotic MIC AST Result Interpretation Breakpoint

Amphotericin B <0.5 Fluconazole <1

Itraconazole <0.125

Voriconazole <0.062

Note: S: Sensitive,R: Resistant, I: Intermediat

Identification Method: MOLDI TOF

Specimen submission time:2016.06.17 15:41Report Release Time: 2016.06.20 10:24 Laboratory Technician: Report Reviewer: Laboratory test order time: 2016.06.17.10:07 Ordering Physician: xxx

**This report applies solely to the submitted specimen. For inquiries regarding test results, please contact the Laboratory prior to 17:00 on working days.**

**Name:** 　 **Gender: M**

**Age:** 28D

**Department: Patient ID:**

**Inpatient No.:**

Neonatology

**Bed No.:** 　 **Specimen Type: Blood**

**Clinical Diagnosis:**

**Sample No.:** **16318488**

**Result:** ***Candida albicans***

Antibiotic MIC AST Result Interpretation Breakpoint

Amphotericin B <0.5 Fluconazole =2

Itraconazole <0.125

Voriconazole =0.25

Note: S: Sensitive,R: Resistant, I: Intermediat

Identification Method: MOLDI TOF

Specimen submission time:2016.07.02 16:34Report Release Time: 2016.07.06 09:12 Laboratory Technician: Report Reviewer: Laboratory test order time: 2016.07.02.13:55 Ordering Physician:xxx

**This report applies solely to the submitted specimen. For inquiries regarding test results, please contact the Laboratory prior to 17:00 on working days.**

**Name:** 　 **Gender: M**

**Age:** 1M

**Department: Patient ID:**

**Inpatient No.:**

Neonatology

**Bed No.:** 　 **Specimen Type: Blood**

**Clinical Diagnosis:**

**Sample No.:** **18318988**

**Result:** ***Candida albicans***

Antibiotic MIC AST Result Interpretation Breakpoint

Amphotericin B <0.5 Fluconazole =2

Itraconazole >4

Voriconazole =0.25

Note: S: Sensitive,R: Resistant, I: Intermediat

Identification Method: MOLDI TOF

Specimen submission time:2018.07.08 14:19Report Release Time: 2018.07.12 09:01 Laboratory Technician: Report Reviewer: Laboratory test order time: 2018.07.08.13:03 Ordering Physician: xxx

**This report applies solely to the submitted specimen. For inquiries regarding test results, please contact the Laboratory prior to 17:00 on working days.**

**Name:** 　 **Gender: M**

**Age:** 59

**Department:** NICU **Patient ID:**

**Inpatient No.:**

**Bed No.:** 　 **Specimen Type: Blood**

**Clinical Diagnosis:**

**Sample No.:** **18319293**

**Result:** ***Candida parapsilosis***

Antibiotic MIC AST Result Interpretation Breakpoint

Amphotericin B <0.5 Fluconazole <1

Itraconazole <0.125

Voriconazole <0.062

Note: S: Sensitive,R: Resistant, I: Intermediat

Identification Method: MOLDI TOF

Specimen submission time:2018.07.12 21:12Report Release Time: 2018.07.15 09:45 Laboratory Technician: Report Reviewer: Laboratory test order time: 2018.07.12.12:44 Ordering Physician:xxx

**This report applies solely to the submitted specimen. For inquiries regarding test results, please contact the Laboratory prior to 17:00 on working days.**

**Name:** 　 **Gender: M**

**Age:** 59

**Department:** NICU **Patient ID:**

**Inpatient No.:**

**Bed No.:** 　 **Specimen Type: Blood**

**Clinical Diagnosis:**

**Sample No.:** **18319234**

**Result:** ***Candida parapsilosis***

Antibiotic MIC AST Result Interpretation Breakpoint

Amphotericin B <0.5 Fluconazole <1

Itraconazole <0.125

Voriconazole <0.062

Note: S: Sensitive,R: Resistant, I: Intermediat

Identification Method: MOLDI TOF

Specimen submission time:2018.07.12 08:09Report Release Time: 2018.07.15 09:45 Laboratory Technician: Report Reviewer: Laboratory test order time: 2018.07.12.06:28 Ordering Physician:xxx

**This report applies solely to the submitted specimen. For inquiries regarding test results, please contact the Laboratory prior to 17:00 on working days.**

**Name:**

**Gender:** **f**

**Age:** 47

**Department:**

**Patient ID:**

**Inpatient No.:**

surgical department

**Bed No.:**

**Specimen Type: Blood**

**Clinical Diagnosis:**

**Sample No.:** **18319486**

**Result:** ***Candida parapsilosis***

Antibiotic MIC AST Result Interpretation Breakpoint

Amphotericin B <0.5 Fluconazole <1

Itraconazole <0.125

Voriconazole <0.062

Note: S: Sensitive,R: Resistant, I: Intermediat

Identification Method: MOLDI TOF

Specimen submission time:2018.07.14 16:01Report Release Time: 2018.07.19 16:43 Laboratory Technician: Report Reviewer: Laboratory test order time: 2018.07.14.15:20 Ordering Physician:xxx

**This report applies solely to the submitted specimen. For inquiries regarding test results, please contact the Laboratory prior to 17:00 on working days.**

**Name:** 　 **Gender: m**

**Age:** 59

**Department:** **NICU Patient ID:**

**Inpatient No.:**

**Bed No.:** 　 **Specimen Type: Blood**

**Clinical Diagnosis:**

**Sample No.:** **18319595**

**Result:** ***Candida parapsilosis***

Antibiotic MIC AST Result Interpretation Breakpoint

Amphotericin B <0.5 Fluconazole <1

Itraconazole <0.125

Voriconazole <0.062

Note: S: Sensitive,R: Resistant, I: Intermediat

Identification Method: MOLDI TOF

Specimen submission time:2018.07.15 19:49Report Release Time: 2018.07.19 08:43 Laboratory Technician: Report Reviewer: Laboratory test order time: 2018.07.15.17:24 Ordering Physician:xxx

**This report applies solely to the submitted specimen. For inquiries regarding test results, please contact the Laboratory prior to 17:00 on working days.**

**Name:** 　 **Gender: m**

**Age:** 63

**Department:** ICU **Patient ID:**

**Inpatient No.:**

**Bed No.:** 　 **Specimen Type: Blood**

**Clinical Diagnosis:**

**Sample No.:** **18321265**

**Result:** ***Candida tropicalis***

Antibiotic MIC AST Result Interpretation Breakpoint

Amphotericin B <0.5 Fluconazole <1

Itraconazole <0.125

Voriconazole =0.125

Note: S: Sensitive,R: Resistant, I: Intermediat

Identification Method: MOLDI TOF

Specimen submission time:2018.08.04 16:16Report Release Time: 2018.08.08 10:40 Laboratory Technician: Report Reviewer: Laboratory test order time: 2018.08.04.11:25 Ordering Physician: xxx

**This report applies solely to the submitted specimen. For inquiries regarding test results, please contact the Laboratory prior to 17:00 on working days.**

**Name:** 　 **Gender: M**

**Age:** 9

**Department: Patient ID:**

**Inpatient No.:**

pediatrics

**Bed No.:** 　 **Specimen Type: Blood**

**Clinical Diagnosis:**

**Sample No.:** **18321494**

**Result:** ***Candida tropicalis***

Antibiotic MIC AST Result Interpretation Breakpoint

Amphotericin B <0.5 Fluconazole <1

Itraconazole <0.125

Voriconazole =0.25

Note: S: Sensitive,R: Resistant, I: Intermediat

Identification Method: MOLDI TOF

Specimen submission time:2018.08.08 07:55Report Release Time: 2018.08.11 10:25 Laboratory Technician: Report Reviewer: Laboratory test order time: 2018.08.08.05:56 Ordering Physician:xxx

**This report applies solely to the submitted specimen. For inquiries regarding test results, please contact the Laboratory prior to 17:00 on working days.**

**Name:** 　 **Gender: m**

**Age:** 9

**Department: Patient ID:**

**Inpatient No.:**

pediatrics

**Bed No.:** 　 **Specimen Type: Blood**

**Clinical Diagnosis:**

**Sample No.:** **18322035**

**Result:** ***Candida tropicalis***

Antibiotic MIC AST Result Interpretation Breakpoint

Amphotericin B <0.5 Fluconazole <1

Itraconazole <0.125

Voriconazole =0.025

Note: S: Sensitive,R: Resistant, I: Intermediat

Identification Method: MOLDI TOF

Specimen submission time:2018.08.13 10:35Report Release Time: 2018.08.16 09:19 Laboratory Technician: Report Reviewer: Laboratory test order time: 2018.08.13.08:06 Ordering Physician:xxx

**This report applies solely to the submitted specimen. For inquiries regarding test results, please contact the Laboratory prior to 17:00 on working days.**

**Name:**

**Gender:** **m**

**Age:** 52

**Department:**

**Patient ID:**

**Inpatient No.:**

surgical department

**Bed No.:**

**Specimen Type: Blood**

**Clinical Diagnosis:**

**Sample No.:** **18322055**

**Result:** ***Candida parapsilosis***

Antibiotic MIC AST Result Interpretation Breakpoint

Amphotericin B <0.5 Fluconazole <1

Itraconazole <0.125

Voriconazole <0.062

Note: S: Sensitive,R: Resistant, I: Intermediat

Identification Method: MOLDI TOF

Specimen submission time:2018.08.14 05:23Report Release Time: 2018.08.17 09:53 Laboratory Technician: Report Reviewer: Laboratory test order time: 2018.08.14.02:01 Ordering Physician:xxx

**This report applies solely to the submitted specimen. For inquiries regarding test results, please contact the Laboratory prior to 17:00 on working days.**

**Name:**

**Gender:** **F**

**Age:** 41

**Department:**

**Patient ID:**

**Inpatient No.:**

surgical department

**Bed No.:**

**Specimen Type: Blood**

**Clinical Diagnosis:**

**Sample No.:** **18323625**

**Result:** ***Candida parapsilosis***

Antibiotic MIC AST Result Interpretation Breakpoint

Amphotericin B <0.5 Fluconazole <1

Itraconazole <0.125

Voriconazole <0.062

Note: S: Sensitive,R: Resistant, I: Intermediat

Identification Method: MOLDI TOF

Specimen submission time:2018.09.01 21:42Report Release Time: 2018.09.05 10:05 Laboratory Technician: Report Reviewer: Laboratory test order time: 2018.09.01.18:53 Ordering Physician:xxx

**This report applies solely to the submitted specimen. For inquiries regarding test results, please contact the Laboratory prior to 17:00 on working days.**

**Name:** 　 **Gender:** **f**

**Age:** 41

**Department: Patient ID:**

**Inpatient No.:**

internal Medicine

**Bed No.:** 　 **Specimen Type: Blood**

**Clinical Diagnosis:**

**Sample No.:** **18322945**

**Result:** ***Candida parapsilosis***

Antibiotic MIC AST Result Interpretation Breakpoint

Amphotericin B <0.5 Fluconazole <1

Itraconazole <0.125

Voriconazole <0.062

Note: S: Sensitive,R: Resistant, I: Intermediat

Identification Method: MOLDI TOF

Specimen submission time:2018.08.24 14:25Report Release Time: 2018.08.29 10:38 Laboratory Technician: Report Reviewer: Laboratory test order time: 2018.08.24.10:25 Ordering Physician:xxx

**This report applies solely to the submitted specimen. For inquiries regarding test results, please contact the Laboratory prior to 17:00 on working days.**

**Name:**

**Gender:** **m**

**Age:** 78

**Department:**

**Patient ID:**

**Inpatient No.:**

**Surgical Department**

**Bed No.:**

**Specimen Type: Blood**

**Clinical Diagnosis:**

**Sample No.:** **18323882**

**Result:** ***Candida tropicalis***

Antibiotic MIC AST Result Interpretation Breakpoint

Amphotericin B <0.5 Fluconazole <1

Itraconazole =25

Voriconazole =0.125

Note: S: Sensitive,R: Resistant, I: Intermediat

Identification Method: MOLDI TOF

Specimen submission time: 2018.09.05 21:09 Report Release Time: 2018.09.09 08:32 Laboratory Technician: Report Reviewer: Laboratory test order time: 2018.09.05 20:02 Ordering Physician: xxx

**This report applies solely to the submitted specimen. For inquiries regarding test results, please contact the Laboratory prior to 17:00 on working days.**

**Name:**

**Gender:** **f**

**Age:** 81

**Department:**

**Patient ID:**

**Inpatient No.:**

**Internal Department**

**Bed No.:**

**Specimen Type: Blood**

**Clinical Diagnosis:**

**Sample No.:** **18326554**

**Result:** ***Candida albicans***

Antibiotic MIC AST Result Interpretation Breakpoint

Amphotericin B <0.5 Fluconazole <1

Itraconazole <0.125

Voriconazole =0.125

Note: S: Sensitive,R: Resistant, I: Intermediat

Identification Method: MOLDI TOF

Specimen submission time: 2018.10.11 23:10 Report Release Time: 2018.10.15 10:11 Laboratory Technician: Report Reviewer: Laboratory test order time: 2018.10.11 13:45 Ordering Physician: xxx

**This report applies solely to the submitted specimen. For inquiries regarding test results, please contact the Laboratory prior to 17:00 on working days.**

**Name:** 　 **Gender: m**

**Age:** 67

**Department:** **ICU Patient ID:**

**Inpatient No.:**

**Bed No.:** 　 **Specimen Type: Blood**

**Clinical Diagnosis:**

**Sample No.:** **18326727**

**Result:** ***Candida parapsilosis***

Antibiotic MIC AST Result Interpretation Breakpoint

Amphotericin B <0.5 Fluconazole <1

Itraconazole <0.125

Voriconazole <0.062

Note: S: Sensitive,R: Resistant, I: Intermediat

Identification Method: MOLDI TOF

Specimen submission time: 2018.10.13 13:25 Report Release Time:2018.10.17 09:47 Laboratory Technician: Report Reviewer: Laboratory test order time: 2018.10.13 09:12 Ordering Physician: xxx

**This report applies solely to the submitted specimen. For inquiries regarding test results, please contact the Laboratory prior to 17:00 on working days.**

**Name:**

**Gender:** **m**

**Age:** 62

**Department:**

**Patient ID:**

**Inpatient No.:**

**Surgical Department**

**Bed No.:**

**Specimen Type: Blood**

**Clinical Diagnosis:**

**Sample No.:** **17326986**

**Result:** ***Candida tropicalis***

Antibiotic MIC AST Result Interpretation Breakpoint

Amphotericin B <0.5 Fluconazole >128

Itraconazole >4

Voriconazole =1

Note: S: Sensitive,R: Resistant, I: Intermediat

Identification Method: MOLDI TOF

Specimen submission time:2017.10.16 15 :36 Report Release Time:2017.10.19 07:51 Laboratory Technician: Report Reviewer: Laboratory test order time: 2017.10.16 12:34 Ordering Physician: xxx

**This report applies solely to the submitted specimen. For inquiries regarding test results, please contact the Laboratory prior to 17:00 on working days.**

**Name:** 　 **Gender: m**

**Age:** 62

**Department:** **ICU Patient ID:**

**Inpatient No.:**

**Bed No.:** 　 **Specimen Type: Blood**

**Clinical Diagnosis:**

**Sample No.:** **17327158**

**Result:** ***Candida tropicalis***

Antibiotic MIC AST Result Interpretation Breakpoint

Amphotericin B <0.5 Fluconazole <1

Itraconazole >4

Voriconazole =1

Note: S: Sensitive,R: Resistant, I: Intermediat

Identification Method: MOLDI TOF

Specimen submission time: 2017.10.16 15:36 Report Release Time: 2017.10.19 07:51 Laboratory Technician: Report Reviewer: Laboratory test order time: 2017.10.16 12:34 Ordering Physician:xxx

**This report applies solely to the submitted specimen. For inquiries regarding test results, please contact the Laboratory prior to 17:00 on working days.**

**Name:** 　 **Gender: m**

**Age:** 88

**Department: Patient ID:**

**Inpatient No.:**

internal Medicine

**Bed No.:** 　 **Specimen Type: Blood**

**Clinical Diagnosis:**

**Sample No.:** **17330732**

**Result:** ***Candida parapsilosis***

Antibiotic MIC AST Result Interpretation Breakpoint

Amphotericin B <0.5 Fluconazole <1

Itraconazole <0.125

Voriconazole <0.062

Note: S: Sensitive,R: Resistant, I: Intermediat

Identification Method: MOLDI TOF

Specimen submission time: 2017.11.29 12:37 Report Release Time: 2017.12.05 08:36 Laboratory Technician: Report Reviewer: Laboratory test order time: 2017.11.29 12:01 Ordering Physician:xxx

**This report applies solely to the submitted specimen. For inquiries regarding test results, please contact the Laboratory prior to 17:00 on working days.**

**Name:** 　 **Gender: m**

**Age:** 67

**Department:** **ICU Patient ID:**

**Inpatient No.:**

**Bed No.:** 　 **Specimen Type: Blood**

**Clinical Diagnosis:**

**Sample No.:** **17327441**

**Result:** ***Candida parapsilosis***

Antibiotic MIC AST Result Interpretation Breakpoint

Amphotericin B <0.5 Fluconazole <1

Itraconazole <0.125

Voriconazole <0.062

Note: S: Sensitive,R: Resistant, I: Intermediat

Identification Method: MOLDI TOF

Specimen submission time:2017.10.21 20:09 Report Release Time: 2017.10.27 08:35 Laboratory Technician: Report Reviewer: Laboratory test order time: 2017.10.21 19:20 Ordering Physician: xxx

**This report applies solely to the submitted specimen. For inquiries regarding test results, please contact the Laboratory prior to 17:00 on working days.**

**Name:** 　 **Gender:** **f**

**Age:** 31

**Department:** **ICU Patient ID:**

**Inpatient No.:**

**Bed No.:** 　 **Specimen Type: Blood**

**Clinical Diagnosis:**

**Sample No.:** **17727926**

**Result:** ***Candida albicans***

Antibiotic MIC AST Result Interpretation Breakpoint

Amphotericin B <0.5 Fluconazole <1

Itraconazole <0.125

Voriconazole <0.062

Note: S: Sensitive,R: Resistant, I: Intermediat

Identification Method: MOLDI TOF

Specimen submission time: 2017.10.27 14:40 Report Release Time: 2017.10.31 09:56 Laboratory Technician: Report Reviewer: Laboratory test order time: 2017.10.27 13:47 Ordering Physician:xxx

**This report applies solely to the submitted specimen. For inquiries regarding test results, please contact the Laboratory prior to 17:00 on working days.**

**Name:**

**Gender:** **m**

**Age:** 59

**Department:**

**Patient ID:**

**Inpatient No.:**

emergency department

**Bed No.:**

**Specimen Type: Blood**

**Clinical Diagnosis:**

**Sample No.:** **19328018**

**Result:** ***Candida glabrata***

Antibiotic MIC

Amphotericin B <0.5 Fluconazole <1

Itraconazole <0.125

Voriconazole <0.062

AST Result Interpretation Breakpoint

Note: S: Sensitive,R: Resistant, I: Intermediat

Identification Method: MOLDI TOF

Specimen submission time:2019.10.28 12:07 Report Release Time: 2019.11.04 08:01 Laboratory Technician: Report Reviewer: Laboratory test order time:2019.10.27 17:03 Ordering Physician:xxx

**This report applies solely to the submitted specimen. For inquiries regarding test results, please contact the Laboratory prior to 17:00 on working days.**

**Name:** 　 **Gender: m**

**Age:** 88

**Department: Patient ID:**

**Inpatient No.:**

internal Medicine

**Bed No.:** 　 **Specimen Type: Blood**

**Clinical Diagnosis:**

**Sample No.:** **19333240**

**Result:** ***Candida parapsilosis***

Antibiotic MIC AST Result Interpretation Breakpoint

Amphotericin B <0.5 Fluconazole <1

Itraconazole <0.125

Voriconazole <0.062

Note: S: Sensitive,R: Resistant, I: Intermediat

Identification Method: MOLDI TOF

Specimen submission time: 2019.12.28 08:01 Report Release Time:2019.12.31 10:35Laboratory Technician: Report Reviewer: Laboratory test order time: 2019.12.28 01:23 Ordering Physician:xxx

**This report applies solely to the submitted specimen. For inquiries regarding test results, please contact the Laboratory prior to 17:00 on working days.**

**Name:**

**Gender:** **m**

**Age:** 88

**Department:**

**Patient ID:**

**Inpatient No.:**

surgical department

**Bed No.:**

**Specimen Type: Blood**

**Clinical Diagnosis:**

**Sample No.:** **19334587**

**Result:** ***Candida parapsilosis***

Antibiotic MIC AST Result Interpretation Breakpoint

Amphotericin B <0.5 Fluconazole <1

Itraconazole <0.125

Voriconazole <0.062

Note: S: Sensitive,R: Resistant, I: Intermediat

Identification Method: MOLDI TOF

Specimen submission time: 2019.01.12 15:12 Report Release Time:2019.01.16 11:50 Laboratory Technician: Report Reviewer: Laboratory test order time: 2019.01 12 14:24 Ordering Physician:xxx

**This report applies solely to the submitted specimen. For inquiries regarding test results, please contact the Laboratory prior to 17:00 on working days.**

**Name:** 　 **Gender:** **f**

**Age:** 34

**Department:** **ICU Patient ID:**

**Inpatient No.:**

**Bed No.:** 　 **Specimen Type: Blood**

**Clinical Diagnosis:**

**Sample No.:** **19331530**

**Result:** ***Candida albicans***

Antibiotic MIC AST Result Interpretation Breakpoint

Amphotericin B <0.5 Fluconazole <1.0

Itraconazole <0.125

Voriconazole <0.062

Note: S: Sensitive,R: Resistant, I: Intermediat

Identification Method: MOLDI TOF

Specimen submission time: 2019.12.08 20:17 Report Release Time: 2019.12.11 10:24 Laboratory Technician: Report Reviewer: Laboratory test order time: 2019.12.08 15:07 Ordering Physician:xxx

**This report applies solely to the submitted specimen. For inquiries regarding test results, please contact the Laboratory prior to 17:00 on working days.**

**Name:** 　 **Gender:** **f**

**Age:** 34

**Department:** **ICU Patient ID:**

**Inpatient No.:**

**Bed No.:** 　 **Specimen Type: Blood**

**Clinical Diagnosis:**

**Sample No.:** **19331534**

**Result:** ***Candida albicans***

Antibiotic MIC AST Result Interpretation Breakpoint

Amphotericin B <0.5 Fluconazole <1.0

Itraconazole <0.125

Voriconazole <0.062

Note: S: Sensitive,R: Resistant, I: Intermediat

Identification Method: MOLDI TOF

Specimen submission time: 2019.12.09 07:28 Report Release Time: 2019.12.12 08:30 Laboratory Technician: Report Reviewer: Laboratory test order time: 2019.12.08 17:44 Ordering Physician:xxx

**This report applies solely to the submitted specimen. For inquiries regarding test results, please contact the Laboratory prior to 17:00 on working days.**

**Name:** 　 **Gender:** **f**

**Age:** 34

**Department:** **ICU Patient ID:**

**Inpatient No.:**

**Bed No.:** 　 **Specimen Type: Blood**

**Clinical Diagnosis:**

**Sample No.:** **20331537**

**Result:** ***Candida albicans***

Antibiotic MIC AST Result Interpretation Breakpoint

Amphotericin B <0.5 Fluconazole <1.0

Itraconazole <0.125

Voriconazole <0.062

Note: S: Sensitive,R: Resistant, I: Intermediat

Identification Method: MOLDI TOF

Specimen submission time:2020.12.09 07:29 Report Release Time:2020.12.12.08:33 Laboratory Technician: Report Reviewer: Laboratory test order time:2020.12.08 22:43 Ordering Physician:xxx

**This report applies solely to the submitted specimen. For inquiries regarding test results, please contact the Laboratory prior to 17:00 on working days.**

**Name:** 　 **Gender:** **f**

**Age:** 75

**Department:** **ICU Patient ID:**

**Inpatient No.:**

**Bed No.:** 　 **Specimen Type: Blood**

**Clinical Diagnosis:**

**Sample No.:** **22331542**

**Result:** ***Candida albicans***

Antibiotic MIC

Amphotericin B <0.5 Fluconazole <1.0

Itraconazole <0.125

Voriconazole <0.062

AST Result Interpretation

WT S

S

Breakpoint

2 2-8

0.12-1

Note: S: Sensitive,R: Resistant, I: Intermediat

Identification Method: MOLDI TOF

Specimen submission time: 2022.12.09 07:30 Report Release Time: 2022.12.13 08:37 Laboratory Technician: Report Reviewer: Laboratory test order time: 2022.12.08 16:01 Ordering Physician:xxx

**This report applies solely to the submitted specimen. For inquiries regarding test results, please contact the Laboratory prior to 17:00 on working days.**

**Name:** 　 **Gender:** **f**

**Age:** 34

**Department:** **ICU Patient ID:**

**Inpatient No.:**

**Bed No.:** 　 **Specimen Type: Blood**

**Clinical Diagnosis:**

**Sample No.:** **22331563**

**Result:** ***Candida albicans***

Antibiotic MIC

Amphotericin B <0.5 Fluconazole <1.0

Itraconazole <0.125

Voriconazole <0.062

AST Result Interpretation

WT S

S

Breakpoint

2 2-8

0.12-1

Note: S: Sensitive,R: Resistant, I: Intermediat, nWT: non-Wild Type, WT: Wild Type

Identification Method: MOLDI TOF

Specimen submission time: 2022.12.09 08:10 Report Release Time:2022.12.13 08:33 Laboratory Technician: Report Reviewer: Laboratory test order time: 2022.12.08 17:44 Ordering Physician:xxx

**This report applies solely to the submitted specimen. For inquiries regarding test results, please contact the Laboratory prior to 17:00 on working days.**

**Name:** 　 **Gender: M**

**Age:** 88

**Department: Patient ID:**

**Inpatient No.:**

internal Medicine

**Bed No.:** 　 **Specimen Type: Blood**

**Clinical Diagnosis:**

**Sample No.:** **22327254**

**Result:** ***Candida parapsilosis***

Antibiotic MIC

Amphotericin B <0.5 Fluconazole <1

Itraconazole <0.125

Voriconazole <0.062

AST Result Interpretation

WT S

WT

S

Breakpoint

1 2-8

0.5

0.12-1

Note: S: Sensitive,R: Resistant, I: Intermediat, nWT: non-Wild Type, WT: Wild Type

Identification Method: MOLDI TOF

Specimen submission time:2022.10.19 16:44 Report Release Time: 2022.10.23 11:21 Laboratory Technician: Report Reviewer: Laboratory test order time:2022.10.19 16:10 Ordering Physician:xxx

**This report applies solely to the submitted specimen. For inquiries regarding test results, please contact the Laboratory prior to 17:00 on working days.**

**Name:**

**Gender:** **M**

**Age:** 88

**Department:**

**Patient ID:**

**Inpatient No.:**

surgical department

**Bed No.:**

**Specimen Type: Blood**

**Clinical Diagnosis:**

**Sample No.:** **22331123**

**Result:** ***Candida parapsilosis***

Antibiotic MIC

Amphotericin B <0.5 Fluconazole <1

Itraconazole <0.125

Voriconazole <0.062

AST Result Interpretation

WT S

WT

S

Breakpoint

1 2-8

0.5

0.12-1

Note: S: Sensitive,R: Resistant, I: Intermediat, nWT: non-Wild Type, WT: Wild Type

Identification Method: MOLDI TOF

Specimen submission time: 2022.12.03 16:07 Report Release Time:2022.12.07 08:16 Laboratory Technician: Report Reviewer: Laboratory test order time:2022.12.03 14:36 Ordering Physician:xxx

**This report applies solely to the submitted specimen. For inquiries regarding test results, please contact the Laboratory prior to 17:00 on working days.**

**Name:** 　 **Gender: M**

**Age:** 88

**Department: Patient ID:**

**Inpatient No.:**

internal Medicine

**Bed No.:** 　 **Specimen Type: Blood**

**Clinical Diagnosis:**

**Sample No.:** **22332444**

**Result:** ***Candida parapsilosis***

Antibiotic MIC

Amphotericin B <0.5 Fluconazole <1

Itraconazole <0.125

Voriconazole <0.062

AST Result Interpretation

WT S

WT

S

Breakpoint

1 2-8

0.5

0.12-1

Note: S: Sensitive,R: Resistant, I: Intermediat, nWT: non-Wild Type, WT: Wild Type

Identification Method: MOLDI TOF

Specimen submission time:2022.12.19 10.44 Report Release Time: 2022.12.23 07:47 Laboratory Technician: Report Reviewer: Laboratory test order time: 2022.12.19 09:04 Ordering Physician:xxx

**This report applies solely to the submitted specimen. For inquiries regarding test results, please contact the Laboratory prior to 17:00 on working days.**

**Name:** 　 **Gender: m**

**Age:** 24d

**Department: Patient ID:**

**Inpatient No.:**

pediatrics

**Bed No.:** 　 **Specimen Type: Blood**

**Clinical Diagnosis:**

**Sample No.:** **17337791**

**Result:** ***Candida guilliermondii***

Antibiotic MIC AST Result Interpretation Breakpoint

Amphotericin B <0.5 Fluconazole =2.0

Itraconazole =0.25

Voriconazole <0.062

Note: S: Sensitive,R: Resistant, I: Intermediat

Identification Method: MOLDI TOF

Specimen submission time: 2017.02.22 11:25 Report Release Time: 2017.02.25 09;55 Laboratory Technician: Report Reviewer: Laboratory test order time:2017.02.22 09:59 Ordering Physician:xxx

**This report applies solely to the submitted specimen. For inquiries regarding test results, please contact the Laboratory prior to 17:00 on working days.**

**Name:**

**Gender:** **m**

**Age:** 63

**Department:**

**Patient ID:**

**Inpatient No.:**

**Surgical Department**

**Bed No.:**

**Specimen Type: Blood**

**Clinical Diagnosis:**

**Sample No.:** **22339053**

**Result:** ***Candida parapsilosis***

Antibiotic MIC

Amphotericin B <0.5 Fluconazole <1.0

Itraconazole <0.125

Voriconazole <0.062

AST Result Interpretation

WT S

S

S

Breakpoint

1 2-8

0.5

0.12-1

Note: S: Sensitive,R: Resistant, I: Intermediat

Identification Method: MOLDI TOF

Specimen submission time: 2022.03.08 22:02 Report Release Time:2022.03.12 06:50 Laboratory Technician: Report Reviewer: Laboratory test order time: 2022.03.08 17:11 Ordering Physician:xxx

**This report applies solely to the submitted specimen. For inquiries regarding test results, please contact the Laboratory prior to 17:00 on working days.**

**Name:** 　 **Gender: m**

**Age:** 58

**Department:** **ICU Patient ID:**

**Inpatient No.:**

**Bed No.:** 　 **Specimen Type: Blood**

**Clinical Diagnosis:**

**Sample No.:** **17339523**

**Result:** ***Candida glabrata***

Antibiotic MIC AST Result Interpretation Breakpoint

Amphotericin B <0.5 Fluconazole =1

Itraconazole =0.125

Voriconazole <0.062

Note: S: Sensitive,R: Resistant, I: Intermediat

Identification Method: MOLDI TOF

Specimen submission time: 2017.03.13 15:14 Report Release Time: 2017.03.21 09:51 Laboratory Technician: Report Reviewer: Laboratory test order time:2017.03.13 10:30 Ordering Physician: xxx

**This report applies solely to the submitted specimen. For inquiries regarding test results, please contact the Laboratory prior to 17:00 on working days.**

**Name:**

**Gender:** **m**

**Age:** 81

**Department:**

**Patient ID:**

**Inpatient No.:**

**Surgical Department**

**Bed No.:**

**Specimen Type: Blood**

**Clinical Diagnosis:**

**Sample No.:** **17340484**

**Result:** ***Candida albicans***

Antibiotic MIC AST Result Interpretation Breakpoint

Amphotericin B <0.5 Fluconazole <1.0

Itraconazole <0.125

Voriconazole <0.062

Note: S: Sensitive,R: Resistant, I: Intermediat

Identification Method: MOLDI TOF

Specimen submission time: 2017.03.22 22:31 Report Release Time: 2017.03.30 09:34 Laboratory Technician: Report Reviewer: Laboratory test order time: 2017.03.22 18:36 Ordering Physician:xxx

**This report applies solely to the submitted specimen. For inquiries regarding test results, please contact the Laboratory prior to 17:00 on working days.**

**Name:** 　 **Gender:** **f**

**Age:** 62

**Department:** **ICU Patient ID:**

**Inpatient No.:**

**Bed No.:** 　 **Specimen Type: Blood**

**Clinical Diagnosis:**

**Sample No.:** **17341701**

**Result:** ***Candida albicans***

Antibiotic MIC AST Result Interpretation Breakpoint

Amphotericin B <0.5 Fluconazole <1.0

Itraconazole =0.25

Voriconazole <0.062

Note: S: Sensitive,R: Resistant, I: Intermediat

Identification Method: MOLDI TOF

Specimen submission time: 2017.04.04 17:31 Report Release Time:2017.04.09 09:00 Laboratory Technician: Report Reviewer: Laboratory test order time:2017.04.04 15:53 Ordering Physician: xxx

**This report applies solely to the submitted specimen. For inquiries regarding test results, please contact the Laboratory prior to 17:00 on working days.**

**Name:** 　 **Gender:** **F**

**Age:** 62

**Department:** **ICU Patient ID:**

**Inpatient No.:**

**Bed No.:** 　 **Specimen Type: Blood**

**Clinical Diagnosis:**

**Sample No.:** **17342146**

**Result:** ***Candida albicans***

Antibiotic MIC AST Result Interpretation Breakpoint

Amphotericin B <0.5 Fluconazole =16.0

Itraconazole =0.25

Voriconazole =1.0

Note: S: Sensitive,R: Resistant, I: Intermediat

Identification Method: MOLDI TOF

Specimen submission time:2017.04.09 15:52 Report Release Time:2017.04.14 07:49 Laboratory Technician: Report Reviewer: Laboratory test order time: 2017.04.09 09:45 Ordering Physician:xxx

**This report applies solely to the submitted specimen. For inquiries regarding test results, please contact the Laboratory prior to 17:00 on working days.**

**Name:**

**Gender:** **m**

**Age:** 62

**Department:**

**Patient ID:**

**Inpatient No.:**

surgical department

**Bed No.:**

**Specimen Type: Blood**

**Clinical Diagnosis:**

**Sample No.:** **17349831**

**Result:** ***Candida albicans***

Antibiotic MIC AST Result Interpretation Breakpoint

Amphotericin B <0.5 Fluconazole =1.0

Itraconazole =0.25

Voriconazole <0.062

Note: S: Sensitive,R: Resistant, I: Intermediat

Identification Method: MOLDI TOF

Specimen submission time: 2017.07.07 12:30 Report Release Time: 2017.07.07 10:54 Laboratory Technician: Report Reviewer: Laboratory test order time:2017.07.04 11:15 Ordering Physician:xxx

**This report applies solely to the submitted specimen. For inquiries regarding test results, please contact the Laboratory prior to 17:00 on working days.**

**Name:** 　 **Gender:** **f**

**Age:** 62

**Department: Patient ID:**

**Inpatient No.:**

internal Medicine

**Bed No.:** 　 **Specimen Type: Blood**

**Clinical Diagnosis:**

**Sample No.:** **17351236**

**Result:** ***Candida tropicalis***

Antibiotic MIC AST Result Interpretation Breakpoint

Amphotericin B <0.5 Fluconazole <1.0

Itraconazole <0.125

Voriconazole <0.062

Note: S: Sensitive,R: Resistant, I: Intermediat

Identification Method: MOLDI TOF

Specimen submission time: 2017.07.19 12;22 Report Release Time: 2017.07.25 08:39 Laboratory Technician: Report Reviewer: Laboratory test order time: 2017.07.19 11:20 Ordering Physician:xxx

**This report applies solely to the submitted specimen. For inquiries regarding test results, please contact the Laboratory prior to 17:00 on working days.**

**Name:** 　 **Gender: m**

**Age:** 62

**Department:** **ICU Patient ID:**

**Inpatient No.:**

**Bed No.:** 　 **Specimen Type: Blood**

**Clinical Diagnosis:**

**Sample No.:** **17362414**

**Result:** ***Candida tropicalis***

Antibiotic MIC AST Result Interpretation Breakpoint

Amphotericin B <0.5 Fluconazole <1.0

Itraconazole <0.125

Voriconazole =0.125

Note: S: Sensitive,R: Resistant, I: Intermediat

Identification Method: MOLDI TOF

Specimen submission time: 2017.08.04 09:14 Report Release Time: 2017.08.07 10:56 Laboratory Technician: Report Reviewer: Laboratory test order time: 2017.08.04 07:07 Ordering Physician:xxx

**This report applies solely to the submitted specimen. For inquiries regarding test results, please contact the Laboratory prior to 17:00 on working days.**

**Name:**

**Gender:** **m**

**Age:** 70

**Department:**

**Patient ID:**

**Inpatient No.:**

**Surgical Department**

**Bed No.:**

**Specimen Type: Blood**

**Clinical Diagnosis:**

**Sample No.:** **17362614**

**Result:** ***Candida parapsilosis***

Antibiotic MIC AST Result Interpretation Breakpoint

Amphotericin B <0.5 Fluconazole <1.0

Itraconazole <0.125

Voriconazole <0.062

Note: S: Sensitive,R: Resistant, I: Intermediat

Identification Method: MOLDI TOF

Specimen submission time: 2017.08.07 00:10 Report Release Time: 2017.08.10 09:33 Laboratory Technician: Report Reviewer: Laboratory test order time: 2017.08.06 14:48 Ordering Physician:xxx

**This report applies solely to the submitted specimen. For inquiries regarding test results, please contact the Laboratory prior to 17:00 on working days.**

**Name:** 　 **Gender:** **f**

**Age:** 59

**Department:** N **ICU Patient ID:**

**Inpatient No.:**

**Bed No.:** 　 **Specimen Type: Blood**

**Clinical Diagnosis:**

**Sample No.:** **17362719**

**Result:** ***Candida parapsilosis***

Antibiotic MIC AST Result Interpretation Breakpoint

Amphotericin B =1.0 Fluconazole <1.0

Itraconazole <0.125

Voriconazole <0.062

Note: S: Sensitive,R: Resistant, I: Intermediat

Identification Method: MOLDI TOF

Specimen submission time: 2017.08.07 19:58 Report Release Time:2017.08.10 09:28 Laboratory Technician: Report Reviewer: Laboratory test order time: 2017.08.07 14:35 Ordering Physician:xxx

**This report applies solely to the submitted specimen. For inquiries regarding test results, please contact the Laboratory prior to 17:00 on working days.**

**Name:** 　 **Gender:** **F**

**Age:** 　59

**Department:** N**ICU Patient ID:**

**Inpatient No.:**

**Bed No.:** 　 **Specimen Type: Blood**

**Clinical Diagnosis:**

**Sample No.:** **17362716**

**Result:** ***Candida parapsilosis***

Antibiotic MIC AST Result Interpretation Breakpoint

Amphotericin B =1.0 Fluconazole <1.0

Itraconazole <0.125

Voriconazole <0.062

Note: S: Sensitive,R: Resistant, I: Intermediat

Identification Method: MOLDI TOF

Specimen submission time:2017.08.07 19:45 Report Release Time: 2017.08.11 09;12 Laboratory Technician: Report Reviewer: Laboratory test order time: 2017.08.07 14:36 Ordering Physician:xxx

**This report applies solely to the submitted specimen. For inquiries regarding test results, please contact the Laboratory prior to 17:00 on working days.**

**Name:** 　 **Gender:** **f**

**Age:** 89

**Department: Patient ID:**

**Inpatient No.:**

internal Medicine

**Bed No.:** 　 **Specimen Type: Blood**

**Clinical Diagnosis:**

**Sample No.:** **17363837**

**Result:** ***Candida tropicalis***

Antibiotic MIC

Amphotericin B <0.5 Fluconazole <1.0

Itraconazole <0.125

Voriconazole <0.062

AST Result Interpretation

Breakpoint

Note: S: Sensitive,R: Resistant, I: Intermediat

Identification Method: MOLDI TOF

Specimen submission time: 2017.08.18 16;06 Report Release Time: 2017.08.21 09:17 Laboratory Technician: Report Reviewer: Laboratory test order time: 2017.08.18 14:48 Ordering Physician:xxx

**This report applies solely to the submitted specimen. For inquiries regarding test results, please contact the Laboratory prior to 17:00 on working days.**

**Name:** 　 **Gender:** **f**

**Age:** 89

**Department: Patient ID:**

**Inpatient No.:**

internal Medicine

**Bed No.:** 　 **Specimen Type: Blood**

**Clinical Diagnosis:**

**Sample No.:** **17363913**

**Result:** ***Candida tropicalis***

Antibiotic MIC

Amphotericin B <0.5 Fluconazole <1.0

Itraconazole <0.125

Voriconazole <0.062

AST Result Interpretation

Breakpoint

Note: S: Sensitive,R: Resistant, I: Intermediat

Identification Method: MOLDI TOF

Specimen submission time: 2017.08.19 22:34 Report Release Time:2017.08.23 10: 44 Laboratory Technician: Report Reviewer: Laboratory test order time: 2017.08.19 18:13 Ordering Physician:xxx

**This report applies solely to the submitted specimen. For inquiries regarding test results, please contact the Laboratory prior to 17:00 on working days.**

**Name:**

**Gender:** **m**

**Age:** 64

**Department:**

**Patient ID:**

**Inpatient No.:**

**Surgical Department**

**Bed No.:**

**Specimen Type: Blood**

**Clinical Diagnosis:**

**Sample No.:** **17364153**

**Result:** ***Candida parapsilosis***

Antibiotic MIC AST Result Interpretation Breakpoint

Amphotericin B <0.5 Fluconazole <1.0

Itraconazole <0.125

Voriconazole <0.062

Note: S: Sensitive,R: Resistant, I: Intermediat

Identification Method: MOLDI TOF

Specimen submission time: 2017.08.22 23:28 Report Release Time:2017.08.27 09:46 Laboratory Technician: Report Reviewer: Laboratory test order time: 2017.08.22 19:21 Ordering Physician:xxx

**This report applies solely to the submitted specimen. For inquiries regarding test results, please contact the Laboratory prior to 17:00 on working days.**

**Name:**

**Gender:** **m**

**Age:** 61

**Department:**

**Patient ID:**

**Inpatient No.:**

**Surgical Department**

**Bed No.:**

**Specimen Type: Blood**

**Clinical Diagnosis:**

**Sample No.:** **17365704**

**Result:** ***Candida parapsilosis***

Antibiotic MIC AST Result Interpretation Breakpoint

Amphotericin B <0.5 Fluconazole <1.0

Itraconazole <0.125

Voriconazole <0.062

Note: S: Sensitive,R: Resistant, I: Intermediat

Identification Method: MOLDI TOF

Specimen submission time: 2017.09.07 21:47 Report Release Time: 2017.09.12 08;51 Laboratory Technician: Report Reviewer: Laboratory test order time:2017.09.07 18:23 Ordering Physician:xxx

**This report applies solely to the submitted specimen. For inquiries regarding test results, please contact the Laboratory prior to 17:00 on working days.**

**Name:**

**Gender:** **m**

**Age:** 64

**Department:**

**Patient ID:**

**Inpatient No.:**

**Surgical Department**

**Bed No.:**

**Specimen Type: Blood**

**Clinical Diagnosis:**

**Sample No.:** **17366080**

**Result:** ***Candida tropicalis***

Antibiotic MIC AST Result Interpretation Breakpoint

Amphotericin B <0.5 Fluconazole <1

Itraconazole <0.125

Voriconazole <0.062

Note: S: Sensitive,R: Resistant, I: Intermediat

Identification Method: MOLDI TOF

Specimen submission time: 2017.09.13 01:30 Report Release Time: 2017.09.16 11:08 Laboratory Technician: Report Reviewer: Laboratory test order time: 2017.09.12 23:58 Ordering Physician:xxx

**This report applies solely to the submitted specimen. For inquiries regarding test results, please contact the Laboratory prior to 17:00 on working days.**

**Name:**

**Gender:** **m**

**Age:** 91

**Department:**

**Patient ID:**

**Inpatient No.:**

internal Medicine

**Bed No.:**

**Specimen Type: Blood**

**Clinical Diagnosis:**

**Sample No.:**

**17368662**

**Result:** ***Candida parapsilosis***

Antibiotic MIC AST Result Interpretation Breakpoint

Amphotericin B <0.5 Fluconazole <1

Itraconazole <0.125

Voriconazole <0.062

Note: S: Sensitive,R: Resistant, I: Intermediat

Identification Method: MOLDI TOF

Specimen submission time: 2017.10.14 10:23 Report Release Time:2017.10.18 07:41 Laboratory Technician: Report Reviewer: Laboratory test order time: 2017.10.14 09:10 Ordering Physician:xxx

**This report applies solely to the submitted specimen. For inquiries regarding test results, please contact the Laboratory prior to 17:00 on working days.**

**Name:** 　 **Gender: m**

**Age:** 41

**Department:** ICU **Patient ID:**

**Inpatient No.:**

**Bed No.:** 　 **Specimen Type: Blood**

**Clinical Diagnosis:**

**Sample No.:** **17375167**

**Result:** ***Candida tropicalis***

Antibiotic MIC AST Result Interpretation Breakpoint

Amphotericin B <0.5 Fluconazole <1.0

Itraconazole <0.125

Voriconazole =0.062

Note: S: Sensitive,R: Resistant, I: Intermediat

Identification Method: MOLDI TOF

Specimen submission time:2017.12.24 16:32 Report Release Time:2017.12.27 11:56 Laboratory Technician: Report Reviewer: Laboratory test order time:2017.12.24 14:34 Ordering Physician:xxx

**This report applies solely to the submitted specimen. For inquiries regarding test results, please contact the Laboratory prior to 17:00 on working days.**

**Name:**

**Gender:** **F**

**Age:** 45

**Department:**

**Patient ID:**

**Inpatient No.:**

emergency department

**Bed No.:**

**Specimen Type: Blood**

**Clinical Diagnosis:**

**Sample No.:** **17771463**

**Result:** ***Candida famata***

Antibiotic MIC AST Result Interpretation Breakpoint

Fluconazole 8 Itraconazole ≥4

Voriconazole 0.5

Note: S: Sensitive,R: Resistant, I: Intermediat

Identification Method: MOLDI TOF

Specimen submission time: 2017.03.01 08:37 Report Release Time: 2017.03.04 13:24 Laboratory Technician: Report Reviewer: Laboratory test order time: 2017.02.28 21:52 Ordering Physician:xxx

**This report applies solely to the submitted specimen. For inquiries regarding test results, please contact the Laboratory prior to 17:00 on working days.**

**Name:** 　 **Gender: M**

**Age:** 16

**Department: Patient ID:**

**Inpatient No.:**

internal Medicine

**Bed No.:** 　 **Specimen Type: Blood**

**Clinical Diagnosis:**

**Sample No.:** **190723202**

**Result:** ***Candida parapsilosis***

Antibiotic MIC AST Result Interpretation Breakpoint

Amphotericin B <0.5 Fluconazole <0.5

Itraconazole =0.5

Voriconazole <0.012

Note: S: Sensitive,R: Resistant, I: Intermediat

Identification Method: MOLDI TOF

Specimen submission time: 2019.07.23 09:51 Report Release Time:2019.07.26 10:21 Laboratory Technician: Report Reviewer: Laboratory test order time:2019.07.20 09:37 Ordering Physician:xxx

**This report applies solely to the submitted specimen. For inquiries regarding test results, please contact the Laboratory prior to 17:00 on working days.**

**Name:** 　 **Gender:** **F**

**Age:** 　2

**Department: Patient ID:**

**Inpatient No.:**

pediatrics

**Bed No.:** 　 **Specimen Type: Blood**

**Clinical Diagnosis:**

**Sample No.:** **190723506**

**Result:** ***Candida parapsilosis***

Antibiotic MIC AST Result Interpretation Breakpoint

Amphotericin B <0.5 Fluconazole <1.0

Itraconazole <0.125

Voriconazole <0.062

Note: S: Sensitive,R: Resistant, I: Intermediat

Identification Method: MOLDI TOF

Specimen submission time: 2019.07.23 09:51 Report Release Time: 2019.07.26 10:21 Laboratory Technician: Report Reviewer: Laboratory test order time: 2019.07.20 09:37 Ordering Physician:xxx

**This report applies solely to the submitted specimen. For inquiries regarding test results, please contact the Laboratory prior to 17:00 on working days.**

**Name:** 　 **Gender:** **F**

**Age:** 2

**Department: Patient ID:**

**Inpatient No.:**

pediatrics

**Bed No.:** 　 **Specimen Type: Blood**

**Clinical Diagnosis:**

**Sample No.:** **190729508**

**Result:** ***Candida parapsilosis***

Antibiotic MIC AST Result Interpretation Breakpoint

Amphotericin B <0.5 Fluconazole <1.0

Itraconazole <0.125

Voriconazole <0.062

Note: S: Sensitive,R: Resistant, I: Intermediat

Identification Method: MOLDI TOF

Specimen submission time: 2019.07.23 08:00 Report Release Time:2019.07.29 09:42 Laboratory Technician: Report Reviewer: Laboratory test order time: 2019.07.22 15:54 Ordering Physician:xxx

**This report applies solely to the submitted specimen. For inquiries regarding test results, please contact the Laboratory prior to 17:00 on working days.**

**Name:**

**Gender:** **M**

**Age:** 44

**Department:**

**Patient ID:**

**Inpatient No.:**

emergency department

**Bed No.:**

**Specimen Type: Blood**

**Clinical Diagnosis:**

**Sample No.:** **190730211**

**Result:** ***Candida tropicalis***

Antibiotic MIC AST Result Interpretation Breakpoint

Amphotericin B <0.5 Fluconazole =16.0

Itraconazole <0.125

Voriconazole =0.5

Note: S: Sensitive,R: Resistant, I: Intermediat

Identification Method: MOLDI TOF

Specimen submission time: 2019.7.30 16;28 Report Release Time:2019.08.03 09:13 Laboratory Technician: Report Reviewer: Laboratory test order time: 2019.07.30 11:01 Ordering Physician:xxx

**This report applies solely to the submitted specimen. For inquiries regarding test results, please contact the Laboratory prior to 17:00 on working days.**

**Name:** 　 **Gender: M**

**Age:** 79

**Department:** **ICU Patient ID:**

**Inpatient No.:**

**Bed No.:** 　 **Specimen Type: Blood**

**Clinical Diagnosis:**

**Sample No.:** **190807212**

**Result:** ***Candida albicans***

Antibiotic MIC AST Result Interpretation Breakpoint

Amphotericin B <0.5 Fluconazole =2.0

Itraconazole <0.06

Voriconazole <0.062

Note: S: Sensitive,R: Resistant, I: Intermediat

Identification Method: MOLDI TOF

Specimen submission time: 2019.08.07 20:25 Report Release Time:2019.08.12 09:57 Laboratory Technician: Report Reviewer: Laboratory test order time: 2019.08.07 17:46 Ordering Physician: xxx

**This report applies solely to the submitted specimen. For inquiries regarding test results, please contact the Laboratory prior to 17:00 on working days.**

**Name:** 　 **Gender:** **F**

**Age:** 2

**Department: Patient ID:**

**Inpatient No.:**

pediatrics

**Bed No.:** 　 **Specimen Type: Blood**

**Clinical Diagnosis:**

**Sample No.:** **190813521**

**Result:** ***Candida albicans***

Antibiotic MIC AST Result Interpretation Breakpoint

Amphotericin B <0.5 Fluconazole <1.0

Itraconazole <0.125

Voriconazole <0.062

Note: S: Sensitive,R: Resistant, I: Intermediat

Identification Method: MOLDI TOF

Specimen submission time: 2019.08.13 19:15 Report Release Time:2019.08.17 08:38 Laboratory Technician: Report Reviewer: Laboratory test order time: 2019.08.13 11:26 Ordering Physician: xxx

**This report applies solely to the submitted specimen. For inquiries regarding test results, please contact the Laboratory prior to 17:00 on working days.**

**Name:** 　 **Gender: M**

**Age:** 65

**Department: Patient ID:**

**Inpatient No.:**

internal Medicine

**Bed No.:** 　 **Specimen Type: Blood**

**Clinical Diagnosis:**

**Sample No.:** **190829203**

**Result:** ***Candida tropicalis***

Antibiotic MIC AST Result Interpretation Breakpoint

Amphotericin B <0.5 Fluconazole =16.0

Itraconazole <0.125

Voriconazole =0.5

Note: S: Sensitive,R: Resistant, I: Intermediat

Identification Method: MOLDI TOF

Specimen submission time: 2019.08.29 08:22 Report Release Time: 2019.09.01 10:54 Laboratory Technician: Report Reviewer: Laboratory test order time: 2019.08.29 05:53 Ordering Physician:xxx

**This report applies solely to the submitted specimen. For inquiries regarding test results, please contact the Laboratory prior to 17:00 on working days.**

**Name:**

**Gender:** **M**

**Age:** 80

**Department:**

**Patient ID:**

**Inpatient No.:**

surgical department

**Bed No.:**

**Specimen Type: Blood**

**Clinical Diagnosis:**

**Sample No.:** **191015206**

**Result:** ***Candida albicans***

Antibiotic KB

Amphotericin B 0.5 Fluconazole 1.0

Itraconazole 0.125

Voriconazole 0.5

AST Result Interpretation

Breakpoint

Note: S: Sensitive,R: Resistant, I: Intermediat

Identification Method: MOLDI TOF

Specimen submission time: 2019.10.15 16:30 Report Release Time: 2019.10.19 09:44 Laboratory Technician: Report Reviewer: Laboratory test order time: 2019.10.15 11:14 Ordering Physician: xxx

**This report applies solely to the submitted specimen. For inquiries regarding test results, please contact the Laboratory prior to 17:00 on working days.**

**Name:**

**Gender:** **F**

**Age:** 48

**Department:**

**Patient ID:**

**Inpatient No.:**

**Surgical Department**

**Bed No.:**

**Specimen Type: Blood**

**Clinical Diagnosis:**

**Sample No.:** **191027202**

**Result:** ***Candida parapsilosis***

Antibiotic MIC AST Result Interpretation Breakpoint

Amphotericin B <0.5 Fluconazole <1

Itraconazole <0.12

Voriconazole <0.06

Note: S: Sensitive,R: Resistant, I: Intermediat

Identification Method: MOLDI TOF

Specimen submission time: 2019.10.27 08:06 Report Release Time:2019.10.30 10:47 Laboratory Technician: Report Reviewer: Laboratory test order time: 2019.10.26 20:00 Ordering Physician: xxx

**This report applies solely to the submitted specimen. For inquiries regarding test results, please contact the Laboratory prior to 17:00 on working days.**

**Name:** 　 **Gender: M**

**Age:** 54

**Department:** **ICU Patient ID:**

**Inpatient No.:**

**Bed No.:** 　 **Specimen Type: Blood**

**Clinical Diagnosis:**

**Sample No.:** **191031202**

**Result:** ***Candida glabrata***

Antibiotic MIC AST Result Interpretation Breakpoint

Amphotericin B <0.5 Fluconazole =1

Itraconazole =0.5

Voriconazole <0.06

Note: S: Sensitive,R: Resistant, I: Intermediat

Identification Method: MOLDI TOF

Specimen submission time: 2019.10.31 01;59 Report Release Time: 2019.11.05 10:03 Laboratory Technician: Report Reviewer: Laboratory test order time: 2019.10.31 00:48 Ordering Physician: xxx

**This report applies solely to the submitted specimen. For inquiries regarding test results, please contact the Laboratory prior to 17:00 on working days.**

**Name:**

**Gender:** **M**

**Age:** 60

**Department:**

**Patient ID:**

**Inpatient No.:**

**Surgical Department**

**Bed No.:**

**Specimen Type: Blood**

**Clinical Diagnosis:**

**Sample No.:** **191107207**

**Result:** ***Candida parapsilosis***

Antibiotic MIC AST Result Interpretation Breakpoint

Amphotericin B <0.5 Fluconazole <1

Itraconazole <0.12

Voriconazole <0.06

Note: S: Sensitive,R: Resistant, I: Intermediat

Identification Method: MOLDI TOF

Specimen submission time: 2019.11.7.20.26 Report Release Time: 2019.11.11.9.46 Laboratory Technician: Report Reviewer: Laboratory test order time:2019.11.7. 17.21 Ordering Physician: xxx

**This report applies solely to the submitted specimen. For inquiries regarding test results, please contact the Laboratory prior to 17:00 on working days.**

**Name:**

**Gender:** **M**

**Age:** 60

**Department:**

**Patient ID:**

**Inpatient No.:**

**Surgical Department**

**Bed No.:**

**Specimen Type: Blood**

**Clinical Diagnosis:**

**Sample No.:** **191109202**

**Result:** ***Candida parapsilosis***

Antibiotic MIC AST Result Interpretation Breakpoint

Amphotericin B <0.5 Fluconazole <1

Itraconazole <0.12

Voriconazole <0.06

Note: S: Sensitive,R: Resistant, I: Intermediat

Identification Method: MOLDI TOF

Specimen submission time:2019.11.09 2:08 Report Release Time:2019.11.12 10:32 Laboratory Technician: Report Reviewer: Laboratory test order time:2019.11.8 19:26 Ordering Physician: xxx

**This report applies solely to the submitted specimen. For inquiries regarding test results, please contact the Laboratory prior to 17:00 on working days.**

**Name:**

**Gender:** **M**

**Age:** 60

**Department:**

**Patient ID:**

**Inpatient No.:**

emergency department

**Bed No.:**

**Specimen Type: Blood**

**Clinical Diagnosis:**

**Sample No.:** **191115208**

**Result:** ***Candida albicans***

Antibiotic MIC AST Result Interpretation Breakpoint

Amphotericin B <0.5 Fluconazole <1

Itraconazole <0.12

Voriconazole <0.06

Note: S: Sensitive,R: Resistant, I: Intermediat

Identification Method: MOLDI TOF

Specimen submission time: 2019.11.15 20:14 Report Release Time: 2019.11.20 9:37Laboratory Technician: Report Reviewer: Laboratory test order time: 2019.11.15 17:55 Ordering Physician: xxx

**This report applies solely to the submitted specimen. For inquiries regarding test results, please contact the Laboratory prior to 17:00 on working days.**

**Name:**

**Gender:** **M**

**Age:** 60

**Department:**

**Patient ID:**

**Inpatient No.:**

emergency department

**Bed No.:**

**Specimen Type: Blood**

**Clinical Diagnosis:**

**Sample No.:** **191117208**

**Result:** ***Candida albicans***

Antibiotic MIC AST Result Interpretation Breakpoint

Amphotericin B <0.5 Fluconazole <1

Itraconazole <0.12

Voriconazole <0.06

Note: S: Sensitive,R: Resistant, I: Intermediat

Identification Method: MOLDI TOF

Specimen submission time:2019.11.17 20:18 Report Release Time:2019.11.2 18:48 Laboratory Technician: Report Reviewer: Laboratory test order time:2019.11.17 18:09 Ordering Physician: xxx

**This report applies solely to the submitted specimen. For inquiries regarding test results, please contact the Laboratory prior to 17:00 on working days.**

**Name:**

**Gender:** **M**

**Age:** 74

**Department:**

**Patient ID:**

**Inpatient No.:**

emergency department

**Bed No.:**

**Specimen Type: Blood**

**Clinical Diagnosis:**

**Sample No.:** **191202208**

**Result:** ***Candida parapsilosis***

Antibiotic MIC AST Result Interpretation Breakpoint

Amphotericin B <0.5 Fluconazole <1

Itraconazole <0.12

Voriconazole <0.06

Note: S: Sensitive,R: Resistant, I: Intermediat

Identification Method: MOLDI TOF

Specimen submission time: 2019.12.2 13:15 Report Release Time: 2019.12.6 09:31 Laboratory Technician: Report Reviewer: Laboratory test order time:2019.12.2 07:24 Ordering Physician: xxx

**This report applies solely to the submitted specimen. For inquiries regarding test results, please contact the Laboratory prior to 17:00 on working days.**

**Name:** 　 **Gender: M**

**Age:** 19

**Department:** **NICU Patient ID:**

**Inpatient No.:**

**Bed No.:** 　 **Specimen Type: Blood**

**Clinical Diagnosis:**

**Sample No.:** **200406204**

**Result:** ***Candida parapsilosis***

Antibiotic

　Amphotericin B Fluconazole Itraconazole

Voriconazole

MIC AST Result Interpretation

<0.5 WT

<1 S <0.12 WT

<0.06 S

Breakpoint

1

2-8 0.5

0.125-1

Note: S: Sensitive,R: Resistant, I: Intermediat, nWT: non-Wild Type, WT: Wild Type

Identification Method: MOLDI TOF

Specimen submission time:2020.4.6 11:07 Report Release Time:2020.4.11 11:03 Laboratory Technician: Report Reviewer: Laboratory test order time:2020.4.6 8:30 Ordering Physician: xxx

**This report applies solely to the submitted specimen. For inquiries regarding test results, please contact the Laboratory prior to 17:00 on working days.**

**Name:** 　 **Gender: M**

**Age:** 64

**Department:** **ICU Patient ID:**

**Inpatient No.:**

**Bed No.:** 　 **Specimen Type: Blood**

**Clinical Diagnosis:**

**Sample No.:** **200520203**

**Result:** ***Candida tropicalis***

Antibiotic

　Amphotericin B

Fluconazole

Itraconazole

Voriconazole

MIC AST Result Interpretation

<0.5 WT

<1 S

=0.25 I

=0.25 I

Breakpoint

　2

2-8

0.125-1

0.125-1

Note: S: Sensitive,R: Resistant, I: Intermediat, nWT: non-Wild Type, WT: Wild Type

Identification Method: MOLDI TOF

Specimen submission time:2020.5.20 15:06 Report Release Time:2020.5.24 11:19 Laboratory Technician: Report Reviewer: Laboratory test order time:2020.5.20 13:39 Ordering Physician: xxx

**This report applies solely to the submitted specimen. For inquiries regarding test results, please contact the Laboratory prior to 17:00 on working days.**

**Name:** 　 **Gender: M**

**Age:** 64

**Department:** **ICU Patient ID:**

**Inpatient No.:**

**Bed No.:** 　 **Specimen Type: Blood**

**Clinical Diagnosis:**

**Sample No.:** **200520203**

**Result:** ***Candida tropicalis***

Antibiotic

　Amphotericin B

Fluconazole

Itraconazole

Voriconazole

MIC AST Result Interpretation

<0.5 WT

<1 S

=0.25 I

=0.25 I

Breakpoint

　2

2-8

0.125-1

0.125-1

Note: S: Sensitive,R: Resistant, I: Intermediat, nWT: non-Wild Type, WT: Wild Type

Identification Method: MOLDI TOF

Specimen submission time:2020.5.20 15:08 Report Release Time:2020.5.24 11:19 Laboratory Technician: Report Reviewer: Laboratory test order time:2020.5.20 13:39 Ordering Physician: xxx

**This report applies solely to the submitted specimen. For inquiries regarding test results, please contact the Laboratory prior to 17:00 on working days.**

**Name:** 　 **Gender: m**

**Age:** 64

**Department:** **ICU Patient ID:**

**Inpatient No.:**

**Bed No.:** 　 **Specimen Type: Blood**

**Clinical Diagnosis:**

**Sample No.:** **200522203**

**Result:** ***Candida tropicalis***

Antibiotic

　Amphotericin B

Fluconazole

Itraconazole

Voriconazole

MIC AST Result Interpretation

<0.5 WT

<1 S

=0.25 I

=0.25 I

Breakpoint

2

2-8

0.125-1

0.125-1

Note: S: Sensitive,R: Resistant, I: Intermediat, nWT: non-Wild Type, WT: Wild Type

Identification Method: MOLDI TOF

Specimen submission time: 2020.5.22 8:28 Report Release Time:2020.5.25 10:13 Laboratory Technician: Report Reviewer: Laboratory test order time:2020.5.21 19:48 Ordering Physician:xxx

**This report applies solely to the submitted specimen. For inquiries regarding test results, please contact the Laboratory prior to 17:00 on working days.**

**Name:**

**Gender:** **F**

**Age:** 75

**Department:**

**Patient ID:**

**Inpatient No.:**

**Surgical Department**

**Bed No.:**

**Specimen Type: Blood**

**Clinical Diagnosis:**

**Sample No.:** **200605207**

**Result:** ***Candida parapsilosis***

Antibiotic

　Amphotericin B Fluconazole Itraconazole

Voriconazole

MIC AST Result Interpretation

<0.5 WT

<1 S <0.12 WT

<0.06 S

Breakpoint

1

2-8 0.5

0.125-1

Note: S: Sensitive,R: Resistant, I: Intermediat, nWT: non-Wild Type, WT: Wild Type

Identification Method: MOLDI TOF

Specimen submission time:2020.6.5 23:49 Report Release Time:2020.6.9 3:50 Laboratory Technician: Report Reviewer: Laboratory test order time:2020.6.5 17:41 Ordering Physician:xxx

**This report applies solely to the submitted specimen. For inquiries regarding test results, please contact the Laboratory prior to 17:00 on working days.**

**Name:**

**Gender:** **f**

**Age:** 75

**Department:**

**Patient ID:**

**Inpatient No.:**

**Surgical Department**

**Bed No.:**

**Specimen Type: Blood**

**Clinical Diagnosis:**

**Sample No.:** **200731206**

**Result:** ***Candida albicans***

Antibiotic

　Amphotericin B

Fluconazole

MIC AST Result Interpretation

<0.5 WT

<1 S

Breakpoint

2

8-64

Itraconazole <0.12

Voriconazole <0.06 S 1-4

Note: S: Sensitive,R: Resistant, I: Intermediat, nWT: non-Wild Type, WT: Wild Type

Identification Method: MOLDI TOF

Specimen submission time: 2020.7.31 14:54 Report Release Time:2020.8.5 10:00 Laboratory Technician: Report Reviewer: Laboratory test order time:2020.7.31 8;59 Ordering Physician: xxx

**This report applies solely to the submitted specimen. For inquiries regarding test results, please contact the Laboratory prior to 17:00 on working days.**

**Name:** 　 **Gender: m**

**Age:** 21

**Department:** EICU **Patient ID:**

**Inpatient No.:**

**Bed No.:** 　 **Specimen Type: Blood**

**Clinical Diagnosis:**

**Sample No.:** **240614019**

**Result:** ***Candida rugosa***

Antibiotic

　Amphotericin B

5-Fluorocytosine

Fluconazole

Itraconazole

MIC AST Result Interpretation

<0.5

≤4 <1

≤0.12

Breakpoint

Voriconazole ≤0.06

Note: S: Sensitive,R: Resistant, I: Intermediat

Identification Method: MOLDI TOF

Specimen submission time:2024.6.14 19:42 Report Release Time:2024.6.17 8:27 Laboratory Technician: Report Reviewer: Laboratory test order time:2024.6.14 17:39 Ordering Physician:xxx

**This report applies solely to the submitted specimen. For inquiries regarding test results, please contact the Laboratory prior to 17:00 on working days.**

**Name:** 　 **Gender: m**

**Age:** 21

**Department: Patient ID:**

**Inpatient No.:**

internal Medicine

**Bed No.:** 　 **Specimen Type: Blood**

**Clinical Diagnosis:**

**Sample No.:** **240930548**

**Result:** ***Candida rugosa***

Antibiotic MIC

Caspofungin 4 Micafungin　 1

Voriconazole ≤0.12

Fluconazole 2

Amphotericin B 1

AST Result Interpretation

Breakpoint

Note: S: Sensitive,R: Resistant, I: Intermediat

Identification Method: MOLDI TOF

Specimen submission time:2024.9.30 16:13 Report Release Time:2024.10.2 9:23 Laboratory Technician: Report Reviewer: Laboratory test order time:2024.9.30 11:49 Ordering Physician: xxx

**This report applies solely to the submitted specimen. For inquiries regarding test results, please contact the Laboratory prior to 17:00 on working days.**

**Name:** 　 **Gender: m**

**Age:** 21

**Department: Patient ID:**

**Inpatient No.:**

internal Medicine

**Bed No.:** 　 **Specimen Type: Blood**

**Clinical Diagnosis:**

**Sample No.:** **210401001**

**Result:** ***Candida famata***

Antibiotic

　Amphotericin B

5-Fluorocytosine

Fluconazole

Itraconazole

MIC AST Result Interpretation

<0.5

≤4 <1

≥8

Breakpoint

Voriconazole <0.06

Note: S: Sensitive, R: Resistant, I: Intermediat

Identification Method: MOLDI TOF

Specimen submission time:2021.4.1 2:22 Report Release Time:2021.4.6 8:19 Laboratory Technician: Report Reviewer: Laboratory test order time:2021.3.31 22:26 Ordering Physician: xxx

**This report applies solely to the submitted specimen. For inquiries regarding test results, please contact the Laboratory prior to 17:00 on working days.**

**Name:** 　 **Gender: m**

**Age:** 23

**Department:** **EICU Patient ID:**

**Inpatient No.:**

**Bed No.:** 　 **Specimen Type: Blood**

**Clinical Diagnosis:**

**Sample No.:** **200816211**

**Result:** ***Candida parapsilosis***

Antibiotic

　Amphotericin B

5-Fluorocytosine

Fluconazole

Itraconazole

MIC AST Result Interpretation

<0.5

≤4 <1

<0.12

Breakpoint

Voriconazole <0.06

Note: S: Sensitive,R: Resistant, I: Intermediat

Identification Method: MOLDI TOF

Specimen submission time: 2020.8.16 19:46 Report Release Time:2020.8.21 10:16 Laboratory Technician: Report Reviewer: Laboratory test order time:2020.8.16 17:15 Ordering Physician: xxx

**This report applies solely to the submitted specimen. For inquiries regarding test results, please contact the Laboratory prior to 17:00 on working days.**

**Name:** 　 **Gender: M**

**Age:** 56

**Department: Patient ID:**

**Inpatient No.:**

internal Medicine

**Bed No.:** 　 **Specimen Type: Blood**

**Clinical Diagnosis:**

**Sample No.:** **201007202**

**Result:** ***Candida tropicalis***

Antibiotic

　Amphotericin B

Fluconazole

Itraconazole

Voriconazole

MIC AST Result Interpretation

<0.5 WT

≥256 R

≥8 nWT

≥16 R

Breakpoint

　2

2-8

0.5

0.125-1

Note: S: Sensitive, R: Resistant, I: Intermediat, nWT: non-Wild Type, WT: Wild Type

Identification Method: MOLDI TOF

Specimen submission time:2020.10.7 17:47 Report Release Time:2020.10.10 3:33 Laboratory Technician: Report Reviewer: Laboratory test order time:2020.10.7 10:54 Ordering Physician:xxx

**This report applies solely to the submitted specimen. For inquiries regarding test results, please contact the Laboratory prior to 17:00 on working days.**

**Name:** 　 **Gender: m**

**Age:** 56

**Department: Patient ID:**

**Inpatient No.:**

internal Medicine

**Bed No.:** 　 **Specimen Type: Blood**

**Clinical Diagnosis:**

**Sample No.:** **201009204**

**Result:** ***Candida tropicalis***

Antibiotic

　Amphotericin B

Fluconazole

Itraconazole

Voriconazole

MIC AST Result Interpretation

<0.5 WT

≥256 R

≥8 nWT

≥16 R

Breakpoint

　2

2-8

0.5

0.125-1

Note: S: Sensitive, R: Resistant, I: Intermediat, nWT: non-Wild Type, WT: Wild Type

Identification Method: MOLDI TOF

Specimen submission time:2020.10.9 11:21 Report Release Time:2020.10.12 8:46 Laboratory Technician: Report Reviewer: Laboratory test order time:2020.10.9 10:33 Ordering Physician: xxx

**This report applies solely to the submitted specimen. For inquiries regarding test results, please contact the Laboratory prior to 17:00 on working days.**

**Name:**

**Gender:** **F**

**Age:** 76

**Department:**

**Patient ID:**

**Inpatient No.:**

**Surgical Department**

**Bed No.:**

**Specimen Type: Blood**

**Clinical Diagnosis:**

**Sample No.:** **201013201**

**Result:** ***Candida glabrata***

Antibiotic

　Amphotericin B

Fluconazole

Itraconazole

MIC AST Result Interpretation

<0.5

=16 I

=0.5

Breakpoint

8-64

Voriconazole <0.06

Note: S: Sensitive,R: Resistant, I: Intermediat

Identification Method: MOLDI TOF

Specimen submission time:2020.10.13 00:32 Report Release Time:2020.10.13 9:58 Laboratory Technician: Report Reviewer: Laboratory test order time:2020.10.12 9:40 Ordering Physician:xxx

**This report applies solely to the submitted specimen. For inquiries regarding test results, please contact the Laboratory prior to 17:00 on working days.**

**Name:**

**Gender:** **M**

**Age:** 62

**Department:**

**Patient ID:**

**Inpatient No.:**

emergency department

**Bed No.:**

**Specimen Type: Blood**

**Clinical Diagnosis:**

**Sample No.:** **201019211**

**Result:** ***Candida glabrata***

Antibiotic

　Amphotericin B

Fluconazole

MIC AST Result Interpretation

<0.5

=16 I

Breakpoint

8-64

Itraconazole =0.5

Voriconazole =0.5

Note: S: Sensitive,R: Resistant, I: Intermediat

Identification Method: MOLDI TOF

Specimen submission time:2020.10.19 20:33 Report Release Time:2020.10.26 9:34 Laboratory Technician: Report Reviewer: Laboratory test order time:0000.00.00.00 Ordering Physician: xxx

**This report applies solely to the submitted specimen. For inquiries regarding test results, please contact the Laboratory prior to 17:00 on working days.**

**Name:**

**Gender:** **M**

**Age:** 62

**Department:**

**Patient ID:**

**Inpatient No.:**

emergency department

**Bed No.:**

**Specimen Type: Blood**

**Clinical Diagnosis:**

**Sample No.:** **201020204**

**Result:** ***Candida glabrata***

Antibiotic

　Amphotericin B

Fluconazole

MIC AST Result Interpretation

<0.5

=16 I

Breakpoint

8-64

Itraconazole =0.5

Voriconazole =0.5

Note: S: Sensitive,R: Resistant, I: Intermediat

Identification Method: MOLDI TOF

Specimen submission time:2020.10.20 16:19 Report Release Time: 2020.10.25 15:17 Laboratory Technician: Report Reviewer: Laboratory test order time:2020.10.20 13:12 Ordering Physician:xxx

**This report applies solely to the submitted specimen. For inquiries regarding test results, please contact the Laboratory prior to 17:00 on working days.**

**Name:**

**Gender:** **M**

**Age:** 67

**Department:**

**Patient ID:**

**Inpatient No.:**

emergency department

**Bed No.:**

**Specimen Type: Blood**

**Clinical Diagnosis:**

**Sample No.:** **201108205**

**Result:** ***Candida albicans***

Antibiotic

　Amphotericin B

Fluconazole

Itraconazole

Voriconazole

MIC AST Result Interpretation

<0.5

<1 S <0.12

<0.06 S

Breakpoint

2-8

1-4

Note: S: Sensitive,R: Resistant, I: Intermediat

Identification Method: MOLDI TOF

Specimen submission time:2020.11.8 20:36 Report Release Time: 2020.11.12 8:38Laboratory Technician: Report Reviewer: Laboratory test order time:2020.11.8 18:45 Ordering Physician:xxx

**This report applies solely to the submitted specimen. For inquiries regarding test results, please contact the Laboratory prior to 17:00 on working days.**

**Name:**

**Gender:** **M**

**Age:** 47

**Department:**

**Patient ID:**

**Inpatient No.:**

**Surgical Department**

**Bed No.:**

**Specimen Type: Blood**

**Clinical Diagnosis:**

**Sample No.:** **201118206**

**Result:** ***Candida albicans***

Antibiotic

　Amphotericin B

Fluconazole

MIC AST Result Interpretation

=4

<1 S

Breakpoint

2-8

Itraconazole <0.12

Voriconazole <0.06 S 1-4

Note: S: Sensitive,R: Resistant, I: Intermediat

Identification Method: MOLDI TOF

Specimen submission time:2020.11.28 16:06 Report Release Time:2020.11.22 09:07 Laboratory Technician: Report Reviewer: Laboratory test order time:2020.11.28 13:53 Ordering Physician:xxx

**This report applies solely to the submitted specimen. For inquiries regarding test results, please contact the Laboratory prior to 17:00 on working days.**

**Name:**

**Gender:** **F**

**Age:** 62

**Department:**

**Patient ID:**

**Inpatient No.:**

**Surgical Department**

**Bed No.:**

**Specimen Type: Blood**

**Clinical Diagnosis:**

**Sample No.:** **201121206**

**Result:** ***Candida albicans***

Antibiotic

　Amphotericin B

Fluconazole

MIC AST Result Interpretation

<0.5

<1 S

Breakpoint

8-64

Itraconazole <0.5

Voriconazole <0.06 S 1-4

Note: S: Sensitive,R: Resistant, I: Intermediat

Identification Method: MOLDI TOF

Specimen submission time:2020.11.21 19:51 Report Release Time:2020.11.28 10:33 Laboratory Technician: Report Reviewer: Laboratory test order time:2020.11.21 14:47 Ordering Physician:xxx

**This report applies solely to the submitted specimen. For inquiries regarding test results, please contact the Laboratory prior to 17:00 on working days.**

**Name:** 　 **Gender: M**

**Age:** 38

**Department:** **EICU Patient ID:**

**Inpatient No.:**

**Bed No.:** 　 **Specimen Type: Blood**

**Clinical Diagnosis:**

**Sample No.:** **201202203**

**Result:** ***Candida albicans***

Antibiotic

　Amphotericin B

Fluconazole

MIC AST Result Interpretation

<0.5

=2 S

Breakpoint

8-64

Itraconazole 0.125

Voriconazole =0.25 S 1-4

Note: S: Sensitive,R: Resistant, I: Intermediat

Identification Method: MOLDI TOF

Specimen submission time:2020.12.2 16:28 Report Release Time: 2020.12.9 8:34Laboratory Technician: Report Reviewer: Laboratory test order time:2020.12.2 15:22 Ordering Physician:xxx

**This report applies solely to the submitted specimen. For inquiries regarding test results, please contact the Laboratory prior to 17:00 on working days.**

**Name:**

**Gender:** **M**

**Age:** 61

**Department:**

**Patient ID:**

**Inpatient No.:**

**Surgical Department**

**Bed No.:**

**Specimen Type: Blood**

**Clinical Diagnosis:**

**Sample No.:** **201206206**

**Result:** ***Candida albicans***

Antibiotic

　Amphotericin B

Fluconazole

MIC AST Result Interpretation

<0.5

≤0.5 S

Breakpoint

8-64

Itraconazole ≤0.12

Voriconazole ≤1 S 1-4

Note: S: Sensitive,R: Resistant, I: Intermediat

Identification Method: MOLDI TOF

Specimen submission time:2020.12.6 17:02 Report Release Time:2020.12.10 9:30 Laboratory Technician: Report Reviewer: Laboratory test order time:2020.12.06 15:02 Ordering Physician:xxx

**This report applies solely to the submitted specimen. For inquiries regarding test results, please contact the Laboratory prior to 17:00 on working days.**

**Name:** 　 **Gender: M**

**Age:** 55

**Department: Patient ID:**

**Inpatient No.:**

internal Medicine

**Bed No.:** 　 **Specimen Type: Blood**

**Clinical Diagnosis:**

**Sample No.:** **201210208**

**Result:** ***Candida tropicalis***

Antibiotic

　Amphotericin B

Fluconazole

MIC AST Result Interpretation

=1

≥256 R

Breakpoint

2-8

Itraconazole ≥8

Voriconazole ≤0.12 S 0.125-1

Note: S: Sensitive,R: Resistant, I: Intermediat

Identification Method: MOLDI TOF

Specimen submission time:2020.12.10 14:26 Report Release Time:2020.12.13 9:33 Laboratory Technician: Report Reviewer: Laboratory test order time:2020.12.10 11:48 Ordering Physician: xxx

**This report applies solely to the submitted specimen. For inquiries regarding test results, please contact the Laboratory prior to 17:00 on working days.**

**Name:**

**Gender:** **F**

**Age:** 50

**Department:**

**Patient ID:**

**Inpatient No.:**

**Surgical Department**

**Bed No.:**

**Specimen Type: Blood**

**Clinical Diagnosis:**

**Sample No.:** **201214211**

**Result:** ***Candida albicans***

Antibiotic

　Amphotericin B

Fluconazole

MIC AST Result Interpretation

<0.5

<1 S

Breakpoint

8-64

Itraconazole <0.12

Voriconazole <0.06 S 1-4

Note: S: Sensitive,R: Resistant, I: Intermediat

Identification Method: MOLDI TOF

Specimen submission time:2020.12.14 23:46 Report Release Time:2020.12.18 9:40 Laboratory Technician: Report Reviewer: Laboratory test order time:2020.12.14 22:12 Ordering Physician: xxx

**This report applies solely to the submitted specimen. For inquiries regarding test results, please contact the Laboratory prior to 17:00 on working days.**

**Name:**

**Gender:** **F**

**Age:** 50

**Department:**

**Patient ID:**

**Inpatient No.:**

**Surgical Department**

**Bed No.:**

**Specimen Type: Blood**

**Clinical Diagnosis:**

**Sample No.:** **201215211**

**Result:** ***Candida albicans***

Antibiotic

　Amphotericin B

Fluconazole

MIC AST Result Interpretation

<0.5

<1 S

Breakpoint

8-64

Itraconazole <0.12

Voriconazole <0.06 S 1-4

Note: S: Sensitive,R: Resistant, I: Intermediat

Identification Method: MOLDI TOF

Specimen submission time:2020.12.15 19:55 Report Release Time:2020.12.19 8:43 Laboratory Techician: Report Reviewer: Laboratory test order time:2020 12.15 13:44 Ordering Physician:xxx

**This report applies solely to the submitted specimen. For inquiries regarding test results, please contact the Laboratory prior to 17:00 on working days.**

**Name:**

**Gender:** **F**

**Age:** 50

**Department:**

**Patient ID:**

**Inpatient No.:**

**Surgical Department**

**Bed No.:**

**Specimen Type: Blood**

**Clinical Diagnosis:**

**Sample No.:** **201224202**

**Result:** ***Candida albicans***

Antibiotic

　Amphotericin B

Fluconazole

MIC AST Result Interpretation

0.5

8 S

Breakpoint

8-64

Itraconazole 0.125

Voriconazole 1 S 1-4

Note: S: Sensitive,R: Resistant, I: Intermediat

Identification Method: MOLDI TOF

Specimen submission time:2020.12.24 9:24 Report Release Time:2020.12.28.10:53 Laboratory Tehnician: Report Reviewer: Laboratory test order time:2020.12.24 7:49 Ordering Physician:xxx

**This report applies solely to the submitted specimen. For inquiries regarding test results, please contact the Laboratory prior to 17:00 on working days.**

**Name:** 　 **Gender:** **F**

**Age:** 76

**Department:** **ICU Patient ID:**

**Inpatient No.:**

**Bed No.:** 　 **Specimen Type: Blood**

**Clinical Diagnosis:**

**Sample No.:** **210203202**

**Result:** ***Candida albicans***

Antibiotic

　Amphotericin B Fluconazole Voriconazole

Itraconazole

MIC AST Result Interpretation

=1

<1 S <0.06 S

<0.12

Breakpoint

8-64 1-4

Note: S: Sensitive,R: Resistant, I: Intermediat

Identification Method: MOLDI TOF

Specimen submission time:2021.2.3 00:57 Report Release Time: 2021.2.7 8:38 Laboratory Technician: Report Reviewer: Laboratory test order time: 2021.2.2 23:59 Ordering Physician:xxx

**This report applies solely to the submitted specimen. For inquiries regarding test results, please contact the Laboratory prior to 17:00 on working days.**

**Name:** 　 **Gender:** **F**

**Age:** 70

**Department:** **ICU Patient ID:**

**Inpatient No.:**

**Bed No.:** 　 **Specimen Type: Blood**

**Clinical Diagnosis:**

**Sample No.:** **210105204**

**Result:** ***Candida albicans***

Antibiotic

　Amphotericin B

Fluconazole

MIC AST Result Interpretation

<0.5

<1 S

Breakpoint

8-64

Itraconazole <0.12

Voriconazole <0.06 S 1-4

Note: S: Sensitive,R: Resistant, I: Intermediat

Identification Method: MOLDI TOF

Specimen submission time: 2021.1.5 12:17 Report Release Time:2021.1.9 12:01 Laboratory Technician: Report Reviewer: Laboratory test order time:2021.1.5 10:13 Ordering Physician:xxx

**This report applies solely to the submitted specimen. For inquiries regarding test results, please contact the Laboratory prior to 17:00 on working days.**

**Name:** 　 **Gender: M**

**Age:** 42

**Department:** **EICU Patient ID:**

**Inpatient No.:**

**Bed No.:** 　 **Specimen Type: Blood**

**Clinical Diagnosis:**

**Sample No.:** **210107201**

**Result:** ***Candida parapsilosis***

Antibiotic

　Amphotericin B

Fluconazole

MIC AST Result Interpretation

<0.5

<1 S

Breakpoint

2-8

Itraconazole <0.12

Voriconazole <0.06 S 1-4

Note: S: Sensitive,R: Resistant, I: Intermediat

Identification Method: MOLDI TOF

Specimen submission time: 2021.1.7 8:40 Report Release Time:2021.1.10 11:34 Laboratory Technician: Report Reviewer: Laboratory test order time:2021.1.7 6:00 ering Physician:xxx

**This report applies solely to the submitted specimen. For inquiries regarding test results, please contact the Laboratory prior to 17:00 on working days.**

**Name:** 　 **Gender:** **F**

**Age:** 70

**Department:** **ICU Patient ID:**

**Inpatient No.:**

**Bed No.:** 　 **Specimen Type: Blood**

**Clinical Diagnosis:**

**Sample No.:** **210107202**

**Result:** ***Candida albicans***

Antibiotic

　Amphotericin B

Fluconazole

MIC AST Result Interpretation

<0.5

<1 S

Breakpoint

8-64

Itraconazole <0.12

Voriconazole <0.06 S 1-4

Note: S: Sensitive,R: Resistant, I: Intermediat

Identification Method: MOLDI TOF

Specimen submission time: 2021.1.7 11:51 Report Release Time: 2021.1.10 11:33 Laboratory Technician: Report Reviewer: Laboratory test order time: 2021.1.7 10:14 Ordering Physician:xxx

**This report applies solely to the submitted specimen. For inquiries regarding test results, please contact the Laboratory prior to 17:00 on working days.**

**Name:** 　 **Gender: M**

**Age:** 47

**Department:** **ICU Patient ID:**

**Inpatient No.:**

**Bed No.:** 　 **Specimen Type: Blood**

**Clinical Diagnosis:**

**Sample No.:** **210115205**

**Result:** ***Candida parapsilosis***

Antibiotic

　Amphotericin B

Fluconazole

MIC AST Result Interpretation

<0.5

<1 S

Breakpoint

2-8

Itraconazole <0.12

Voriconazole <0.06 S 0.125-1

Note: S: Sensitive,R: Resistant, I: Intermediat

Identification Method: MOLDI TOF

Specimen submission time:2021.1.15 21:23 Report Release Time: 2021.1.19 9:20 Laboratory Technician: Report Reviewer: Laboratory test order time:2021.1.15 9:51 Ordering Physician:xxx

**This report applies solely to the submitted specimen. For inquiries regarding test results, please contact the Laboratory prior to 17:00 on working days.**

**Name:** 　 **Gender: M**

**Age:** 47

**Department:** **ICU Patient ID:**

**Inpatient No.:**

**Bed No.:** 　 **Specimen Type: Blood**

**Clinical Diagnosis:**

**Sample No.:** **210115533**

**Result:** ***Candida parapsilosis***

Antibiotic

　Amphotericin B

Fluconazole

MIC AST Result Interpretation

<0.5

<1 S

Breakpoint

2-8

Itraconazole <0.12

Voriconazole <0.06 S 0.125-1

Note: S: Sensitive,R: Resistant, I: Intermediat

Identification Method: MOLDI TOF

Specimen submission time: 2021.1.15 21:22 Report Release Time: 2021.1.19 9:21 Laboratory Technician: Report Reviewer: Laboratory test order time: 2021.1.15 14:49 Ordering Physician:xxx

**This report applies solely to the submitted specimen. For inquiries regarding test results, please contact the Laboratory prior to 17:00 on working days.**

**Name:** 　 **Gender:** **F**

**Age:** 76

**Department:** **ICU Patient ID:**

**Inpatient No.:**

**Bed No.:** 　 **Specimen Type: Blood**

**Clinical Diagnosis:**

**Sample No.:** **210104210**

**Result:** ***Candida albicans***

Antibiotic

　Amphotericin B

Fluconazole

MIC AST Result Interpretation

<0.5

<1 S

Breakpoint

8-64

Itraconazole <0.12

Voriconazole <0.06 S 1-4

Note: S: Sensitive,R: Resistant, I: Intermediat

Identification Method: MOLDI TOF

Specimen submission time: 2021.1.4 18:03 Report Release Time: 2021.1.11 9:31 Laboratory Technician: Report Reviewer: Laboratory test order time:2021.1.4 16:22 Ordering Physician:xxx

**This report applies solely to the submitted specimen. For inquiries regarding test results, please contact the Laboratory prior to 17:00 on working days.**

**Name:** 　 **Gender:** **F**

**Age:** 76

**Department:** **ICU Patient ID:**

**Inpatient No.:**

**Bed No.:** 　 **Specimen Type: Blood**

**Clinical Diagnosis:**

**Sample No.:** **210215201**

**Result:** ***Candida albicans***

Antibiotic

　Amphotericin B

Fluconazole

MIC AST Result Interpretation

=1

<1 S

Breakpoint

8-64

Itraconazole <0.12

Voriconazole <0.06 S 1-4

Note: S: Sensitive,R: Resistant, I: Intermediat

Identification Method: MOLDI TOF

Specimen submission time: 2021.2.15 8:03 Report Release Time: 2021.2.19 10:56 Laboratory Technician: Report Reviewer: Laboratory test order time:2021.2.14 23:00 Ordering Physician:xxx

**This report applies solely to the submitted specimen. For inquiries regarding test results, please contact the Laboratory prior to 17:00 on working days.**

**Name:** 　 **Gender:** **F**

**Age:** 76

**Department:** **ICU Patient ID:**

**Inpatient No.:**

**Bed No.:** 　 **Specimen Type: Blood**

**Clinical Diagnosis:**

**Sample No.:** **210217206**

**Result:** ***Candida albicans***

Antibiotic

　Amphotericin B

Fluconazole

MIC AST Result Interpretation

=1

<1 S

Breakpoint

8-64

Itraconazole <0.12

Voriconazole <0.06 S 1-4

Note: S: Sensitive,R: Resistant, I: Intermediat

Identification Method: MOLDI TOF

Specimen submission time: 2021.2.17 17:19 Report Release Time:2021.2.21 8:17 Laboratory Technician: Report Reviewer: Laboratory test order time:2021.2.17 14:04 Ordering Physician:xxx

**This report applies solely to the submitted specimen. For inquiries regarding test results, please contact the Laboratory prior to 17:00 on working days.**

**Name:**

**Gender:** **M**

**Age:** 74

**Department:**

**Patient ID:**

**Inpatient No.:**

**Surgical Department**

**Bed No.:**

**Specimen Type: Blood**

**Clinical Diagnosis:**

**Sample No.:** **210228203**

**Result:** ***Candida albicans***

Antibiotic

　Amphotericin B

Fluconazole

MIC AST Result Interpretation

<0.5

<1 S

Breakpoint

8-64

Itraconazole <0.12

Voriconazole <0.5 S 1-4

Note: S: Sensitive,R: Resistant, I: Intermediat

Identification Method: MOLDI TOF

Specimen submission time: 2021.2.28 1:45 Report Release Time:2021.3.4 9:49 Laboratory Technician: Report Reviewer: Laboratory test order time:2021.2.27 22:45 Ordering Physician:xxx

**This report applies solely to the submitted specimen. For inquiries regarding test results, please contact the Laboratory prior to 17:00 on working days.**

**Name:** 　 **Gender:** **F**

**Age:** 76

**Department:** **ICU Patient ID:**

**Inpatient No.:**

**Bed No.:** 　 **Specimen Type: Blood**

**Clinical Diagnosis:**

**Sample No.:** **210304211**

**Result:** ***Candida albicans***

Antibiotic

　Amphotericin B

Fluconazole

MIC AST Result Interpretation

=1

<1 S

Breakpoint

8-64

Itraconazole <0.12

Voriconazole <0.06 S 1-4

Note: S: Sensitive,R: Resistant, I: Intermediat

Identification Method: MOLDI TOF

Specimen submission time:2021.3.4 19:16 Report Release Time: 2021.3.9 09:03 Laboratory Technician: Report Reviewer: Laboratory test order time: 2021.3.4 17:19 Ordering Physician:xxx

**This report applies solely to the submitted specimen. For inquiries regarding test results, please contact the Laboratory prior to 17:00 on working days.**

**Name:**

**Gender:** **M**

**Age:** 56

**Department:**

**Patient ID:**

**Inpatient No.:**

**Surgical Department**

**Bed No.:**

**Specimen Type: Blood**

**Clinical Diagnosis:**

**Sample No.:** **210312216**

**Result:** ***Candida parapsilosis***

Antibiotic

　Amphotericin B

Fluconazole

MIC AST Result Interpretation

<0.5

<1 S

Breakpoint

2-8

Itraconazole <0.12

Voriconazole <0.06 S 0.125-1

Note: S: Sensitive,R: Resistant, I: Intermediat

Identification Method: MOLDI TOF

Specimen submission time:2021.3.12 21:18 Report Release Time:2021.3.16 8:55 Laboratory Technician: Report Reviewer: Laboratory test order time:2021.3.12 19:00 Ordering Physician:xxx

**This report applies solely to the submitted specimen. For inquiries regarding test results, please contact the Laboratory prior to 17:00 on working days.**

**Name:**

**Gender:** **M**

**Age:** 56

**Department:**

**Patient ID:**

**Inpatient No.:**

**Surgical Department**

**Bed No.:**

**Specimen Type: Blood**

**Clinical Diagnosis:**

**Sample No.:** **210312511**

**Result:** ***Candida parapsilosis***

Antibiotic

　Amphotericin B

Fluconazole

MIC AST Result Interpretation

<0.5

<1 S

Breakpoint

2-8

Itraconazole <0.12

Voriconazole <0.06 S 0.125-1

Note: S: Sensitive,R: Resistant, I: Intermediat

Identification Method: MOLDI TOF

Specimen submission time: 2021.3.12 14:56 Report Release Time:2021.3.15 9:20 Laboratory Technician: Report Reviewer: Laboratory test order time:2021.3.11 10:25 Ordering Physician:xxx

**This report applies solely to the submitted specimen. For inquiries regarding test results, please contact the Laboratory prior to 17:00 on working days.**

**Name:**

**Gender:** **M**

**Age:** 74

**Department:**

**Patient ID:**

**Inpatient No.:**

**Surgical Department**

**Bed No.:c27**

**Specimen Type:Blood**

**Clinical Diagnosis:**

**Sample No.:** **210423205**

**Result:** ***Candida albicans***

Antibiotic

　Amphotericin B

Fluconazole

MIC AST Result Interpretation

<0.5

<1 S

Breakpoint

8-64

Itraconazole <0.12

Voriconazole <0.06 S 1-4

Note: S: Sensitive,R: Resistant, I: Intermediat

Identification Method: MOLDI TOF

Specimen submission time:2021.4.23 22:47 Report Release Time:2021.4.30 9:36 Laboratory Technician: Report Reviewer: Laboratory test order time:2021.4.23 20:42 Ordering Physician:xxx

**This report applies solely to the submitted specimen. For inquiries regarding test results, please contact the Laboratory prior to 17:00 on working days.**

**Name:**

**Gender:** **M**

**Age:** 74

**Department:**

**Patient ID:**

**Inpatient No.:**

**Surgical Department**

**Bed No.:**

**Specimen Type: Blood**

**Clinical Diagnosis:**

**Sample No.:** **210414503**

**Result:** ***Candida albicans***

Antibiotic

　Amphotericin B

Fluconazole

MIC AST Result Interpretation

<0.5

<1 S

Breakpoint

8-64

Itraconazole <0.12

Voriconazole <0.06 S 1-4

Note: S: Sensitive,R: Resistant, I: Intermediat

Identification Method: MOLDI TOF

Specimen submission time:2021.4.14 07:56 Report Release Time:2021.4.16 10:20 Laboratory Technician: Report Reviewer: Laboratory test order time:2021.4.13 15:35 Ordering Physician:xxx

**This report applies solely to the submitted specimen. For inquiries regarding test results, please contact the Laboratory prior to 17:00 on working days.**

**Name:**

**Gender:** **M**

**Age:** 74

**Department:**

**Patient ID:**

**Inpatient No.:**

**Surgical Department**

**Bed No.:**

**Specimen Type: Blood**

**Clinical Diagnosis:**

**Sample No.:** **210411208**

**Result:** ***Candida albicans***

Antibiotic

　Amphotericin B Fluconazole Itraconazole

Voriconazole

MIC AST Result Interpretation

<0.5

<1 S <0.12

<0.06 S

Breakpoint

　 8-64

1-4

Note: S: Sensitive,R: Resistant, I: Intermediat

Identification Method: MOLDI TOF

Specimen submission time:2021.4.11 20:30 Report Release Time:2021.4.16 10:20 Laboratory Technician: Report Reviewer: Laboratory test order time:2021.4.11 09:39 Ordering Physician:xxx

**This report applies solely to the submitted specimen. For inquiries regarding test results, please contact the Laboratory prior to 17:00 on working days.**

**Name:** 　 **Gender: M**

**Age:** 69

**Department:** **ICU Patient ID:**

**Inpatient No.:**

**Bed No.:** 　 **Specimen Type: Blood**

**Clinical Diagnosis:**

**Sample No.:** **210522201**

**Result:** ***Candida parapsilosis***

Antibiotic

　Amphotericin B

Fluconazole

MIC AST Result Interpretation

<0.5

<1 S

Breakpoint

2-8

Itraconazole <0.12

Voriconazole <0.06 S 0.125-1

Note: S: Sensitive,R: Resistant, I: Intermediat

Identification Method: MOLDI TOF

Specimen submission time: 2021.5.22 16:38 Report Release Time:2021.5.26 08:31 Laboratory Technician: Report Reviewer: Laboratory test order time:2021.5.22 14:56 Ordering Physician:xxx

**This report applies solely to the submitted specimen. For inquiries regarding test results, please contact the Laboratory prior to 17:00 on working days.**

**Name:** 　 **Gender: M**

**Age:** 69

**Department:** **ICU Patient ID:**

**Inpatient No.:**

**Bed No.:** 　 **Specimen Type: Blood**

**Clinical Diagnosis:**

**Sample No.:** **200513211**

**Result:** ***Candida parapsilosis***

Antibiotic

　Amphotericin B

Fluconazole

MIC AST Result Interpretation

<0.5

<1 S

Breakpoint

2-8

Itraconazole <0.12

Voriconazole <0.06 S 0.125-1

Note: S: Sensitive,R: Resistant, I: Intermediat

Identification Method: MOLDI TOF

Specimen submission time:2020.5.13 20:01 Report Release Time:2020.5.17 08:42 Laboratory Technician: Report Reviewer: Laboratory test order time:2020.5.13 18:24 Ordering Physician:xxx

**This report applies solely to the submitted specimen. For inquiries regarding test results, please contact the Laboratory prior to 17:00 on working days.**

**Name:** 　 **Gender: M**

**Age:** 69

**Department:** **ICU Patient ID:**

**Inpatient No.:**

**Bed No.:** 　 **Specimen Type: Blood**

**Clinical Diagnosis:**

**Sample No.:** **200509208**

**Result:** ***Candida parapsilosis***

Antibiotic

　Amphotericin B

Fluconazole

MIC AST Result Interpretation

<0.5

<1 S

Breakpoint

2-8

Itraconazole <0.12

Voriconazole <0.06 S 0.125-1

Note: S: Sensitive,R: Resistant, I: Intermediat

Identification Method: MOLDI TOF

Specimen submission time:2020.5.09 11:02 Report Release Time:2020.5.13 08:48 Laboratory Technician: Report Reviewer: Laboratory test order time:2020.5.09 09:21 Ordering Physician:xxx

**This report applies solely to the submitted specimen. For inquiries regarding test results, please contact the Laboratory prior to 17:00 on working days.**

**Name:** 　 **Gender: m**

**Age:** 69

**Department:** **ICU Patient ID:**

**Inpatient No.:**

**Bed No.:** 　 **Specimen Type: Blood**

**Clinical Diagnosis:**

**Sample No.:** **220510209**

**Result:** ***Candida parapsilosis***

Antibiotic

　Amphotericin B

Fluconazole

Itraconazole

Voriconazole

MIC AST Result Interpretation

<0.5 WT

<1 S

<0.12 WT

<0.06 S

Breakpoint

　1

2-8

0.5

0.125-1

Note: S: Sensitive,R: Resistant, I: Intermediat, nWT: non-Wild Type, WT: Wild Type

Identification Method: MOLDI TOF

Specimen submission time:2022.5.10 20:09 Report Release Time:2022.5.14 10:48 Laboratory Technician: Report Reviewer: Laboratory test order time:2022.5.10 15:41 Ordering Physician:xxx

**This report applies solely to the submitted specimen. For inquiries regarding test results, please contact the Laboratory prior to 17:00 on working days.**

**Name:** 　 **Gender: M**

**Age:** 69

**Department:** **ICU Patient ID:**

**Inpatient No.:**

**Bed No.:** 　 **Specimen Type: Blood**

**Clinical Diagnosis:**

**Sample No.:** **220505203**

**Result:** ***Candida parapsilosis***

Antibiotic

　Amphotericin B

Fluconazole

Itraconazole

Voriconazole

MIC AST Result Interpretation

<0.5 WT

<1 S

<0.12 WT

<0.06 S

Breakpoint

　1

2-8

0.5

0.125-1

Note: S: Sensitive,R: Resistant, I: Intermediat, nWT: non-Wild Type, WT: Wild Type

Identification Method: MOLDI TOF

Specimen submission time:2022.5.05 16:26 Report Release Time:2022.5.11 9:37 Laboratory Technician: Report Reviewer: Laboratory test order time:2022.5.05 12:09 Ordering Physician: xxx

**This report applies solely to the submitted specimen. For inquiries regarding test results, please contact the Laboratory prior to 17:00 on working days.**

**Name:**

**Gender:** **F**

**Age:** 70

**Department:**

**Patient ID:**

**Inpatient No.:**

surgical department

**Bed No.:**

**Specimen Type: Blood**

**Clinical Diagnosis:**

**Sample No.:** **220616213**

**Result:** ***Candida albicans***

Antibiotic

　Amphotericin B

Fluconazole

MIC AST Result Interpretation

<0.5 WT

<1 S

Breakpoint

2

2-8

Itraconazole <0.12

Voriconazole <0.06 S 1-4

Note: S: Sensitive,R: Resistant, I: Intermediat, nWT: non-Wild Type, WT: Wild Type

Identification Method: MOLDI TOF

Specimen submission time:2022.06.16 21:20 Report Release Time: 2022.06.22 11:14 Laboratory Technician: Report Reviewer: Laboratory test order time: 2022.06.16 19:44 Ordering Physician:xxx

**This report applies solely to the submitted specimen. For inquiries regarding test results, please contact the Laboratory prior to 17:00 on working days.**

**Name:**

**Gender:** **F**

**Age:** 70

**Department:**

**Patient ID:**

**Inpatient No.:**

**Surgical Department**

**Bed No.:**

**Specimen Type: Blood**

**Clinical Diagnosis:**

**Sample No.:** **220612202**

**Result:** ***Candida albicans***

Antibiotic

　Amphotericin B

Fluconazole

MIC AST Result Interpretation

<0.5 WT

<1 S

Breakpoint

　2

2-8

Itraconazole <0.12

Voriconazole <0.06 S 1-4

Note: S: Sensitive,R: Resistant, I: Intermediat, nWT: non-Wild Type, WT: Wild Type

Identification Method: MOLDI TOF

Specimen submission time: 2022.06.12 06:31 Report Release Time: 2022.06.15 09:36 Laboratory Technician: Report Reviewer: Laboratory test order time: 2022.06.12 02:03 Ordering Physician: xxx

**This report applies solely to the submitted specimen. For inquiries regarding test results, please contact the Laboratory prior to 17:00 on working days.**

**Name:**

**Gender:** **F**

**Age:** 54

**Department:**

**Patient ID:**

**Inpatient No.:**

**Surgical Department**

**Bed No.:**

**Specimen Type: Blood**

**Clinical Diagnosis:**

**Sample No.:** **220620205**

**Result:** ***Candida albicans***

Antibiotic

　Amphotericin B

Fluconazole

MIC AST Result Interpretation

<0.5 WT

<1 S

Breakpoint

2

2-8

Itraconazole <0.12

Voriconazole <0.06 S 1-4

Note: S: Sensitive,R: Resistant, I: Intermediat, nWT: non-Wild Type, WT: Wild Type

Identification Method: MOLDI TOF

Specimen submission time: 2022.06.20 01:02 Report Release Time: 2022.06.23 10:01 Laboratory Technician: Report Reviewer: Laboratory test order time: 2022.06.19 21:42 Ordering Physician:xxx

**This report applies solely to the submitted specimen. For inquiries regarding test results, please contact the Laboratory prior to 17:00 on working days.**

**Name:** 　 **Gender:** **f**

**Age:** 79

**Department:** **EICU Patient ID:**

**Inpatient No.:**

**Bed No.:** 　 **Specimen Type: Blood**

**Clinical Diagnosis:**

**Sample No.:** **220626203**

**Result:** ***Candida tropicalis***

Antibiotic

　Amphotericin B

Fluconazole

Itraconazole

Voriconazole

MIC AST Result Interpretation

≤0.5 WT

≤2 S

≤0.12 WT

≤0.06 S

Breakpoint

　2

2-8 0.5

0.125-1

Note: S: Sensitive,R: Resistant, I: Intermediate, nWT: non-Wild Type, WT: Wild Type

Identification Method: MOLDI TOF

Specimen submission time: 2022.06.26 19:25 Report Release Time:2022.06.29 10:05 Laboratory Technician: Report Reviewer: Laboratory test order time: 2022.06.26 18:32 Ordering Physician:xxx

**This report applies solely to the submitted specimen. For inquiries regarding test results, please contact the Laboratory prior to 17:00 on working days.**

**Name:**

**Gender:** **f**

**Age:** 82

**Department:**

**Patient ID:**

**Inpatient No.:**

**Surgical Department**

**Bed No.:**

**Specimen Type: Blood**

**Clinical Diagnosis:**

**Sample No.:** **220705205**

**Result:** ***Candida albicans***

Antibiotic

　Amphotericin B

Fluconazole

Itraconazole

Voriconazole

MIC AST Result Interpretation

<0.5 WT

<1 S

<0.12

<0.06 S

Breakpoint

2

8-64

0.12-1

Note: S: Sensitive,R: Resistant, I: Intermediat, nWT: non-Wild Type, WT: Wild Type

Identification Method: MOLDI TOF

Specimen submission time: 2022.07.05 16:04 Report Release Time: 2022.07.10 10:17 Laboratory Technician: Report Reviewer: Laboratory test order time: 2022.07.05 08:05 Ordering Physician:xxx

**This report applies solely to the submitted specimen. For inquiries regarding test results, please contact the Laboratory prior to 17:00 on working days.**

**Name:**

**Gender:** **F**

**Age:** 82

**Department:**

**Patient ID:**

**Inpatient No.:**

**Surgical Department**

**Bed No.:**

**Specimen Type: Blood**

**Clinical Diagnosis:**

**Sample No.:** **220706502**

**Result:** ***Candida albicans***

Antibiotic

　Amphotericin B

Fluconazole

Itraconazole

Voriconazole

MIC AST Result Interpretation

<0.5 WT

<1 S

<0.12 S

<0.06 S

Breakpoint

　2

2-8

0.12-1

Note: S: Sensitive,R: Resistant, I: Intermediat, nWT: non-Wild Type, WT: Wild Type

Identification Method: MOLDI TOF

Specimen submission time: 2022.07.06 07:52 Report Release Time: 2022.07.08 09:17 Laboratory Technician: Report Reviewer: Laboratory test order time: 2022.07.05 15:47 Ordering Physician:xxx

**This report applies solely to the submitted specimen. For inquiries regarding test results, please contact the Laboratory prior to 17:00 on working days.**

**Name:**

**Gender:** **m**

**Age:** 45

**Department:**

**Patient ID:**

**Inpatient No.:**

emergency department

**Bed No.:**

**Specimen Type: Blood**

**Clinical Diagnosis:**

**Sample No.:** **220719202**

**Result:** ***Candida parapsilosis***

Antibiotic

　Amphotericin B

Fluconazole

Itraconazole

Voriconazole

MIC AST Result Interpretation

<0.5 WT

<1 S

<0.12 WT

<0.06 S

Breakpoint

　1

2-8

0.5

0.125-1

Note: S: Sensitive,R: Resistant, I: Intermediat, nWT: non-Wild Type, WT: Wild Type

Identification Method: MOLDI TOF

Specimen submission time: 2022.07.19 03:23 Report Release Time:2022.07.23 11:43 Laboratory Technician: Report Reviewer: Laboratory test order time: 0000.00.00 00:00 Ordering Physician:xxx

**This report applies solely to the submitted specimen. For inquiries regarding test results, please contact the Laboratory prior to 17:00 on working days.**

**Name:** 　 **Gender:** **f**

**Age:** 58

**Department:** ICU **Patient ID:**

**Inpatient No.:**

**Bed No.:** 　 **Specimen Type: Blood**

**Clinical Diagnosis:**

**Sample No.:** **220729204**

**Result:** ***Candida albicans***

Antibiotic

　Amphotericin B

Fluconazole

Itraconazole

Voriconazole

MIC AST Result Interpretation

<0.5 WT

<1 S

<0.12 S

<0.06 S

Breakpoint

　2

2-8

0.12-1

Note: S: Sensitive,R: Resistant, I: Intermediat, nWT: non-Wild Type, WT: Wild Type

Identification Method: MOLDI TOF

Specimen submission time: 2022.07.29 10:55 Report Release Time: 2022.08.04 09:31 Laboratory Technician: Report Reviewer: Laboratory test order time:2022.07.29 10:15 Ordering Physician:xxx

**This report applies solely to the submitted specimen. For inquiries regarding test results, please contact the Laboratory prior to 17:00 on working days.**

**Name:**

**Gender:** **f**

**Age:** 58

**Department:**

**Patient ID:**

**Inpatient No.:**

surgical department

**Bed No.:**

**Specimen Type: Blood**

**Clinical Diagnosis:**

**Sample No.:** **220723207**

**Result:** ***Candida albicans***

Antibiotic

　Amphotericin B

Fluconazole

Itraconazole

Voriconazole

MIC AST Result Interpretation

<0.5 WT

<1 S

<0.12 S

<0.06 S

Breakpoint

　2

2-8

0.12-1

Note: S: Sensitive,R: Resistant, I: Intermediat，nWT: non-Wild Type, WT: Wild Type

Identification Method: MOLDI TOF

Specimen submission time: 2022.07.23 09:50 Report Release Time: 2022.07.27 14:24 Laboratory Technician: Report Reviewer: Laboratory test order time: 2022.07.23 08:59 Ordering Physician:xxx

**This report applies solely to the submitted specimen. For inquiries regarding test results, please contact the Laboratory prior to 17:00 on working days.**

**Name:** 　 **Gender:** **f**

**Age:** 57

**Department:** **INCU Patient ID:**

**Inpatient No.:**

**Bed No.:** 　 **Specimen Type: Blood**

**Clinical Diagnosis:**

**Sample No.:** **220803516**

**Result:** ***Candida albicans***

Antibiotic

　Amphotericin B

Fluconazole

Itraconazole

Voriconazole

MIC AST Result Interpretation

<0.5 WT

<1 S

<0.12 S

<0.06 S

Breakpoint

　2

2-8

0.12-1

Note: S: Sensitive,R: Resistant, I: Intermediat，nWT: non-Wild Type, WT: Wild Type

Identification Method: MOLDI TOF

Specimen submission time: 2022.08.03 15:41 Report Release Time: 2022.08.08 09:40 Laboratory Technician: Report Reviewer: Laboratory test order time: 2022.08.03 09:44 Ordering Physician: xxx

**This report applies solely to the submitted specimen. For inquiries regarding test results, please contact the Laboratory prior to 17:00 on working days.**

**Name:** 　 **Gender:** **f**

**Age:** 57

**Department:** **NICU Patient ID:**

**Inpatient No.:**

**Bed No.:** 　 **Specimen Type: Blood**

**Clinical Diagnosis:**

**Sample No.:** **220802525**

**Result:** ***Candida albicans***

Antibiotic

　Amphotericin B

Fluconazole

MIC AST Result Interpretation

<0.5 WT

<1 S

Breakpoint

2

2-8

Itraconazole <0.12

Voriconazole <0.06 S 0.12-1

Note: S: Sensitive,R: Resistant, I: Intermediat, nWT: non-Wild Type, WT: Wild Type

Identification Method: MOLDI TOF

Specimen submission time: 2022.08.02 20:32 Report Release Time:2022.08.09 10:40 Laboratory Technician: Report Reviewer: Laboratory test order time: 2022.08.02 16:35 Ordering Physician:xxx

**This report applies solely to the submitted specimen. For inquiries regarding test results, please contact the Laboratory prior to 17:00 on working days.**

**Name:** 　 **Gender:** **f**

**Age:** 57

**Department:** **NICU Patient ID:**

**Inpatient No.:**

**Bed No.:** 　 **Specimen Type: Blood**

**Clinical Diagnosis:**

**Sample No.:** **220802210**

**Result:** ***Candida albicans***

Antibiotic

　Amphotericin B

Fluconazole

MIC AST Result Interpretation

<0.5 WT

<1 S

Breakpoint

2

2-8

Itraconazole <0.12

Voriconazole <0.06 S 0.12-1

Note: S: Sensitive,R: Resistant, I: Intermediat nWT: non-Wild Type, WT: Wild Type

Identification Method: MOLDI TOF

Specimen submission time: 2022.08.02 20:31 Report Release Time: 2022.08.08 08:54 Laboratory Technician: Report Reviewer: Laboratory test order time: 2022.08.02 15:42 Ordering Physician:xxx

**This report applies solely to the submitted specimen. For inquiries regarding test results, please contact the Laboratory prior to 17:00 on working days.**

**Name:** 　 **Gender:** **F**

**Age:** 57

**Department:** **NICU Patient ID:**

**Inpatient No.:**

**Bed No.:** 　 **SpecimenType: Blood**

**Clinical Diagnosis:**

**Sample No.:** **220729213**

**Result:** ***Candida albicans***

Antibiotic

　Amphotericin B

Fluconazole

MIC AST Result Interpretation

<0.5 WT

<1 S

Breakpoint

2

2-8

Itraconazole <0.12

Voriconazole <0.06 S 0.125-1

Note: S: Sensitive,R: Resistant, I: Intermediat, nWT: non-Wild Type, WT: Wild Type

Identification Method: MOLDI TOF

Specimen submission time: 2022.07.29 19:06 Report Release Time: 2022.08.04 09:35 Laboratory Technician: Report Reviewer: Laboratory test order time:2022.07.29 16:13 Ordering Physician:xxx

**This report applies solely to the submitted specimen. For inquiries regarding test results, please contact the Laboratory prior to 17:00 on working days.**

**Name:**

**Gender:** **m**

**Age:** 69

**Department:**

**Patient ID:**

**Inpatient No.:**

**Surgical Department**

**Bed No.:**

**Specimen Type: Blood**

**Clinical Diagnosis:**

**Sample No.:** **220810215**

**Result:** ***Candida albicans***

Antibiotic

　Amphotericin B

Fluconazole

MIC AST Result Interpretation

<0.5 WT

<0.5 S

Breakpoint

2

2-8

Itraconazole <0.125

Voriconazole <0.062 S 0.125-1

Note: S: Sensitive,R: Resistant, I: Intermediat, nWT: non-Wild Type, WT: Wild Type

Identification Method: MOLDI TOF

Specimen submission time: 2022.08.10 22:02 Report Release Time: 2022.08.13 09:58 Laboratory Technician: Report Reviewer: Laboratory test order time: 2022.08.10 18:21 Ordering Physician:xxx

**This report applies solely to the submitted specimen. For inquiries regarding test results, please contact the Laboratory prior to 17:00 on working days.**

**Name:**

**Gender:** **m**

**Age:** 69

**Department:**

**Patient ID:**

**Inpatient No.:**

**Surgical Department**

**Bed No.:**

**Specimen Type:Blood**

**Clinical Diagnosis:**

**Sample No.:** **220811504**

**Result:** ***Candida albicans***

Antibiotic

　Amphotericin B

Fluconazole

MIC AST Result Interpretation

<0.5 WT

<0.5 S

Breakpoint

2

2-8

Itraconazole <0.125

Voriconazole <0.062 S 0.125-1

Note: S: Sensitive,R: Resistant, I: Intermediat, nWT: non-Wild Type, WT: Wild Type

Identification Method: MOLDI TOF

Specimen submission time: 2022.08.11 08:01 Report Release Time: 2022.08.13 09:53 Laboratory Technician: Report Reviewer: Laboratory test order time: 2022.08.10 20:04 Ordering Physician:xxx

**This report applies solely to the submitted specimen. For inquiries regarding test results, please contact the Laboratory prior to 17:00 on working days.**

**Name:** 　 **Gender:** **F**

**Age:** 70

**Department:** **ICU Patient ID:**

**Inpatient No.:**

**Bed No.:** 　 **Specimen Type: Blood**

**Clinical Diagnosis:**

**Sample No.:** **220818203**

**Result:** ***Candida albicans***

Antibiotic

　Amphotericin B

Fluconazole

MIC AST Result Interpretation

<0.5 WT

<1 S

Breakpoint

2

2-8

Itraconazole <0.12

Voriconazole <0.06 S 0.125-1

Note: S: Sensitive,R: Resistant, I: Intermediat, nWT: non-Wild Type, WT: Wild Type

Identification Method: MOLDI TOF

Specimen submission time: 2022.08.18 03:26 Report Release Time: 2022 08.22 09:27 Laboratory Technician: Report Reviewer: Laboratory test order time: 2022.08.18 01:15 Ordering Physician:xxx

**This report applies solely to the submitted specimen. For inquiries regarding test results, please contact the Laboratory prior to 17:00 on working days.**

**Name:** 　 **Gender: M**

**Age:** 86

**Department:** **NICU Patient ID:**

**Inpatient No.:**

**Bed No.:** 　 **Specimen Type: Blood**

**Clinical Diagnosis:**

**Sample No.:** **220818206**

**Result:** ***Candida parapsilosis***

Antibiotic

　Amphotericin B

Fluconazole

Itraconazole

Voriconazole

MIC AST Result Interpretation

<0.5 WT

<1 S

<0.12 WT

<0.06 S

Breakpoint

1

2-8

0.5

0.125-1

Note: S: Sensitive,R: Resistant, I: Intermediat, nWT: non-Wild Type, WT: Wild Type

Identification Method: MOLDI TOF

Specimen submission time: 2022.08.18 14:00 Report Release Time:2022.08.22 08:49 Laboratory Technician: Report Reviewer: Laboratory test order time: 2022.08.18 11:17 Ordering Physician: xxx

**This report applies solely to the submitted specimen. For inquiries regarding test results, please contact the Laboratory prior to 17:00 on working days.**

**Name:**

**Gender:** **f**

**Age:** 70

**Department:**

**Patient ID:**

**Inpatient No.:**

emergency department

**Bed No.:**

**Specimen Type: Blood**

**Clinical Diagnosis:**

**Sample No.:** **220818501**

**Result:** ***Candida albicans***

Antibiotic

　Amphotericin B

Fluconazole

MIC AST Result Interpretation

<0.5 WT

<1 S

Breakpoint

2

2-8

Itraconazole <0.12

Voriconazole <0.06 S 0.125-1

Note: S: Sensitive,R: Resistant, I: Intermediat, nWT: non-Wild Type, WT: Wild Type

Identification Method: MOLDI TOF

Specimen submission time: 2022.08.18 02:32 Report Release Time:2022.08.22 09:29 Laboratory Technician: Report Reviewer: Laboratory test order time: 2022.08.17 21:55 Ordering Physician:xxx

**This report applies solely to the submitted specimen. For inquiries regarding test results, please contact the Laboratory prior to 17:00 on working days.**

**Name:**

**Gender:** **F**

**Age:** 70

**Department:**

**Patient ID:**

**Inpatient No.:**

**emergency department**

**Bed No.:**

**Specimen Type: Blood**

**Clinical Diagnosis:**

**Sample No.:** **220818502**

**Result:** ***Candida albicans***

Antibiotic

　Amphotericin B

Fluconazole

MIC AST Result Interpretation

<0.5 WT

<1 S

Breakpoint

2

2-8

Itraconazole <0.12

Voriconazole <0.06 S 0.125-1

Note: S: Sensitive,R: Resistant, I: Intermediat, nWT: non-Wild Type, WT: Wild Type

Identification Method: MOLDI TOF

Specimen submission time: 2022.08.18 02:32 Report Release Time: 2022.08.21 12:01 Laboratory Technician: Report Reviewer: Laboratory test order time: 2022.08.17 21:57 Ordering Physician:xxx

**This report applies solely to the submitted specimen. For inquiries regarding test results, please contact the Laboratory prior to 17:00 on working days.**

**Name:** 　 **Gender: m**

**Age:** 86

**Department:** N **ICU Patient ID:**

**Inpatient No.:**

**Bed No.:** 　 **Specimen Type: Blood**

**Clinical Diagnosis:**

**Sample No.:** **220818522**

**Result:** ***Candida parapsilosis***

Antibiotic

　Amphotericin B

Fluconazole

Itraconazole

Voriconazole

MIC AST Result Interpretation

<0.5 WT

<1 S

<0.12 WT

<0.06 S

Breakpoint

1

2-8

0.5

0.125-1

Note: S: Sensitive,R: Resistant, I: Intermediat, nWT: non-Wild Type, WT: Wild Type

Identification Method: MOLDI TOF

Specimen submission time: 2022.08.18 13:56 Report Release Time: 2022.08.21 12:02 Laboratory Technician: Report Reviewer: Laboratory test order time: 2022.08.18 11:18 Ordering Physician:xxx

**This report applies solely to the submitted specimen. For inquiries regarding test results, please contact the Laboratory prior to 17:00 on working days.**

**Name:**

**Gender:** **M**

**Age:** 47

**Department:**

**Patient ID:**

**Inpatient No.:**

**Surgical Department**

**Bed No.:**

**Specimen Type: Blood**

**Clinical Diagnosis:**

**Sample No.:** **220819204**

**Result:** ***Candida albicans***

Antibiotic

　Amphotericin B

Fluconazole

MIC AST Result Interpretation

<0.5 WT

<1 S

Breakpoint

2

2-8

Itraconazole <0.12

Voriconazole <0.06 S 0.125-1

Note: S: Sensitive,R: Resistant, I: Intermediat, nWT: non-Wild Type, WT: Wild Type

Identification Method: MOLDI TOF

Specimen submission time: 2022.08.19 08:09 Report Release Time: 2022.08.23 09:28 Laboratory Technician: Report Reviewer: Laboratory test order time:2022.08.18 23:06 Ordering Physician:xxx

**This report applies solely to the submitted specimen. For inquiries regarding test results, please contact the Laboratory prior to 17:00 on working days.**

**Name:** 　 **Gender: m**

**Age:** 86

**Department:** **NICU Patient ID:**

**Inpatient No.:**

**Bed No.:** 　 **Specimen Type: Blood**

**Clinical Diagnosis:**

**Sample No.:** **220819209**

**Result:** ***Candida parapsilosis***

Antibiotic

　Amphotericin B

Fluconazole

Itraconazole

Voriconazole

MIC AST Result Interpretation

<0.5 WT

<1 S

<0.12 WT

<0.06 S

Breakpoint

1

2-8

0.5

0.125-1

Note: S: Sensitive,R: Resistant, I: Intermediat, nWT: non-Wild Type, WT: Wild Type

Identification Method: MOLDI TOF

Specimen submission time: 2022.08.19 14:02 Report Release Time: 2022.08.22 08:39 Laboratory Technician: Report Reviewer: Laboratory test order time: 2022.08.19 10:34 Ordering Physician:xxx

**This report applies solely to the submitted specimen. For inquiries regarding test results, please contact the Laboratory prior to 17:00 on working days.**

**Name:** 　 **Gender: M**

**Age:** 86

**Department:** **NICU Patient ID:**

**Inpatient No.:**

**Bed No.:** 　 **Specimen Type: Blood**

**Clinical Diagnosis:**

**Sample No.:** **220819515**

**Result:** ***Candida parapsilosis***

Antibiotic

　Amphotericin B

Fluconazole

Itraconazole

Voriconazole

MIC AST Result Interpretation

<0.5 WT

<1 S

<0.12 WT

<0.06 S

Breakpoint

1

2-8

0.5

0.125-1

Note: S: Sensitive,R: Resistant, I: Intermediat, nWT: non-Wild Type, WT: Wild Type

Identification Method: MOLDI TOF

Specimen submission time: 2022.08.19 14:03 Report Release Time: 2022.08.22 08:38 Laboratory Technician: Report Reviewer: Laboratory test order time: 2022.08.19 10:34 Ordering Physician:xxx

**This report applies solely to the submitted specimen. For inquiries regarding test results, please contact the Laboratory prior to 17:00 on working days.**

**Name:** 　 **Gender: M**

**Age:** 86

**Department:** **NICU Patient ID:**

**Inpatient No.:**

**Bed No.:** 　 **Specimen Type: Blood**

**Clinical Diagnosis:**

**Sample No.:** **220819519**

**Result:** ***Candida parapsilosis***

Antibiotic

　Amphotericin B

Fluconazole

Itraconazole

Voriconazole

MIC AST Result Interpretation

<0.5 WT

<1 S

<0.12 WT

<0.06 S

Breakpoint

1

2-8

0.5

0.125-1

Note: S: Sensitive,R: Resistant, I: Intermediat, nWT: non-Wild Type, WT: Wild Type

Identification Method: MOLDI TOF

Specimen submission time: 2022.08.1915:27 Report Release Time: 2022.08.22 08:39 Laboratory Technician: Report Reviewer: Laboratory test order time: 2022.08.19 13:58 Ordering Physician:xxx

**This report applies solely to the submitted specimen. For inquiries regarding test results, please contact the Laboratory prior to 17:00 on working days.**

**Name:**

**Gender:** **m**

**Age:** 60

**Department:**

**Patient ID:**

**Inpatient No.:**

emergency department

**Bed No.:**

**Specimen Type: Blood**

**Clinical Diagnosis:**

**Sample No.:** **220826202**

**Result:** ***Candida parapsilosis***

Antibiotic

　Amphotericin B

Fluconazole

Itraconazole

Voriconazole

MIC AST Result Interpretation

<0.5 WT

<1 S

<0.12 WT

<0.06 S

Breakpoint

1

2-8

0.5

0.125-1

Note: S: Sensitive,R: Resistant, I: Intermediat, nWT: non-Wild Type, WT: Wild Type

Identification Method: MOLDI TOF

Specimen submission time: 2022.08.26 00:43 Report Release Time: 2022.08.30 09:53 Laboratory Technician: Report Reviewer: Laboratory test order time: 2022.08.25 19:59 Ordering Physician:xxx

**This report applies solely to the submitted specimen. For inquiries regarding test results, please contact the Laboratory prior to 17:00 on working days.**

**Name:** 　 **Gender: M**

**Age:** 69

**Department:** **NICU Patient ID:**

**Inpatient No.:**

**Bed No.:** 　 **Specimen Type: Blood**

**Clinical Diagnosis:**

**Sample No.:** **220909526**

**Result:** ***Candida parapsilosis***

Antibiotic

　Amphotericin B

Fluconazole

Itraconazole

Voriconazole

MIC AST Result Interpretation

<0.5 WT

<1 S

<0.12 WT

<0.06 S

Breakpoint

1

2-8

0.5

0.125-1

Note: S: Sensitive,R: Resistant, I: Intermediat, nWT: non-Wild Type, WT: Wild Type

Identification Method: MOLDI TOF

Specimen submission time:2022.09.09 16:00 Report Release Time:2022 09.12 10:12 Laboratory Technician: Report Reviewer: Laboratory test order time: 2022.09.09 15:20 Ordering Physician:xxx

**This report applies solely to the submitted specimen. For inquiries regarding test results, please contact the Laboratory prior to 17:00 on working days.**

**Name:**

**Gender:** **F**

**Age:** 83

**Department:**

**Patient ID:**

**Inpatient No.:**

emergency department

**Bed No.:**

**Specimen Type: Blood**

**Clinical Diagnosis:**

**Sample No.:** **220923201**

**Result:** ***Candida albicans***

Antibiotic

　Amphotericin B

Fluconazole

Itraconazole

Voriconazole

MIC AST Result Interpretation

<0.5 WT

<1 S

<0.125 S

<0.06 S

Breakpoint

2

2-8

0.12-1

Note: S: Sensitive,R: Resistant, I: Intermediat, nWT: non-Wild Type, WT: Wild Type

Identification Method: MOLDI TOF

Specimen submission time: 2022.09.23 01:28 Report Release Time: 2022.09.27 08:53 Laboratory Technician: Report Reviewer: Laboratory test order time:0000.0.0 00:00 Ordering Physician: xxx

**This report applies solely to the submitted specimen. For inquiries regarding test results, please contact the Laboratory prior to 17:00 on working days.**

**Name:**

**Gender:** **f**

**Age:** 83

**Department:**

**Patient ID:**

**Inpatient No.:**

emergency department

**Bed No.:**

**Specimen Type: Blood**

**Clinical Diagnosis:**

**Sample No.:** **220925207**

**Result:** ***Candida albicans***

Antibiotic

　Amphotericin B

Fluconazole

MIC AST Result Interpretation

<0.5 WT

<1 S

Breakpoint

2

2-8

Itraconazole <0.125

Voriconazole <0.06 S 0.12-1

Note: S: Sensitive,R: Resistant, I: Intermediat, nWT: non-Wild Type, WT: Wild Type

Identification Method: MOLDI TOF

Specimen submission time: 2022.09.25 20:25 Report Release Time: 2022.10.02 11:37 Laboratory Technician: Report Reviewer: Laboratory test order time: 2022.09.25 15:43 Ordering Physician:xxx

**This report applies solely to the submitted specimen. For inquiries regarding test results, please contact the Laboratory prior to 17:00 on working days.**

**Name:**

**Gender:** **F**

**Age:** 83

**Department:**

**Patient ID:**

**Inpatient No.:**

emergency department

**Bed No.:**

**Specimen Type: Blood**

**Clinical Diagnosis:**

**Sample No.:** **221117202**

**Result:** ***Candida albicans***

Antibiotic

　Amphotericin B

Fluconazole

Itraconazole

Voriconazole

MIC AST Result Interpretation

<0.5 WT

<1 S

<0.12 S

<0.06 S

Breakpoint

2

2-4

0.5-2

Note: S: Sensitive,R: Resistant, I: Intermediat, nWT: non-Wild Type, WT: Wild Type

Identification Method: MOLDI TOF

Specimen submission time: 2022.11.17 09:57 Report Release Time: 2022.11.21 10:54 Laboratory Technician: Report Reviewer: Laboratory test order time: 2022.11.16 20:20 Ordering Physician: xxx

**This report applies solely to the submitted specimen. For inquiries regarding test results, please contact the Laboratory prior to 17:00 on working days.**

**Name:** 　 **Gender: M**

**Age:** 11

**Department: Patient ID:**

**Inpatient No.:**

pediatrics

**Bed No.:** 　 **Specimen Type: Blood**

**Clinical Diagnosis:**

**Sample No.:** **220929508**

**Result:** ***Candida tropicalis***

Antibiotic MIC

　Amphotericin <0.5 B

Fluconazole <1

AST Result Interpretation WT

S

Breakpoint 2

2-8

Note: S: Sensitive,R: Resistant, I: Intermediat, nWT: non-Wild Type, WT: Wild Type

Identification Method: MOLDI TOF

Specimen submission time: 2022.09.29 10:48 Report Release Time: 2022.10.2 11:36 Laboratory Technician: Report Reviewer: Laboratory test order time: 2022.09.29 09:10 Ordering Physician:xxx

**This report applies solely to the submitted specimen. For inquiries regarding test results, please contact the Laboratory prior to 17:00 on working days.**

**Name:**

**Gender:** **m**

**Age:** 11

**Department:**

**Patient ID:**

**Inpatient No.:**

day-care unit

**Bed No.:**

**Specimen Type: Blood**

**Clinical Diagnosis:**

**Sample No.:** **230929509**

**Result:** ***Candida tropicalis***

Antibiotic MIC

　Amphotericin <0.5 B

Fluconazole <1

AST Result Interpretation WT

S

Breakpoint

2

2-8

Note: S: Sensitive,R: Resistant, I: Intermediat, nWT: non-Wild Type, WT: Wild Type

Identification Method: MOLDI TOF

Specimen submission time:2023.09.29 10:48 Report Release Time:2023.10.02 11:36 Laboratory Technician: Report Reviewer: Laboratory test order time:2023.09:29 09:10 Ordering Physician: xxx

**This report applies solely to the submitted specimen. For inquiries regarding test results, please contact the Laboratory prior to 17:00 on working days.**

**Name:**

**Gender:** **M**

**Age:** 11

**Department:**

**Patient ID:**

**Inpatient No.:**

day-care unit

**Bed No.:**

**Specimen Type: Blood**

**Clinical Diagnosis:**

**Sample No.:** **230929509**

**Result:** ***Candida tropicalis***

Antibiotic MIC

　Amphotericin <0.5 B

Fluconazole <1

AST Result Interpretation WT

S

Breakpoint

2

2-8

Note: S: Sensitive,R: Resistant, I: Intermediat, nWT: non-Wild Type, WT: Wild Type

Identification Method: MOLDI TOF

Specimen submission time:2023.09.29 10:48 Report Release Time:2023.10.2 11:36 Laboratory Technician: Report Reviewer: Laboratory test order time:2023.09.29 09:10 Ordering Physician:xxx

**This report applies solely to the submitted specimen. For inquiries regarding test results, please contact the Laboratory prior to 17:00 on working days.**

**Name:**

**Gender:** **M**

**Age:** 11

**Department:**

**Patient ID:**

**Inpatient No.:**

day-care unit

**Bed No.:**

**Specimen Type: Blood**

**Clinical Diagnosis:**

**Sample No.:** **230929509**

**Result:** ***Candida tropicalis***

Antibiotic MIC

　Amphotericin <0.5 B

Fluconazole <1

AST Result Interpretation

WT S

Breakpoint

2 2-8

Note: S: Sensitive,R: Resistant, I: Intermediat, nWT: non-Wild Type, WT: Wild Type

Identification Method: MOLDI TOF

Specimen submission time:2023.09.29 10:48 Report Release Time:2023.10.02 11:36 Laboratory Technician: Report Reviewer: Laboratory test order time:2023.09.29 09:10 Ordering Physician: xxx

**This report applies solely to the submitted specimen. For inquiries regarding test results, please contact the Laboratory prior to 17:00 on working days.**

**Name:**

**Gender:** **M**

**Age:** 67

**Department:**

**Patient ID:**

**Inpatient No.:**

　surgical department

**Bed No.:**

**Specimen Type: Blood**

**Clinical Diagnosis:**

**Sample No.:** **231115204**

**Result:** ***Candida tropicalis***

Antibiotic MIC

　Amphotericin <0.5 B

Fluconazole <1

Itraconazole ＜0.12

Voriconazole ＜0.06

AST Result Interpretation WT

S WT

S

Breakpoint 2

2-8 0.5

0.12-1

Note: S: Sensitive,R: Resistant, I: Intermediat, nWT: non-Wild Type, WT: Wild Type

Identification Method: MOLDI TOF

Specimen submission time:2023.11.15 20:38 Report Release Time:2023.11.19 08:59 Laboratory Technician: Report Reviewer: Laboratory test order time:2023.11.15 18:47 Ordering Physician :xxx

**This report applies solely to the submitted specimen. For inquiries regarding test results, please contact the Laboratory prior to 17:00 on working days.**

**Name:**

**Gender:** **m**

**Age:** 67

**Department:**

**Patient ID:**

**Inpatient No.:**

surgical department

**Bed No.:**

**Specimen Type: Blood**

**Clinical Diagnosis:**

**Sample No.:** **231004204**

**Result:** ***Candida parapsilosis***

Antibiotic MIC

　Amphotericin ≤0.5 B

Fluconazole ≤1

Itraconazole ≤0.12

Voriconazole ≤0.06

AST Result Interpretation WT

S WT

S

Breakpoint 　1

2-8 0.5

0.125-1

Note: S: Sensitive,R: Resistant, I: Intermediat, nWT: non-Wild Type, WT: Wild Type

Identification Method: MOLDI TOF

Specimen submission time:2023.10.04 21:09 Report Release Time:2023.10.09 10:15 Laboratory Technician: Report Reviewer: Laboratory test order time:2023.10.04 18:02 Ordering Physician:xxx

**This report applies solely to the submitted specimen. For inquiries regarding test results, please contact the Laboratory prior to 17:00 on working days.**

**Name:** 　 **Gender:** **f**

**Age:** 36

**Department: Patient ID:**

**Inpatient No.:**

internal Medicine

**Bed No.:** 　 **Specimen Type: Blood**

**Clinical Diagnosis:**

**Sample No.:** **231012208**

**Result:** ***Candida tropicalis***

Antibiotic MIC

　Amphotericin <0.5 B

Fluconazole 2

Itraconazole <0.125

Voriconazole 0.125

AST Result Interpretation WT

S WT

S

Breakpoint 2

2-8 0.5

0.125-1

Note: S: Sensitive,R: Resistant, I: Intermediat, nWT: non-Wild Type, WT: Wild Type

Identification Method: MOLDI TOF

Specimen submission time:2023.10.12 21:29 Report Release Time:2023.10.16 09:25 Laboratory Technician: Report Reviewer: Laboratory test order time:2023.10.12 20:28 Ordering Physician:xxx

**This report applies solely to the submitted specimen. For inquiries regarding test results, please contact the Laboratory prior to 17:00 on working days.**

**Name:**

**Gender:** **M**

**Age:** 78

**Department:**

**Patient ID:**

**Inpatient No.:**

　internal Medic ine

**Bed No.:**

**Specimen Type: Blood**

**Clinical Diagnosis:**

**Sample No.:** **230221019**

**Result:** ***Candida norvegensis***

Antibiotic MIC AST Result Interpretation Breakpoint

　Amphotericin B

Fluconazole Itraconazole

Voriconazole

≤4

4

≤0.12

0.25

Note: S: Sensitive,R: Resistant, I: Intermediat

Identification Method: MOLDI TOF

Specimen submission time:2023.02.21 04:01 Report Release Time:2023.02.24 09:16 Laboratory Technician: Report Reviewer: Laboratory test order time:2023.02.21 02:01 Ordering Physician: xxx

**This report applies solely to the submitted specimen. For inquiries regarding test results, please contact the Laboratory prior to 17:00 on working days.**

**Name:**

**Gender:** **M**

**Age:** 35

**Department:**

**Patient ID:**

**Inpatient No.:**

　surgical department

**Bed No.:**

**Specimen Type: Blood**

**Clinical Diagnosis:**

**Sample No.:** **231021206**

**Result:** ***Candida parapsilosis***

Antibiotic MIC

　Amphotericin ＜0.5 B

Fluconazole ＜1

Itraconazole ＜0.12

Voriconazole ＜0.06

AST Result Interpretation WT

S

WT

S

Breakpoint

　1

2-8

0.5

0.125-1

Note: S: Sensitive,R: Resistant, I: Intermediat, nWT: non-Wild Type, WT: Wild Type

Identification Method: MOLDI TOF

Specimen submission time:2023.10.21 16:06 Report Release Time:2023.10.26 10:13 Laboratory Technician: Report Reviewer: Laboratory test order time:2023.10.21 09:51 Ordering Physician: xxx

**This report applies solely to the submitted specimen. For inquiries regarding test results, please contact the Laboratory prior to 17:00 on working days.**

**Name:** 　 **Gender: M**

**Age:** 57

**Department:** 　ICU **Patient ID:**

**Inpatient No.:**

**Bed No.:** 　 **Specimen Type: Blood**

**Clinical Diagnosis:**

**Sample No.:** **231022205**

**Result:** ***Candida parapsilosis***

Antibiotic MIC

　Amphotericin ＜0.5 B

Fluconazole ＜1

Itraconazole ＜0.12

Voriconazole ＜0.06

AST Result Interpretation WT

S　 WT

S

Breakpoint

1 2-8

0.5

0.125-1

Note: S: Sensitive,R: Resistant, I: Intermediat, nWT: non-Wild Type, WT: Wild Type

Identification Method: MOLDI TOF

Specimen submission time:2023.10.22.11:40 Report Release Time:2023.10.26 10:12 Laboratory Technician: Report Reviewer: Laboratory test order time:2023.10.22 10:14 Ordering Physician: xxx

**This report applies solely to the submitted specimen. For inquiries regarding test results, please contact the Laboratory prior to 17:00 on working days.**

**Name:** 　 **Gender: M**

**Age:** 70

**Department: Patient ID:**

**Inpatient No.:**

Internal Medicine

**Bed No.；** 　 **Specimen Type: Blood**

**Clinical Diagnosis:**

**Sample No.:** **231022211**

**Result:** ***Candida tropicalis***

Antibiotic MIC

　Amphotericin ＜0.5 B

Fluconazole ≥256

Itraconazole ＝4

Voriconazole ≥16

AST Result Interpretation WT

R S

nWT

S

Breakpoint

2

2-8

0.5

0.125-1

Note: S: Sensitive,R: Resistant, I: Intermediat, nWT: non-Wild Type, WT: Wild Type

Identification Method: MOLDI TOF

Specimen submission time:2023.10.22 16:41 Report Release Time:2023.10.25 09:36 Laboratory Technician: Report Reviewer: Laboratory test order time:2023.10.22 15:50 Ordering Physician: xxx

**This report applies solely to the submitted specimen. For inquiries regarding test results, please contact the Laboratory prior to 17:00 on working days.**

**Name:**

**Gender:** **m**

**Age:** 67

**Department:**

**Patient ID:**

**Inpatient No.:**

　surgical department

**Bed No.；**

**Specimen Type: Blood**

**Clinical Diagnosis:**

**Sample No.:** **231022219**

**Result:** ***Candida tropicalis***

Antibiotic MIC

　Amphotericin ＜0.5 B

Fluconazole ＜0.5

Itraconazole ＜0.12

Voriconazole ＜0.062

AST Result Interpretation WT

S

WT

S

Breakpoint

2

2-8

0.5

0.125-1

Note: S: Sensitive,R: Resistant, I: Intermediat, nWT: non-Wild Type, WT: Wild Type

Identification Method: MOLDI TOF

Specimen submission time:2023.10.22 18:21 Report Release Time:2023.10.26 10:16 Laboratory Technician: Report Reviewer: Laboratory test order time:2023.10.22 16:41 Ordering Physician:xxx

**This report applies solely to the submitted specimen. For inquiries regarding test results, please contact the Laboratory prior to 17:00 on working days.**

**Name:**

**Gender:** **m**

**Age:** 57

**Department:**

**Patient ID:**

**Inpatient No.:**

**emergency department**

**Bed No.；**

**Specimen Type: Blood**

**Clinical Diagnosis:**

**Sample No.:** **231028205**

**Result:** ***Candida parapsilosis***

Antibiotic MIC

　Amphotericin ＜0.5 B

Fluconazole ＜1

Itraconazole ＜0.12

Voriconazole ＜0.06

AST Result Interpretation

WT

S

WT

S

Breakpoint

1

2-8

0.5

0.125-1

Note: S: Sensitive,R: Resistant, I: Intermediat, nWT: non-Wild Type, WT: Wild Type

Identification Method: MOLDI TOF

Specimen submission time:2023.10.28 05:26 Report Release Time:2023.11.1 08:44 Laboratory Technician: Report Reviewer: Laboratory test order time:2023.10.27 21:32 Ordering Physician:xxx

**This report applies solely to the submitted specimen. For inquiries regarding test results, please contact the Laboratory prior to 17:00 on working days.**

**Name:** 　 **Gender: m**

**Age:** 65

**Department:** 　ICU **Patient ID:**

**Inpatient No.:**

**Bed No.；** 　 **Specimen Type: Blood**

**Clinical Diagnosis:**

**Sample No.:** **231028205**

**Result:** ***Candida tropicalis***

Antibiotic MIC

　Amphotericin ＜0.5 B

Fluconazole ≤0.5

Itraconazole ≤0.12

Voriconazole ≤0.06

AST Result Interpretation WT

S

WT

S

Breakpoint

　2

2-8

0.5

0.125-1

Note: S: Sensitive,R: Resistant, I: Intermediat, nWT: non-Wild Type, WT: Wild Type

Identification Method: MOLDI TOF

Specimen submission time:2023.10.28 18:40 Report Release Time:2023.11.02 10:21 Laboratory Technician: Report Reviewer: Laboratory test order time:2023.10.28 16:47 Ordering Physician:xxx

**This report applies solely to the submitted specimen. For inquiries regarding test results, please contact the Laboratory prior to 17:00 on working days.**

**Name:**

**Gender:** **M**

**Age:** 67

**Department:**

**Patient ID:**

**Inpatient No.:**

**Surgical Department**

**Bed No.:**

**Specimen Type: Blood**

**Clinical Diagnosis:**

**Sample No.:** **241104208**

**Result:** ***Candida tropicalis***

Antibiotic

　Amphotericin B

Fluconazole

Itraconazole

Voriconazole

MIC AST Result Interpretation

<0.5 WT

≤0.5 S

≤0.12 WT

≤0.06 2 S

Breakpoint

　2

2-8

0.5

0.125-1

Note: S: Sensitive,R: Resistant, I: Intermediat, nWT: non-Wild Type, WT: Wild Type

Identification Method: MOLDI TOF

Specimen submission time:2024.11.04 20:14 Report Release Time:2024.11.08 9:39 Laboratory Technician: Report Reviewer: Laboratory test order time:2024.11.04 10:46 Ordering Physician: xxx

**This report applies solely to the submitted specimen. For inquiries regarding test results, please contact the Laboratory prior to 17:00 on working days.**

**Name:**

**Gender:** **M**

**Age:** 67

**Department:**

**Patient ID:**

**Inpatient No.:**

**Surgical Department**

**Bed No.:**

**Specimen Type:Blood**

**Clinical Diagnosis:**

**Sample No.:** **241105512**

**Result:** ***Candida tropicalis***

Antibiotic

　Amphotericin B

Fluconazole

Itraconazole

Voriconazole

MIC AST Result Interpretation

<0.5 WT

≤0.5 S

≤0.12 WT

≤0.062 S

Breakpoint

2

2-8

0.5

0.125-1

Note: S: Sensitive,R: Resistant, I: Intermediat, nWT: non-Wild Type, WT: Wild Type

Identification Method: MOLDI TOF

Specimen submission time:2024.11.05 13:06 Report Release Time:2024.11.07 07:34 Laboratory Technician: Report Reviewer: Laboratory test order time:2024.11.05 08:24 Ordering Physician:xxx

**This report applies solely to the submitted specimen. For inquiries regarding test results, please contact the Laboratory prior to 17:00 on working days.**

**Name:**

**Gender:** **M**

**Age:** 67

**Department:**

**Patient ID:**

**Inpatient No.:**

**Surgical Department**

**Bed No.:**

**Specimen Type:Blood**

**Clinical Diagnosis:**

**Sample No.:** **241006205**

**Result:** ***Candida parapsilosis***

Antibiotic

　Amphotericin B

Fluconazole

Itraconazole

Voriconazole

MIC AST Result Interpretation

≤0.5 WT

≤1 S

≤0.12 WT

≤0.06 S

Breakpoint

1

2-8

0.5

0.125-1

Note: S: Sensitive,R: Resistant, I: Intermediat, nWT: non-Wild Type, WT: Wild Type

Identification Method: MOLDI TOF

Specimen submission time:2024.10.06 16:57 Report Release Time:2024.10.09 13:45 Laboratory Technician: Report Reviewer: Laboratory test order time:2024.10.06 11:07 Ordering Physician:xxx

**This report applies solely to the submitted specimen. For inquiries regarding test results, please contact the Laboratory prior to 17:00 on working days.**

**Name:**

**Gender:** **F**

**Age:** 83

**Department:**

**Patient ID:**

**Inpatient No.:**

emergency department

**Bed No.:**

**Specimen Type:Blood**

**Clinical Diagnosis:**

**Sample No.:** **241113207**

**Result:** ***Candida albicans***

Antibiotic

　Amphotericin B

Fluconazole

MIC AST Result Interpretation

<0.5 WT

<1 S

Breakpoint

2

2-4

Itraconazole <0.12

Voriconazole <0.06 S 0.5-2

Note: S: Sensitive,R: Resistant, I: Intermediat, nWT: non-Wild Type, WT: Wild Type

Identification Method: MOLDI TOF

Specimen submission time:2024.11.13 20:27 Report Release Time:2024.11.18 10:05 Laboratory Technician: Report Reviewer: Laboratory test order time:2024.11.13 19:31 Ordering Physician: xxx

**This report applies solely to the submitted specimen. For inquiries regarding test results, please contact the Laboratory prior to 17:00 on working days.**

**Name:**

**Gender:** **M**

**Age:** 67

**Department:**

**Patient ID:**

**Inpatient No.:**

**Surgical Department**

**Bed No.:**

**Specimen Type:Blood**

**Clinical Diagnosis:**

**Sample No.:** **241113203**

**Result:** ***Candida parapsilosis***

Antibiotic

　Amphotericin B

Fluconazole

Itraconazole

Voriconazole

MIC AST Result Interpretation

<0.5 WT

<1 S

<0.12 WT

<0.06 S

Breakpoint

1

2-8

0.5

0.12-1

Note: S: Sensitive,R: Resistant, I: Intermediat, nWT: non-Wild Type, WT: Wild Type

Identification Method: MOLDI TOF

Specimen submission time:2024.11.13 15:47 Report Release Time:2024.11.18 10:03 Laboratory Technician: Report Reviewer: Laboratory test order time:2024.11.13 13:02 Ordering Physician:xxx

**This report applies solely to the submitted specimen. For inquiries regarding test results, please contact the Laboratory prior to 17:00 on working days.**

**Name:** 　 **Gender:** **f**

**Age:** 83

**Department:** ICU **Patient ID:**

**Inpatient No.:**

**Bed No.:** 　 **Specimen Type: Blood**

**Clinical Diagnosis:**

**Sample No.:** **240925207**

**Result:** ***Candida albicans***

Antibiotic

　Amphotericin B

Fluconazole

MIC AST Result Interpretation

<0.5 WT

≤1 S

Breakpoint

2

2-8

Itraconazole ≤0.125

Voriconazole ≤0.06 S 0.12-1

Note: S: Sensitive,R: Resistant, I: Intermediat, nWT: non-Wild Type, WT: Wild Type

Identification Method: MOLDI TOF

Specimen submission time:2024.9.25 20:25 Report Release Time:2024.10,2 11:37 Laboratory Technician: Report Reviewer: Laboratory test order time:2024.09.25 15:43 Ordering Physician:xxx

**This report applies solely to the submitted specimen. For inquiries regarding test results, please contact the Laboratory prior to 17:00 on working days.**

**Name:**

**Gender:** **m**

**Age:** 70

**Department:**

**Patient ID:**

**Inpatient No.:**

emergency department

**Bed No.:**

**Specimen Type:Blood**

**Clinical Diagnosis:**

**Sample No.:** **241121205**

**Result:** ***Candida tropicalis***

Antibiotic

　Amphotericin B

Fluconazole

Itraconazole

Voriconazole

MIC AST Result Interpretation

<0.5 WT

≤0.5 S

≤0.12 WT

≤0.06 S

Breakpoint

2

2-8 0.5

0.125-1

Note: S: Sensitive,R: Resistant, I: Intermediat, nWT: non-Wild Type, WT: Wild Type

Identification Method: MOLDI TOF

Specimen submission time: 2024.11.21 20:39 Report Release Time:2024.11.24 09:33 Laboratory Technician: Report Reviewer: Laboratory test order time: 2024.11.21 17:18 Ordering Physician:xxx

**This report applies solely to the submitted specimen. For inquiries regarding test results, please contact the Laboratory prior to 17:00 on working days.**

**Name:**

**Gender:** **M**

**Age:** 70

**Department:**

**Patient ID:**

**Inpatient No.:**

emergency department

**Bed No.:**

**Specimen Type: Blood**

**Clinical Diagnosis:**

**Sample No.:** **241123204**

**Result:** ***Candida tropicalis***

Antibiotic

　Amphotericin B

Fluconazole

Itraconazole

Voriconazole

MIC AST Result Interpretation

<0.5 WT

≤0.5 S

≤0.12 WT

≤0.06 S

Breakpoint

2

2-8 0.5

0.125-1

Note: S: Sensitive,R: Resistant, I: Intermediat, nWT: non-Wild Type, WT: Wild Type

Identification Method: MOLDI TOF

Specimen submission time: 2024.11.23 16:50 Report Release Time:2024.11.26 09:45 Laboratory Technician: Report Reviewer: Laboratory test order time:2024.11.23 15:19 Ordering Physician:xxx

**This report applies solely to the submitted specimen. For inquiries regarding test results, please contact the Laboratory prior to 17:00 on working days.**

**Name:**

**Gender:** **M**

**Age:** 70

**Department:**

**Patient ID:**

**Inpatient No.:**

emergency department

**Bed No.:**

**Specimen Type: Blood**

**Clinical Diagnosis:**

**Sample No.:** **241123515**

**Result:** ***Candida tropicalis***

Antibiotic

　Amphotericin B

Fluconazole

Itraconazole

Voriconazole

MIC AST Result Interpretation

<0.5 WT

≤0.5 S

≤0.12 WT

≤0.06 S

Breakpoint

2

2-8 0.5

0.125-1

Note: S: Sensitive,R: Resistant, I: Intermediat, nWT: non-Wild Type, WT: Wild Type

Identification Method: MOLDI TOF

Specimen submission time:2024.11.23 14:17 Report Release Time:2024.11.26 9:45 Laboratory Technician: Report Reviewer: Laboratory test order time:2024.11.23 08:52 Ordering Physician: xxx

**This report applies solely to the submitted specimen. For inquiries regarding test results, please contact the Laboratory prior to 17:00 on working days.**

**Name:**

**Gender:** **M**

**Age:** 67

**Department:**

**Patient ID:**

**Inpatient No.:**

surgical department

**Bed No.:**

**Specimen Type: Blood**

**Clinical Diagnosis:**

**Sample No.:** **241124204**

**Result:** ***Candida parapsilosis***

Antibiotic

　Amphotericin B

Fluconazole

Itraconazole

Voriconazole

MIC AST Result Interpretation

<0.5 WT

<1 S

<0.12 WT

<0.06 S

Breakpoint

1

2-8

0.5

0.12-1

Note: S: Sensitive,R: Resistant, I: Intermediat, nWT: non-Wild Type, WT: Wild Type

Identification Method: MOLDI TOF

Specimen submission time:2024.11.24 08:03 Report Release Time:2024.11.28 07:30 Laboratory Technician: Report Reviewer: Laboratory test order time:2024.11.23 20:00 Ordering Physician: xxx

**This report applies solely to the submitted specimen. For inquiries regarding test results, please contact the Laboratory prior to 17:00 on working days.**

**Name:** 　 **Gender:** **F**

**Age:** 83

**Department:** ICU **Patient ID:**

**Inpatient No.:**

**Bed No.:** 　 **Specimen Type: Blood**

**Clinical Diagnosis:**

**Sample No.:** **241130202**

**Result:** ***Candida albicans***

Antibiotic

　Amphotericin B

Fluconazole

MIC AST Result Interpretation

<0.5 WT

<1 S

Breakpoint

2

2-4

Itraconazole <0.12

Voriconazole <0.06 S 0.5-2

Note: S: Sensitive,R: Resistant, I: Intermediat, nWT: non-Wild Type, WT: Wild Type

Identification Method: MOLDI TOF

Specimen submission time:2024.11.30 00:45 Report Release Time:2024.12.03 10:01 Laboratory Technician: Report Reviewer: Laboratory test order time:2024.11.29 22:21 Ordering Physician:xxx

**This report applies solely to the submitted specimen. For inquiries regarding test results, please contact the Laboratory prior to 17:00 on working days.**

**Name:** 　 **Gender: m**

**Age:** 　61

**Department: Patient ID:**

**Inpatient No.:**

internal Medicine

**Bed No.:** 　 **Specimen Type: Blood**

**Clinical Diagnosis:**

**Sample No.:** **241123093**

**Result:** ***Candida guilliermondii***

Antibiotic

　Amphotericin B

Fluconazole

MIC AST Result Interpretation

≤0.5

≥128

Breakpoint

Itraconazole ≥4

Voriconazole ≥8

Note: S: Sensitive,R: Resistant, I: Intermediat, nWT: non-Wild Type, WT: Wild Type

Identification Method: MOLDI TOF

Specimen submission time:2024.12.23 16:34 Report Release Time:2024.11.28 9:18 Laboratory Technician: Report Reviewer: Laboratory test order time:2024.11.23 12:44 Ordering Physician:xxx

**This report applies solely to the submitted specimen. For inquiries regarding test results, please contact the Laboratory prior to 17:00 on working days.**

**Name:** 　 **Gender:** **f**

**Age:** 88

**Department:** 　EICU **Patient ID:**

**Inpatient No.:**

**Bed No.:** 　 **Specimen Type: Blood**

**Clinical Diagnosis:**

**Sample No.:** **241217219**

**Result:** ***Candida tropicalis***

Antibiotic

　Amphotericin B

Fluconazole

Itraconazole

Voriconazole

MIC AST Result Interpretation

<0.5 WT

8 R

0.5 WT

0.125　 S

Breakpoint

2

2-8 0.5

0.125-1

Note: S: Sensitive,R: Resistant, I: Intermediat, nWT: non-Wild Type, WT: Wild Type

Identification Method: MOLDI TOF

Specimen submission time:2024.12.17 23:17 Report Release Time:2024.12.21 10:17 Laboratory Technician: Report Reviewer: Laboratory test order time:2024.12.17 22:01 Ordering Physician: xxx

**This report applies solely to the submitted specimen. For inquiries regarding test results, please contact the Laboratory prior to 17:00 on working days.**

**Name:** 　 **Gender: M**

**Age:** 8D

**Department: Patient ID:**

**Inpatient No.:**

Neonatology

**Bed No.:** 　 **Specimen Type: Blood**

**Clinical Diagnosis:**

**Sample No.:** **241221512**

**Result:** ***Candida parapsilosis***

Antibiotic

　Amphotericin B

Fluconazole

Itraconazole

Voriconazole

MIC AST Result Interpretation

≤0.5 WT

≤1 S

≤0.12 S

≤0.06　 S

Breakpoint

1

2-8

0.5

0.125-1

Note: S: Sensitive,R: Resistant, I: Intermediat, nWT: non-Wild Type, WT: Wild Type

Identification Method: MOLDI TOF

Specimen submission time:2024.12.21 14:56 Report Release Time:2024.12.25 09:48 Laboratory Technician: Report Reviewer: Laboratory test order time:2024.12.21 12:27 Ordering Physician: xxx

**This report applies solely to the submitted specimen. For inquiries regarding test results, please contact the Laboratory prior to 17:00 on working days.**

**Name:** 　 **Gender: M**

**Age:** 8D

**Department: Patient ID:**

**Inpatient No.:**

Neonatology

**Bed No.:** 　 **Specimen Type: Blood**

**Clinical Diagnosis:**

**Sample No.:** **241221513**

**Result:** ***Candida parapsilosis***

Antibiotic

　Amphotericin B

Fluconazole

Itraconazole

Voriconazole

MIC AST Result Interpretation

≤0.5 WT

≤1 S

≤0.12 WT

≤0.06　 S

Breakpoint

1

2-8

0.5

0.125-1

Note: S: Sensitive,R: Resistant, I: Intermediat, nWT: non-Wild Type, WT: Wild Type

Identification Method: MOLDI TOF

Specimen submission time:2024.12.21 14:26 Report Release Time:2024.12.25 09:48 Laboratory Technician: Report Reviewer: Laboratory test order time:2024.12.21 12:37 Ordering Physician: xxx

**This report applies solely to the submitted specimen. For inquiries regarding test results, please contact the Laboratory prior to 17:00 on working days.**

**Name:** 　 **Gender: M**

**Age:** 33

**Department:** 　ICU **Patient ID:**

**Inpatient No.:**

**Bed No.:** 　 **Specimen Type: Blood**

**Clinical Diagnosis:**

**Sample No.:** **241229202**

**Result:** ***Candida parapsilosis***

Antibiotic

　Amphotericin B

Fluconazole

Itraconazole

Voriconazole

MIC AST Result Interpretation

≤0.5 WT

≤1 S

≤0.12 WT

≤0.06　 S

Breakpoint

1

2-8

0.5

0.125-1

Note: S: Sensitive,R: Resistant, I: Intermediat, nWT: non-Wild Type, WT: Wild Type

Identification Method: MOLDI TOF

Specimen submission time:2024.12.29 01:47 Report Release Time:2025.1.03 10:57 Laboratory Technician: Report Reviewer: Laboratory test order time:2024.12.28 21:22 Ordering Physician:xxx

**This report applies solely to the submitted specimen. For inquiries regarding test results, please contact the Laboratory prior to 17:00 on working days.**

**Name:** 　 **Gender: M**

**Age:** 33

**Department:** 　ICU **Patient ID:**

**Inpatient No.:**

**Bed No.:** 　 **Specimen Type: Blood**

**Clinical Diagnosis:**

**Sample No.:** **241229206**

**Result:** ***Candida parapsilosis***

Antibiotic

　Amphotericin B

Fluconazole

Itraconazole

Voriconazole

MIC AST Result Interpretation

≤0.5 WT

≤1 S

≤0.12 WT

≤0.06　 S

Breakpoint

1

2-8

0.5

0.125-1

Note: S: Sensitive,R: Resistant, I: Intermediat, nWT: non-Wild Type, WT: Wild Type

Identification Method: MOLDI TOF

Specimen submission time:2024.12.29 10:55 Report Release Time:2025.01.03 10:56 Laboratory Technician: Report Reviewer: Laboratory test order time:2024.12.29 10.02 Ordering Physician:xxx

**This report applies solely to the submitted specimen. For inquiries regarding test results, please contact the Laboratory prior to 17:00 on working days.**

**Name:** 　 **Gender: m**

**Age:** 69

**Department:** 　ICU **Patient ID:**

**Inpatient No.:**

**Bed No.:** 　 **Specimen Type: Blood**

**Clinical Diagnosis:**

**Sample No.:** **240212208**

**Result:** ***Candida albicans***

Antibiotic

　Amphotericin B

Fluconazole

MIC AST Result Interpretation

<0.5 WT

<1 S

Breakpoint

2

2-4

Itraconazole <0.12

Voriconazole <0.06　 S 0.5-2

Note: S: Sensitive,R: Resistant, I: Intermediat, nWT: non-Wild Type, WT: Wild Type

Identification Method: MOLDI TOF

Specimen submission time:2024.2.12 23:26 Report Release Time:2024.2.16 10:26 Laboratory Technician: Report Reviewer: Laboratory test order time:2024.2.12 13:46 Ordering Physician:xxx

**This report applies solely to the submitted specimen. For inquiries regarding test results, please contact the Laboratory prior to 17:00 on working days.**

**Name:** 　 **Gender: M**

**Age:** 68

**Department:** 　EICU **Patient ID:**

**Inpatient No.:**

**Bed No.:** 　 **Specimen Type: Blood**

**Clinical Diagnosis:**

**Sample No.:** **240225214**

**Result:** ***Candida glabrata***

Antibiotic

　Amphotericin B

Fluconazole

Itraconazole

Voriconazole

MIC AST Result Interpretation

<0.5 WT

=32 SDD

=4 nWT

=8 nWT

Breakpoint

2

8-64

4

0.25

Note: S: Sensitive,R: Resistant, I: Intermediat, nWT: non-Wild Type, WT: Wild Type

Identification Method: MOLDI TOF

Specimen submission time:2024.2.25 15:26 Report Release Time:2024.3.03 10:06 Laboratory Technician: Report Reviewer: Laboratory test order time:2024.2.25 14:58 Ordering Physician:xxx

**This report applies solely to the submitted specimen. For inquiries regarding test results, please contact the Laboratory prior to 17:00 on working days.**

**Name:** 　 **Gender: m**

**Age:** 68

**Department:** 　EICU **Patient ID:**

**Inpatient No.:**

**Bed No.:** 　 **Specimen Type: Blood**

**Clinical Diagnosis:**

**Sample No.:** **240228202**

**Result:** ***Candida glabrata***

Antibiotic

　Amphotericin B

Fluconazole

Itraconazole

Voriconazole

MIC AST Result Interpretation

<0.5 WT

=32 SDD

=4 nWT

=8 nWT

Breakpoint

2

8-64

4

0.25

Note: S: Sensitive,R: Resistant, I: Intermediat

Identification Method: MOLDI TOF

Specimen submission time:2024.2.28 8:07 Report Release Time:2024.3.03 10:16 Laboratory Technician: Report Reviewer: Laboratory test order time:2024.2.28 6:39 Ordering Physician:xxx

**This report applies solely to the submitted specimen. For inquiries regarding test results, please contact the Laboratory prior to 17:00 on working days.**

**Name:** 　 **Gender: m**

**Age:** 73

**Department:** 　ICU **Patient ID:**

**Inpatient No.:**

**Bed No.:** 　 **Specimen Type: Blood**

**Clinical Diagnosis:**

**Sample No.:** **240328206**

**Result:**

***Candida parapsilosis***

Antibiotic

　Amphotericin B

Fluconazole

Itraconazole

Voriconazole

MIC AST Result Interpretation

≤0.5 WT

=16 R

=16 nWT

<0.06 S

Breakpoint

1

2-8

0.5

0.12-1

Note: S: Sensitive,R: Resistant, I: Intermediat, nWT: non-Wild Type, WT: Wild Type

Identification Method: MOLDI TOF

Specimen submission time:2024.3.28 20:55 Report Release Time:2024.04.04 9:32 Laboratory Technician: Report Reviewer: Laboratory test order time:2024.3.28 17:56 Ordering Physician:xxx

**This report applies solely to the submitted specimen. For inquiries regarding test results, please contact the Laboratory prior to 17:00 on working days.**

**Name:** 　 **Gender: m**

**Age:** 68

**Department:** 　EICU **Patient ID:**

**Inpatient No.:**

**Bed No.:** 　 **Specimen Type: Blood**

**Clinical Diagnosis:**

**Sample No.:** **240410201**

**Result:**

***Candida parapsilosis***

Antibiotic

　Amphotericin B

Fluconazole

Itraconazole

Voriconazole

MIC AST Result Interpretation

<0.5 WT

<1 S

<0.12 WT

<0.06 S

Breakpoint

1

2-8

0.5

0.125-1

Note: S: Sensitive,R: Resistant, I: Intermediat, nWT: non-Wild Type, WT: Wild Type

Identification Method: MOLDI TOF

Specimen submission time:2024.3.29 18:54 Report Release Time: 2024.4.03 11:53 Laboratory Technician: Report Reviewer: Laboratory test order time:2024.3.29 16:28 Ordering Physician:xxx

**This report applies solely to the submitted specimen. For inquiries regarding test results, please contact the Laboratory prior to 17:00 on working days.**

**Name:** 　 **Gender:** **f**

**Age:** 73

**Department:** 　EICU **Patient ID:**

**Inpatient No.:**

**Bed No.:** 　 **Specimen Type: Blood**

**Clinical Diagnosis:**

**Sample No.:** **240408204**

**Result:**

***Candida parapsilosis***

Antibiotic

　Amphotericin B

Fluconazole

Itraconazole

Voriconazole

MIC AST Result Interpretation

<0.5 WT

=2 S

<0.125 WT

<0.12 S

Breakpoint

1

2-8

0.5

0.125-1

Note: S: Sensitive,R: Resistant, I: Intermediat, nWT: non-Wild Type, WT: Wild Type

Identification Method: MOLDI TOF

Specimen submission time:2024.4.08 10:46 Report Release Time:2024.4.11 9:20 Laboratory Technician: Report Reviewer: Laboratory test order time:2024.4.08 10:09 Ordering Physician:xxx

**This report applies solely to the submitted specimen. For inquiries regarding test results, please contact the Laboratory prior to 17:00 on working days.**

**Name:** 　 **Gender:** **f**

**Age:** 64

**Department:** 　ICU **Patient ID:**

**Inpatient No.:**

**Bed No.:** 　 **Specimen Type: Blood**

**Clinical Diagnosis:**

**Sample No.:** **240410201**

**Result:**

***Candida albicans***

Antibiotic

　Amphotericin B

Fluconazole

MIC AST Result Interpretation

<0.5 WT

<1 S

Breakpoint

2

2-8

Itraconazole <0.12

Voriconazole <0.06 S 0.12-1

Note: S: Sensitive,R: Resistant, I: Intermediat, nWT: non-Wild Type, WT: Wild Type

Identification Method: MOLDI TOF

Specimen submission time:2024.4.10 8:11 Report Release Time:2024.4.15 10:01 Laboratory Technician: Report Reviewer: Laboratory test order time:2024.4.10 4:29 Ordering Physician:xxx

**This report applies solely to the submitted specimen. For inquiries regarding test results, please contact the Laboratory prior to 17:00 on working days.**

**Name:** 　 **Gender:** **f**

**Age:** 64

**Department:** 　ICU **Patient ID:**

**Inpatient No.:**

**Bed No.:** 　 **Specimen Type: Blood**

**Clinical Diagnosis:**

**Sample No.:** **240410212**

**Result:**

***Candida albicans***

Antibiotic

　Amphotericin B

Fluconazole

MIC AST Result Interpretation

<0.5 WT

<1 S

Breakpoint

2

2-8

Itraconazole <0.12

Voriconazole <0.06 S 0.125-1

Note: S: Sensitive,R: Resistant, I: Intermediat, nWT: non-Wild Type, WT: Wild Type

Identification Method: MOLDI TOF

Specimen submission time:2024.4.10 17:04 Report Release Time:2024.4.15 9:26 Laboratory Technician: Report Reviewer: Laboratory test order time:2024.4.10 13:11 Ordering Physician:xxx

**This report applies solely to the submitted specimen. For inquiries regarding test results, please contact the Laboratory prior to 17:00 on working days.**

**Name:**

**Gender:** **m**

**Age:** 74

**Department:**

**Patient ID:**

**Inpatient No.:**

emergency department

**Bed No.:**

**Specimen Type: Blood**

**Clinical Diagnosis:**

**Sample No.:** **240412208**

**Result:** ***Candida tropicalis***

Antibiotic

　Amphotericin B

Fluconazole

Itraconazole

MIC AST Result Interpretation

<0.5 WT

4 I

<0.5 WT

Breakpoint

2

2-8

0.5

Voriconazole <0.25 0.125-1

Note: S: Sensitive,R: Resistant, I: Intermediat, nWT: non-Wild Type, WT: Wild Type

Identification Method: MOLDI TOF

Specimen submission time: 2024.4.12 22:10 Report Release Time:2024.4.15 9:26 Laboratory Technician: Report Reviewer: Laboratory test order time:2024.4.12 22:16 Ordering Physician:xxx

**This report applies solely to the submitted specimen. For inquiries regarding test results, please contact the Laboratory prior to 17:00 on working days.**

**Name:**

**Gender:** **m**

**Age:** 65

**Department:**

**Patient ID:**

**Inpatient No.:**

　surgical department

**Bed No.:**

**Specimen Type: Blood**

**Clinical Diagnosis:**

**Sample No.:** **240418203**

**Result:** ***Candida parapsilosis***

Antibiotic

　Amphotericin B

Fluconazole

Itraconazole

Voriconazole

MIC AST Result Interpretation

<0.5 WT

=2 S

<0.12 WT

<0.06　 S

Breakpoint

1

2-8

0.5

0.125-1

Note: S: Sensitive,R: Resistant, I: Intermediat

Identification Method: MOLDI TOF

Specimen submission time:2024.4.18 13:44 Report Release Time:2024.4 22 :47 Laboratory Technician: Report Reviewer: Laboratory test order time:2024.4.18 12:59 Ordering Physician: xxx

**This report applies solely to the submitted specimen. For inquiries regarding test results, please contact the Laboratory prior to 17:00 on working days.**

**Name:**

**Gender:** **M**

**Age:** 65

**Department:**

**Patient ID:**

**Inpatient No.:**

　surgical department

**Bed No.:**

**Specimen Type: Blood**

**Clinical Diagnosis:**

**Sample No.:** **240419205**

**Result:** ***Candida parapsilosis***

Antibiotic

　Amphotericin B

Fluconazole

Itraconazole

Voriconazole

MIC AST Result Interpretation

<0.5 WT

=2 S

<0.12 WT

<0.06　 S

Breakpoint

1

2-8

0.5

0.125-1

Note: S: Sensitive,R: Resistant, I: Intermediat, nWT: non-Wild Type, WT: Wild Type

Identification Method: MOLDI TOF

Specimen submission time:2024.4.19 14:43 Report Release Time:2024.4.23 10:55 Laboratory Technician: Report Reviewer: Laboratory test order time:2024.4.19 13:38 Ordering Physician:xxx

**This report applies solely to the submitted specimen. For inquiries regarding test results, please contact the Laboratory prior to 17:00 on working days.**

**Name:**

**Gender:** **m**

**Age:** 65

**Department:**

**Patient ID:**

**Inpatient No.:**

　surgical department

**Bed No.:**

**Specimen Type: Blood**

**Clinical Diagnosis:**

**Sample No.:** **240420203**

**Result:** ***Candida parapsilosis***

Antibiotic

　Amphotericin B

Fluconazole

Itraconazole

Voriconazole

MIC AST Result Interpretation

<0.5 WT

<1 S

<0.12 WT

<0.06　 S

Breakpoint

1

2-8

0.5

0.125-1

Note: S: Sensitive,R: Resistant, I: Intermediat, nWT: non-Wild Type, WT: Wild Type

Identification Method: MOLDI TOF

Specimen submission time:2024.4.20 9:47 Report Release Time:2024.4.24 10:10 Laboratory Technician: Report Reviewer: Laboratory test order time:2024.4.20 9:01 Ordering Physician:xxx

**This report applies solely to the submitted specimen. For inquiries regarding test results, please contact the Laboratory prior to 17:00 on working days.**

**Name:** 　 **Gender:** **f**

**Age:** 73

**Department:** EICU　 **Patient ID:**

**Inpatient No.:**

**Bed No.:** 　 **Specimen Type: Blood**

**Clinical Diagnosis:**

**Sample No.:** **240421209**

**Result:** ***Candida parapsilosis***

Antibiotic

　Amphotericin B

Fluconazole

Itraconazole

Voriconazole

MIC AST Result Interpretation

<0.5 WT

=2 S

=0.125 WT

=0.12 S

Breakpoint

1

2-8

0.5

0.125-1

Note: S: Sensitive,R: Resistant, I: Intermediat, nWT: non-Wild Type, WT: Wild Type

Identification Method: MOLDI TOF

Specimen submission time:2024.4.21 16:23 Report Release Time:2024.4.27 9:35 Laboratory Technician: Report Reviewer: Laboratory test order time:2024.4.21 15:22 Ordering Physician:xxx

**This report applies solely to the submitted specimen. For inquiries regarding test results, please contact the Laboratory prior to 17:00 on working days.**

**Name:**

**Gender:** **M**

**Age:** 65

**Department:**

**Patient ID:**

**Inpatient No.:**

　surgical department

**Bed No.:**

**Specimen Type: Blood**

**Clinical Diagnosis:**

**Sample No.:** **240525202**

**Result:** ***Candida parapsilosis***

Antibiotic

　Amphotericin B

Fluconazole

Itraconazole

Voriconazole

MIC AST Result Interpretation

<0.5 WT

<1 S

<0.12 WT

<0.06 S

Breakpoint

1

2-8

0.5

0.125-1

Note: S: Sensitive,R: Resistant, I: Intermediat

Identification Method: MOLDI TOF

Specimen submission time: 2024.05.25 14:04 Report Release Time:2024.05.29 08:47 Laboratory Technician: Report Reviewer: Laboratory test order time: 2024.05.25 10:52 Ordering Physician: xxx

**This report applies solely to the submitted specimen. For inquiries regarding test results, please contact the Laboratory prior to 17:00 on working days.**

**Name:**

**Gender:** **m**

**Age:** 65

**Department:**

**Patient ID:**

**Inpatient No.:**

surgical department

**Bed No.:**

**Specimen Type: Blood**

**Clinical Diagnosis:**

**Sample No.:** **24061212**

**Result:** ***Candida parapsilosis***

Antibiotic

　Amphotericin B

Fluconazole

Itraconazole

Voriconazole

MIC AST Result Interpretation

≤4

≤1 S

≤0.12 WT

≤0.06　 S

Breakpoint

1

2-8

0.5

0.125-1

Note: S: Sensitive,R: Resistant, I: Intermediat, nWT: non-Wild Type, WT: Wild Type

Identification Method: MOLDI TOF

Specimen submission time: 2024.06.11 22:42 Report Release Time: 2024.06.15 10:22 Laboratory Technician: Report Reviewer: Laboratory test order time: 2024.06.11 19.25 Ordering Physician: xxx

**This report applies solely to the submitted specimen. For inquiries regarding test results, please contact the Laboratory prior to 17:00 on working days.**

**Name:**

**Gender:** **m**

**Age:** 48

**Department:**

**Patient ID:**

**Inpatient No.:**

surgical department

**Bed No.:**

**Specimen Type: Blood**

**Clinical Diagnosis:**

**Sample No.:** **240616203**

**Result:** ***Candida parapsilosis***

Antibiotic

　Amphotericin B

Fluconazole

Itraconazole

Voriconazole

MIC AST Result Interpretation

0.5　 WT

1 S

0.125 WT

0.06 S

Breakpoint

1

2-8

0.5

0.125-1

Note: S: Sensitive,R: Resistant, I: Intermediat, nWT: non-Wild Type, WT: Wild Type

Identification Method: MOLDI TOF

Specimen submission time: 2024.06.16 09:45 Report Release Time: 2024.06.20 09:51 Laboratory Technician: Report Reviewer: Laboratory test order time: 2024.06.15 15:05Ordering Physician:xxx

**This report applies solely to the submitted specimen. For inquiries regarding test results, please contact the Laboratory prior to 17:00 on working days.**

**Name:** 　 **Gender: m**

**Age:** 60

**Department: Patient ID:**

**Inpatient No.:**

internal Medicine

**Bed No.:** 　 **Specimen Type: Blood**

**Clinical Diagnosis:**

**Sample No.:** **240625201**

**Result:** ***Candida tropicalis***

Antibiotic

　Amphotericin B

Fluconazole

Itraconazole

Voriconazole

MIC AST Result Interpretation

<0.5 WT

≥256 R

=4 nWT

≥16 R

Breakpoint

2

2-8

0.5

0.125-1

Note: S: Sensitive,R: Resistant, I: Intermediat, nWT: non-Wild Type, WT: Wild Type

Identification Method: MOLDI TOF

Specimen submission time: 2024.06.25 10:12 Report Release Time: 2024.06.28 09.28 Laboratory Technician: Report Reviewer: Laboratory test order time: 2024.06.25 08.03 Ordering Physician:xxx

**This report applies solely to the submitted specimen. For inquiries regarding test results, please contact the Laboratory prior to 17:00 on working days.**
